# Palladium-Catalyzed Allylic C–F Bond Functionalization of Pentafluoroethyl Alkenes with Heteroatom Nucleophiles

**DOI:** 10.1021/acs.orglett.5c03217

**Published:** 2025-09-01

**Authors:** Zhengjie Fu, Gavin Chit Tsui

**Affiliations:** † Department of Chemistry, 26451 The Chinese University of Hong Kong, Shatin, New Territories, Hong Kong SAR, China; ‡ Shanghai-Hong Kong Joint Laboratory in Chemical Synthesis, The Chinese University of Hong Kong, Shatin, New Territories, Hong Kong SAR, China

## Abstract

A diastereoselective Pd-catalyzed allylic C–F bond functionalization of pentafluoroethyl alkenes is described. Heteroatom nucleophiles including amines, alcohols, and thiols can be employed to construct new C–N, C–O, or C–S bonds with concomitant cleavage of an allylic C–F bond. Both 1,1- and 1,2-disubstituted pentafluoroalkenes can be utilized to synthesize novel tetra- and trisubstituted alkenes containing an sp^2^-carbon connected to both F and CF_3_ in good to excellent diastereoselectivities.

## Graphical abstract

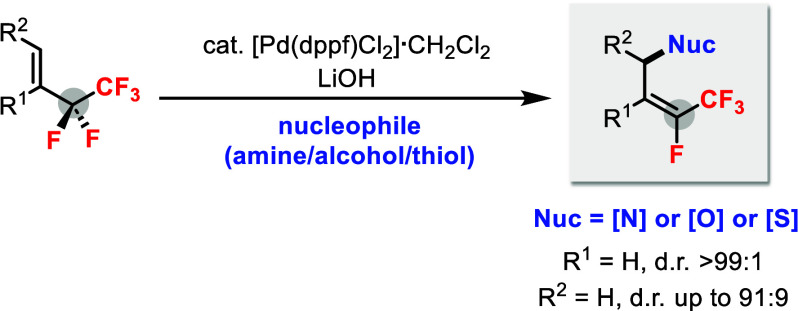

The introduction of fluorine or fluorine-containing groups into nonfluorinated precursors is nowadays standard procedure for synthesizing value-added organofluorine compounds.^1^ An equally important yet less intuitive approach also exists where selective cleavage and functionalization of C–F bonds in poly-/perfluorinated molecules gives access to partially fluorinated compounds. This approach has several unique advantages:^2^ (1) certain perfluorinated starting materials are inexpensive compared to their halogenated analogues; (2) C–F bonds are robust and can be carried downstream in multistep synthesis for late-stage functionalization; (3) new fluorinated motifs can be generated that would be difficult by C–F bond formation. The *defluorinative functionalization* ^3^ strategy has garnered intense research interest in recent years and has become an indispensable tool for synthetic chemists to prepare pharmaceutically relevant fluorinated molecules.^4^

Despite the tremendous progress in transition-metal-catalyzed C–F bond functionalization,^5^ palladium-catalyzed activation of allylic C–F bonds remained challenging. In an early report (2007), Fujii and co-workers described a Pd-catalyzed reductive defluorination of allylic *gem*-difluorides with PhSiH_3_ to generate monofluoroalkenes.^6^ Subsequent Pd-catalyzed transformations of allylic C–F bonds with carbon nucleophiles were also developed.^7^ However, the corresponding transformations with *heteroatom* nucleophiles have been severely limited. A breakthrough from the Paquin group documented the Pd-catalyzed allylic amination of cyclic allylic *gem*-difluorides to synthesize β-aminofluoroalkenes (Scheme 1a).^8^ Various amines could be employed as nucleophiles in this reaction. More recently, the Lautens group described the use of 3,3-difluoroindolines in the allylic C–F bond amination with morpholine (Scheme 1b).^9^ The Pd-catalyzed reaction gave the double addition product, whereas the Pd-free conditions led to the monofluoroalkene product.^10^

**Scheme 1:**
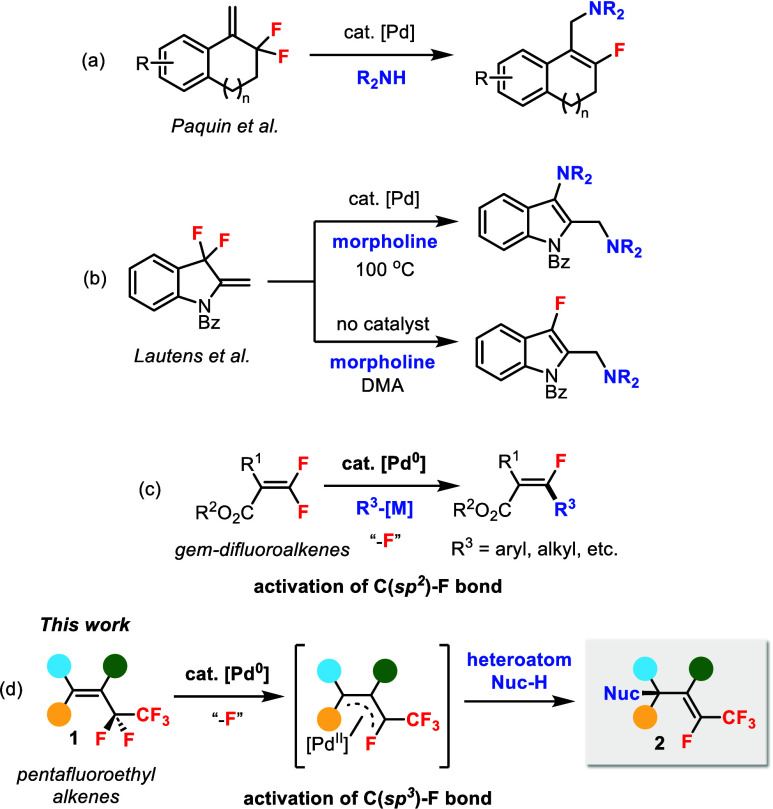
Pd-Catalyzed Allylic C–F Bond Functionalization

Our group has a continuing interest in palladium catalysis. Previously, we have developed a series of diastereoselective Pd-catalyzed C–F bond functionalizations of *gem*-difluoroalkenes for the synthesis of monofluoroalkenes (Scheme 1c).^11^ In this work, we design a new reaction motif using *pentafluoroethyl* alkenes **1** ^12^ in Pd-catalyzed defluorinative functionalization (Scheme 1d). The allylic C–F bond of **1** is presumably activated through oxidative addition by Pd(0) to generate the π-allyl palladium complex, which then reacts with a heteroatom nucleophile (Nuc-H) to form a carbon–heteroatom bond. Thus, a novel class of functionalized fluoroalkenes **2** can be obtained by eliminating one C–F bond. Compound **2** contains a unique alkene moiety connected to *both* F and CF_3_, a hybrid of monofluoroalkene and trifluoromethyl alkene,^13^ which may find advantages in therapeutic applications and patentability.^14^ Defluorofunctionalization of pentafluoroethyl alkenes has been much less investigated than the *trifluoromethyl* alkenes. In fact, S_N_2′-type reactions of α-(trifluoromethyl)­styrenes with nitrogen nucleophiles are known for forming *gem*-difluoroalkene products.^15^ The allylic C–F bond can be substituted by a C–N bond under basic conditions, even without catalysts. To the best of our knowledge, systematic studies of the defluorinative functionalization of pentafluoroethyl alkenes **1** with heteroatom nucleophiles are unexplored.

The 1,1-disubstituted pentafluoroethyl alkene **1a** was chosen as a model substrate to react with morpholine in the Pd-catalyzed *defluoroamination* reaction (Table 1). Extensive screening of reaction parameters was performed, and some important trends are shown below.^16^ Using [Pd­(dppf)­Cl_2_]·CH_2_Cl_2_ ^8^ as the catalyst and LiOH^7b^ as the base at 80 °C, the reaction afforded product **2a** in 91% yield with the *E*-product as the major diastereomer (*E*/*Z* = 81:19) (entry 1). Reaction did not occur without the Pd catalyst, and using Pd­(PPh_3_)_2_Cl_2_ gave a decent yield but poor diastereoselectivity (entries 2 and 3). Other Pd catalysts were ineffective for this reaction (entry 4). Using a larger excess of morpholine or a smaller amount of LiOH caused decreases in both yield and diastereoselectivity (entries 5 and 6). Replacing 1,4-dioxane by other solvents gave lower yields and diastereoselectivities (entry 7). Reaction at 60 °C showed similar results; however, lower temperature (40 °C) led to a poor yield, and higher temperature (100 °C) decreased the diastereoselectivity (entry 8). The use of NaOH as the base was ineffective (entry 9). It has been demonstrated that the “F–Li” interaction was important in Pd-catalyzed allylic C–F bond activation by eliminating LiF.^7b^ The impact of the ligands was also investigated using Pd­(COD)­Cl_2_ as the catalyst. The ligand dppf was superior to that of dppbz, dppp, or dppe in terms of yield and diastereoselectivity (entries 10–13). The conditions at 60 °C (entry 8) were chosen for the subsequent studies of the scope of the Pd-catalyzed defluoroamination of **1** (Scheme 2).

**Table 1:**
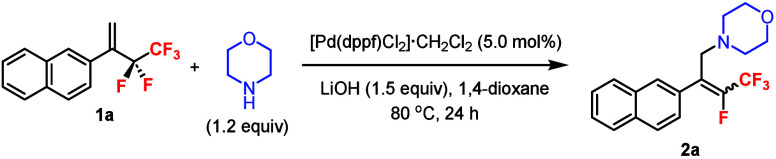
Effects of Reaction Parameters in the Defluoroamination of **1a** with Morpholine^a^

entry	deviation from above	yield (%) of **2a** ^b^	*E*/*Z* of **2a** ^b^
1	none	91	81:19
2	no Pd catalyst	0	–
3	Pd(PPh_3_)_2_Cl_2_	82	57:43
4	[Pd_2_(dba)_3_]/Pd(COD)Cl_2_/PdCl_2_	<5/<5/0	–
5	2.5 equiv morpholine	56	66:34
6	0.5 equiv LiOH	63	41:59
7	toluene/DCE/DMF as solvent	<5/22/71	–/77:23/72:28
8	40/**60**/100 °C	11/**87**/86	82:18/**82:18**/67:33
9	NaOH as base, 60 °C	<5	-
10	Pd(COD)Cl_2_ (5.0 mol %) + dppf (5.0 mol %), 60 °C	90	81:19
11	Pd(COD)Cl_2_ (5.0 mol %) + dppbz (5.0 mol %), 60 °C	77	58:42
12	Pd(COD)Cl_2_ (5.0 mol %) + dppp (5.0 mol %), 60 °C	8	75:25
13	Pd(COD)Cl_2_ (5.0 mol %) + dppe (5.0 mol %), 60 °C	<5	-

a Unless specified otherwise, reactions were carried out using **1a** (0.1 mmol) under argon.

b Determined by ^19^F NMR analysis of the crude mixture using benzotrifluoride as the internal standard.

**Scheme 2:**
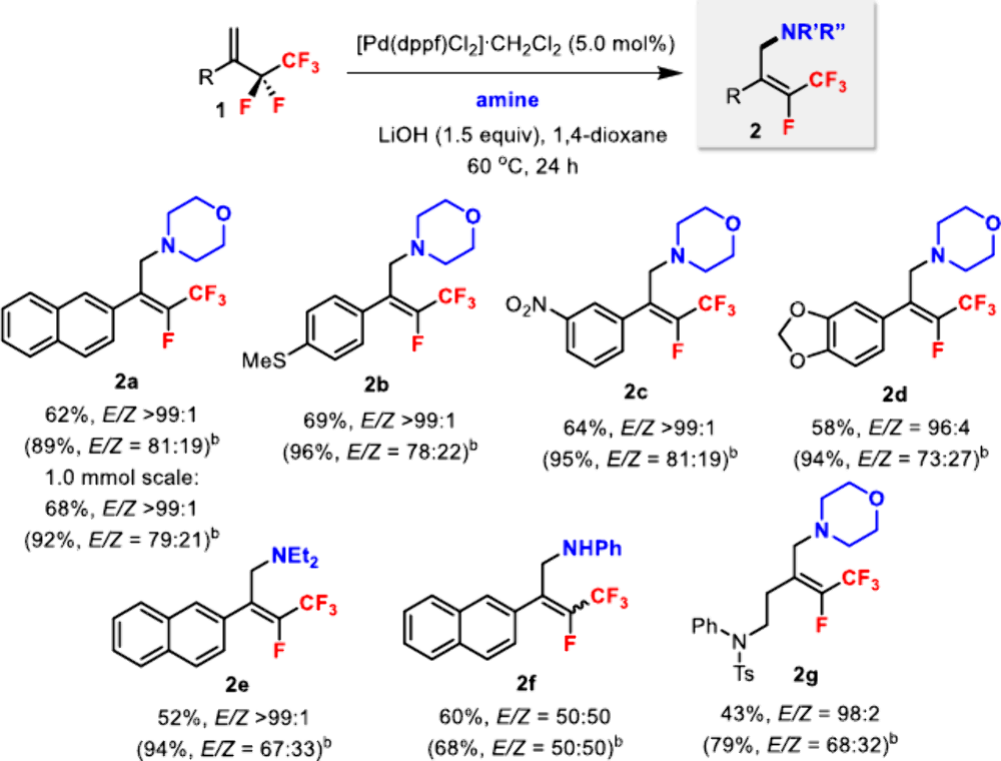
Pd-Catalyzed Defluoroamination of 1,1-Disubstituted Pentafluoroethyl Alkenes **1** with Amines^a^

a Unless specified otherwise, reactions were carried out using **1** (0.2 mmol) and amine (0.24 mmol) under argon. Isolated yields. The *E*/*Z* ratios of **2** were determined by ^19^F NMR analysis of the isolated products.
b Yield and *E*/*Z* ratio of the crude mixture by ^19^F NMR analysis.

The reaction of **1a** with morpholine could be carried out on a 1.0 mmol scale, and the major (*E*)-isomer was isolated in 68% yield with *E*/*Z* > 99:1. The (*E*)-alkene geometry of **2a** was established by ^19^F–^1^H HOESY experiments.^16^ Other aryl substituents were tolerated in the substrates, and the products were isolated in moderate yields with high *E*/*Z* ratios (**2b**–**d**). Another secondary amine (**2e**) and a primary amine (**2f**) could be employed as the nucleophile, albeit in lower diastereoselectivities than morpholine. An alkyl substituent in the substrate was also demonstrated (**2g**). It is worth mentioning that apart from **2f** the major (*E*)-products of these reactions could be *isolated* by column chromatography in 96:4 to >99:1 *E*/*Z* ratios.

Alcohols were also viable nucleophiles in the Pd-catalyzed defluoroalkoxylation of **1** (Scheme 3). Compared to amine nucleophiles, the reaction between **1a** and benzyl alcohol required higher temperature and catalyst loading to reach an acceptable yield.^16^ Benzyl alcohols with electron-withdrawing and -donating groups were tolerated (**3a**–**c**). Phenol was reactive; however, the diastereoselectivity was much poorer (**3d**). On the other hand, 2-naphthalenethiol showed comparable yield and diastereoselectivity as benzyl alcohols (**3e**). Perfluoroalkyl alcohols such as trifluoroethanol (**3f**) and hexafluoroisopropanol (**3g**) were also suitable for C–O bond formation. Simple alcohols such as methanol gave only a low yield (15%, *E*/*Z* = 80:20). Different aryl substituents of **1** were tolerated (**3h**–**i**), and an alkyl substituent (**3j**) was also compatible in the reaction. The (*E*)-alkene geometry for **3c** and **3e** was confirmed by ^19^F–^1^H HOESY experiments.^16^

**Scheme 3:**
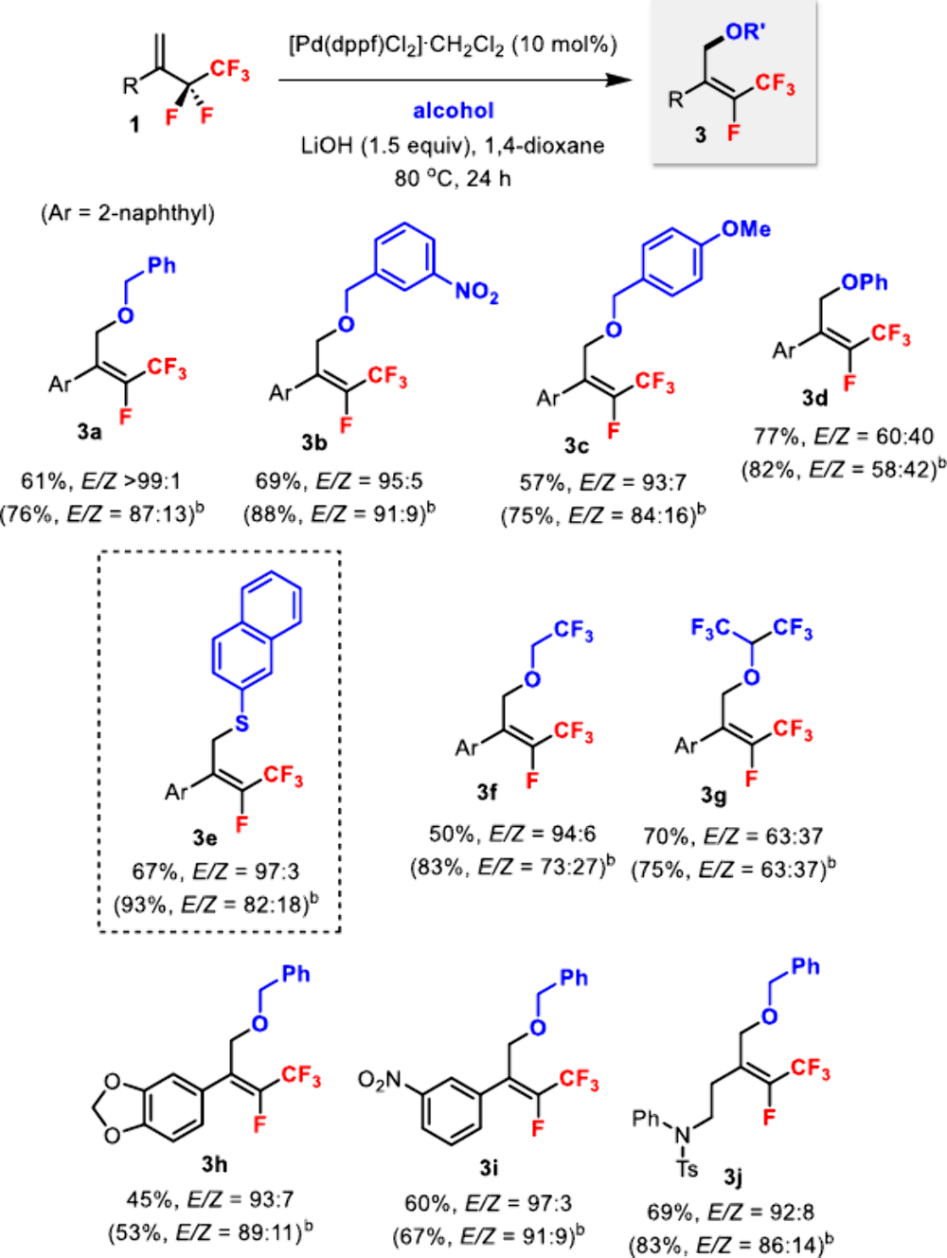
Pd-Catalyzed Defluoroalkoxylation of 1,1-Disubstituted Pentafluoroethyl Alkenes **1** with Alcohols^a^

a Unless specified otherwise, reactions were carried out using **1** (0.2 mmol) and alcohol (0.32 mmol) under argon. Isolated yields. The *E*/*Z* ratios of **3** were determined by ^19^F NMR analysis of the isolated products.
b Yield and *E*/*Z* ratio of the crude mixture by ^19^F NMR analysis.

Moreover, the reaction could be extended to the use of a *carbon* nucleophile such as ethyl acetoacetate (eq 1). Alkene **1a** reacted with the β-keto ester smoothly in 75% yield with 81:19 diastereoselectivity. Upon isolation, the product **4** was obtained in >99:1 diastereomeric ratio. The (*Z*)-alkene geometry was verified by ^19^F–^1^H HOESY experiments.^16^ Although soft carbon nucleophiles such as ethyl acetoacetate were known to react with trifluoromethyl alkenes without metal catalysts,^17^ our control experiment showed that no reaction took place between **1a** and ethyl acetoacetate in the absence of the Pd catalyst.
1

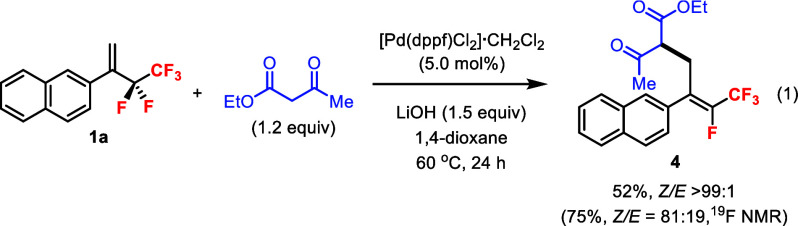




Next, the defluoroamination of 1,2-disubstituted alkenes **5** was explored under Pd catalytic conditions (Scheme 4). The reaction between morpholine and **5a** gave only 51% yield of **6a** at 80 °C.^16^ However, increasing the temperature to 100 °C significantly improved the yield, and **6a** was isolated in 87% yield even on a 1.0 mmol scale. To our delight, only one diastereomer with the (*Z*)-alkene geometry was obtained as proved by ^19^F–^1^H HOESY experiments of (*Z*)-**6a**.^16^ Other cyclic (**6b**) and acyclic (**6c**) secondary amines were also demonstrated. Primary benzylamines with different aryl substituents were compatible (**6d**–**f**). Aliphatic primary amines required higher catalyst loadings and a larger excess of amines (**6g**–**i**). Aniline only gave trace product. Substituent groups on the aromatic substrates including amide (**6j**), *tert*-butyl (**6k**), and chloro (**6l**–**m**) were tolerated. Alkyl substituents were also compatible (**6n**–**o**). Moreover, product **6p** could be obtained in excellent diastereoselectivity from the alkene containing a −C_4_F_9_ group. In contrast to the 1,1-disubstituted alkenes **1**, nucleophiles such as benzyl alcohol and ethyl acetoacetate were unreactive with **5a** under the standard conditions, and only trace amounts of products were detected. The increased steric hindrance of **5** (internal alkenes) presumably lowered their reactivity with weaker nucleophiles such as alcohols.^8^ We have also screened several chiral ligands with Pd­(COD)­Cl_2_ catalyst for the formation of **6a**, while the product could be obtained in various yields as one diastereomer. Essentially no enantioselectivities were observed.^16^

**Scheme 4:**
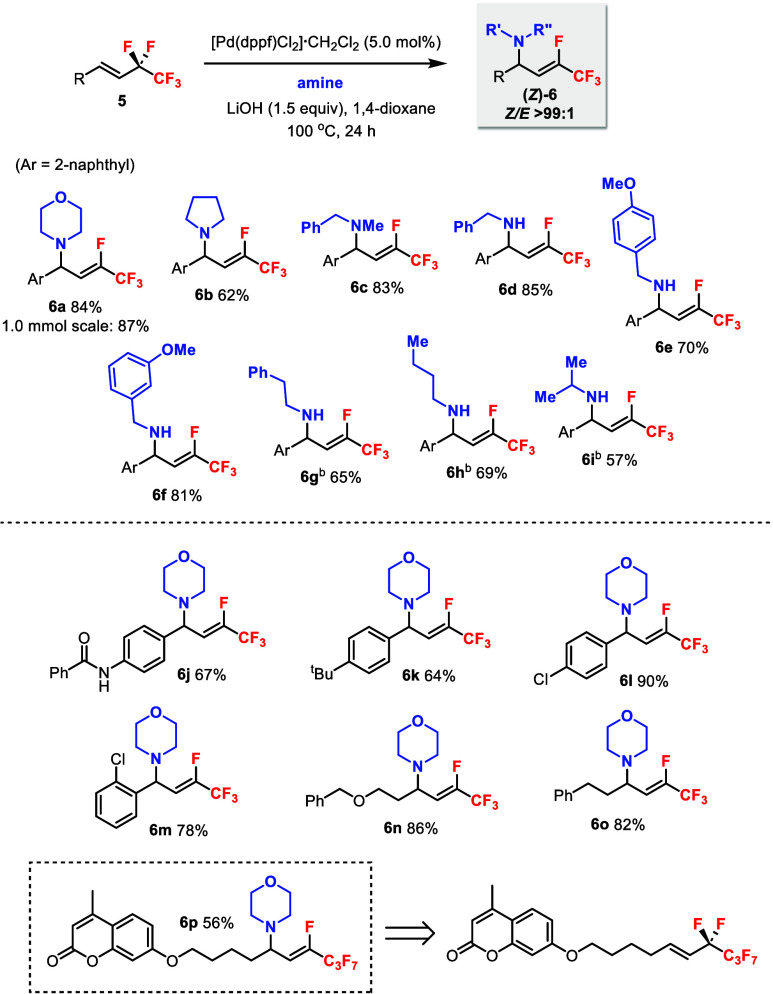
Diastereoselective Pd-Catalyzed Defluoroamination of 1,2-Disubstituted Pentafluoroethyl Alkenes **5** with Amines^a^

a Unless specified otherwise, reactions were carried out using **5** (0.2 mmol) and amine (0.24 mmol) under argon. Isolated yields. The *Z*/*E* ratios (>99:1) of **6** were determined by ^19^F NMR analysis of the isolated products.
b Used 10 mol % Pd catalyst and 0.48 mmol of amine.

Compound **2a** was a useful synthon for further derivatization (Scheme 5). Methylation of **2a** with MeOTf^15b^ generated the quaternary ammonium salt **7**, and the *E*/*Z* ratio was improved to 95:5 upon recrystallization. Subsequent S_N_2′ substitution^15c^ with heteroatom nucleophiles afforded products **8** containing a quaternary carbon center connected to F and CF_3_. Alcohols including benzyl alcohols (**8a**–**b**), allyl alcohol (**8c**), and methanol (**8d**), as well as a thiol (**8e**), were effective nucleophiles in this reaction.

**Scheme 5:**
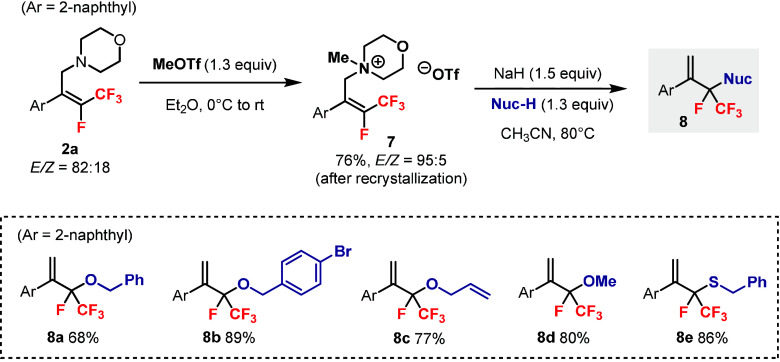
Synthesis of **8** via Substitution Reactions of the Quaternary Ammonium Salt **7**

A series of control experiments were carried out to provide more insight into the reaction details (Scheme 6). Using a diastereomerically pure (*E*)-**5o** or an *E*/*Z* mixture of **5o** (14:86) afforded the product (*Z*)-**6o** as a single diastereomer in comparable yields (Scheme 6a). Thus, the diastereocontrol of the Pd-catalyzed defluoroamination was independent of the *E*/*Z* ratios of the substrates, indicating a *stereoconvergent* process. It was found that the pentafluoroethyl alkenes **1** were intrinsically more reactive than the *trifluoromethyl* alkene under identical conditions (Scheme 6b, cf. Scheme 2). The extra two electronegative fluorine atoms of the pentafluoroethyl group can presumably enhance the alkene reactivity. A moderate yield of the defluoroamination product **2a** was observed from the 1,1-disubstituted alkene **1a** without the Pd catalyst by the S_N_2′-type reaction (Scheme 6c). However, the (*Z*)-product was the major product as confirmed by ^19^F–^1^H HOESY experiments.^16^ Therefore, the complementary (*E*)-selectivity in the formation of (*E*)-**2** from **1** (cf. Scheme 2) was controlled by the Pd catalytic pathway. In general, the Pd-catalyzed defluoroamination showed higher yields and broader scope of the substrates than those under the Pd-free conditions. No reaction took place with the 1,2-disubstituted alkene **5a** via the S_N_2′ pathway (Scheme 6d).

**Scheme 6:**
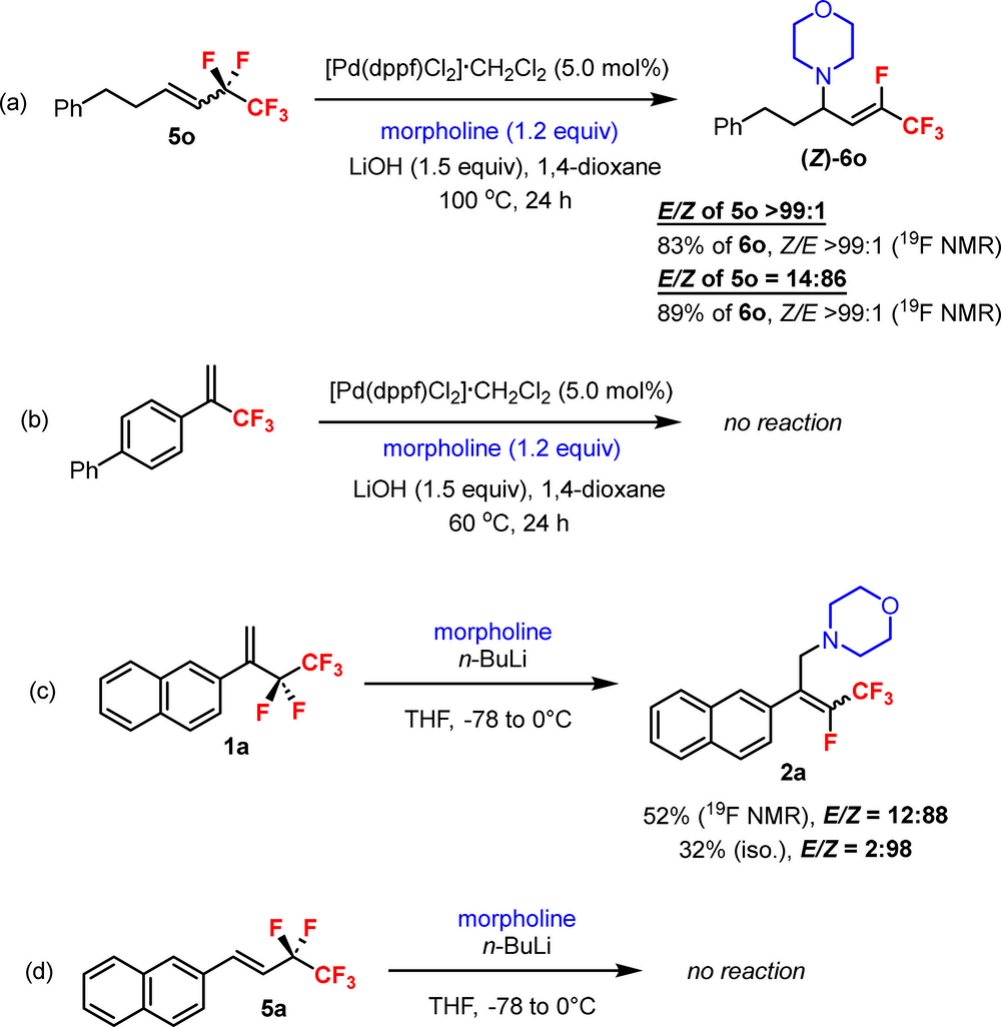
Control Experiments

In summary, we have developed a Pd-catalyzed allylic C–F bond functionalization of pentafluoroethyl alkenes using heteroatom nucleophiles. In particular, amines and alcohols were effective nucleophiles for the defluoroamination and defluoroalkoxylation of 1,1- and 1,2-disubstituted pentafluoroethyl alkene substrates. The products were a novel class of tetra- and trisubstituted alkenes containing both F and CF_3_ on the same carbon. Good to excellent diastereoselectivities could be obtained, and often the products could be isolated as a single diastereomer. Derivatization of the product was also possible to generate allylic quaternary carbon bearing F and CF_3_. Further development of transition-metal-catalyzed defluorofunctionalization of pentafluoroethyl alkenes is ongoing in our laboratory.

## Supplementary Material



## Data Availability

The data underlying this study are available in the published article and its Supporting Information.

## Notes

Experimental procedures, optimization data, characterization data, and spectral data (PDF)

## References

[1] b Kirsch, P. Modern Fluoroorganic Chemistry, 1st ed.; John Wiley & Sons, Ltd: Weinheim, Germany, 2004.

[2] Coates G., Rekhroukh F., Crimmin M. R. (2019). Breaking Carbon-Fluorine
Bonds with Main Group Nucleophiles. Synlett.

[3] Hooker L. V., Bandar J. S. (2023). Synthetic Advantages
of Defluorinative C-F Bond Functionalization. Angew. Chem., Int. Ed..

[4] Wang Q., Bian Y., Dhawan G., Zhang W., Sorochinsky A. E., Makarem A., Soloshonok V. A., Han J. (2024). FDA approved fluorine-containing drugs in 2023. Chin. Chem. Lett..

[5] Koley S., Altman R. A. (2020). Recent
Advances in Transition Metal-Catalyzed Functionalization of *gem*-Difluoroalkenes. Isr. J. Chem..

[6] Narumi T., Tomita K., Inokuchi E., Kobayashi K., Oishi S., Ohno H., Fujii N. (2007). Facile synthesis of
fluoroalkenes by palladium-catalyzed reductive defluorination of allylic *gem*-difluorides. Org. Lett..

[7] Hazari A., Gouverneur V., Brown J. M. (2009). Palladium-catalyzed
substitution of allylic fluorides. Angew. Chem.,
Int. Ed..

[8] Pigeon X., Bergeron M., Barabe F., Dube P., Frost H. N., Paquin J. F. (2010). Activation of allylic
C-F bonds: palladium-catalyzed
allylic amination of 3,3-difluoropropenes. Angew.
Chem., Int. Ed..

[9] Zeidan N., Zambri M., Unger S., Dank C., Torelli A., Mirabi B., Lautens M. (2020). Synthesis and Reactions of 3,3-Difluoro-2-exo-methylidene
Indolines. Org. Lett..

[10] Ichikawa J., Nadano R., Ito N. (2006). 5-endo Heck-type cyclization
of 2-(trifluoromethyl)­allyl ketone oximes: Synthesis of 4-difluoromethylene-substituted
1-pyrrolines. Chem. Commun..

[11] Ma Q., Wang Y., Tsui G. C. (2020). Stereoselective
Palladium-Catalyzed C-F Bond Alkynylation of Tetrasubstituted *gem*-Difluoroalkenes. Angew. Chem.,
Int. Ed..

[12] Dong T., Shen Q., Tsui G. C. (2024). Synthesis and application of well-defined [Ph_4_P]^+^[Cu­(CF_2_CF_3_)_2_]^−^ complex as a versatile pentafluoroethylating reagent. Chem. Sci..

[13] Drouin M., Hamel J.-D., Paquin J. F. (2018). Synthesis
of Monofluoroalkenes: A Leap Forward. Synthesis.

[14] Cahard, D. ; Ma, J.-A. Emerging Fluorinated Motifs: Synthesis, Properties, and Applications; Wiley-VCH: Weinheim, 2020.

[15] Fuchibe K., Takahashi M., Ichikawa J. (2012). Substitution of two fluorine atoms in a trifluoromethyl
group: regioselective synthesis of 3-fluoropyrazoles. Angew. Chem., Int. Ed..

[16] See the Supporting Information (SI) for full details.

[17] Gao Y., Qin W., Tian M.-Q., Zhao X., Hu X.-H. (2022). Defluorinative Alkylation
of Trifluoromethyl Alkenes with Soft Carbon Nucleophiles Enabled by
a Catalytic Amount of Base. Adv. Synth. Catal..

