# Supplementary material for: Palladium-Catalyzed Allylic C–F Bond Functionalization of Pentafluoroethyl Alkenes with Heteroatom Nucleophiles
Source: Org Lett. 2025 Sep 1;27(36):10141–6. doi: 10.1021/acs.orglett.5c03217 (PMC12442228; doi:10.1021/acs.orglett.5c03217)
Supplement: Supplementary file 1 [file ol5c03217_si_001.pdf]

Supporting Information

Palladium-Catalyzed Allylic C-F Bond Functionalization of Pentafluoroethyl Alkenes with Heteroatom Nucleophiles

Zhengjie Fu[a] and Gavin Chit Tsui*[a,b]

[a]Department of Chemistry, The Chinese University of Hong Kong, Shatin, New Territories, Hong Kong SAR, China
E-mail: gctsui@cuhk.edu.hk
[b]Shanghai-Hong Kong Joint Laboratory in Chemical Synthesis, The Chinese University of Hong Kong, Shatin, New Territories, Hong Kong SAR, China

Experimental Procedures and Spectral Data

Table of Contents:

I. General Experimental……………………………………………………………………………**S2**
II. Materials……………………………………………………………………………………**S2**
III. Instrumentation……………………………………………………………………………**S2**
IV. Experimental Procedures…………………………………………………………………**S4**
V. Optimization Tables………………………………………………………………………**S9**
VI. ^{19}F-^{1}H HOESY NMR Studies……………………………………………………**S12**
VII. Characterization Data of Substrates……………………………………………………**S15**
VIII. Characterization Data of Products………………………………………………………**S17**
IX. Reference…………………………………………………………………………………**S29**
X. Spectra of Substrates ………………………………………………………………………**S30**
XI. Spectra of Products …………………………………………………………………………**S38**

I. General Experimental.

Unless otherwise noted, reactions were carried out in a 10 mL glass tube with magnetic stirring. Reactions that require heating were carried out in the oil bath. Analytical thin layer chromatography (TLC) was performed with Merck silica gel 60 F254 aluminum plates. Visualization was done under a UV lamp (254 nm) and by immersion in potassium permanganate ($KMnO_4$), followed by heating using a heat gun. Organic solutions were concentrated by rotary evaporation at 23-40 °C. Purification of reaction products were generally done by flash column chromatography with Silicycle 60-230 mesh silica gel. Structural assignments were made with additional information from ^{19}F-^{1}H HOESY experiments.

II. Materials.

Anhydrous LiOH was purchased from Aladdin Scientific. Phosphine ligands were purchased from J&K Scientific, Leyan and Bidepharm. Palladium catalysts were purchased from J&K Scientific, Acros and Bidepharm. Amines and alcohols were purchased from Dieckmann, Energy Chemical and J&K Scientific. Pentafluoroethyl alkenes for substrates synthesis were prepared according to literature procedure. Other chemicals for substrates preparation were purchased from Acros, J&K Scientific, Aldrich and Dieckmann.

III. Instrumentation.

Proton nuclear magnetic resonance spectra (^{1}H NMR), carbon nuclear magnetic resonance spectra (^{13}C NMR) and fluorine nuclear magnetic resonance spectra (^{19}F NMR) were recorded at 23 °C on Bruker 400 MHz or 500 MHz spectrometer in $CDCl_3$. Chemical shifts of ^{1}H NMR spectra were reported as parts per million in δ scale using residual solvent signal ($CDCl_3$: 7.26 ppm) or tetramethylsilane (0.00 ppm) as internal standard. Chemical shifts of ^{13}C NMR spectra were reported using residual solvent signal of $CDCl_3$ (77.16 ppm) on the δ scale. Chemical shifts of ^{19}F NMR were reported as parts per million in δ scale using benzotrifluoride (-63.72 ppm) as internal standard. Data are represented as follows: chemical shift (δ ppm), multiplicity (s = singlet, d = doublet, t = triplet, q = quartet, m = multiplet), coupling constant (J, Hz) and integration. High resolution mass spectra (HRMS) were obtained on a Finnigan MAT 95XL GC Mass Spectrometer or a Thermo Scientific Q Exactive Focus Mass Spectrometer or a Bruker SolariX 9.4T FTMS with Q Exactive Focus Orbitrap.

Substrates 1 and 5

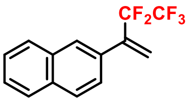

1a

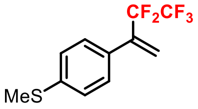

1b

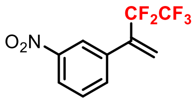

1c

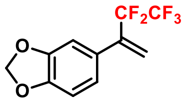

1d

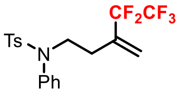

1g

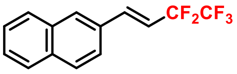

5a

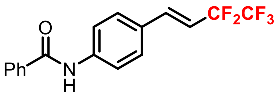

5j

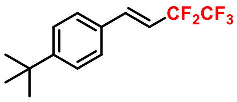

5k

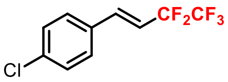

5l

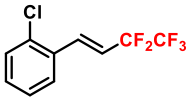

5m

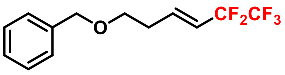

5n

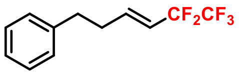

5o

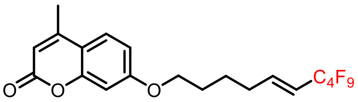

5p

Note: substrates **1** and **5** are prepared according to literature procedures[1-3], **1a, 5a, 5j-5m, 5o** and **5p** are known compounds.

IV. Experimental Procedures.

General procedure (I) for the synthesis of pentafluoroethyl ketones S1:

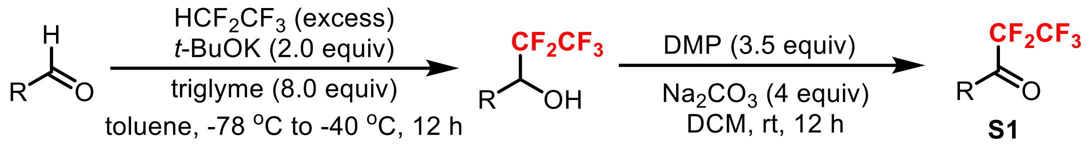

Pentafluoroethyl ketones **S1** was synthesized according to literature procedure with minor changes.[1] In a glove box, to an oven-dried 100 mL round-bottom flask equipped with *t*-BuOK (2.2 g, 20.0 mmol, 2.0 equiv) and a stir bar. The flask was sealed with a septum, brought out of the glove box, and put under an argon atmosphere. The solution of triglyme (14.4 mL, 80 mmol, 8.0 equiv) in toluene (40 mL) was added under -78 °C, then pentafluoroethane (HCF_2CF_3) was bubbled into the mixture by using a needle connected to the HCF_2CF_3 cylinder at room temperature for 30 min. After removing the HCF_2CF_3 inlet, the mixture was warmed to -40°C. Then the solution of aldehyde (10.0 mmol) in toluene (10.0 mL) was added through syringe. And the reaction mixture was stirred at the same temperature for 12 h, then it was quenched with sat. NH_4Cl aq. (50.0 mL). The aqueous layer was extracted with Et_2O (50 mL × 3), and the combined organic layers were washed with brine, and then dried over anhydrous Na_2SO_4. After filtration and evaporation under vacuum, the residue was directly used for the next step without purification.

To a solution of the α-CF_2CF_3 alcohol (10 mmol, 1 equiv) in DCM (60 mL) in a round bottom flask, Dess-Martin periodinane (14.8 g, 35 mmol, 3.5 equiv) and Na_2CO_3 (4.2 g, 40 mmol, 4 equiv) was added. The solution was stirred at room temperature for 12 h. Then water was added, and the obtained suspension was stirred for an additional 30 min, the mixture was extracted with DCM (60 mL × 3). The organic phase was washed with brine and then dried over anhydrous Na_2SO_4. The solvent was removed under vacuo by rotary evaporation to give the α-CF_2CF_3 ketones **S1**.

General procedure (II) for the synthesis of pentafluoroalkenes 1:

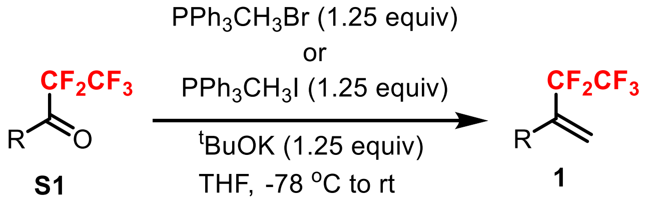

To an oven-dried round bottom flask was added methyltriphenylphosphonium iodide (2.0 g, 5 mmol, 1.25 equiv) or methyltriphenylphosphonium bromide (1.8 g, 5 mmol, 1.25 equiv) and ^{t}BuOK (562 mg, 5.0 mmol, 1.25 equiv) in THF (10 mL) under argon. The mixture was stirred at room temperature for 30 mins and cooled to -78 °C, then α-CF_2CF_3 ketones **S1** (4 mmol, 1 equiv) in THF (5 mL) was added under -78 °C. The reaction mixture was allowed to warm up to room temperature with vigorously stirring over 12 h. Later the reaction was quenched with water (50 mL) and extracted with DCM (15 mL × 3). The organic layer was dried over anhydrous Na_2SO_4 and concentrated in vacuo. The residue was purified by flash column chromatography to afford the corresponding alkenes **1**.

General Procedure (III) for the synthesis of alkenyl iodides S2

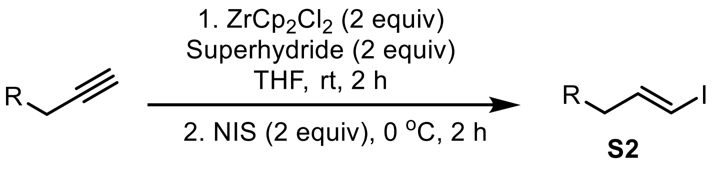

Alkenyl iodides **S2** were synthesized according to the literature procedure.[1] A round bottom flask charged with $ZrCp_2Cl_2$ (2.9 g, 10 mmol, 2 equiv) was added THF (10 mL). The superhydried solution (1.0 M in THF, 10 mmol, 2 equiv) was added. The solution was stirred in the dark for 2 h before a solution of alkyne (5 mmol, 1 equiv) in THF (10 mL) was added. The reaction was stirred for 15 min and cooled to 0°C, then the NIS (2.3 g, 10 mmol, 2 equiv) in THF(10 mL) solution was added.

The reaction was stirred for 2 h and then quenched by NH_4Cl solution. The reaction mixture was extracted with Et_2O (3 × 30 ml). The combined organic layers were washed with brine, dried over anhydrous Na_2SO_4 and concentrated in vacuo. The residue was purified by flash column chromatography on silica gel to afford the corresponding alkenyl iodides **S2**.

General procedure (IV) for the synthesis of pentafluoroethyl alkenes 1:

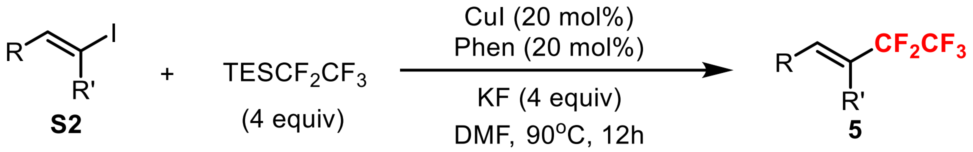

Pentafluoroethyl alkenes **5** was synthesized according to the literature procedure.[2] To an oven-dried round bottom flask equipped with a stir bar, the alkenyl iodide (3 mmol, 1 equiv), CuI (114.0 mg, 0.6 mmol, 0.2 equiv), 1,10-Phenanthroline (108.0 mg, 0.6 mmol, 0.2 equiv) and KF (696.0 mg, 12.0 mmol, 4 equiv) were added in the glovebox. The round bottom flask was sealed with a rubber septum and removed from the glovebox. $TESCF_2CF_3$ (2.8 g, 12 mmol, 4 equiv) and DMF (30 mL) were then added to the flask through syringe under argon. The resulting mixture was stirred at 90°C for 12 h. After cooling to room temperature, the reaction mixture was extracted with Et_2O (3 × 30 ml). The combined organic layers were washed with H_2O (2 × 30 ml), then brine (2 × 30 ml), dried over anhydrous Na_2SO_4 and concentrated in vacuo. The residue was purified by flash column chromatography on silica gel to afford the corresponding pentafluoroethyl alkenes **5**.

General procedure (V) for Palladium(II)-Catalyzed Defluoroamination of Pentafluoroethyl Alkenes 1 with amines

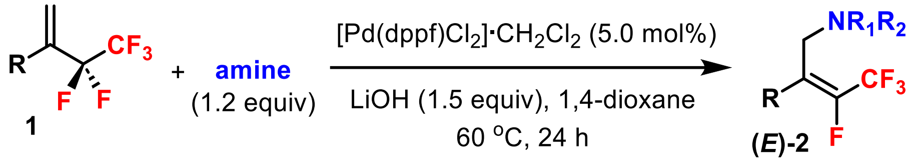

To an oven-dried 10 mL glass tube equipped with a stir bar was added LiOH (7.2 mg, 0.3 mmol) and $[Pd(dppf)Cl_2]\cdot CH_2Cl_2$ (8.04 mg, 0.01 mmol). Then the tube was sealed with a septum, evacuated and refilled with argon three times, then a solution of **1** (0.2 mmol) and amine (0.24 mmol) in 1,4-dioxane (1 mL) was added through syringe under argon. The resulting mixture was heated at 60 °C with an oil bath for 24 h with rigorous stirring. After cooling to room temperature, the reaction mixture was extracted with CH_2Cl_2 (3 × 10 mL). The combined organic layers were washed with H_2O (2 × 10 mL), then brine (2 × 10 mL), dried over anhydrous Na_2SO_4 and concentrated in vacuo. The residue was purified by flash column chromatography on silica gel to afford products **2**.

General procedure (VI) for Palladium(II)-Catalyzed Defluoroamination of Pentafluoroethyl Alkenes 1 with Alcohols

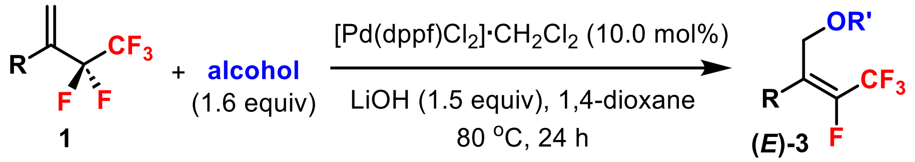

To an oven-dried 10 mL glass tube equipped with a stir bar was added LiOH (7.2 mg, 0.3 mmol) and $[Pd(dppf)Cl_2]\cdot CH_2Cl_2$ (16.1 mg, 0.02 mmol). Then the tube was sealed with a septum, evacuated and refilled with argon three times, then a solution of **1** (0.2 mmol) and alcohol (0.32 mmol) in 1,4-dioxane (1 mL) was added through syringe under argon. The resulting mixture was heated at 80 °C with an oil bath for 24 h with rigorous stirring. After cooling to room temperature, the reaction mixture was extracted with CH_2Cl_2 (3 × 10 mL). The combined organic layers were washed with H_2O (2 × 10 mL), then brine (2 × 10 mL), dried over anhydrous Na_2SO_4 and concentrated in vacuo. The residue was purified by flash column chromatography on silica gel to afford products **3**.

General procedure (VII) for Palladium(II)-Catalyzed Defluoroamination of Pentafluoroethyl Alkenes 1 with acetoacetate

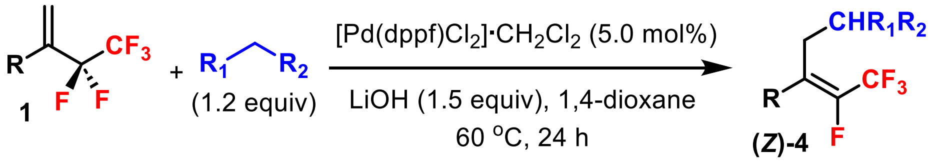

To an oven-dried 10 mL glass tube equipped with a stir bar was added LiOH (7.2 mg, 0.3 mmol) and [Pd(dppf)Cl_2]·CH_2Cl_2 (8.04 mg, 0.01 mmol). Then the tube was sealed with a septum, evacuated and refilled with argon three times, then a solution of **1** (0.2 mmol) and acetoacetate (0.24 mmol) in 1,4-dioxane (1 mL) was added through syringe under argon. The resulting mixture was heated at 60 °C with an oil bath for 24 h with rigorous stirring. After cooling to room temperature, the reaction mixture was extracted with CH_2Cl_2 (3 × 10 mL). The combined organic layers were washed with H_2O (2 × 10 mL), then brine (2 × 10 mL), dried over anhydrous Na_2SO_4 and concentrated in vacuo. The residue was purified by flash column chromatography on silica gel to afford products **4**.

General procedure (VIII) for Palladium(II)-Catalyzed Defluoroamination of Pentafluoroethyl Alkenes 1 with Amines

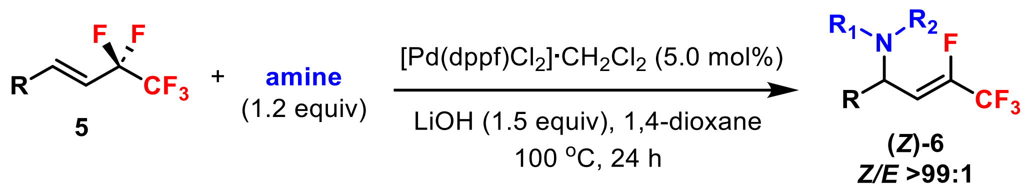

To an oven-dried 10 mL glass tube equipped with a stir bar was added LiOH (7.2 mg, 0.3 mmol) and [Pd(dppf)Cl_2]·CH_2Cl_2 (8.04 mg, 0.01 mmol). Then the tube was sealed with a septum, evacuated and refilled with argon three times, then a solution of **5** (0.2 mmol) and amine (0.24 mmol) in 1,4-dioxane (1 mL) was added through syringe under argon. The resulting mixture was heated at 100 °C with an oil bath for 24 h with rigorous stirring. After cooling to room temperature, the reaction mixture was extracted with CH_2Cl_2 (3 × 10 mL). The combined organic layers were washed with H_2O (2 × 10 mL), then brine (2 × 10 mL), dried over anhydrous Na_2SO_4 and concentrated in vacuo. The residue was purified by flash column chromatography on silica gel to afford products **6**.

General procedure (IX) for Palladium(II)-Catalyzed Defluoroarylation of Pentafluoroethyl Alkenes 1 with Amines

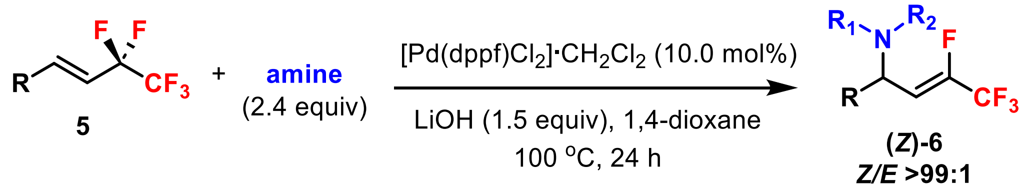

To an oven-dried 10 mL glass tube equipped with a stir bar was added LiOH (7.2 mg, 0.3 mmol) and [Pd(dppf)Cl_2]·CH_2Cl_2 (8.04 mg, 0.02 mmol). Then the tube was sealed with a septum, evacuated and refilled with argon three times, then a solution of **5** (0.2 mmol) and amine (0. 48 mmol) in 1,4-dioxane (1 mL) was added through syringe under argon. The resulting mixture was heated at 100 °C with an oil bath for 24 h with rigorous stirring. After cooling to room temperature, the reaction mixture was extracted with CH_2Cl_2 (3 × 10 mL). The combined organic layers were washed with H_2O (2 × 10 mL), then brine (2 × 10 mL), dried over anhydrous Na_2SO_4 and concentrated in vacuo. The residue was purified by flash column chromatography on silica gel to afford products **6**.

General procedure (X) for S_N2' of 7

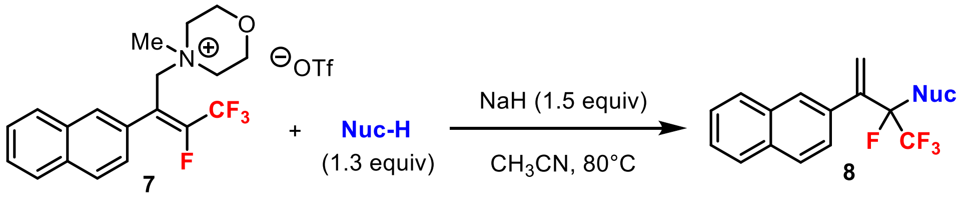

To an oven-dried 10 mL glass tube equipped with a stir bar was added NaH (7.2 mg, 0.3 mmol and nucleophile (1.3 eq.). Then the tube was sealed with a septum, evacuated and refilled with argon three times, then a solution of **7** (100.6 mg, 0.2 mmol) in CH_3CN (1 mL) was added through syringe under argon and the mixture was heated at 80 °C with stirring in an oil bath for 12h. After cooling to room temperature, the reaction mixture was filtered and concentrated in vacuo. The residue was purified by flash column chromatography on silica gel to afford products **8.**

Synthesis of 7: (E)-4-methyl-4-(3,4,4,4-tetrafluoro-2-(naphthalen-2-yl)but-2-en-1-yl)morpholin-4-iumtrifluoromethanesulfonate salt

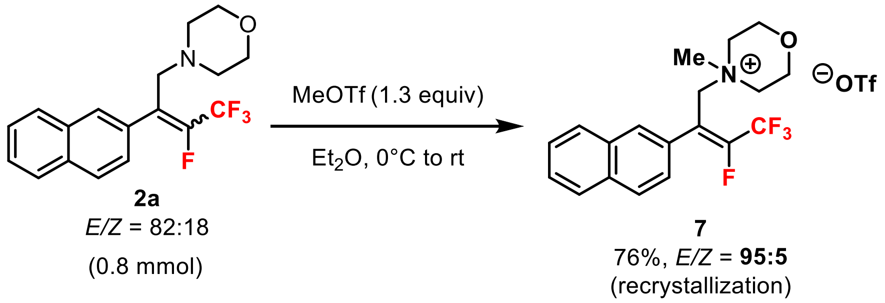

7 is prepared followed by literature.[3] using **2a** (0.27 g, 0.8 mmol) and MeOTf (0.17 g, 1.0 mmol). The product was purified by flash column chromatography on silica gel (ethyl acetate: hexane = 1: 20) and obtained as a pale yellow solid (67.8 mg, 76% yield, *E/Z* = 95:5, *E* isomer), R_f = 0.48 (ethyl acetate: hexane = 1: 5). ^{1}**H NMR** (500 MHz, Acetone-d_6): δ (ppm) δ 8.30 (s, 1H), 8.06 (d, J = 8.6 Hz, 1H), 8.02 – 7.95 (m, 2H), 7.76 (dq, J = 8.6, 1.6 Hz, 1H), 7.68 – 7.58 (m, 2H), 4.90 (s, 2H), 3.86 (s, 4H), 3.66 – 3.35 (m, 7H). ^{13}**C NMR** (101 MHz, $CDCl_3$): δ (ppm) 147.7 (dq, J = 262.9, 38.3 Hz), 134.7, 134.0, 130.1, 130.0 (dd, J = 8.3, 3.9 Hz), 129.7 – 129.3 (m), 128.7 (d, J = 5.4 Hz), 128.6, 127.9, 126.5, 123.6, 121.7 – 118.2 (m), 119.7 (dq, J = 15.6, 2.4 Hz), 64.2, 55.9 (dq, J = 5.9, 3.1 Hz), 53.7, 53.5. ^{19}**F NMR** (471 MHz, Acetone-d_6): δ (ppm) -65.23 (d, J = 8.3 Hz, 3F), -79.22 (s, 3F), -118.70 (s, 1F). **HRMS** m/z (ESI): calcd. for $C_{19}H_{20}F_4NO$ $[M]^+$: 354.1476; found: 354.1471; calcd. for CF_3O_3S $[M]^-$: 148.9526; found: 148.9524.

Synthesis of Z-2a

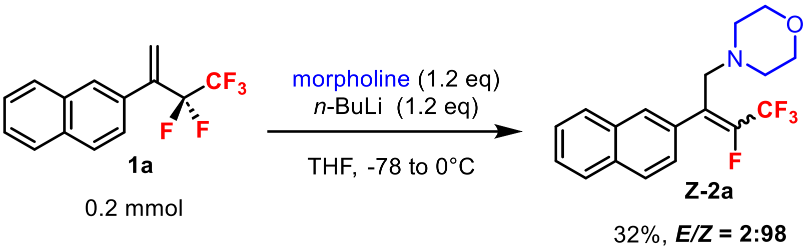

Z-2a is prepared followed by literature.[3] In a flame-dried flask equipped with a stir bar and filled with argon were added morpholine (20.9 mg, 0.24 mmol) and anhydrous THF (0.2 mL). The flask was cooled to -78 °C for 10 min. Then a 1.6 M solution of n-BuLi (0.15 mL, 0.24 mmol, 1.2 equiv) in hexanes was added dropwise to the flask. The solution was stirred at −78 °C for 1 h and gradually became cloudy and white. After this time, **1a** (54.4 mg, 0.2 mmol) was added to the flask dropwise over 5 min. The solution was stirred at −78 °C for 1 h and after this time was warmed to 0 °C. The solution was stirred at 0 °C for 1 h and then was poured into a separatory funnel containing saturated aqueous NH_4Cl. The aqueous layer was extracted with EtOAc 3 times. The combined organic layers were dried (Na_2SO_4) and the solvent was removed under reduced pressure. The resultant crude product material was purified by flash column chromatography on silica gel (ethyl acetate: hexane = 1: 40) and obtained as a yellow oil (42.1 mg, 32% yield, *E/Z* = 2:98, *Z* isomer), R_f = 0.40 (ethyl acetate: hexane = 1: 5). ^{1}**H NMR** (400 MHz, $CDCl_3$): δ (ppm) 7.85 (dd, J = 7.1, 4.2 Hz, 3H), 7.72 (s, 1H), 7.53 (dt, J = 6.3, 3.4 Hz, 2H), 7.36 (dd, J = 8.5, 1.8 Hz, 1H), 3.65 (t, J = 4.7 Hz, 4H), 3.50 (s, 2H), 2.51 (t, J = 4.7 Hz, 4H). ^{13}**C NMR** (101 MHz, $CDCl_3$): δ (ppm) 144.7 (dq, J = 257.8, 37.1 Hz), 133.1, 132.9, 131.3 (d, J = 4.8 Hz), 128.3, 128.0, 127.9, 127.82, 127.77, 126.8, 126.6, 126.1, 118.9 (qd, J = 273.8, 42.9 Hz), 66.9, 58.0 (d, J = 3.7 Hz), 53.4. ^{19}**F NMR** (471 MHz, $CDCl_3$): δ (ppm) -65.91 (d, J = 9.1 Hz, 3F), -128.76(s, 1F). **HRMS** m/z (ESI): calcd. for $C_{18}H_{18}F_4NO$ $[M+H]^+$: 340.1319; found: 340.1316.

1 mmol scale synthesis of 2a

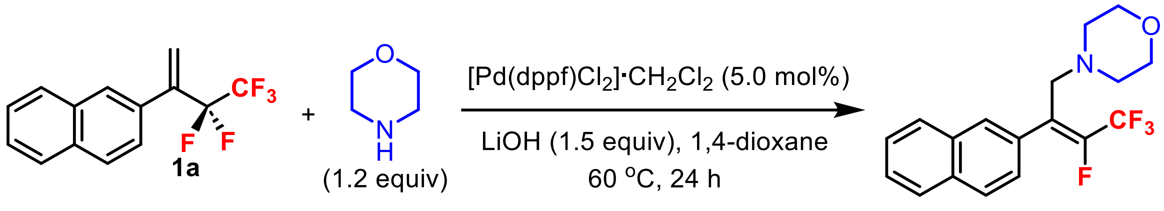

(*E*)-2a

To an oven-dried 25 mL round bottom flask equipped with a stir bar was added LiOH (36 mg, 1.5 mmol) and [Pd(dppf)Cl_2]·CH_2Cl_2 (40.2 mg, 0.05 mmol). Then the tube was sealed with a septum, evacuated and refilled with argon three times, then a solution of **1a** (0.27 g, 1 mmol) and morpholine (0.10 g, 1.2 mmol) in 1,4-dioxane (5 mL) was added through syringe under argon. The resulting mixture was heated at 60 °C with an oil bath for 24 h with rigorous stirring. After cooling to room temperature, the reaction mixture was filtered and concentrated in vacuo. The residue was purified by flash column chromatography on silica gel to afford products **2a** as a yellow oil (230.4 mg, 68% yield), R_f = 0.43 (ethyl acetate: hexane = 1: 5).

V. Optimization Tables

Table S1. Optimization studies for Pd-catalyzed defluoroamination of pentafluoroethyl alkene 1a with morpholine.[a]

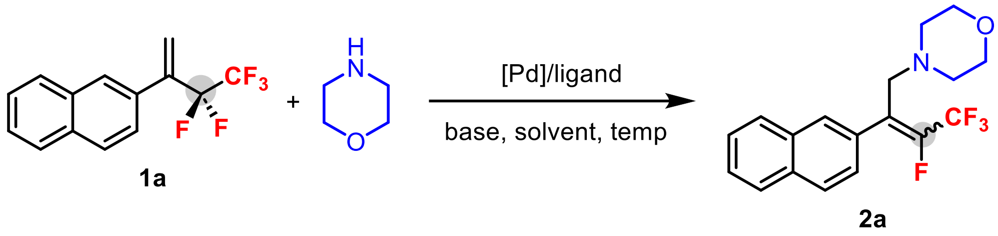

entry	morpholine equiv	[Pd] (mol%)	ligand (mol%)	base (equiv)	solvent	temp (°C)	yield (%)[b]	*E/Z* ratio[b]
1	2.0	$[Pd(dppf)Cl_2]\cdot CH_2Cl_2$ (5.0)	none	none	1,4-Dioxane	80	28	78:22
2	2.0	$[Pd(dppf)Cl_2]\cdot CH_2Cl_2$ (5.0)	none	LiOH (1.5)	1,4-Dioxane	80	78	75:25
3	**2.5**	$[Pd(dppf)Cl_2]\cdot CH_2Cl_2$ (5.0)	none	LiOH (1.5)	1,4-Dioxane	80	56	66:34
4	2.0	$[Pd(dppf)Cl_2]\cdot CH_2Cl_2$ (5.0)	none	LiOH (1.5)	1,4-Dioxane	**60**	72	82:18
5	2.0	**none**	none	LiOH (1.5)	1,4-Dioxane	80	0	n.d.
6	3.0	$[Pd(dppf)Cl_2]\cdot CH_2Cl_2$ (5.0)	none	LiOH (1.5)	1,4-Dioxane	80	36	75:25
7	1.2	$[Pd(dppf)Cl_2]\cdot CH_2Cl_2$ (5.0)	none	LiOH (1.5)	1,4-Dioxane	80	91	81:19
8	1.0	$[Pd(dppf)Cl_2]\cdot CH_2Cl_2$ (5.0)	none	LiOH (1.5)	1,4-Dioxane	80	82	78:22
9	1.2	$[Pd(dppf)Cl_2]\cdot CH_2Cl_2$ (5.0)	none	LiOH (3.0)	1,4-Dioxane	80	90	82:18
10	1.2	$[Pd(dppf)Cl_2]\cdot CH_2Cl_2$ (5.0)	none	LiOH (1.0)	1,4-Dioxane	80	70	56:44
11	1.2	$[Pd(dppf)Cl_2]\cdot CH_2Cl_2$ (5.0)	none	LiOH (0.5)	1,4-Dioxane	80	63	41:59
12	1.2	$[Pd(dppf)Cl_2]\cdot CH_2Cl_2$ (5.0)	none	LiOH (1.5)	DMF	80	71	72:28
13	1.2	$[Pd(dppf)Cl_2]\cdot CH_2Cl_2$ (5.0)	none	LiOH (1.5)	toluene	80	<5	n.d.
14	1.2	$[Pd(dppf)Cl_2]\cdot CH_2Cl_2$ (5.0)	none	LiOH (1.5)	DCE	80	22	77:23
15	1.2	none	none	LiOH (1.5)	1,4-Dioxane	80	0	n.d.
16	1.2	$Pd(OAc)_2$ (5.0)	none	LiOH (1.5)	1,4-Dioxane	80	0	n.d.
17	1.2	$PdCl_2$ (5.0)	none	LiOH (1.5)	1,4-Dioxane	80	0	n.d.
18	1.2	$Pd_2(dba)_3]$ (5.0)	none	LiOH (1.5)	1,4-Dioxane	80	<5	n.d.
19	1.2	$Pd(TFA)_2$ (5.0)	none	LiOH (1.5)	1,4-Dioxane	80	<5	n.d.
20	1.2	$Pd(PPh_3)_2Cl_2$ (5.0)	none	LiOH (1.5)	1,4-Dioxane	80	82	57:43
21	1.2	$Pd(COD)Cl_2$ (5.0)	none	LiOH (1.5)	1,4-Dioxane	80	<5	n.d.
22	1.2	PEPPSI™-IPr (5.0)	none	LiOH (1.5)	1,4-Dioxane	80	10	80:20
**23**	**1.2**	**$[Pd(dppf)Cl_2]\cdot CH_2Cl_2$ (5.0)**	**none**	**LiOH (1.5)**	**1,4-Dioxane**	**60**	**87**	**82:18**
24	1.2	$[Pd(dppf)Cl_2]\cdot CH_2Cl_2$ (5.0)	none	none	1,4-Dioxane	60	0	n.d.
25	1.2	$[Pd(dppf)Cl_2]\cdot CH_2Cl_2$ (5.0)	none	LiOH (1.5)	1,4-Dioxane	50	30	83:17
26	1.2	$[Pd(dppf)Cl_2]\cdot CH_2Cl_2$ (5.0)	none	LiOH (1.5)	1,4-Dioxane	40	11	82:18
27	1.2	$[Pd(dppf)Cl_2]\cdot CH_2Cl_2$ (5.0)	none	LiOH (1.5)	1,4-Dioxane	100	86	67:33
28	1.2	$[Pd(dppf)Cl_2]\cdot CH_2Cl_2$ (5.0)	none	NaOH (1.5)	1,4-Dioxane	60	<5	n.d.
29	1.2	$Pd(COD)Cl_2$ (5.0)	dppf (5.0)	LiOH (1.5)	1,4-Dioxane	60	90	81:19
30	1.2	$Pd(COD)Cl_2$ (5.0)	dppf (10.0)	LiOH (1.5)	1,4-Dioxane	60	87	86:14
31	1.2	$Pd(COD)Cl_2$ (5.0)	1,1'-bis(dichlorophosphino) ferrocene (5.0)	LiOH (1.5)	1,4-Dioxane	60	0	n.d.
32	1.2	$Pd(COD)Cl_2$ (5.0)	dppm (5.0)	LiOH (1.5)	1,4-Dioxane	60	<5	n.d.
33	1.2	$Pd(COD)Cl_2$ (5.0)	dppe (5.0)	LiOH (1.5)	1,4-Dioxane	60	<5	n.d.
34	1.2	$Pd(COD)Cl_2$ (5.0)	dppp (5.0)	LiOH (1.5)	1,4-Dioxane	60	8	75:25

35	1.2	Pd(COD)Cl_2 (5.0)	dppbz (5.0)	LiOH (1.5)	1,4-Dioxane	60	77	58:42

[a]Unless specified otherwise, reactions were carried out using 0.1 mmol **1a** and 0.5 mL solvent under argon for 24 h. [b]Yields and *E/Z* ratios were determined by ^{19}F NMR analysis of the crude mixture using benzotrifluoride as internal standard.

Table S2. Optimization studies for Pd-catalyzed defluoroamination of pentafluoroethyl alkene 1a with alcohol.[a]

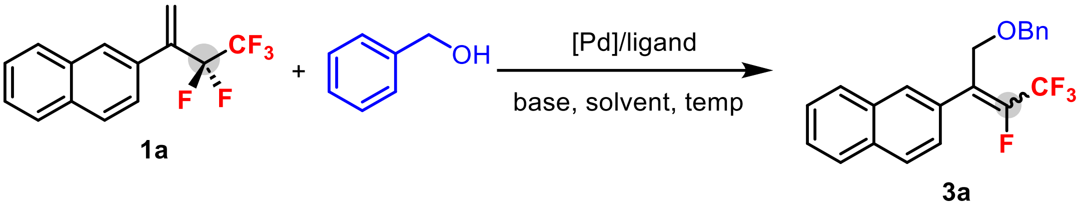

entry	BnOH equiv	[Pd] (mol%)	base (equiv)	solvent	temp (°C)	yield (%)[b]	*E/Z* ratio[b]
1	1.2	[Pd(dppf)Cl_2]·CH_2Cl_2 (5.0)	LiOH (1.5)	1,4-Dioxane	60	50	78: 12
2	1.2	[Pd(dppf)Cl_2]·CH_2Cl_2 (5.0)	LiOH (1.5)	1,4-Dioxane	80	67	79:21
3	1.2	[Pd(dppf)Cl_2]·CH_2Cl_2 (5.0)	LiOH (1.5)	1,4-Dioxane	100	36	75:25
4	1.2	[Pd(dppf)Cl_2]·CH_2Cl_2 (5.0)	LiOH (3.0)	1,4-Dioxane	80	N.D.	/
5	2.5	[Pd(dppf)Cl_2]·CH_2Cl_2 (5.0)	LiOH (1.5)	1,4-Dioxane	80	58	78:22
6	1.6	[Pd(dppf)Cl_2]·CH_2Cl_2 (5.0)	LiOH (1.5)	1,4-Dioxane	80	72	87:13
**7**	**1.6**	**[Pd(dppf)Cl_2]·CH_2Cl_2 (10.0)**	**LiOH (1.5)**	**1,4-Dioxane**	**80**	**78**	**88:12**
8	1.6	/	LiOH (1.5)	1,4-Dioxane	80	0	/

[a]Unless specified otherwise, reactions were carried out using 0.1 mmol **1a** and 0.5 mL solvent under argon for 24 h. [b]Yields and *E/Z* ratios were determined by ^{19}F NMR analysis of the crude mixture using benzotrifluoride as internal standard.

Table S3. Optimization studies for Pd-catalyzed defluoroamination of pentafluoroethyl alkene 5a with morpholine.[a]

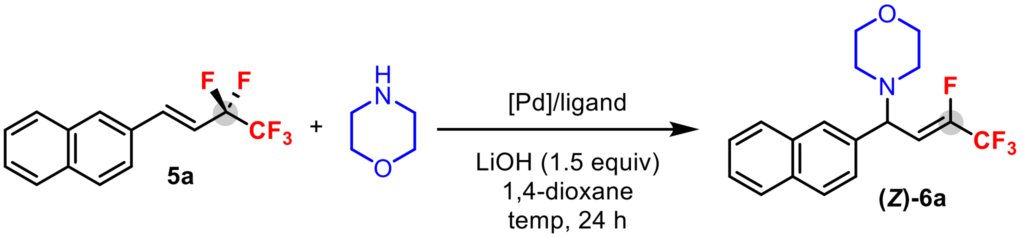

entry	morpholine equiv	[Pd] (mol%)	ligand (mol%)	temp (°C)	yield (%)[b]	*Z/E* ratio[b]	e.r.[d]
1	1.2	[Pd(dppf)Cl₂]·CH₂Cl₂ (5.0)	none	80	51	>99:1	n.d.
2	2.0	[Pd(dppf)Cl₂]·CH₂Cl₂ (5.0)	none	80	82	>99:1	n.d.
**3**	**1.2**	**[Pd(dppf)Cl₂]·CH₂Cl₂ (5.0)**	**none**	**100**	**94**	**>99:1**	**n.d.**
4	1.2	Pd(COD)Cl₂ (5.0)	dppf (5.0)	100	24	>99:1	n.d.
5	1.2	Pd(PPh₃)₂Cl₂ (5.0)	dppf (5.0)	100	21	>99:1	n.d.
6	1.2	Pd(OAc)₂ (5.0)	dppf (5.0)	100	6	>99:1	n.d.
7	1.2	Pd(dba)₂ (5.0)	dppf (5.0)	100	13	>99:1	n.d.
8	1.2	Pd(CN)₂Cl₂ (5.0)	dppf (5.0)	100	27	>99:1	n.d.
9	1.2	PdCl₂ (5.0)	dppf (5.0)	100	9	>99:1	n.d.
10	2.5	Pd(COD)Cl₂ (5.0)	dppf (5.0)	100	39	>99:1	n.d.
11	2.5	Pd(COD)Cl₂ (10.0)	dppf (10.0)	100	86	>99:1	n.d.
12	2.5	Pd(COD)Cl₂ (10.0)	none	100	0	n.d.	n.d.
13	2.5	[Pd(dppf)Cl₂]·CH₂Cl₂ (5.0)	none	100	0	n.d.	n.d.
14[c]	2.5	none	none	100	0	n.d.	n.d.
15	2.5	Pd(COD)Cl₂ (10.0)	Trost ligand (10.0)	100	56	>99:1	52:48
16	2.5	Pd(COD)Cl₂ (10.0)	(*R*)-BINAP (10.0)	100	36	>99:1	54:46
17	2.5	Pd(COD)Cl₂ (10.0)	(*R*)-SEGPHOS (10.0)	100	45	>99:1	52:48
18	2.5	Pd(COD)Cl₂ (10.0)	(*S,S*)-BDPP (10.0)	100	97	>99:1	50:50
19	2.5	Pd(COD)Cl₂ (10.0)	(*R*)-Xyl-BINAP (10.0)	100	14	>99:1	49:51

[a]Unless specified otherwise, reactions were carried out using 0.1 mmol **5a** and 0.5 mL solvent under argon. [b]Yields and *Z/E* ratios were determined by ^{19}F NMR analysis of the crude mixture using benzotrifluoride as internal standard. [c]Without LiOH. [d]The enantiomeric ratio (e.r.) was determined by HPLC on a chiral stationary phase column (Column: IB, hexane:isopropanol = 99:1, rate = 1 mL/min, rt).

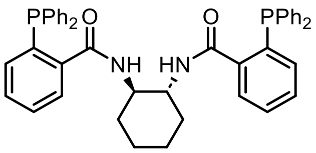

Trost Ligand

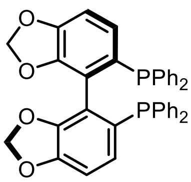

(*R*)-SEGPHOS

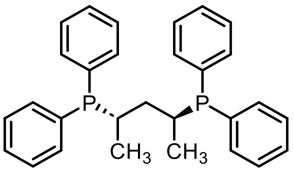

(*S,S*)-BDPP

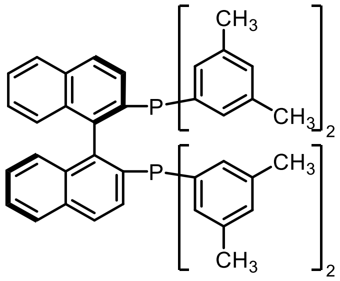

(*R*)-Xyl-BINAP

VI. ^{19}F-^{1}H HOESY NMR Studies

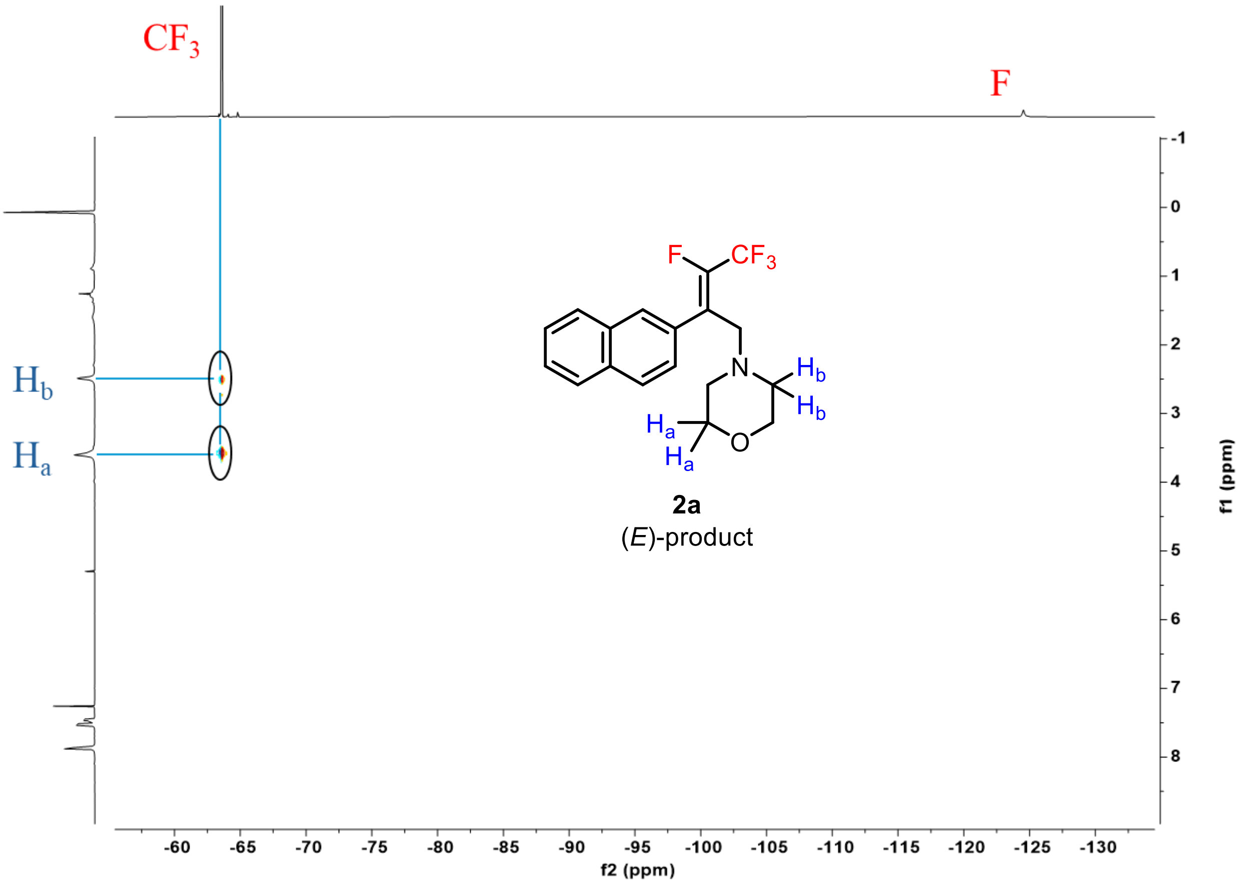

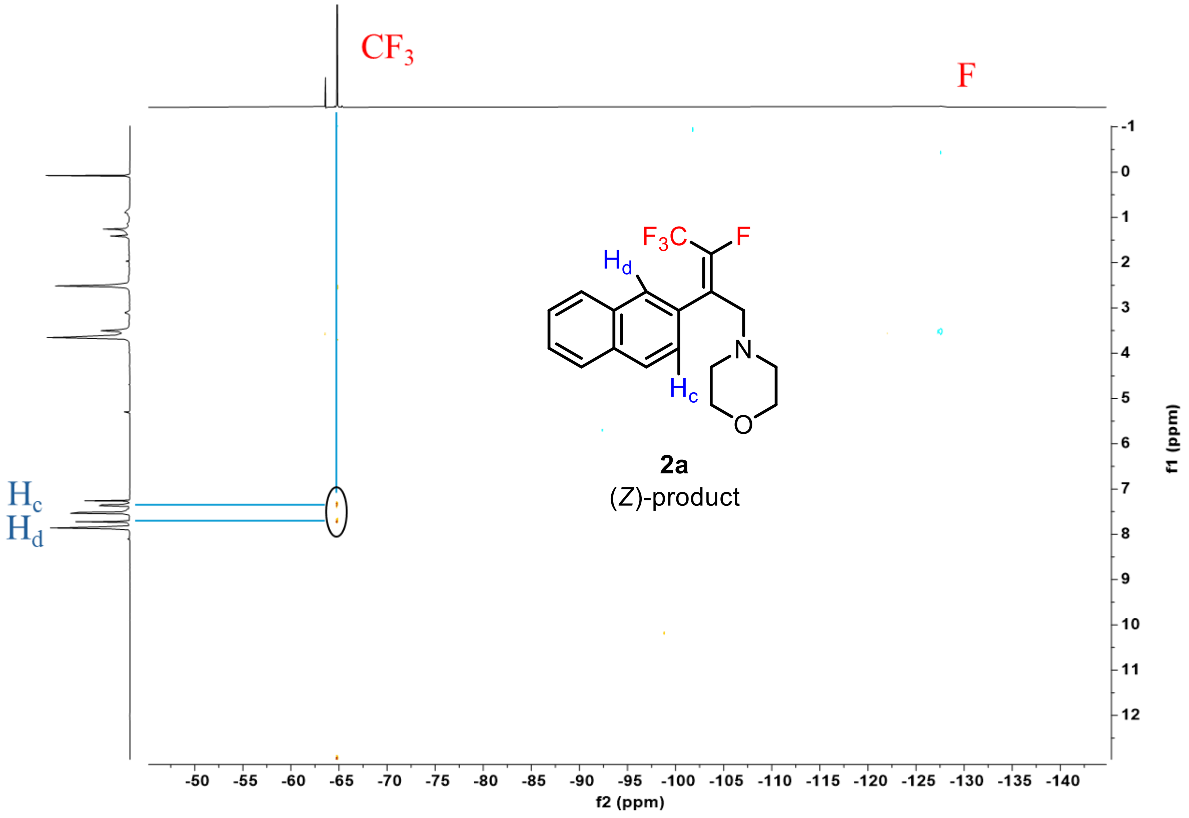

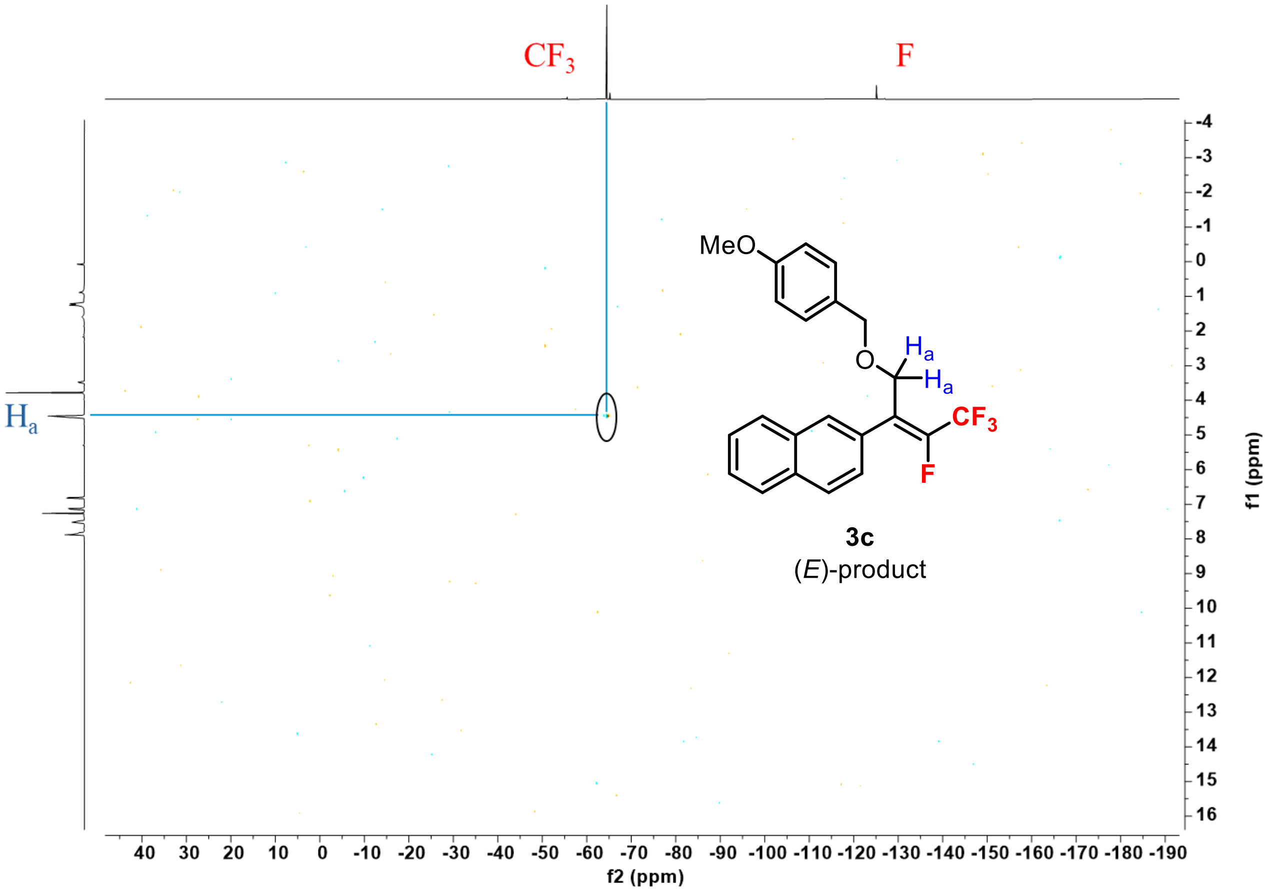
CF3
F
Ha
MeO
O
Ha
Ha
CF3
F
3c
(E)-product
f1 (ppm)
f2 (ppm)

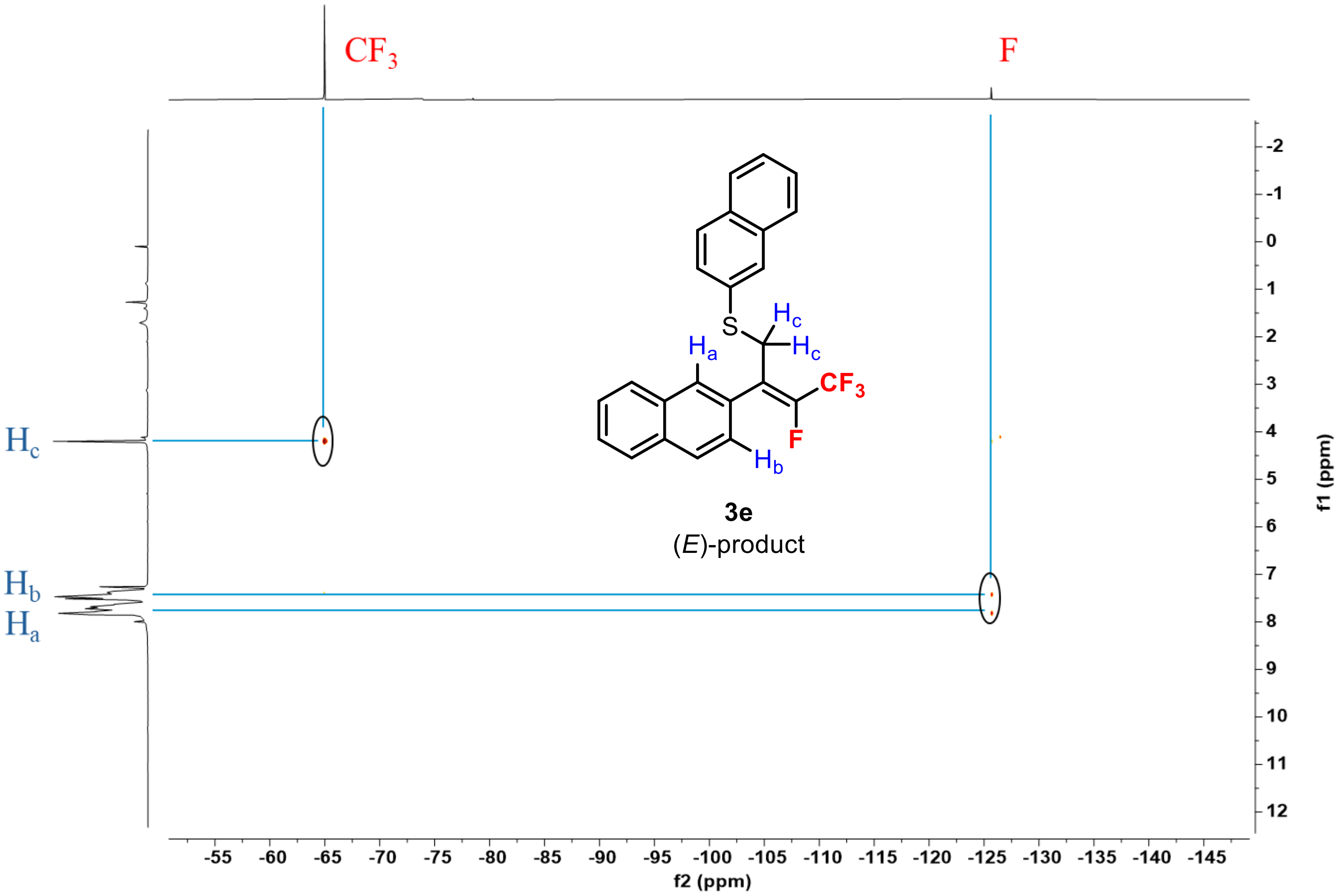
CF3
F
Hc
Hb
Ha
S
Hc
Hc
Ha
CF3
F
Hb
3e
(E)-product
f1 (ppm)
f2 (ppm)

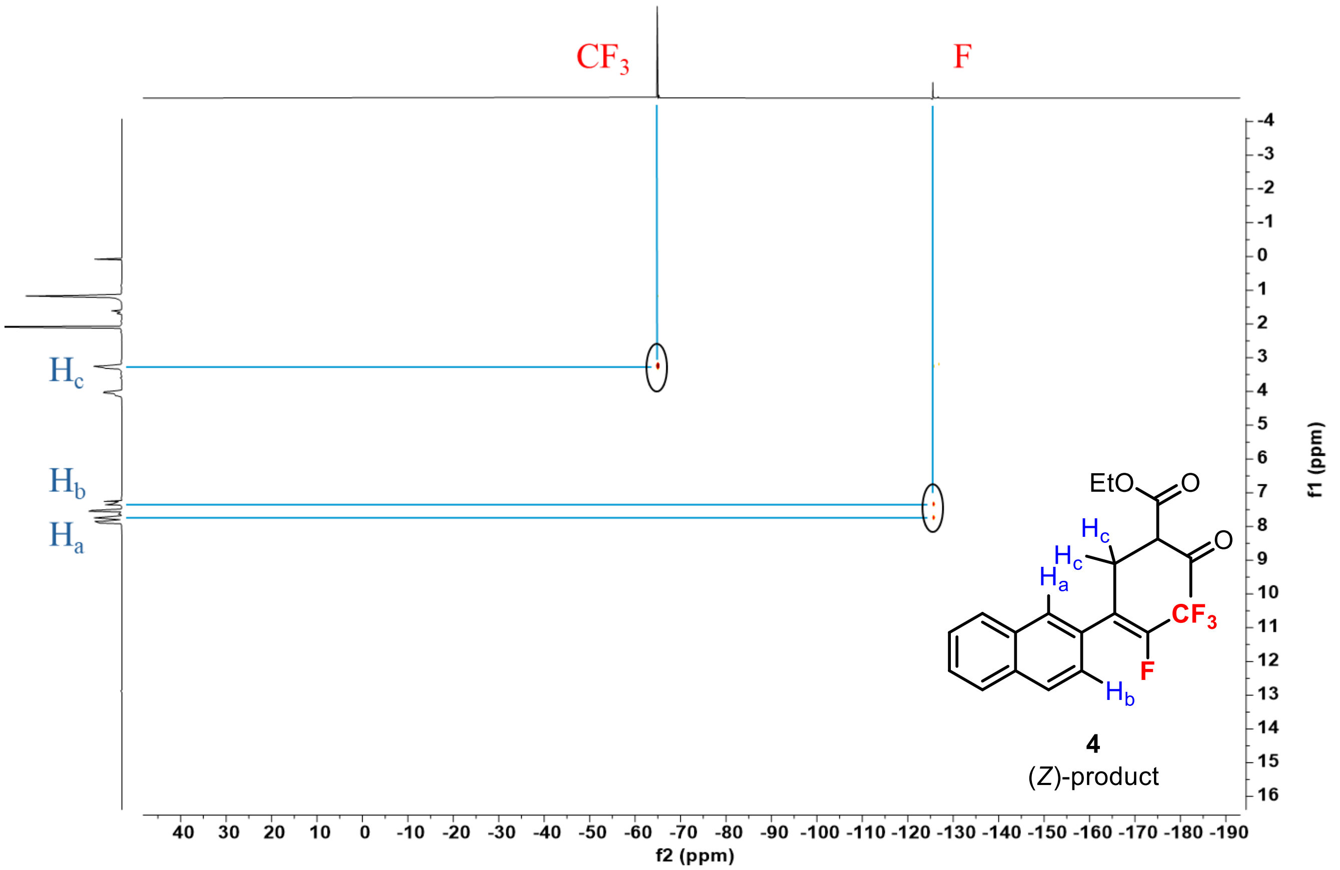
CF3
F
Hc
Hb
Ha
EtO
O
CF3
F
4
(Z)-product
f2 (ppm)
f1 (ppm)

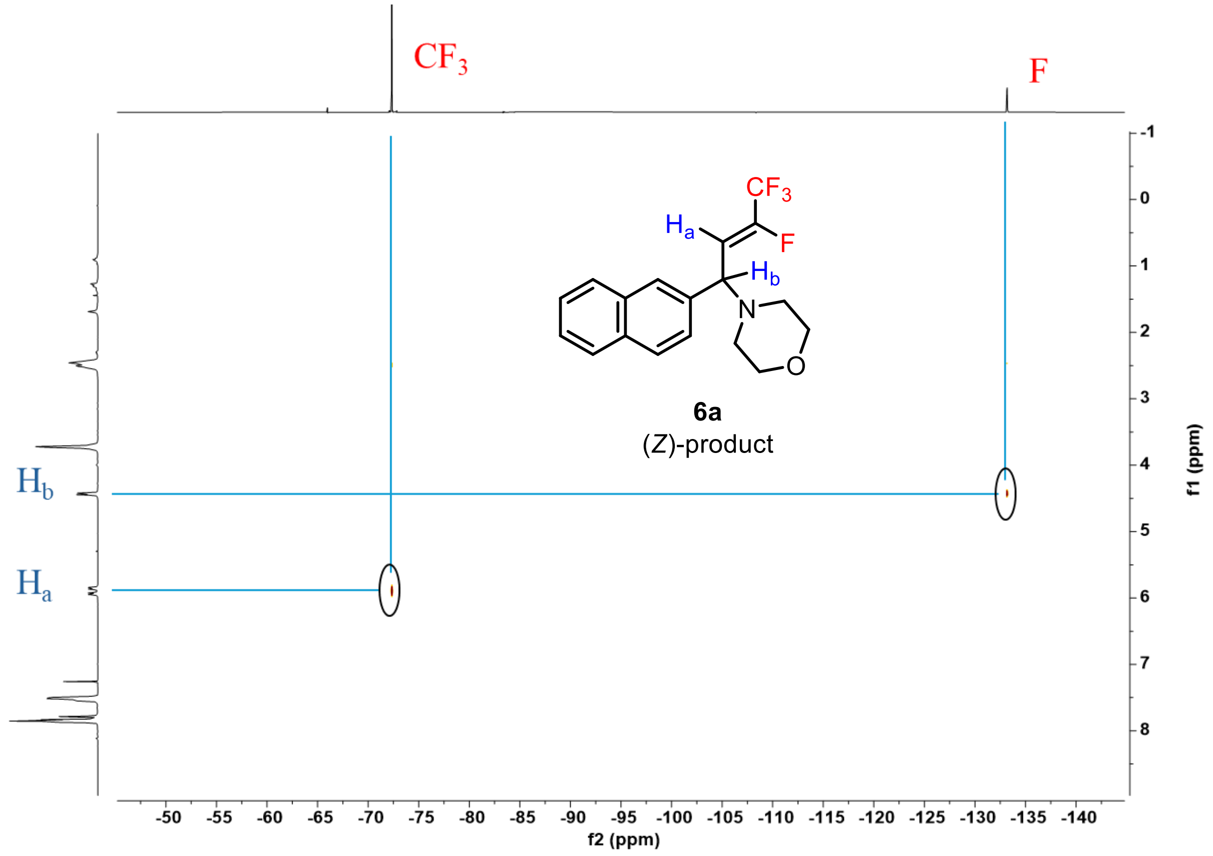
CF3
F
Hb
Ha
N
O
6a
(Z)-product
f2 (ppm)
f1 (ppm)

VII. Characterization Data of Substrates

methyl(4-(3,3,4,4,4-pentafluorobut-1-en-2-yl)phenyl)sulfane (1b)

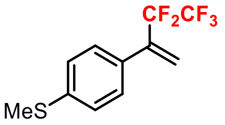

Following the general procedure (II), *a*-CF_2CF_3 ketone **S1b** (1.08 g, 4 mmol) was converted to **1b** as a colorless oil (0.65 g, 61% yield), $R_f = 0.55$ (hexane). **^{1}H NMR** (500 MHz, Chloroform-*d*) δ 7.34 (d, *J* = 8.1 Hz, 2H), 7.27 (dd, *J* = 8.4, 1.5 Hz, 2H), 6.01 (d, *J* = 1.5 Hz, 1H), 5.80 (t, *J* = 1.5 Hz, 1H), 2.53 (s, 3H). **^{13}C NMR** (126 MHz, Chloroform-*d*) δ 140.0 (d, *J* = 2.0 Hz), 138.0 (t, *J* = 21.4 Hz), 131.2, 128.8, 125.9, 124.3 (t, *J* = 8.6 Hz), 119.1 (qt, *J* = 287.0, 38.3 Hz), 113.0 (tq, *J* = 254.2, 37.8 Hz), 15.2 (d, *J* = 3.0 Hz). **^{19}F NMR** (471 MHz, Chloroform-*d*) δ -82.86 (s, 3F), -113.20 (s, 2F). **HRMS** (APCI) m/z: $[M+H]^+$ Calcd for $C_{11}H_{10}F_5S$ 269.0418; Found 269.0418.

1-nitro-3-(3,3,4,4,4-pentafluorobut-1-en-2-yl)benzene (1c)

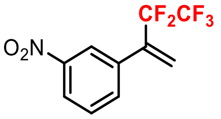

Following the general procedure (II), *a*-CF_2CF_3 ketone **S1c** (1.08 g, 4 mmol) was converted to **1c** as a colorless oil (0.80 g, 75% yield), $R_f = 0.3$ (hexane : DCM = 4:1). **^{1}H NMR** (500 MHz, Chloroform-*d*) δ 8.35 – 8.11 (m, 2H), 7.71 (d, *J* = 7.7 Hz, 1H), 7.58 (t, *J* = 7.8 Hz, 1H), 6.16 (d, *J* = 2.0 Hz, 1H), 5.92 (t, *J* = 1.6 Hz, 1H). **^{13}C NMR (**126 MHz, Chloroform-*d*) δ 148.2, 136.6 (td, *J* = 22.1, 2.5 Hz), 136.2, 134.5, 129.6, 126.9 (t, *J* = 8.2 Hz), 123.8 (d, *J* = 1.8 Hz), 123.6, 118.8 (qt, *J* = 286.7, 38.0 Hz), 112.5 (tq, *J* = 255.0, 38.2 Hz). **^{19}F NMR** (471 MHz, Chloroform-*d*) δ -82.86 – -83.12 (m, 3F), -113.43 – -113.79 (m, 2F). **HRMS** (APCI) m/z: $[M]^-$ Calcd for $C_{10}H_6F_5NO_2$ 267.0324; Found 267.0325.

5-(3,3,4,4,4-pentafluorobut-1-en-2-yl)benzo[*d*][1,3]dioxole (1d)

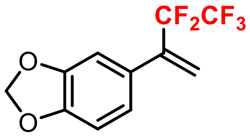

Following the general procedure (II), *a*-CF_2CF_3 ketone **S1d** (1.07 g, 4 mmol) was converted to **1d** as a colorless oil (0.66 g, 62% yield), $R_f = 0.43$ (hexane). **^{1}H NMR** (500 MHz, $CDCl_3$): δ (ppm) 6.91 – 6.80 (m, 3H), 6.01 (s, 2H), 5.97 (s, 1H), 5.76 (s, 1H). **^{13}C NMR** (126 MHz, $CDCl_3$): δ (ppm) 148.3, 147.7, 138.2 (t, *J* = 21.3 Hz), 128.6, 124.3 (t, *J* = 8.4 Hz), 122.7, 119.2 (qt, *J* = 286.8, 38.3 Hz), 113.1 (tq, *J* = 254.6, 37.8 Hz), 109.1, 108.3, 101.5. **^{19}F NMR** (471 MHz, $CDCl_3$): δ (ppm) -82.86 (s, 3F), -113.28 (s, 2F). **HRMS** (APCI) m/z: $[M+H]^+$ Calcd for $C_{11}H_8F_5O_2$ 267.0439; Found 267.0435.

4-methyl-*N*-(4,4,5,5,5-pentafluoro-3-methylenepentyl)-*N* phenylbenzenesulfonamide (1g)

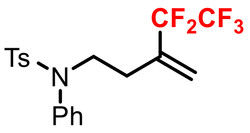

Following the general procedure (IV), corresponding alkenyl iodide (427.0 mg, 1 mmol) was converted to **1g** as a white solid; **(**0.34 g, 80% yield), $R_f = 0.50$ (EA: Hexane = 1:20) **^{1}H NMR** (500 MHz, $CDCl_3$) δ 7.48 (d, *J* = 8.2 Hz, 2H), 7.36 – 7.32 (m, 3H), 7.27 (d, *J* = 8.0 Hz, 2H), 7.07 – 7.00 (m, 2H), 5.81 (s, 1H), 5.72 (s, 1H), 3.75 (t, *J* = 7.3 Hz, 2H), 2.44 (s, 3H), 2.38 (t, *J* = 7.3 Hz, 2H). **^{13}C NMR** (126 MHz, $CDCl_3$) δ 143.8, 138.7, 134.9, 134.1 (t, *J* = 21.8 Hz), 129.6, 129.3, 128.8, 128.3, 127.8, 123.3 (t, *J* = 8.6 Hz), 119.0 (qt, *J* = 286.6, 38.6 Hz), 113.1 (tq, *J* = 253.8, 37.8 Hz), 49.0, 28.8, 21.6. **^{19}F NMR** (471 MHz, $CDCl_3$) δ -83.85 (s, 3F), -116.32 (s, 2F). **HRMS** (ESI) m/z: $[M+Na]^+$ Calcd for $C_{19}H_{18}F_5NO_2SNa$ 442.0871; Found 442.0869.

(*E*)-(((5,5,6,6,6-pentafluorohex-3-en-1-yl)oxy)methyl)benzene (5n)

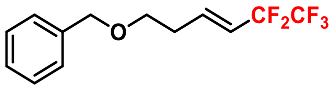

Following the general procedure (IV), alkenyl iodide **S5n** (865 mg, 3 mmol) was converted to **5n** as a colorless oil (706 mg, 84% yield), $R_f = 0.55$ (hexane : DCM = 2:1). **^{1}H NMR** (500 MHz, Chloroform-*d*) δ 7.34 (dq, *J* = 16.4, 8.0 Hz, 5H), 6.48 (dtt, *J* = 15.8, 6.9, 2.0 Hz, 1H), 5.69 (dt, *J* = 16.1, 11.9 Hz, 1H), 4.53 (s, 2H), 3.59 (t, *J* = 6.3 Hz, 2H), 2.66 – 2.33 (m, 2H). **^{13}C NMR** (101 MHz, Chloroform-*d*) δ 139.9 (t, *J* = 8.9 Hz), 138.2, 128.6, 127.9, 127.8, 124.2 – 116.8 (m), 118.4 (t, *J* = 23.1 Hz), 116.0 – 108.2 (m), 73.2, 68.2 (d, *J* = 1.7 Hz), 32.6. **^{19}F NMR** (471 MHz, Chloroform-*d*) δ -85.34 (s, 3F), -115.31 (d, *J* = 11.4 Hz, 2F). **HRMS** (ESI) m/z: $[M+Na]^+$ Calcd for $C_{13}H_{13}F_5ONa$ 303.0779; Found 303.0779.

VIII. Characterization Data of Products

(E)-4-(3,4,4,4-tetrafluoro-2-(naphthalen-2-yl)but-2-en-1-yl)morpholine (2a)

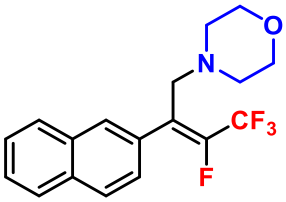

Prepared according to general procedures V using styrene **1a** (54.4 mg, 0.2 mmol) and morpholine (20.9 mg, 0.24 mmol). The product was purified by flash column chromatography on silica gel (ethyl acetate: hexane = 1: 60) and obtained as a yellow oil (42.1 mg, 62% yield, *E/Z* >99:1, *E* isomer), R_f = 0.42 (ethyl acetate: hexane = 1: 5). ^{1}**H NMR** (400 MHz, $CDCl_3$): δ (ppm) 7.89 – 7.84 (m, 4H), 7.55 – 7.50 (m, 2H), 7.46 (dt, *J* = 8.6, 1.6 Hz, 1H), 3.59 (t, *J* = 4.6 Hz, 4H), 3.55 (s, 2H), 2.47 (t, *J* = 4.6 Hz, 4H). ^{13}**C NMR** (126 MHz, $CDCl_3$): δ (ppm) 144.2 (dq, *J* = 257.8, 38.1 Hz), 133.2, 133.0, 131.7, 128.4, 128.0, 127.9 (d, *J* = 3.4 Hz), 127.8, 126.8, 126.5, 125.9 (d, *J* = 2.7 Hz), 124.8, 119.8 (dd, *J* = 275.1, 43.0 Hz), 66.9, 56.2, 53.2. ^{19}**F NMR** (471 MHz, $CDCl_3$): δ (ppm) -64.70 (d, *J* = 7.6 Hz, 3F), -125.63 (s, 1F). **HRMS** m/z (ESI): calcd. for $C_{18}H_{18}F_4NO$ $[M+H]^+$: 340.1319; found: 340.1316.

(E)-4-(3,4,4,4-tetrafluoro-2-(4-(methylthio)phenyl)but-2-en-1-yl)morpholine (2b)

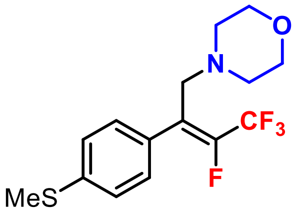

Prepared according to general procedures V using styrene **1b** (53.6 mg, 0.2 mmol) and morpholine (20.9 mg, 0.24 mmol). The product was purified by flash column chromatography on silica gel (ethyl acetate: hexane = 1: 60) and obtained as a yellow oil (46.2 mg, 69% yield, *E/Z* >99:1, *E* isomer), R_f = 0.42 (ethyl acetate: hexane = 1: 5). ^{1}**H NMR** (500 MHz, $CDCl_3$): δ (ppm) 7.34 – 7.30 (m, 2H), 7.28 – 7.22 (m, 2H), 3.60 (t, *J* = 4.7 Hz, 4H), 3.41 (s, 2H), 2.50 (s, 3H), 2.42 (t, *J* = 4.6 Hz, 4H). ^{13}**C NMR** (126 MHz, $CDCl_3$): δ (ppm) 144.0 (dq, *J* = 257.7, 38.3 Hz), 139.6, 130.5, 128.9 (d, *J* = 3.3 Hz), 125.8, 124.0, 119.7 (qd, *J* = 273.8, 42.6 Hz), 66.9, 55.9, 53.1, 15.4. ^{19}**F NMR** (471 MHz, $CDCl_3$): δ (ppm) -64.58 (d, *J* = 7.9 Hz, 3F), -125.91 (s, 1F). **HRMS** m/z (ESI): calcd. for $C_{15}H_{18}F_4NOS$ $[M+H]^+$: 336.1040; found: 336.1036.

(E)-4-(3,4,4,4-tetrafluoro-2-(3-nitrophenyl)but-2-en-1-yl)morpholine (2c)

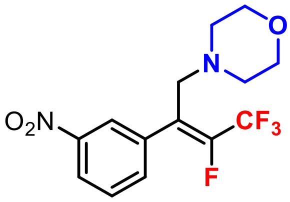

Prepared according to general procedures V using styrene **1c** (53.4 mg, 0.2 mmol) and morpholine (20.9 mg, 0.24 mmol). The product was purified by flash column chromatography on silica gel (ethyl acetate: hexane = 1: 60) and obtained as a yellow oil (42.7 mg, 64% yield, *E/Z* >99:1, *E* isomer), R_f = 0.32 (ethyl acetate: hexane = 1: 5). ^{1}**H NMR** (500 MHz, $CDCl_3$): δ (ppm) 8.39 – 8.31 (m, 1H), 8.25 (dd, *J* = 8.3, 2.3 Hz, 1H), 7.82 – 7.68 (m, 1H), 7.59 (t, *J* = 8.0 Hz, 1H), 3.62 (t, *J* = 4.7 Hz, 4H), 3.47 (s, 2H), 2.47 (t, *J* = 4.8 Hz, 4H). ^{13}**C NMR** (101 MHz, $CDCl_3$): δ (ppm) 148.3, 145.1 (dq, *J* = 261.4, 38.7 Hz), 135.5, 134.8, 134.7, 129.4, 123.74, 123.71, 119.3 (qd, *J* = 274.2, 42.0 Hz), 66.7, 55.7, 53.1. ^{19}**F NMR** (471 MHz, $CDCl_3$): δ (ppm) -64.85 (d, *J* = 7.7 Hz, 3F), -123.15 (s, 1F). **HRMS** m/z (ESI): calcd. for $C_{14}H_{15}F_4N_2O_3$ $[M+H]^+$: 335.1013; found: 335.1012.

(E)-4-(2-(benzo[d][1,3]dioxol-5-yl)-3,4,4,4-tetrafluorobut-2-en-1-yl)morpholine (2d)

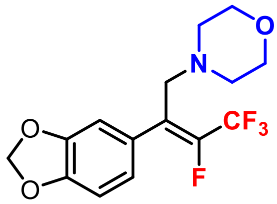

Prepared according to general procedures V using styrene **1d** (53.2 mg, 0.2 mmol) and morpholine (20.9 mg, 0.24 mmol). The product was purified by flash column chromatography on silica gel (ethyl acetate: hexane = 1: 60) and obtained as a yellow oil (38.5 mg, 58% yield, *E/Z* = 96:4, *E* isomer), R_f = 0.35 (ethyl acetate: hexane = 1: 5). **^{1}H NMR** (500 MHz, $CDCl_3$): δ (ppm) 6.92 – 6.80 (m, 3H), 6.00 (s, 2H), 3.62 (t, J = 4.7 Hz, 4H), 3.37 (t, J = 2.1 Hz, 2H), 2.43 (t, J = 4.7 Hz, 4H). **^{13}C NMR** (126 MHz, $CDCl_3$): δ (ppm) 147.9, 147.7, 144.0 (dq, J = 256.7, 38.3 Hz), 127.7, 122.4 (d, J = 3.7 Hz), 119.8 (qd, J = 273.5, 42.6 Hz), 109.0 (d, J = 3.5 Hz), 108.3, 101.4, 67.0, 56.3 (dd, J = 3.6, 1.9 Hz), 53.5, 53.2. **^{19}F NMR** (471 MHz, $CDCl_3$): δ (ppm) -64.57 (d, J = 8.5 Hz, 3F), -125.92 (q, J = 7.5 Hz, 1F). **HRMS** m/z (ESI): calcd. for $C_{15}H_{16}F_4NO_3$ $[M+H]^+$: 334.1061; found: 334.1058.

(E)-N,N-diethyl-3,4,4,4-tetrafluoro-2-(naphthalen-2-yl)but-2-en-1-amine (2e)

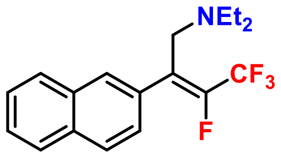

Prepared according to general procedures V using styrene **1a** (54.4 mg, 0.2 mmol) and Diethylamine (17.5 mg, 0.24 mmol). The product was purified by flash column chromatography on silica gel (ethyl acetate: hexane = 1: 60) and obtained as a yellow oil (33.7 mg, 52% yield, *E/Z* >99:1, *E* isomer), R_f = 0.37 (ethyl acetate: hexane = 1: 5). **^{1}H NMR** (500 MHz, $CDCl_3$): δ (ppm) 7.88 – 7.82 (m, 4H), 7.53 – 7.49 (m, 2H), 7.44 (d, J = 8.5 Hz, 1H), 3.61 (s, 2H), 2.50 (q, J = 7.2 Hz, 4H), 0.92 (t, J = 7.2 Hz, 6H). **^{13}C NMR** (126 MHz, $CDCl_3$): δ (ppm) 143.5 (dq, J = 253.5, 41.4 Hz), 133.2, 133.1, 131.7, 128.4, 128.03, 128.00, 127.8, 126.6, 126.4, 126.1 (2C), 119.9 (qd, J = 273.6, 43.2 Hz), 51.0, 46.5, 11.6. **^{19}F NMR** (471 MHz, $CDCl_3$): δ (ppm) -64.79 (d, J = 8.2 Hz, 3F), -126.94 (s, 1F). **HRMS** m/z (ESI): calcd. for $C_{18}H_{20}F_4N$ $[M+H]^+$: 326.1526; found: 326.1524.

N-(3,4,4,4-tetrafluoro-2-(naphthalen-2-yl)but-2-en-1-yl)aniline (2f)

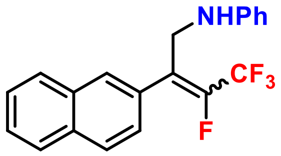

Prepared according to general procedures V using styrene **1a** (54.4 mg, 0.2 mmol) and aniline (22.4 mg, 0.24 mmol). The product was purified by flash column chromatography on silica gel (ethyl acetate: hexane = 1: 60) and obtained as a yellow oil (41.4 mg, 60% yield, E/Z = 50:50), R_f = 0.57 (DCM: hexane = 1: 1). **^{1}H NMR** (500 MHz, $CDCl_3$): δ (ppm) 7.91 – 7.77 (m, 7H), 7.62 – 7.47 (m, 5H), 7.42 (dt, J = 8.6, 1.6 Hz, 1H), 7.26 – 7.15 (m, 5H), 6.80 (q, J = 7.6 Hz, 2H), 6.60 (dd, J = 8.2, 6.8 Hz, 4H), 4.41 – 4.36 (m, 2H), 4.33 (dd, J = 3.0, 1.5 Hz, 2H), 3.28 (bs, 2H). **^{13}C NMR** (126 MHz, $CDCl_3$): δ (ppm) 146.9, 146.7, 143.8 (dq, J = 259.1, 38.6 Hz), 143.7 (dq, J = 256.9, 37.8 Hz), 133.4, 133.2, 133.1, 132.9, 130.1, 129.5, 129.44, 129.39, 128.6, 128.4, 128.3, 128.19, 128.15, 128.12, 128.06 (d, J = 2.0 Hz), 128.0 (d, J = 1.5 Hz), 127.88, 127.85, 127.8, 127.1, 126.9, 126.8, 125.9 (dd, J = 3.1, 1.5 Hz), 125.5 (d, J = 3.1 Hz), 123.4 – 117.0 (m), 118.9, 118.7, 113.9, 113.5, 43.4 (d, J = 6.2 Hz), 43.0 – 42.4 (m). **^{19}F NMR** (471 MHz, $CDCl_3$): δ (ppm) -65.80 (d, J = 8.4 Hz, 3F), -66.41 (d, J = 9.6 Hz, 3F) , -126.47 (q, J = 8.3 Hz, 1F), -129.57 (q, J = 8.6 Hz, 1F). **HRMS** m/z (APCI): calcd. for $C_{20}H_{16}F_4N$ $[M+H]^+$: 346.1213; found: 346.1210.

(E)-4-methyl-N-phenyl-N-(4,5,5,5-tetrafluoro-3-(morpholinomethyl)pent-3-en-1-yl)benzenesulfonamide (2g)

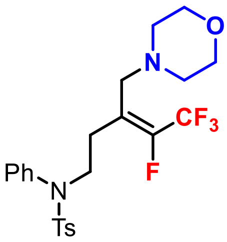

Prepared according to general procedures V using **1g** (83.8 mg, 0.2 mmol) and morpholine (20.9 mg, 0.24 mmol). The product was purified by flash column chromatography on silica gel (ethyl acetate: hexane = 1: 60) and obtained as a yellow oil (41.8 mg, 43% yield, *E/Z* = 98:2, *E* isomer), R_f = 0.27 (DCM: hexane = 1: 1). **^{1}H NMR** (500 MHz, $CDCl_3$): δ (ppm) 7.44 (dd, *J* = 8.4, 2.2 Hz, 2H), 7.34 – 7.28 (m, 3H), 7.28 – 7.22 (m, 2H), 7.04 (dt, *J* = 7.5, 2.4 Hz, 2H), 3.73 (t, *J* = 6.7 Hz, 2H), 3.63 (s, 4H), 3.13 (s, 2H), 2.48 – 2.43 (m, 2H), 2.42 (s, 3H), 2.37 (t, *J* = 4.4 Hz, 4H). **^{13}C NMR** (126 MHz, $CDCl_3$): δ (ppm) 144.6 (dq, *J* = 253.2, 38.5 Hz), 143.7, 138.8, 134.9, 129.6, 129.1, 128.7, 128.2, 127.8, 122.3 (dq, *J* = 10.1, 2.5 Hz), 119.2 (qd, *J* = 273.4, 42.9 Hz), 67.0, 53.6, 53.5, 47.8, 26.5 (d, *J* = 5.5 Hz), 21.7. **^{19}F NMR** (471 MHz, $CDCl_3$): δ (ppm) -64.79 (d, *J* = 7.8 Hz, 3F), -126.21 – -126.53 (m, 1F). **HRMS** m/z (ESI): calcd. for $C_{23}H_{27}F_4N_2O_3S$ $[M+H]^+$: 487.1673; found: 487.1673.

(E)-2-(1-(benzyloxy)-3,4,4,4-tetrafluorobut-2-en-2-yl)naphthalene (3a)

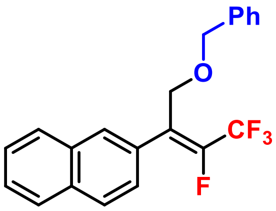

Prepared according to general procedures VI using styrene **1a** (54.4 mg, 0.2 mmol) and Benzyl alcohol (34.6 mg, 0.32 mmol). The product was purified by flash column chromatography on silica gel (ethyl acetate: hexane = 1: 60) and obtained as a yellow oil (43.8 mg, 61% yield, *E/Z* >99:1, *E* isomer), R_f = 0.34 (ethyl acetate: hexane = 1: 5). **^{1}H NMR** (500 MHz, $CDCl_3$): δ (ppm) 7.94 – 7.78 (m, 4H), 7.58 – 7.48 (m, 3H), 7.34 – 7.26 (m, 3H), 7.25 – 7.20 (m, 2H), 4.62 – 4.45 (m, 4H). **^{13}C NMR** (101 MHz, $CDCl_3$): δ (ppm) 144.8 (dq, J = 261.6, 38.6 Hz), 137.4, 133.3, 133.1, 130.7, 128.6, 128.5, 128.3, 128.2, 128.05, 128.02, 127.8, 127.0, 126.6, 125.8 (d, J = 3.4 Hz), 124.8 – 124.4 (m), 119.6 (qd, J = 273.9, 42.2 Hz), 72.9, 66.1 (dq, J = 5.5, 2.8 Hz). **^{19}F NMR** (471 MHz, $CDCl_3$): δ (ppm) -65.70 (d, J = 8.0 Hz, 3F), -126.13 (q, J = 8.3 Hz, 1F). **HRMS** m/z (ESI): calcd. for $C_{21}H_{16}F_4ONa$ $[M+Na]^+$: 383.1030; found: 383.1021.

(E)-2-(3,4,4,4-tetrafluoro-1-((3-nitrobenzyl)oxy)but-2-en-2-yl)naphthalene (3b)

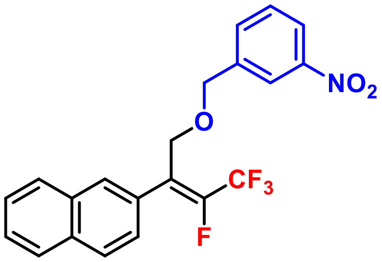

Prepared according to general procedures VI using styrene **1a** (54.4 mg, 0.2 mmol) and 3-Nitrobenzyl alcohol (49.0 mg, 0.32 mmol). The product was purified by flash column chromatography on silica gel (ethyl acetate: hexane = 1: 60) and obtained as a yellow oil (55.8 mg, 69% yield, *E/Z* = 95:5, *E* isomer), R_f = 0.32 (ethyl acetate: hexane = 1: 5). **^{1}H NMR** (500 MHz, $CDCl_3$): δ (ppm) 8.12 – 8.03 (m, 2H), 7.93 – 7.80 (m, 4H), 7.57 – 7.47 (m, 4H), 7.42 (t, *J* = 7.9 Hz, 1H), 4.62 (s, 2H), 4.60 (s, 2H). **^{13}C NMR** (126 MHz, $CDCl_3$): δ (ppm) 148.4, 145.0 (dq, *J* = 262.4, 38.7 Hz), 139.6, 133.5, 133.3, 133.1, 130.2, 129.5, 128.5, 128.4, 128.23, 128.20, 127.9, 127.1, 126.8, 125.6 (d, *J* = 3.4 Hz), 122.9, 122.5, 119.6 (qd, *J* = 274.0, 42.1 Hz), 71.4, 66.6 (dq, *J* = 5.6, 2.8 Hz). **^{19}F NMR** (471 MHz, $CDCl_3$): δ (ppm) -65.65 (d, *J* = 8.1 Hz, 3F), -125.42 (q, *J* = 7.6 Hz, 1F). **HRMS** m/z (ESI): calcd. for $C_{21}H_{15}F_4NO_3Na$ $[M+Na]^+$:

428.0880; found: 428.0871.

(E)-2-(3,4,4,4-tetrafluoro-1-((4-methoxybenzyl)oxy)but-2-en-2-yl)naphthalene (3c)

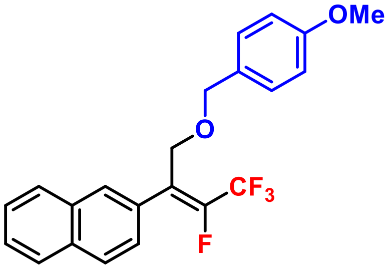

Prepared according to general procedures VI using styrene **1a** (54.4 mg, 0.2 mmol) and 4-Methoxybenzyl alcohol (44.2 mg, 0.32 mmol). The product was purified by flash column chromatography on silica gel (ethyl acetate: hexane = 1: 60) and obtained as a yellow oil (44.5 mg, 57% yield, *E/Z* = 93:7, *E* isomer), R_f = 0.46 (ethyl acetate: hexane = 1: 10). **^{1}H NMR** (500 MHz, $CDCl_3$): δ (ppm) 7.95 – 7.72 (m, 4H), 7.56 – 7.45 (m, 3H), 7.18 – 7.06 (m, 2H), 6.87 – 6.76 (m, 2H), 4.49 (t, *J* = 2.1 Hz, 2H), 4.45 (s, 2H), 3.78 (s, 3H). **^{13}C NMR** (126 MHz, $CDCl_3$): δ (ppm) 159.5, 144.8 (dq, *J* = 261.2, 38.6 Hz), 133.3, 133.1, 130.7, 129.7, 129.6, 129.5, 128.5, 128.24 (d, *J* = 3.4 Hz), 128.20, 127.8, 126.9, 126.6, 125.9 (d, *J* = 3.5 Hz), 121.9 – 116.5 (m), 113.9, 72.6, 65.7 (dq, *J* = 5.5, 2.7 Hz), 55.4. **^{19}F NMR** (471 MHz, $CDCl_3$): δ (ppm) -65.72 (d, *J* = 7.9 Hz, 3F), -126.31 (q, *J* = 8.4 Hz, 1F). **HRMS** m/z (ESI): calcd. for $C_{22}H_{18}F_4O_2Na$ $[M+Na]^+$: 413.1135; found: 413.1127.

2-(3,4,4,4-tetrafluoro-1-phenoxybut-2-en-2-yl)naphthalene (3d)

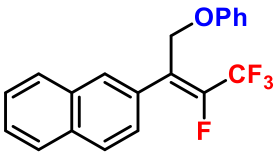

Prepared according to general procedures VI using styrene **1a** (54.4 mg, 0.2 mmol) and Phenol (30.1 mg, 0.32 mmol). The product was purified by flash column chromatography on silica gel (ethyl acetate: hexane = 1: 60) and obtained as a yellow oil (53.3 mg, 77% yield, *E/Z* = 60:40), R_f = 0.56 (ethyl acetate: hexane = 1: 10). **^{1}H NMR** (500 MHz, $CDCl_3$): δ (ppm) 8.03 (s, 1H), 7.97 – 7.85 (m, 6H), 7.79 (s, 1H), 7.65 – 7.52 (m, 5H), 7.45 – 7.27 (m, 5H), 7.12 – 6.87 (m, 6H), 5.13 – 4.96 (m, 4H). **^{13}C NMR** (126 MHz, $CDCl_3$): δ (ppm) 158.3, 158.1, 145.5 (dq, *J* = 262.4, 39.1 Hz), 144.6 (dq, *J* = 261.5, 37.9 Hz), 133.4, 133.3, 133.1, 132.9, 130.4, 129.71, 129.69, 129.4, 129.2 (d, *J* = 4.3 Hz), 128.5, 128.4, 128.3, 128.24, 128.15, 127.9, 127.8, 127.1, 126.9, 126.7, 126.6, 126.1, 125.7 (d, *J* = 3.5 Hz), 124.9, 124.8, 121.8, 121.7, 120.9 – 116.8 (m), 115.1, 114.9, 64.9 (d, *J* = 7.1 Hz), 64.8 (dq, *J* = 6.0, 2.9 Hz). **^{19}F NMR** (471 MHz, $CDCl_3$): δ (ppm) -66.19 (d, *J* = 8.7 Hz, 2F, minor), -66.51 (d, *J* = 8.8 Hz, 3F, major), -125.04 (q, *J* = 8.1 Hz, 1F, minor), -126.63 – -126.76 (m, 1F, major). **HRMS** m/z (APCI): calcd. for $C_{20}H_{15}F_4O$ $[M+H]^+$: 347.1054; found: 347.1046.

(E)-naphthalen-2-yl(3,4,4,4-tetrafluoro-2-(naphthalen-2-yl)but-2-en-1-yl)sulfane (3e)

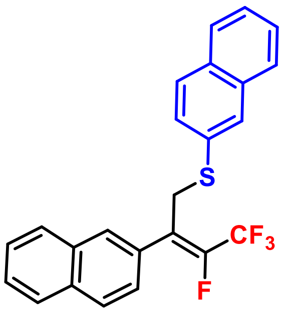

Prepared according to general procedures VI using styrene **1a** (54.4 mg, 0.2 mmol) and 2-Naphthalenethiol (51.3 mg, 0.32 mmol). The product was purified by flash column chromatography on silica gel (ethyl acetate: hexane = 1: 60) and obtained as a yellow solid (55.2 mg, 67% yield, *E/Z* = 97:3, *E* isomer), R_f = 0.49 (ethyl acetate: hexane = 1: 10). **^{1}H NMR** (400 MHz, $CDCl_3$): δ (ppm) 7.87 – 7.78 (m, 3H), 7.81 – 7.73 (m, 2H), 7.75 – 7.66 (m, 2H), 7.67 – 7.60 (m, 1H), 7.55 – 7.49 (m, 2H), 7.48 – 7.44 (m, 2H), 7.42 (dt, *J* = 8.5, 1.6 Hz, 1H), 7.37 (dd, *J* = 8.5, 1.9 Hz, 1H), 4.20 (t, *J* = 1.7 Hz, 2H). **^{13}C NMR** (126 MHz, $CDCl_3$): δ (ppm) 143.2 (dq,

J = 257.6, 38.4 Hz), 133.6, 133.3, 133.0, 132.5, 131.7, 131.1, 130.9, 129.3, 129.1, 128.7, 128.4, 128.32, 128.28 (d, J = 3.2 Hz), 127.82, 127.77, 127.5, 127.0, 126.70, 126.67, 126.5, 125.8 (d, J = 3.1 Hz), 119.6 (dd, J = 273.8, 42.3 Hz), 35.8 – 33.6 (m). **^{19}F NMR** (471 MHz, $CDCl_3$): δ (ppm) -66.19 (d, J = 8.6 Hz, 3F), -126.88 (q, J = 9.0 Hz, 1F). **HRMS** m/z (APCI): calcd. for $C_{24}H_{17}F_4S$ $[M+H]^+$: 413.0982; found: 413.0971.

(E)-2-(3,4,4,4-tetrafluoro-1-(2,2,2-trifluoroethoxy)but-2-en-2-yl)naphthalene (3f)

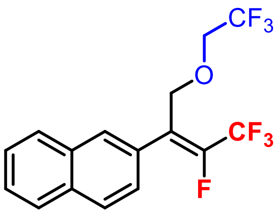

Prepared according to general procedures VI using styrene **1a** (54.4 mg, 0.2 mmol) and trifluoroethanol (32.0 mg, 0.32 mmol). The product was purified by flash column chromatography on silica gel (ethyl acetate: hexane = 1: 60) and obtained as a yellow oil (35.3 mg, 50% yield, *E/Z* = 94:6, *E* isomer), R_f = 0.60 (ethyl acetate: hexane = 1: 10). **^{1}H NMR** (400 MHz, $CDCl_3$): δ (ppm) 7.95 – 7.83 (m, 4H), 7.62 – 7.46 (m, 3H), 4.68 (s, 2H), 3.83 (q, J = 8.6 Hz, 2H). **^{13}C NMR** (126 MHz, $CDCl_3$): δ (ppm) 145.6 (dq, J = 263.7, 38.8 Hz), 133.4, 133.1, 130.0, 128.52, 128.49, 128.3, 128.2, 127.9, 127.2, 126.8, 125.5 (d, J = 3.4 Hz), 125.3 – 122.0 (m), 121.0 – 117.8 (m), 68.3 (d, J = 3.0 Hz), 67.9 (q, J = 34.7 Hz). **^{19}F NMR** (471 MHz, $CDCl_3$): δ (ppm) -65.79 (d, J = 8.3 Hz, 3F), -75.14 (t, J = 8.7 Hz, 3F), -124.97 (q, J = 8.6 Hz, 1F). **HRMS** m/z (APCI): calcd. for $C_{16}H_{11}F_7O$ $[M]^+$: 352.0693; found: 352.0686.

2-(3,4,4,4-tetrafluoro-1-((1,1,1,3,3,3-hexafluoropropan-2-yl)oxy)but-2-en-2-yl)naphthalene (3g)

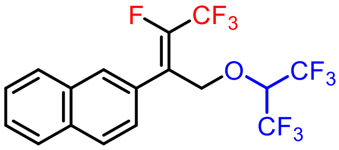

Prepared according to general procedures VI using styrene **1a** (54.4 mg, 0.2 mmol) and 1,1,1,3,3,3-Hexafluoro-2-propanol (53.8 mg, 0.32 mmol). The product was purified by flash column chromatography on silica gel (ethyl acetate: hexane = 1: 60) and obtained as a yellow oil (58.7 mg, 70% yield, *E/Z* = 63:37), R_f = 0.58 (ethyl acetate: hexane = 1: 10). **^{1}H NMR** (500 MHz, $CDCl_3$): δ (ppm) 7.96 – 7.82 (m, 10H), 7.74 (s, 2H), 7.59 – 7.52 (m, 6H), 7.49 (dt, J = 8.6, 1.8 Hz, 1H), 7.34 (dd, J = 8.4, 1.8 Hz, 2H), 4.87 (s, 2H, minor), 4.79 (d, J = 3.8 Hz, 4H, major), 4.19 – 4.02 (m, 3H). **^{13}C NMR** (126 MHz, $CDCl_3$): δ (ppm) 146.6 (dq, J = 265.9, 38.8 Hz), 145.8 (dq, J = 264.4, 38.4 Hz), 133.5, 133.4, 133.1, 133.0, 129.8, 128.83, 128.79, 128.6, 128.53, 128.51, 128.4, 128.3, 128.2, 127.91, 127.86, 127.3, 127.1, 126.9, 125.6 (d, J = 3.0 Hz), 125.3 (d, J = 3.7 Hz), 122.4 (d, J = 3.9 Hz), 124.9 – 115.3 (m), 120.2 (d, J = 3.0 Hz), 77.4 – 75.3 (m), 71.5 (d, J = 7.0 Hz), 70.6 (dq, J = 6.1, 3.4 Hz). **^{19}F NMR** (471 MHz, $CDCl_3$): δ (ppm) -66.11 (d, J = 8.0 Hz, 3F, minor), -66.82 (d, J = 9.1 Hz, 5F, major), -74.90 (d, J = 5.8 Hz, 6F, minor), -75.02 (d, J = 5.9 Hz, 9F, major), -122.69 (q, J = 8.1 Hz, 1F, minor), -125.36 (qd, J = 8.8, 4.4 Hz, 2F, major). **HRMS** m/z (ESI): calcd. for $C_{17}H_{10}F_{10}OCl$ $[M+Cl]^-$: 455.0266; found: 455.0268.

(E)-5-(1-(benzyloxy)-3,4,4,4-tetrafluorobut-2-en-2-yl)benzo[d][1,3]dioxole (3h)

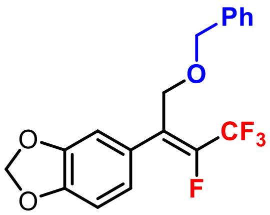

Prepared according to general procedures VI using styrene **1d** (53.2 mg, 0.2 mmol) and Benzyl alcohol (34.6 mg, 0.32 mmol). The product was purified by flash column chromatography on silica gel (ethyl acetate: hexane = 1: 60) and obtained as a yellow oil (31.8 mg, 45% yield, *E/Z* = 93:7, *E* isomer), R_f = 0.48 (ethyl acetate: hexane = 1: 5). **^{1}H NMR** (500 MHz, $CDCl_3$): δ (ppm) 7.36 – 7.29

(m, 3H), 7.28 – 7.22 (m, 2H), 6.99 – 6.87 (m, 2H), 6.84 (dd, J = 8.6, 1.4 Hz, 1H), 6.00 (s, 2H), 4.50 (s, 2H), 4.39 (s, 2H). **^{13}C NMR** (126 MHz, CDCl$_3$): δ (ppm) 148.1, 147.9, 144.5 (dd, J = 261.1, 38.6 Hz), 137.4, 128.6, 128.1, 128.0, 126.7, 122.6 (d, J = 4.0 Hz), 119.6 (dd, J = 273.8, 42.3 Hz), 109.1 (d, J = 4.1 Hz), 108.5, 101.5, 72.84, 72.80, 66.1 (dq, J = 5.5, 2.7 Hz). **^{19}F NMR** (471 MHz, CDCl$_3$): δ (ppm) -65.52 (d, J = 7.8 Hz, 3F), -126.48 (q, J = 8.3 Hz, 1F). **HRMS** m/z (ESI): calcd. for $C_{18}H_{14}F_4O_3Na$ [M+Na]$^+$: 377.0771; found: 377.0762.

(E)-1-(1-(benzyloxy)-3,4,4,4-tetrafluorobut-2-en-2-yl)-3-nitrobenzene (3i)

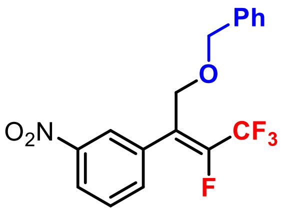

Prepared according to general procedures VI using styrene **1c** (53.4 mg, 0.2 mmol) and Benzyl alcohol (34.6 mg, 0.32 mmol). The product was purified by flash column chromatography on silica gel (ethyl acetate: hexane = 1: 60) and obtained as a yellow oil (42.5 mg, 60% yield, E/Z = 97:3, E isomer), R_f = 0.39 (ethyl acetate: hexane = 1: 5). **^{1}H NMR** (400 MHz, CDCl$_3$): δ (ppm) 8.35 – 8.17 (m, 2H), 7.73 (d, J = 7.7 Hz, 1H), 7.59 (t, J = 8.0 Hz, 1H), 7.37 – 7.27 (m, 3H), 7.26 – 7.19 (m, 2H), 4.52 (s, 2H), 4.45 (s, 2H). **^{13}C NMR** (101 MHz, CDCl$_3$): δ (ppm) 148.4, 145.6 (dd, J = 265.2, 39.2 Hz), 136.9, 134.79, 134.76, 129.6, 128.7, 128.3, 128.1, 123.8, 123.7 (d, J = 3.7 Hz), 122.7 (d, J = 8.5 Hz), 119.2 (dd, J = 274.4, 41.8 Hz), 73.2, 65.4 (dq, J = 5.8, 3.0 Hz). **^{19}F NMR** (471 MHz, CDCl$_3$): δ (ppm) -65.91 (d, J = 7.7 Hz, 3F), -123.71 (q, J = 7.9 Hz, 1F). **HRMS** m/z (ESI): calcd. for $C_{17}H_{13}F_4NO_3Na$ [M+Na]$^+$: 378.0724; found: 378.0724.

(E)-N-(3-((benzyloxy)methyl)-4,5,5,5-tetrafluoropent-3-en-1-yl)-4-methyl-N-phenylbenzenesulfonamide (3j)

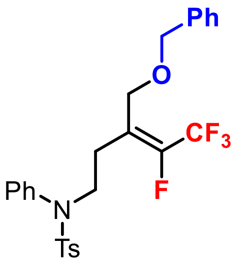

Prepared according to general procedures VI using styrene **1g** (83.8 mg, 0.2 mmol) and Benzyl alcohol (34.6 mg, 0.32 mmol). The product was purified by flash column chromatography on silica gel (ethyl acetate: hexane = 1: 60) and obtained as a yellow oil (69.8 mg, 69% yield, E/Z = 92:8, E isomer), R_f = 0.31 (ethyl acetate: hexane = 1: 5). **^{1}H NMR** (500 MHz, CDCl$_3$): δ (ppm) 7.44 – 7.38 (m, 2H), 7.35 – 7.32 (m, 2H), 7.31 – 7.26 (m, 6H), 7.22 (d, J = 8.1 Hz, 2H), 7.08 – 6.93 (m, 2H), 4.46 (s, 2H), 4.22 – 4.17 (m, 2H), 3.71 (t, J = 7.2 Hz, 2H), 2.59 – 2.46 (m, 2H), 2.42 (s, 3H). **^{13}C NMR** (126 MHz, CDCl$_3$): δ (ppm) 144.7 (dq, J = 255.9, 38.9 Hz), 143.6, 138.9, 137.5, 135.0, 129.5, 129.2, 128.8, 128.6, 128.2, 128.0, 127.9, 127.8, 122.0 (dq, J = 10.7, 2.5 Hz), 119.0 (qd, J = 273.5, 42.8 Hz), 72.9, 64.5 (dq, J = 6.3, 3.2 Hz), 47.9, 26.5 (d, J = 5.4 Hz), 21.7. **^{19}F NMR** (471 MHz, CDCl$_3$): δ (ppm) -66.02 (d, J = 8.0 Hz, 3F), -127.09 – -127.24 (m, 1F). **HRMS** m/z (APCI): calcd. for $C_{26}H_{26}F_4NO_3S$ [M+H]$^+$: 508.1564; found: 508.1562.

(Z)-ethyl 2-acetyl-5,6,6,6-tetrafluoro-4-(naphthalen-2-yl)hex-4-enoate (4)

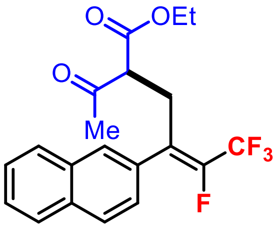

Prepared according to general procedures VII using styrene **1a** (54.4 mg, 0.2 mmol) and Ethyl acetoacetate (41.6 mg, 0.32 mmol).

The product was purified by flash column chromatography on silica gel (ethyl acetate: hexane = 1: 60) and obtained as a yellow oil (39.7 mg, 52% yield, *Z/E* > 99:1, *Z* isomer), R_f = 0.43 (ethyl acetate: hexane = 1: 10). **^{1}H NMR** (500 MHz, $CDCl_3$): δ (ppm) 7.93 – 7.83 (m, 3H), 7.74 (d, J = 1.7 Hz, 1H), 7.57 – 7.50 (m, 2H), 7.35 (d, J = 8.5 Hz, 1H), 4.10 – 3.94 (m, 2H), 3.31 (t, J = 7.2 Hz, 1H), 3.29 – 3.22 (m, 2H), 2.08 (s, 3H), 1.17 (t, J = 7.1 Hz, 3H). **^{13}C NMR** (126 MHz, $CDCl_3$): δ (ppm) 201.0, 168.6, 142.9 (dq, J = 254.5, 38.0 Hz), 133.3, 133.1, 130.5 (d, J = 1.8 Hz), 128.7, 128.3, 128.00, 127.97, 127.9, 127.1, 126.9, 125.6 (d, J = 2.8 Hz), 119.7 (qd, J = 273.7, 42.5 Hz), 61.9, 57.6 (d, J = 3.4 Hz), 29.0, 27.9 – 27.7 (m), 13.9. **^{19}F NMR** (377 MHz, $CDCl_3$): δ (ppm) -66.03 (d, J = 8.5 Hz, 3F), -126.68 (q, J = 8.5 Hz, 1F). **HRMS** m/z (ESI): calcd. for $C_{20}H_{17}F_4O_3$ $[M-H]^-$: 381.1119; found: 381.1119.

(Z)-4-(3,4,4,4-tetrafluoro-1-(naphthalen-2-yl)but-2-en-1-yl)morpholine (6a)

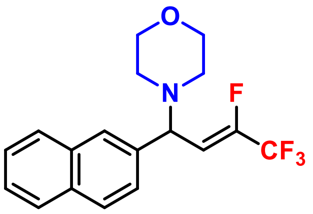

Prepared according to general procedures VIII using styrene **5a** (54.4 mg, 0.2 mmol) and morpholine (20.9 mg, 0.24 mmol). The product was purified by flash column chromatography on silica gel (ethyl acetate: hexane = 1: 10) and obtained as a yellow oil (56.9 mg, 84% yield, *Z/E* > 99:1, *Z* isomer), R_f = 0.38 (ethyl acetate: hexane = 1: 5). **^{1}H NMR** (400 MHz, $CDCl_3$): δ (ppm) 7.93 – 7.72 (m, 4H), 7.61 – 7.43 (m, 3H), 5.91 (dd, J = 32.5, 10.1 Hz, 1H), 4.44 (d, J = 10.1 Hz, 1H), 3.80 – 3.63 (m, 4H), 2.57 – 2.36 (m, 4H). **^{13}C NMR** (126 MHz, $CDCl_3$): δ (ppm) 146.8 (dq, J = 260.7, 39.4 Hz), 136.4, 133.5, 133.3, 129.1, 128.0, 127.9, 127.3, 126.6, 126.5, 125.3, 118.3 (qd, J = 271.9, 41.9 Hz), 114.0, 67.1, 64.4, 51.8. **^{19}F NMR** (471 MHz, $CDCl_3$): δ (ppm) -73.44 (d, J = 11.3 Hz, 3F), -133.80 – -134.60 (m, 1F). **HRMS** m/z (ESI): calcd. for $C_{18}H_{18}F_4NO$ $[M+H]^+$: 340.1319; found: 340.1314.

(Z)-1-(3,4,4,4-tetrafluoro-1-(naphthalen-2-yl)but-2-en-1-yl)pyrrolidine (6b)

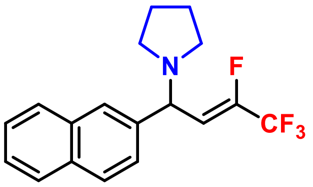

Prepared according to general procedures VIII using styrene **5a** (54.4 mg, 0.2 mmol) and tetrahydropyrrolidine (17.2 mg, 0.24 mmol). The product was purified by flash column chromatography on silica gel (ethyl acetate: hexane = 1: 10) and obtained as a yellow oil (40.1 mg, 62% yield, *Z/E* > 99:1, *Z* isomer), R_f = 0.39 (ethyl acetate: hexane = 1: 5). **^{1}H NMR** (500 MHz, $CDCl_3$): δ (ppm) 7.89 – 7.72 (m, 4H), 7.55 (dd, J = 8.6, 1.7 Hz, 1H), 7.49 (qd, J = 7.2, 6.8, 3.4 Hz, 2H), 5.97 (dd, J = 32.7, 10.0 Hz, 1H), 4.39 (d, J = 10.0 Hz, 1H), 2.71 – 2.35 (m, 4H), 1.90 – 1.70 (m, 4H). **^{13}C NMR** (126 MHz, $CDCl_3$): δ (ppm) 145.5 (dq, J = 259.2, 39.3 Hz), 138.2, 133.6, 133.3, 128.9, 128.0, 127.8, 126.7, 126.4, 126.3, 125.4, 118.5 (qd, J = 271.7, 41.8 Hz), 115.7, 63.9, 52.9, 23.5. **^{19}F NMR** (471 MHz, $CDCl_3$): δ (ppm) -73.55 (d, J = 11.5 Hz, 3F), -134.72 – -135.65 (m, 1F). **HRMS** m/z (ESI): calcd. for $C_{18}H_{18}F_4N$ $[M+H]^+$: 324.1370; found: 324.1366.

(Z)-N-benzyl-3,4,4,4-tetrafluoro-N-methyl-1-(naphthalen-2-yl)but-2-en-1-amine (6c)

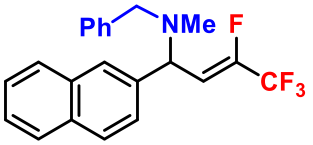

Prepared according to general procedures VIII using styrene **5a** (54.4 mg, 0.2 mmol) and N-Methylbenzylamine (29.1 mg, 0.24 mmol). The product was purified by flash column chromatography on silica gel (ethyl acetate: hexane = 1: 10) and obtained as a yellow oil (61.9 mg, 83% yield, *Z/E* > 99:1, *Z* isomer), R_f = 0.41 (ethyl acetate: hexane = 1: 5). **^{1}H NMR** (500 MHz, $CDCl_3$): δ (ppm) 7.95 – 7.83 (m, 4H), 7.75 – 7.68 (m, 1H), 7.57 – 7.48 (m, 2H), 7.42 – 7.33 (m, 4H), 7.32 – 7.26 (m, 1H), 6.07 (dd, J = 32.9,

10.1 Hz, 1H), 4.78 (d, J = 10.1 Hz, 1H), 3.70 – 3.49 (m, 2H), 2.23 (s, 3H). **^{13}C NMR** (126 MHz, $CDCl_3$): δ (ppm) 146.8 (dq, J = 259.6, 39.2 Hz), 138.9, 137.6, 133.5, 133.3, 129.0, 128.9, 128.5, 128.1, 127.9, 127.3, 126.8, 126.5, 126.4, 125.6, 118.4 (qd, J = 271.9, 42.2 Hz), 113.2, 62.6, 59.0, 39.2. **^{19}F NMR** (471 MHz, $CDCl_3$): δ (ppm) -73.3 (d, J = 11.4 Hz, 3F), -133.9 (s, 1F). **HRMS** m/z (ESI): calcd. for $C_{22}H_{20}F_4N$ $[M+H]^+$: 374.1526; found: 374.1521.

(Z)-N-benzyl-3,4,4,4-tetrafluoro-1-(naphthalen-2-yl)but-2-en-1-amine (6d)

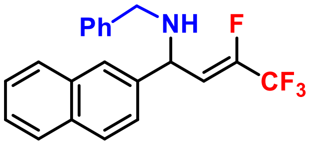

Prepared according to general procedures VIII using styrene **5a** (54.4 mg, 0.2 mmol) and Benzylamine (25.8 mg, 0.24 mmol). The product was purified by flash column chromatography on silica gel (ethyl acetate: hexane = 1: 10) and obtained as a yellow oil (61.0 mg, 85% yield, Z/E > 99:1, Z isomer), R_f = 0.42 (ethyl acetate: hexane = 1: 5). **^{1}H NMR** (400 MHz, $CDCl_3$): δ (ppm) 7.92 – 7.83 (m, 4H), 7.60 – 7.47 (m, 3H), 7.42 – 7.35 (m, 4H), 7.34 – 7.28 (m, 1H), 5.88 (dd, J = 32.9, 9.5 Hz, 1H), 5.00 (d, J = 9.5 Hz, 1H), 3.84 (d, J = 13.2 Hz, 1H), 3.79 (d, J = 13.2 Hz, 1H), 2.15 (bs, 1H). **^{13}C NMR** (126 MHz, $CDCl_3$): δ (ppm) 150.01, 142.23, 140.44, 134.40, 129.95, 129.06, 128.99, 128.87, 128.12, 127.83, 127.73, 127.29, 127.11, 121.43 (t, $J_{C\text{-}F}$ = 31.1 Hz), 120.10 (qt, $J_{C\text{-}F}$ = 288.9, 38.7 Hz), 111.05 (tq, $J_{C\text{-}F}$ = 257.04, 41.3 Hz), 54.86. **^{19}F NMR** (471 MHz, $CDCl_3$): δ (ppm) -84.98 (t, J = 3.7 Hz, 3F), -109.00 (d, J = 4.1 Hz, 2F). **HRMS** m/z (ESI): calcd. for $C_{21}H_{18}F_4N$ $[M+H]^+$: 360.1370; found: 360.1366.

(Z)-3,4,4,4-tetrafluoro-N-(4-methoxybenzyl)-1-(naphthalen-2-yl)but-2-en-1-amine (6e)

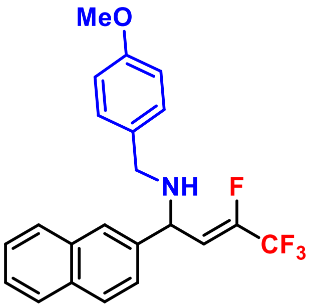

Prepared according to general procedures VIII using styrene **5a** (54.4 mg, 0.2 mmol) and 4-Methoxybenzylamine (32.9 mg, 0.24 mmol). The product was purified by flash column chromatography on silica gel (ethyl acetate: hexane = 1: 10) and obtained as a yellow oil (54.6 mg, 70% yield, Z/E > 99:1, Z isomer), R_f = 0.40 (ethyl acetate: hexane = 1: 5). **^{1}H NMR** (500 MHz, $CDCl_3$): δ (ppm) 7.90 – 7.83 (m, 4H), 7.59 – 7.46 (m, 3H), 7.33 – 7.20 (m, 2H), 6.90 (d, J = 8.1 Hz, 2H), 5.87 (dd, J = 33.0, 9.5 Hz, 1H), 4.97 (d, J = 9.6 Hz, 1H), 3.82 (s, 3H), 3.79 – 3.61 (m, 2H), 2.02 (bs, 1H). **^{13}C NMR** (101 MHz, $CDCl_3$): δ (ppm) 159.1, 146.1 (dd, J = 260.1, 39.3 Hz), 137.7, 133.6, 133.3, 131.3, 129.7, 129.1, 128.1, 127.9, 126.6, 126.4, 126.2, 124.9, 118.4 (dd, J = 271.8, 41.8 Hz), 115.5, 114.1, 55.7 (d, J = 2.4 Hz), 55.4, 50.9. **^{19}F NMR** (471 MHz, $CDCl_3$): δ (ppm) -73.47 (d, J = 11.6 Hz, 3F), -135.08 (s, 1F). **HRMS** m/z (ESI): calcd. for $C_{22}H_{20}F_4NO$ $[M+H]^+$: 390.1476; found: 390.1471.

(Z)-3,4,4,4-tetrafluoro-N-(3-methoxybenzyl)-1-(naphthalen-2-yl)but-2-en-1-amine (6f)

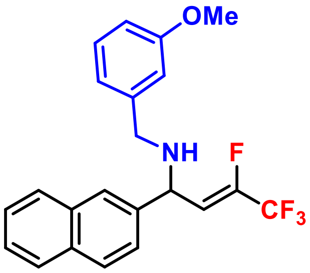

Prepared according to general procedures VIII using styrene **5a** (54.4 mg, 0.2 mmol) and 3-Methoxybenzylamine (32.9 mg, 0.24 mmol). The product was purified by flash column chromatography on silica gel (ethyl acetate: hexane = 1: 10) and obtained as a yellow oil (62.9 mg, 81% yield, Z/E > 99:1, Z isomer), R_f = 0.38 (ethyl acetate: hexane = 1: 5). **^{1}H NMR** (500 MHz, $CDCl_3$): δ (ppm) 7.87 – 7.79 (m, 4H), 7.55 – 7.44 (m, 3H), 7.26 – 7.21 (m, 1H), 6.90 (d, J = 7.0 Hz, 2H), 6.84 – 6.78 (m, 1H), 5.86 (dd, J =

32.8, 9.5 Hz, 1H), 4.95 (d, J = 9.5 Hz, 1H), 3.79 (s, 3H), 3.75 (d, J = 5.4 Hz, 2H), 1.27 (bs, 1H). ^{13}**C NMR** (126 MHz, $CDCl_3$): δ (ppm) 160.0, 146.0 (dq, J = 221.2, 39.9 Hz), 133.5, 133.3, 129.7, 129.1, 128.1, 127.9, 126.6, 126.4, 126.3 (2C), 124.9, 120.7, 118.4 (qd, J = 271.8, 41.8 Hz), 115.4, 113.9, 113.1 (2C), 55.8, 55.4, 51.4. ^{19}**F NMR** (471 MHz, $CDCl_3$): δ (ppm) -73.47 (d, J = 11.4 Hz, 3F), -134.80 (s, 1F). **HRMS** m/z (ESI): calcd. for $C_{22}H_{20}F_4NO$ $[M+H]^+$: 390.1476; found: 390.1472.

(Z)-3,4,4,4-tetrafluoro-1-(naphthalen-2-yl)-N-phenethylbut-2-en-1-amine (6g)

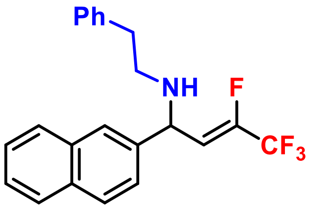

Prepared according to general procedures IX using styrene **5a** (54.4 mg, 0.2 mmol) and Phenethylamine (58.2 mg, 0.48 mmol). The product was purified by flash column chromatography on silica gel (ethyl acetate: hexane = 1: 10) and obtained as a yellow oil (48.6 mg, 65% yield, Z/E > 99:1, Z isomer), R_f = 0.39 (ethyl acetate: hexane = 1: 5). ^{1}**H NMR** (400 MHz, $CDCl_3$): δ (ppm) 7.88 – 7.77 (m, 4H), 7.55 – 7.43 (m, 3H), 7.36 – 7.16 (m, 5H), 5.82 (dd, J = 32.8, 9.6 Hz, 1H), 4.96 (d, J = 9.5 Hz, 1H), 3.00 – 2.81 (m, 4H), 2.03 (bs, 1H). ^{13}**C NMR** (101 MHz, $CDCl_3$): δ (ppm) 146.1 (dq, J = 260.0, 39.2 Hz), 139.5, 137.7, 133.5, 133.3, 129.0, 128.9, 128.7, 128.1, 127.8, 126.6, 126.5, 126.4, 126.1, 124.8, 118.4 (qd, J = 271.8, 41.8 Hz), 115.4, 56.5 (d, J = 2.5 Hz), 48.7, 36.1. ^{19}**F NMR** (471 MHz, $CDCl_3$): δ (ppm) -73.49 (d, J = 11.3 Hz, 3F), -135.34(s, 1F). **HRMS** m/z (ESI): calcd. for $C_{22}H_{20}F_4N$ $[M+H]^+$: 374.1526; found: 374.1521.

(Z)-N-butyl-3,4,4,4-tetrafluoro-1-(naphthalen-2-yl)but-2-en-1-amine (6h)

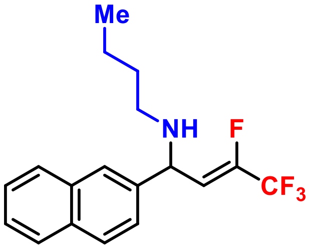

Prepared according to general procedures IX using styrene **5a** (54.4 mg, 0.2 mmol) and Butylamine (35.2 mg, 0.48 mmol). The product was purified by flash column chromatography on silica gel (ethyl acetate: hexane = 1: 10) and obtained as a yellow oil (44.8 mg, 69% yield, Z/E > 99:1, Z isomer), R_f = 0.42 (ethyl acetate: hexane = 1: 5). ^{1}**H NMR** (500 MHz, $CDCl_3$): δ (ppm) 7.89 – 7.81 (m, 4H), 7.54 – 7.46 (m, 3H), 5.83 (dd, J = 33.0, 9.6 Hz, 1H), 4.93 (d, J = 9.5 Hz, 1H), 2.63 (ddt, J = 31.6, 11.4, 7.2 Hz, 2H), 1.53 (p, J = 7.2 Hz, 2H), 1.44 – 1.32 (m, 3H), 0.93 (t, J = 7.3 Hz, 3H). ^{13}**C NMR** (126 MHz, $CDCl_3$): δ (ppm) 145.9 (dq, J = 258.9, 38.9 Hz), 138.4, 133.6, 133.2, 128.9, 128.0, 127.8, 126.5, 126.3, 125.9, 124.9, 118.5 (qd, J = 271.6, 41.9 Hz), 116.1 (dq, J = 6.4, 3.1 Hz), 56.8, 47.6, 32.3, 20.5, 14.1. ^{19}**F NMR** (471 MHz, $CDCl_3$): δ (ppm) -73.49 (d, J = 11.7 Hz, 3F), -135.80 (dq, J = 33.8, 11.5 Hz, 1F). **HRMS** m/z (ESI): calcd. for $C_{18}H_{20}F_4N$ $[M+H]^+$: 326.1526; found: 326.1522.

(Z)-3,4,4,4-tetrafluoro-N-isopropyl-1-(naphthalen-2-yl)but-2-en-1-amine (6i)

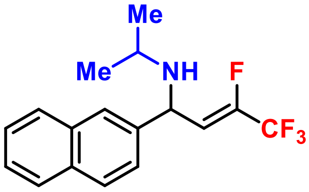

Prepared according to general procedures IX using styrene **5a** (54.4 mg, 0.2 mmol) and Isopropylamine (28.4 mg, 0.48 mmol). The product was purified by flash column chromatography on silica gel (ethyl acetate: hexane = 1: 10) and obtained as a yellow oil (35.6 mg, 57% yield, Z/E > 99:1, Z isomer), R_f = 0.45 (ethyl acetate: hexane = 1: 5). ^{1}**H NMR** (500 MHz, $CDCl_3$): δ (ppm) 7.88 – 7.81

(m, 4H), 7.54 – 7.45 (m, 3H), 5.81 (dd, J = 33.0, 9.6 Hz, 1H), 5.06 (d, J = 9.6 Hz, 1H), 2.84 (hept, J = 6.2 Hz, 1H), 1.78 (bs, 1H), 1.13 (dd, J = 15.9, 6.3 Hz, 6H). ^{13}**C NMR** (126 MHz, $CDCl_3$): δ (ppm) 145.7 (dq, J = 258.0, 39.5, 38.9 Hz), 138.4, 133.6, 133.2, 129.0, 128.0, 127.8, 126.5, 126.3, 125.9, 124.9, 118.5 (qd, J = 271.6, 42.2 Hz), 116.1, 53.8 (d, J = 2.5 Hz), 46.4, 23.1, 23.0. ^{19}**F NMR** (471 MHz, $CDCl_3$): δ (ppm) -73.46 (d, J = 11.3 Hz, 3F), -135.97(s, 1F). **HRMS** m/z (ESI): calcd. for $C_{17}H_{18}F_4N$ $[M+H]^+$: 312.1370; found: 312.1364.

(Z)-N-(4-(3,4,4,4-tetrafluoro-1-morpholinobut-2-en-1-yl)phenyl)benzamide (6j)

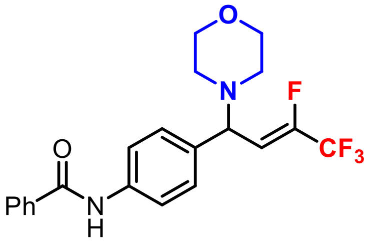

Prepared according to general procedures VIII using styrene **5j** (68.2 mg, 0.2 mmol) and morpholine (20.9 mg, 0.24 mmol). The product was purified by flash column chromatography on silica gel (ethyl acetate: hexane = 1: 10) and obtained as a yellow oil (54.6 mg, 67% yield, *Z/E* > 99:1, *Z* isomer), R_f = 0.24 (ethyl acetate: hexane = 1: 5). ^{1}**H NMR** (500 MHz, $CDCl_3$): δ (ppm) 8.22 – 8.08 (m, 1H), 7.84 (d, J = 7.7 Hz, 2H), 7.64 (d, J = 8.3 Hz, 2H), 7.57 – 7.49 (m, 1H), 7.49 – 7.41 (m, 2H), 7.33 (d, J = 8.2 Hz, 2H), 5.77 (dd, J = 32.5, 10.1 Hz, 1H), 4.24 (d, J = 10.1 Hz, 1H), 3.69 (t, J = 4.7 Hz, 4H), 2.56 – 2.25 (m, 4H). ^{13}**C NMR** (126 MHz, $CDCl_3$): δ (ppm) 166.1, 146.6 (dq, J = 259.9, 39.4 Hz), 138.0, 135.0, 134.9, 132.1, 128.9, 128.7, 127.2, 120.9, 118.3 (qd, J = 271.7, 41.7 Hz), 114.0, 67.0, 63.7, 51.6. ^{19}**F NMR** (471 MHz, $CDCl_3$): δ (ppm) -73.43 (d, J = 11.3 Hz, 3F), -134.21 – -134.87 (m, 1F). **HRMS** m/z (ESI): calcd. for $C_{21}H_{20}F_4N_2O_2Na$ $[M+Na]^+$: 431.1353; found: 431.1347.

(Z)-4-(1-(4-(tert-butyl)phenyl)-3,4,4,4-tetrafluorobut-2-en-1-yl)morpholine (6k)

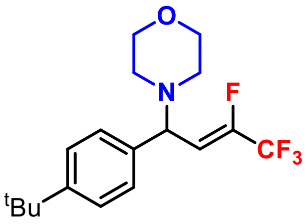

Prepared according to general procedures VIII using styrene **5k** (55.6 mg, 0.2 mmol) and morpholine (20.9 mg, 0.24 mmol). The product was purified by flash column chromatography on silica gel (ethyl acetate: hexane = 1: 10) and obtained as a yellow oil (44.2 mg, 64% yield, *Z/E* > 99:1, *Z* isomer), R_f = 0.40 (ethyl acetate: hexane = 1: 5). ^{1}**H NMR** (500 MHz, $CDCl_3$): δ (ppm) 7.37 (d, J = 8.1 Hz, 2H), 7.29 – 7.24 (m, 2H), 5.78 (dd, J = 32.6, 10.2 Hz, 1H), 4.23 (d, J = 10.2 Hz, 1H), 3.70 (t, J = 4.5 Hz, 4H), 2.53 – 2.28 (m, 4H), 1.32 (s, 9H). ^{13}**C NMR** (101 MHz, $CDCl_3$): δ (ppm) 151.3, 146.5 (dq, J = 259.4, 39.1 Hz), 135.7, 127.7, 126.0, 118.4 (qd, J = 271.7, 42.0 Hz), 114.2 (dq, J = 6.6, 3.4 Hz), 67.1, 63.8, 51.7, 34.7, 31.5. ^{19}**F NMR** (471 MHz, $CDCl_3$): δ (ppm) -73.42 (d, J = 11.4 Hz, 3F), -135.05 (dq, J = 33.4, 11.6 Hz, 1F). **HRMS** m/z (ESI): calcd. for $C_{18}H_{24}F_4NO$ $[M+H]^+$: 346.1789; found: 346.1786.

(Z)-4-(1-(4-chlorophenyl)-3,4,4,4-tetrafluorobut-2-en-1-yl)morpholine (6l)

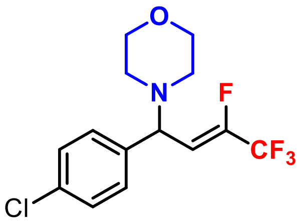

Prepared according to general procedures VIII using styrene **5l** (51.2 mg, 0.2 mmol) and morpholine (20.9 mg, 0.24 mmol). The product was purified by flash column chromatography on silica gel (ethyl acetate: hexane = 1: 10) and obtained as a yellow oil (58.1 mg, 90% yield, *Z/E* > 99:1, *Z* isomer), R_f = 0.38 (ethyl acetate: hexane = 1: 5). ^{1}**H NMR** (500 MHz, $CDCl_3$): δ (ppm) 7.37 – 7.27 (m, 4H), 5.73 (dd, J = 32.3, 10.1 Hz, 1H), 4.25 (d, J = 10.0 Hz, 1H), 3.69 (s, 4H), 2.51 – 2.21 (m, 4H). ^{13}**C NMR** (126 MHz, $CDCl_3$): δ (ppm) 146.9 (dq, J = 260.3, 39.4 Hz), 137.6, 134.1, 129.32, 129.28, 118.2 (qd, J = 271.6, 42.0 Hz), 113.6, 67.0, 63.4, 51.6. ^{19}**F**

NMR (471 MHz, $CDCl_3$): δ (ppm) -73.50 (d, J = 11.3 Hz, 3F), -133.89 (dq, J = 33.1, 11.1 Hz, 1F). **HRMS** m/z (ESI): calcd. for $C_{14}H_{15}ClF_4NO$ $[M+H]^+$: 324.0773; found: 324.0771.

(Z)-4-(1-(2-chlorophenyl)-3,4,4,4-tetrafluorobut-2-en-1-yl)morpholine (6m)

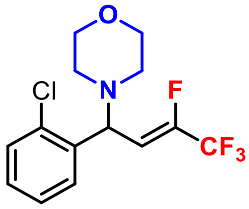

Prepared according to general procedures VIII using styrene **5m** (51.2 mg, 0.2 mmol) and morpholine (20.9 mg, 0.24 mmol). The product was purified by flash column chromatography on silica gel (ethyl acetate: hexane = 1: 10) and obtained as a yellow oil (50.4 mg, 78% yield, Z/E > 99:1, Z isomer), R_f = 0.37 (ethyl acetate: hexane = 1: 5). **^{1}H NMR** (500 MHz, $CDCl_3$): δ (ppm) 7.55 (d, J = 7.8 Hz, 1H), 7.38 (d, J = 7.9 Hz, 1H), 7.33 – 7.27 (m, 1H), 7.27 – 7.19 (m, 1H), 5.64 (dd, J = 32.1, 10.1 Hz, 1H), 4.82 (d, J = 10.1 Hz, 1H), 3.75 – 3.63 (m, 4H), 2.56 – 2.34 (m, 4H). **^{13}C NMR** (126 MHz, $CDCl_3$): δ (ppm) 147.1 (dd, J = 262.0, 39.8 Hz), 136.5, 134.4, 130.3, 129.3, 129.2, 127.6, 118.3 (dd, J = 272.0, 41.5 Hz), 114.1 – 111.7 (m), 67.0, 59.9, 51.7. **^{19}F NMR** (471 MHz, $CDCl_3$): δ (ppm) -73.50 (d, J = 11.3 Hz, 3F), -131.98 (dq, J = 33.1, 11.3 Hz, 1F). **HRMS** m/z (ESI): calcd. for $C_{14}H_{15}ClF_4NO$ $[M+H]^+$: 324.0773; found: 324.0770.

(Z)-4-(1-(benzyloxy)-5,6,6,6-tetrafluorohex-4-en-3-yl)morpholine (6n)

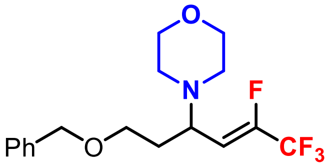

Prepared according to general procedures VIII using styrene **5n** (56.0 mg, 0.2 mmol) and morpholine (20.9 mg, 0.24 mmol). The product was purified by flash column chromatography on silica gel (ethyl acetate: hexane = 1: 10) and obtained as a yellow oil (59.6 mg, 86% yield, Z/E > 99:1, Z isomer), R_f = 0.39 (ethyl acetate: hexane = 1: 5). **^{1}H NMR** (400 MHz, $CDCl_3$): δ (ppm) 7.39 – 7.26 (m, 5H), 5.55 (dd, J = 33.9, 10.3 Hz, 1H), 4.49 (s, 2H), 3.80 – 3.62 (m, 5H), 3.61 – 3.52 (m, 1H), 3.50 – 3.40 (m, 1H), 2.61 – 2.37 (m, 4H), 2.12 – 1.95 (m, 1H), 1.74 (ddt, J = 14.0, 8.2, 6.0 Hz, 1H). **^{13}C NMR** (126 MHz, $CDCl_3$): δ (ppm) 147.3 (dq, J = 258.7, 38.9 Hz), 138.3, 128.5, 127.8, 127.7, 118.3 (qd, J = 271.9, 43.0 Hz), 111.4 – 110.9 (m), 73.3, 67.1, 66.6, 55.4, 49.5, 31.8. **^{19}F NMR** (471 MHz, $CDCl_3$): δ (ppm) -73.17 (d, J = 11.4 Hz, 3F), -132.71 (s, 1F). **HRMS** m/z (ESI): calcd. for $C_{17}H_{22}F_4NO_2$ $[M+H]^+$: 348.1581; found: 348.1577.

(Z)-4-(5,6,6,6-tetrafluoro-1-phenylhex-4-en-3-yl)morpholine (6o)

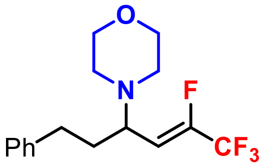

Prepared according to general procedures VIII using styrene **5o** (50.0 mg, 0.2 mmol) and morpholine (20.9 mg, 0.24 mmol). The product was purified by flash column chromatography on silica gel (ethyl acetate: hexane = 1: 10) and obtained as a yellow oil (52.1 mg, 82% yield, Z/E > 99:1, Z isomer), R_f = 0.43 (ethyl acetate: hexane = 1: 5). **^{1}H NMR** (500 MHz, $CDCl_3$): δ (ppm) 7.33 – 7.27 (m, 2H), 7.24 – 7.14 (m, 3H), 5.57 (dd, J = 34.1, 10.2 Hz, 1H), 3.78 – 3.63 (m, 4H), 3.49 (dt, J = 10.1, 7.2 Hz, 1H), 2.67 (t, J = 7.9 Hz, 2H), 2.56 (ddd, J = 10.0, 6.1, 3.1 Hz, 2H), 2.46 (ddd, J = 10.8, 6.1, 3.2 Hz, 2H), 2.03 (dq, J = 14.5, 7.4 Hz, 1H), 1.83 – 1.72 (m, 1H). **^{13}C NMR** (126 MHz, $CDCl_3$): δ (ppm) 147.3 (dq, J = 258.4, 39.0 Hz), 141.4, 128.6, 128.5, 126.2, 118.3 (qd, J = 271.9, 43.0 Hz), 111.4 (dq, J = 8.8, 2.8 Hz), 67.2, 57.6, 49.4, 33.5, 32.1. **^{19}F NMR** (471 MHz, $CDCl_3$): δ (ppm) -73.15 (d, J = 11.4 Hz, 3F), -132.55 (dq, J = 34.6, 11.6 Hz, 1F). **HRMS** m/z (ESI): calcd. for $C_{16}H_{20}F_4NO$ $[M+H]^+$: 318.1476; found: 318.1474.

(Z)-4-methyl-7-((7,10,10,10,10,10,10,10-octafluoro-5-morpholino-10l8-dec-6-en-8-yn-1-yl)oxy)-2H-chromen-2-one (6p)

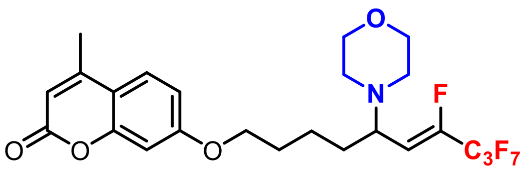

Prepared according to general procedures VIII using styrene **5p** (89.6 mg, 0.2 mmol) and morpholine (20.9 mg, 0.24 mmol). The product was purified by flash column chromatography on silica gel (ethyl acetate: hexane = 1: 10) and obtained as a yellow oil (60.9 mg, 56% yield, *Z/E* > 99:1, *Z* isomer), R_f = 0.34 (ethyl acetate: hexane = 1: 5). **^{1}H NMR** (500 MHz, $CDCl_3$): δ (ppm) 7.46 (d, *J* = 8.8 Hz, 1H), 6.84 – 6.72 (m, 2H), 6.10 (s, 1H), 5.69 – 5.43 (m, 1H), 3.99 (t, *J* = 6.4 Hz, 2H), 3.80 – 3.42 (m, 5H), 2.61 – 2.40 (m, 3H), 2.37 (s, 2H), 1.88 – 1.69 (m, 3H), 1.57 – 1.42 (m, 4H), 1.39 – 1.34 (m, 1H). **^{13}C NMR** (126 MHz, $CDCl_3$): δ (ppm) 162.1, 161.5, 155.3, 152.7, 147.1 (dt, *J* = 259.3, 29.1 Hz), 125.6, 117.6 (qt, *J* = 286.4, 33.2 Hz), 115.2 – 114.2 (m), 113.6, 112.7, 111.9, 110.7 – 105.5 (m), 101.3, 68.2, 67.2, 58.4, 49.6, 31.4, 28.8, 22.4, 18.7. **^{19}F NMR** (471 MHz, $CDCl_3$): δ (ppm) -81.78 (t, *J* = 8.8 Hz, 3F), -119.26 (ddq, *J* = 24.4, 17.4, 8.5 Hz, 2F), -128.35 (d, *J* = 8.4 Hz, 2F), -128.49 – -128.89 (m, 1F). **HRMS** m/z (ESI): calcd. for $C_{24}H_{26}F_8NO_4$ $[M+H]^+$: 544.1729; found: 544.1728.

2-(3-(benzyloxy)-3,4,4,4-tetrafluorobut-1-en-2-yl)naphthalene (8a)

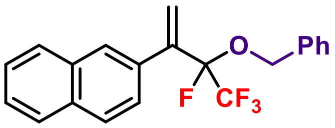

Prepared according to general procedures X using styrene **7** (100.6 mg, 0.2 mmol) and Benzyl alcohol (28.1 mg, 0.26 mmol). The product was purified by flash column chromatography on silica gel (ethyl acetate: hexane = 1: 60) and obtained as a yellow oil (48.9 mg, 68% yield), R_f = 0.47 (ethyl acetate: hexane = 1: 10). **^{1}H NMR** (500 MHz, $CDCl_3$): δ (ppm) 7.96 (s, 1H), 7.88 – 7.82 (m, 3H), 7.61 – 7.49 (m, 3H), 7.43 – 7.32 (m, 5H), 6.09 (s, 1H), 5.99 (d, *J* = 4.1 Hz, 1H) , 4.92 (dd, *J* = 33.1, 11.4 Hz, 2H). **^{13}C NMR** (126 MHz, $CDCl_3$): δ (ppm) 140.8, 140.6, 135.9, 133.9 (d, *J* = 2.3 Hz), 133.2, 128.7, 128.5, 128.4, 127.92, 127.91, 127.8, 127.7, 126.7, 126.5, 126.1, 124.6 (d, *J* = 9.0 Hz), 120.7 (qd, *J* = 286.7, 36.3 Hz), 109.1 (dq, *J* = 236.4, 34.7 Hz), 66.9 (d, *J* = 3.9 Hz). **^{19}F NMR** (471 MHz, $CDCl_3$): δ (ppm) -82.52 (d, *J* = 3.8 Hz, 3F), -126.19 – -126.27 (m, 1F). **HRMS** m/z (ESI): calcd. for $C_{21}H_{16}F_4ONa$ $[M+Na]^+$: 383.1030; found: 383.1026.

2-(3-((4-bromobenzyl)oxy)-3,4,4,4-tetrafluorobut-1-en-2-yl)naphthalene (8b)

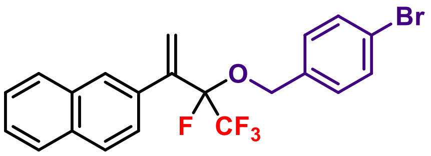

Prepared according to general procedures X using styrene **7** (100.6 mg, 0.2 mmol) and 4-Bromobenzyl alcohol (48.3 mg, 0.26 mmol). The product was purified by flash column chromatography on silica gel (ethyl acetate: hexane = 1: 60) and obtained as a yellow oil (77.8 mg, 89% yield), R_f = 0.48 (ethyl acetate: hexane = 1: 10). **^{1}H NMR** (500 MHz, $CDCl_3$): δ (ppm) 7.91 (s, 1H), 7.88 – 7.78 (m, 3H), 7.57 – 7.47 (m, 5H), 7.22 (d, *J* = 8.1 Hz, 2H), 6.05 (s, 1H), 5.98 (d, *J* = 3.9 Hz, 1H), 4.94 – 4.70 (m, 2H). **^{13}C NMR** (126 MHz, $CDCl_3$): δ (ppm) 140.7, 140.5, 134.9, 133.7 (d, *J* = 2.4 Hz), 133.1 (d, *J* = 2.6 Hz), 131.9, 129.5, 128.5, 128.0, 127.8, 127.7, 126.8, 126.6, 126.0, 124.8 (d, *J* = 9.1 Hz), 122.4, 120.6 (qd, *J* = 286.7, 36.1 Hz), 109.1 (dq, *J* = 236.5, 34.7 Hz), 66.2 (d, *J* = 4.0 Hz). **^{19}F NMR** (471 MHz, $CDCl_3$): δ (ppm) -82.70 (d, *J* = 3.9 Hz, 3F) , -126.09 – -126.23 (m, 1F). **HRMS** m/z (ESI): calcd. for $C_{21}H_{15}BrF_4ONa$ $[M+Na]^+$: 461.0135; found: 461.0134.

2-(3-(allyloxy)-3,4,4,4-tetrafluorobut-1-en-2-yl)naphthalene (8c)

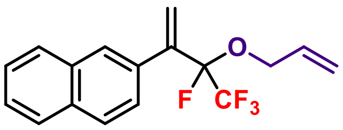

Prepared according to general procedures X using styrene **7** (100.6 mg, 0.2 mmol) and Allylic alcohol (15.1 mg, 0.26 mmol). The product was purified by flash column chromatography on silica gel (ethyl acetate: hexane = 1: 60) and obtained as a yellow oil (47.7 mg, 77% yield), R_f = 0.60 (ethyl acetate: hexane = 1: 10). **^{1}H NMR** (500 MHz, $CDCl_3$): δ (ppm) 7.90 (s, 1H), 7.87 – 7.80 (m, 3H), 7.56 – 7.45 (m, 3H), 6.01 (s, 1H), 5.96 (dt, J = 16.6, 5.7 Hz, 1H), 5.92 (d, J = 4.0 Hz, 1H), 5.41 (d, J = 17.2 Hz, 1H), 5.29 (d, J = 10.5 Hz, 1H), 4.38 (ddd, J = 24.0, 12.6, 5.4 Hz, 2H). **^{13}C NMR** (126 MHz, $CDCl_3$): δ (ppm) 140.7, 140.4, 133.9 (d, J = 2.4 Hz), 133.1 (d, J = 2.4 Hz), 132.5, 128.5, 127.9, 127.73, 127.68, 126.7, 126.5, 126.1 (d, J = 1.8 Hz), 124.5 (d, J = 8.9 Hz), 120.6 (qd, J = 286.7, 36.1 Hz), 118.2, 109.1 (dd, J = 236.0, 34.5 Hz), 66.0 (d, J = 3.5 Hz). **^{19}F NMR** (471 MHz, $CDCl_3$): δ (ppm) -82.68 (d, J = 4.1 Hz, 3F), -126.27 – -126.36 (m, 1F). **HRMS** m/z (ESI): calcd. for $C_{17}H_{14}F_4ONa$ $[M+Na]^+$: 333.0873; found: 333.0871.

2-(3,4,4,4-tetrafluoro-3-methoxybut-1-en-2-yl)naphthalene (8d)

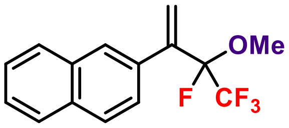

Prepared according to general procedures X using styrene **7** (100.6 mg, 0.2 mmol) and Methanol (8.3 mg, 0.26 mmol). The product was purified by flash column chromatography on silica gel (ethyl acetate: hexane = 1: 60) and obtained as a yellow oil (45.44 mg, 80% yield), R_f = 0.58 (ethyl acetate: hexane = 1: 10). **^{1}H NMR** (500 MHz, $CDCl_3$): δ (ppm) 7.93 – 7.75 (m, 4H), 7.55 – 7.48 (m, 3H), 5.99 (s, 1H), 5.93 (d, J = 4.1 Hz, 1H), 3.63 (s, 3H). **^{13}C NMR** (126 MHz, $CDCl_3$): δ (ppm) 140.2, 140.0, 133.9 (d, J = 2.6 Hz), 133.1 (d, J = 4.5 Hz), 128.5, 127.9, 127.7, 126.7, 126.5, 126.0, 124.6 (d, J = 9.4 Hz), 120.7 (qd, J = 286.7, 36.2 Hz), 109.2 (dq, J = 235.8, 34.5 Hz), 52.0 (d, J = 4.2 Hz). **^{19}F NMR** (471 MHz, $CDCl_3$): δ (ppm) -82.71 (d, J = 4.1 Hz, 3F), -129.35 – -129.42 (m, 1F). **HRMS** m/z (APCI): calcd. for $C_{15}H_{12}F_4O$ $[M]^+$: 284.0819; found: 284.0816.

benzyl(1,1,1,2-tetrafluoro-3-(naphthalen-2-yl)but-3-en-2-yl)sulfane (8e)

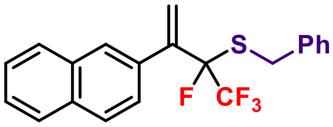

Prepared according to general procedures X using styrene **7** (100.6 mg, 0.2 mmol) and Benzylthiol (32.2 mg, 0.26 mmol). The product was purified by flash column chromatography on silica gel (ethyl acetate: hexane = 1: 60) and obtained as a yellow oil (64.7 mg, 86% yield), R_f = 0.43 (ethyl acetate: hexane = 1: 10). **^{1}H NMR** (500 MHz, $CDCl_3$): δ (ppm) 7.88 – 7.78 (m, 4H), 7.56 – 7.48 (m, 3H), 7.39 – 7.28 (m, 5H), 5.93 (s, 1H), 5.65 (d, J = 4.6 Hz, 1H), 4.15 (s, 2H). **^{13}C NMR** (126 MHz, $CDCl_3$): δ (ppm) 143.1, 142.9, 135.3, 134.4 (d, J = 2.6 Hz), 133.0 (d, J = 14.0 Hz), 129.5, 128.9, 128.5, 128.4, 127.9, 127.7, 127.5, 126.9 (d, J = 1.9 Hz), 126.6, 126.4, 122.9 (qd, J = 285.8, 35.6 Hz), 122.3 (d, J = 6.6 Hz), 102.7 (dq, J = 232.3, 33.2 Hz), 35.0 – 33.9 (m). **^{19}F NMR** (471 MHz, $CDCl_3$): δ (ppm) -75.6 (d, J = 11.1 Hz, 3F), -143.9 (qd, J = 11.8, 11.3, 4.5 Hz, 1F). **HRMS** m/z (ESI): calcd. for $C_{21}H_{16}F_4SNa$ $[M+Na]^+$: 399.0801; found: 399.0800.

IX. Reference

1. Fujihira, Y.; Hirano, K.; Ono, M.; Mimura, H.; Kagawa, T.; Sedgwick, D. M.; Fustero, S.; Shibata, N. Pentafluoroethylation of Carbonyl Compounds by HFC-125 via the Encapsulation of the K Cation with Glymes. *J. Org. Chem.* **2021**, *86*, 5883-5893.
2. Tang, Y.; Tsui, G. C. Copper-Catalyzed Pentafluoroethylation of Aryl/Alkenyl Iodides with Pentafluoroethylsilane. *Org. Chem. Front.* **2024**, *11*, 4366–4370.
3. (a) Tang, L.; Liu, Z. Y.; She, W.; Feng, C. Selective single C-F bond arylation of trifluoromethylalkene derivatives. *Chem. Sci.* **2019**, *10*, 8701-8705. (b) Ye, F.; Ge, Y.; Spannenberg, A.; Neumann, H.; Xu, L. W.; Beller, M. 3,3-Difluoroallyl ammonium salts: highly versatile, stable and selective gem-difluoroallylation reagents. *Nat. Commun.* **2021**, *12*, 3257.

X. Spectra of Substrates

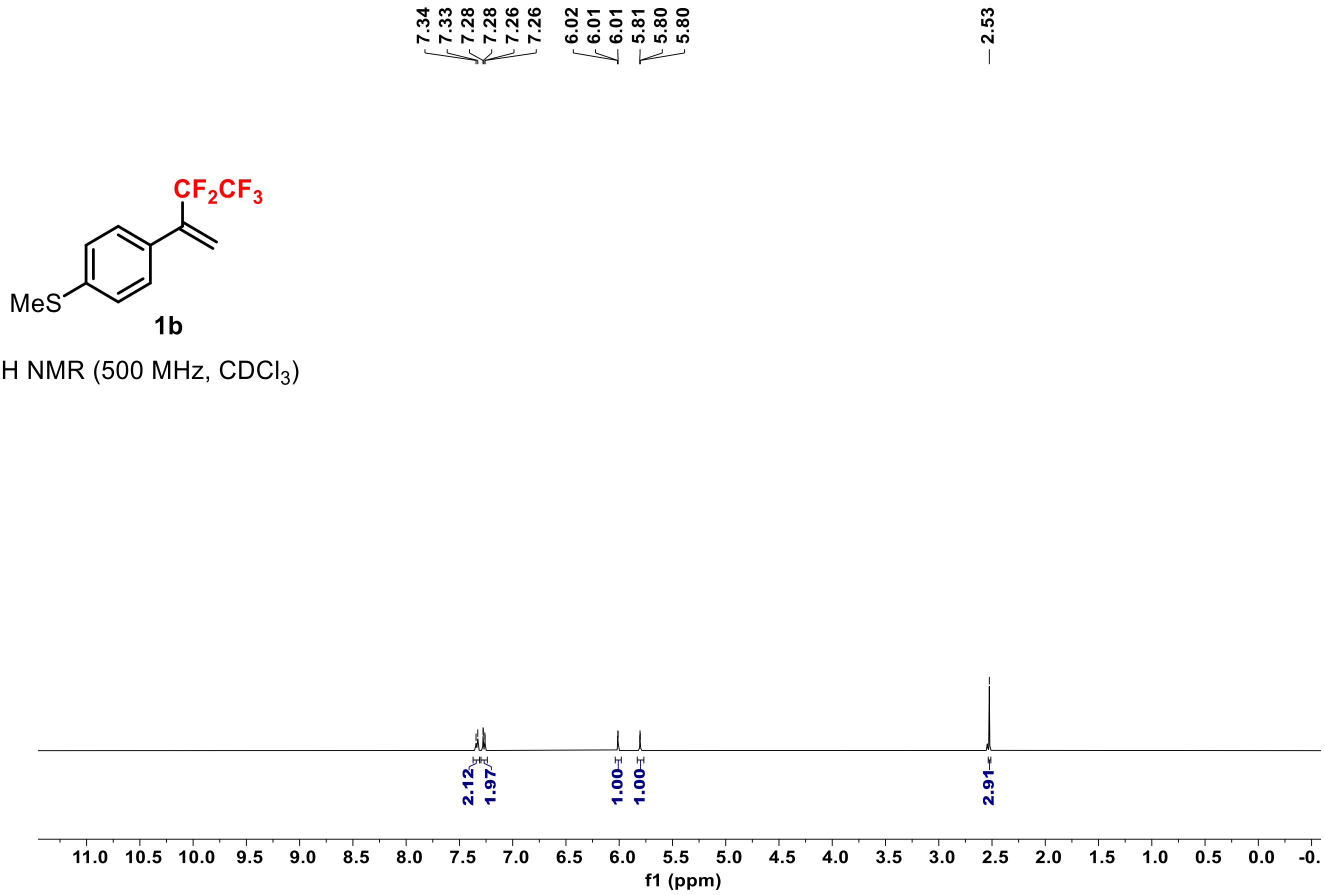

1b

^{1}H NMR (500 MHz, $CDCl_3$)

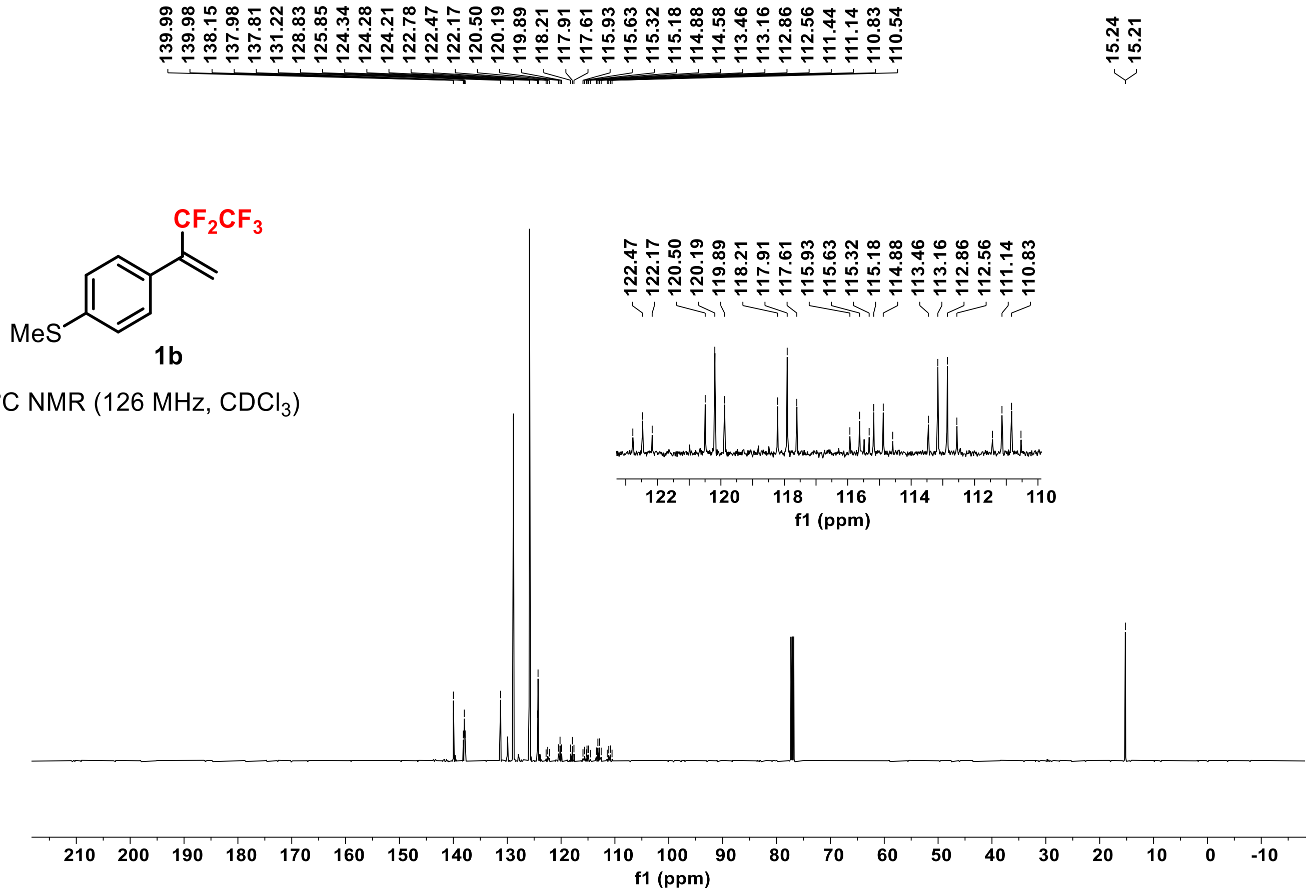

1b

^{13}C NMR (126 MHz, $CDCl_3$)

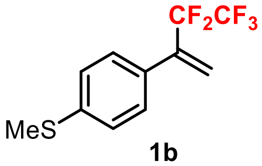

1b

^{19}F NMR (471 MHz, $CDCl_3$)

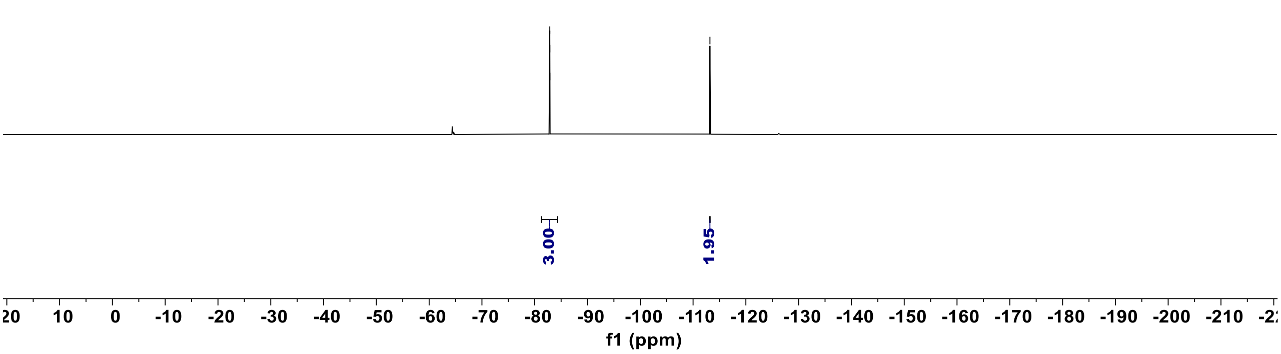

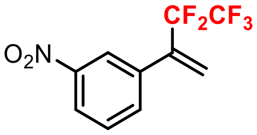

1c

^{1}H NMR (500 MHz, $CDCl_3$)

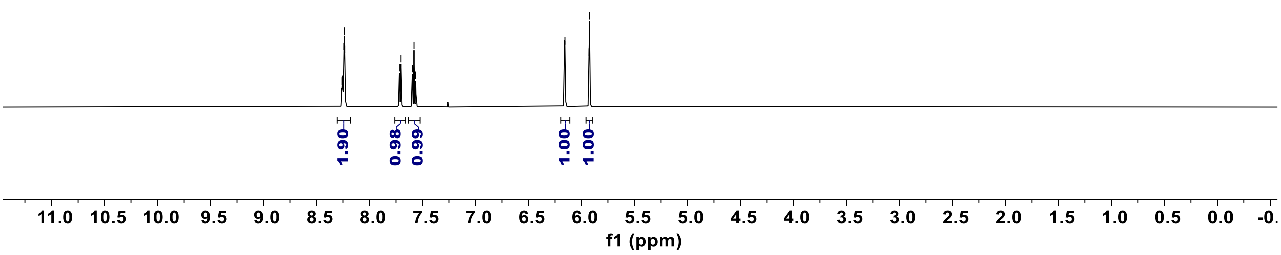

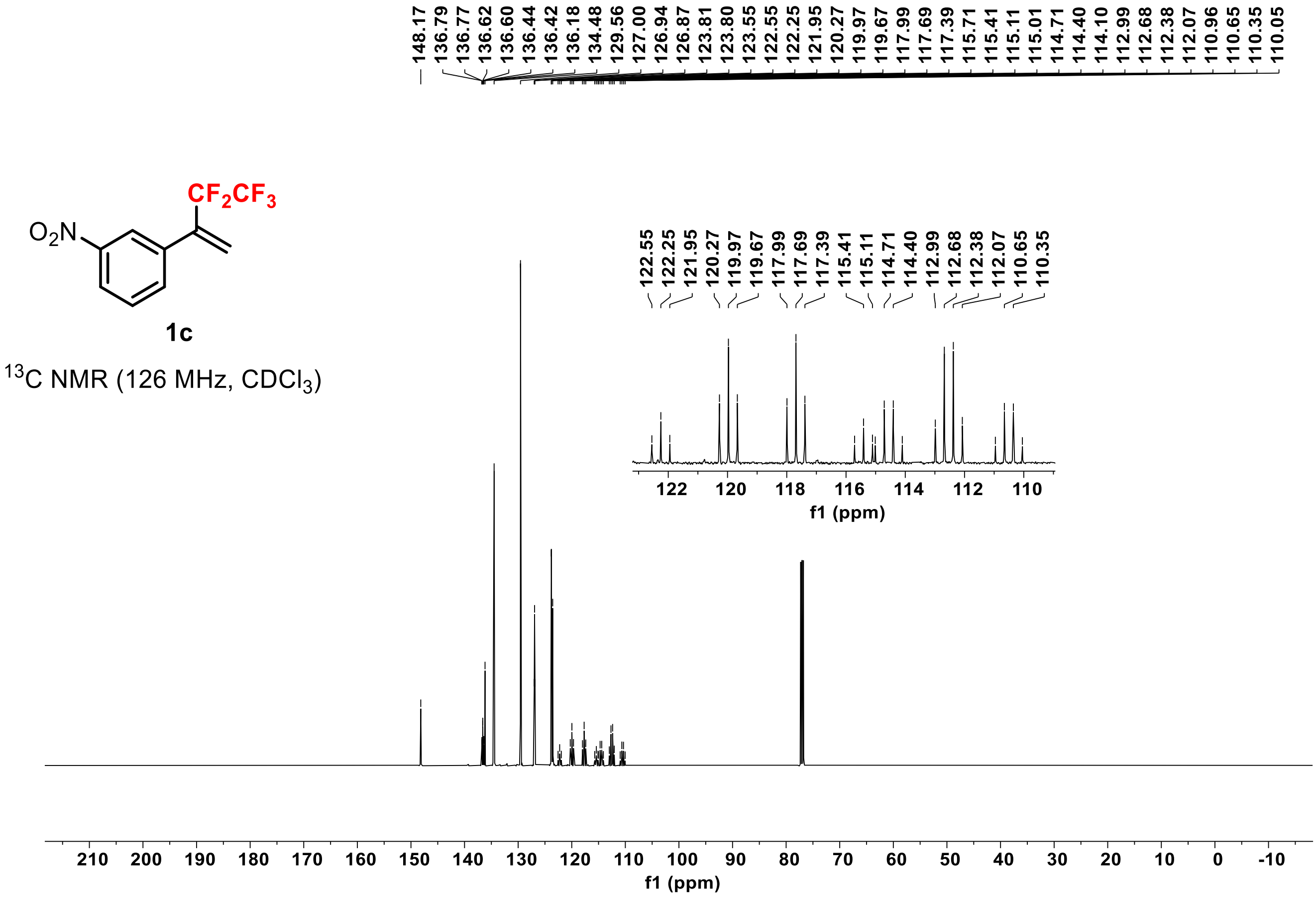

^{13}C NMR (126 MHz, $CDCl_3$)

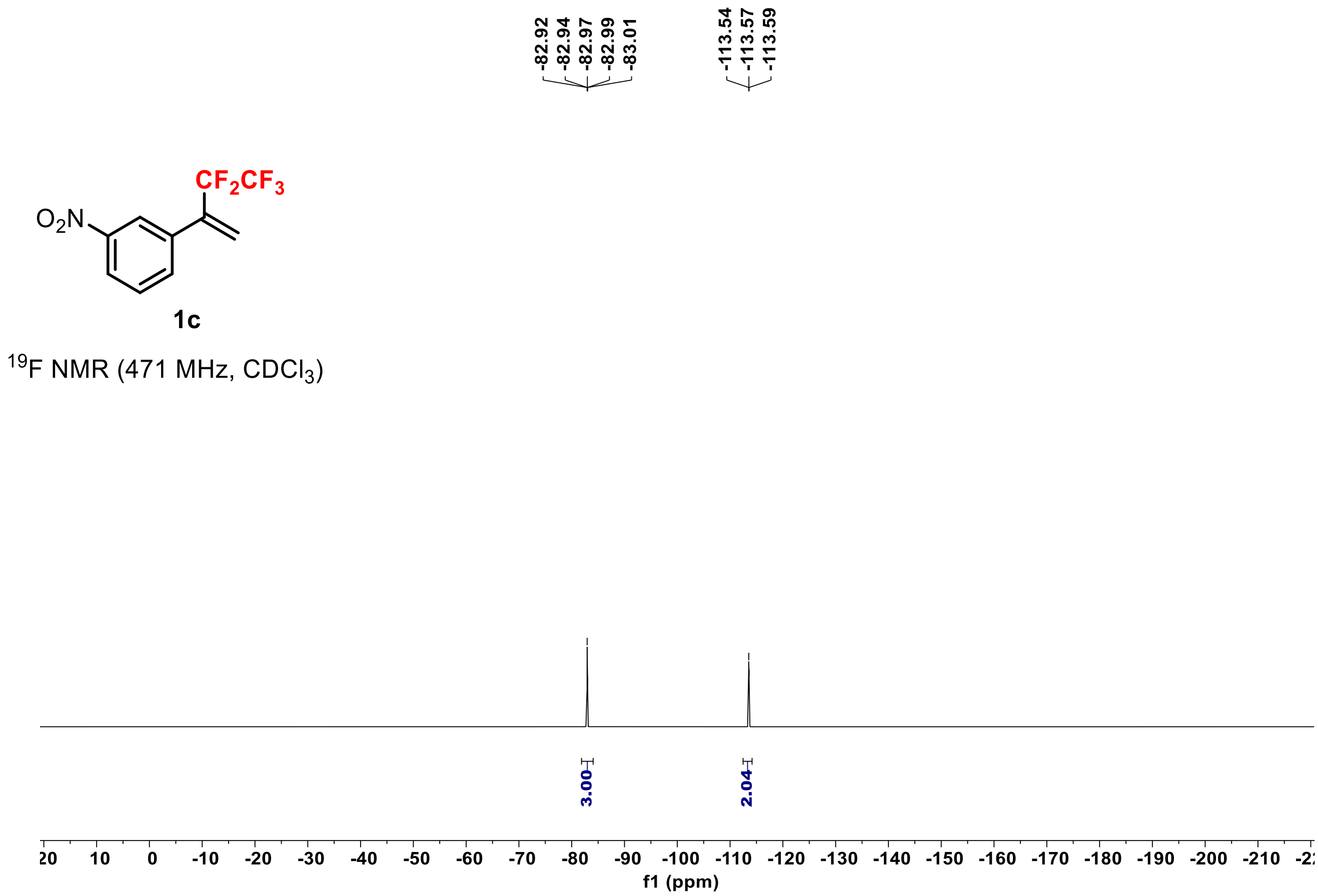

^{19}F NMR (471 MHz, $CDCl_3$)

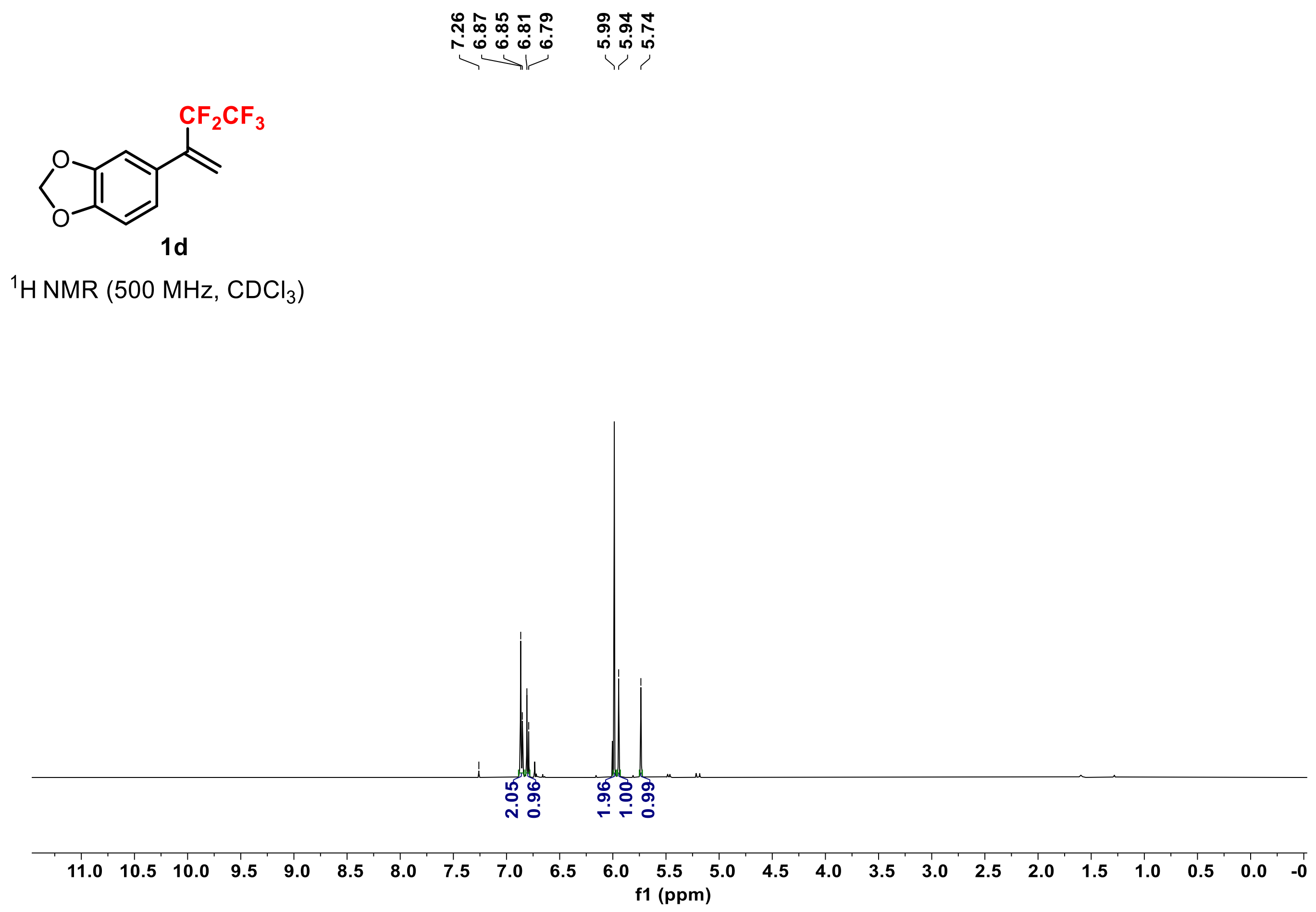
CF2CF3
1d
1H NMR (500 MHz, CDCl3)
7.26
6.87
6.85
6.81
6.79
5.99
5.94
5.74
2.05
0.96
1.96
1.00
0.99
11.0 10.5 10.0 9.5 9.0 8.5 8.0 7.5 7.0 6.5 6.0 5.5 5.0 4.5 4.0 3.5 3.0 2.5 2.0 1.5 1.0 0.5 0.0 -0
f1 (ppm)

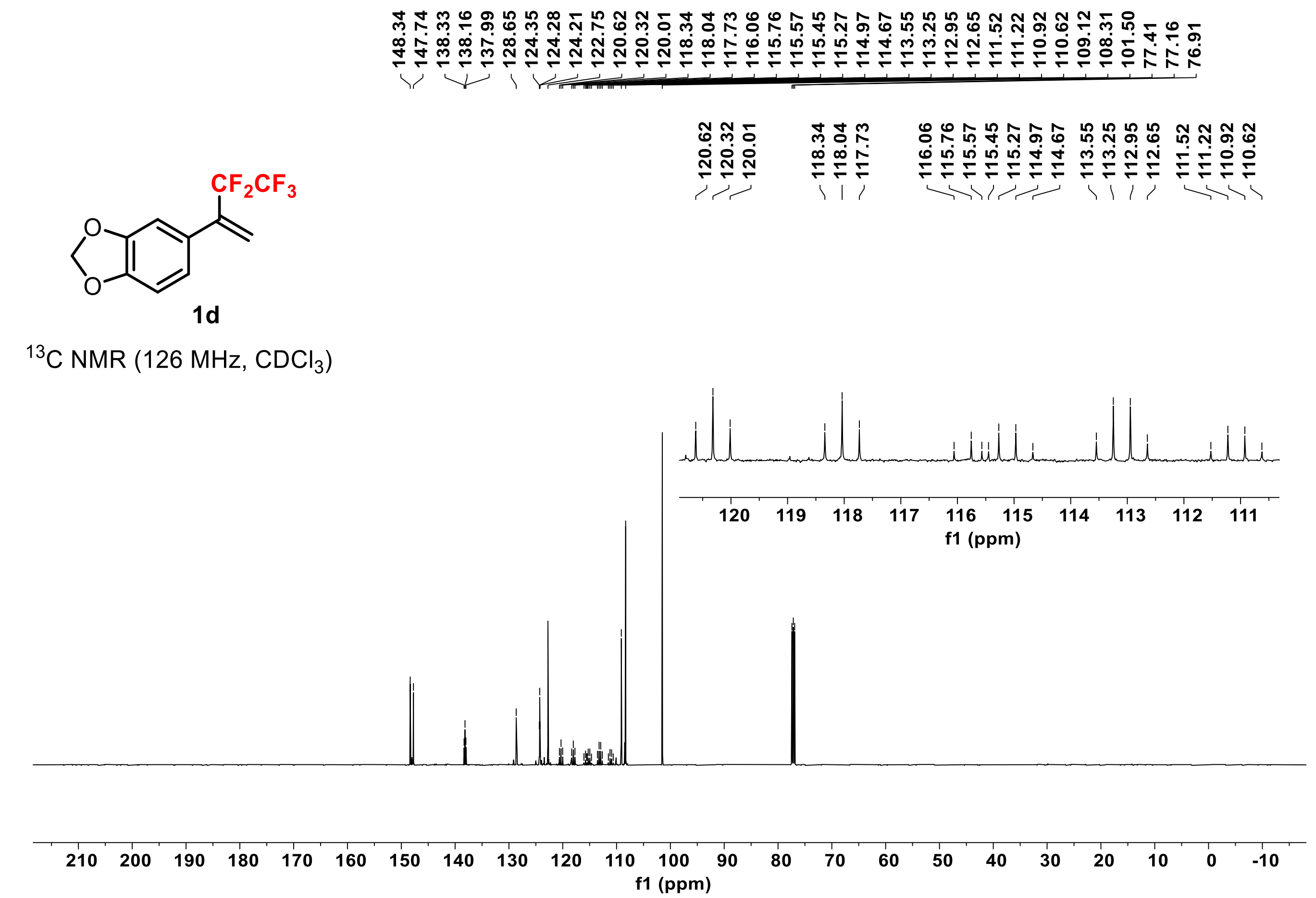
CF2CF3
1d
13C NMR (126 MHz, CDCl3)
148.34
147.74
138.33
138.16
137.99
128.65
124.35
124.28
124.21
122.75
120.62
120.32
120.01
118.34
118.04
117.73
116.06
115.76
115.57
115.45
115.27
114.97
114.67
113.55
113.25
112.95
112.65
111.52
111.22
110.92
110.62
109.12
108.31
101.50
77.41
77.16
76.91
120 119 118 117 116 115 114 113 112 111
f1 (ppm)
210 200 190 180 170 160 150 140 130 120 110 100 90 80 70 60 50 40 30 20 10 0 -10
f1 (ppm)

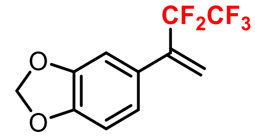

1d

^{19}F NMR (471 MHz, $CDCl_3$)

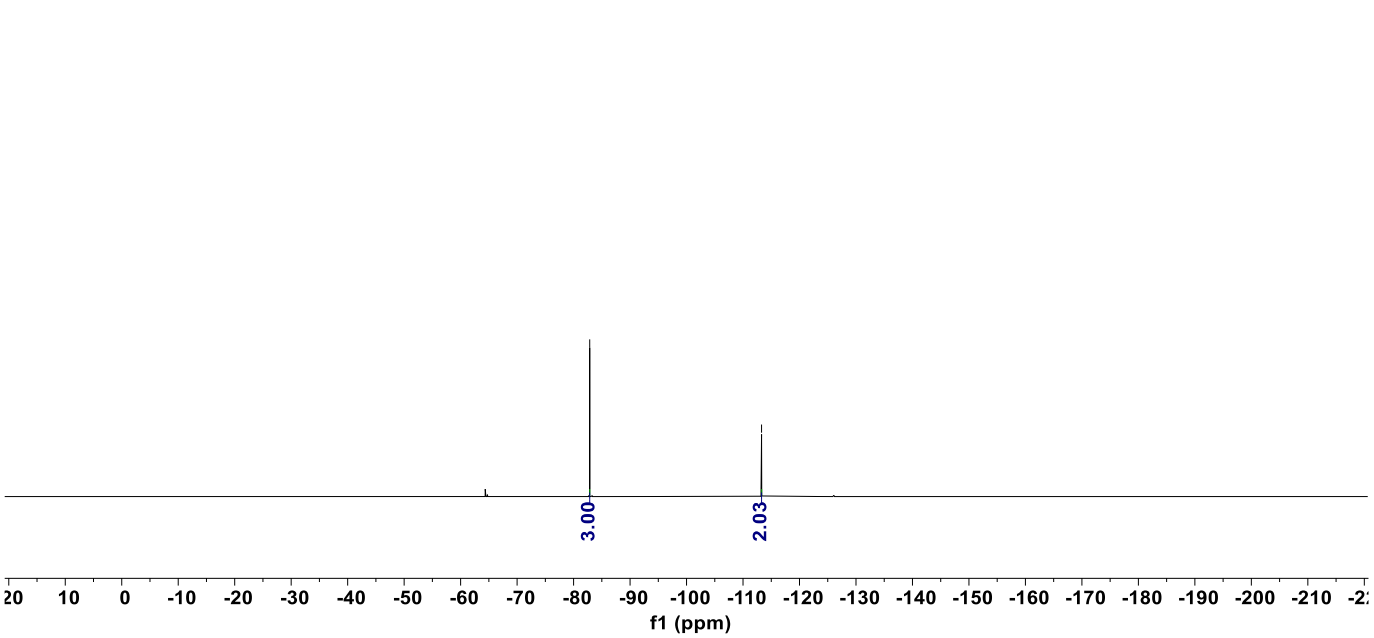

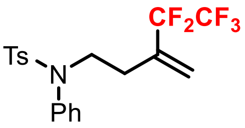

1g

^{1}H NMR (500 MHz, $CDCl_3$)

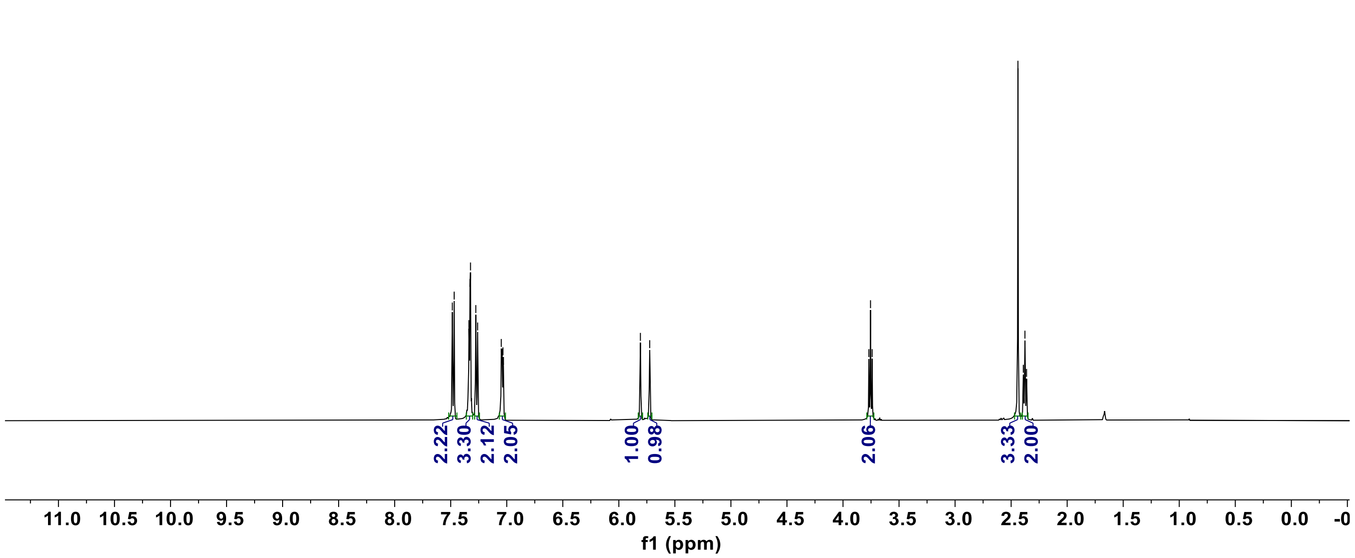

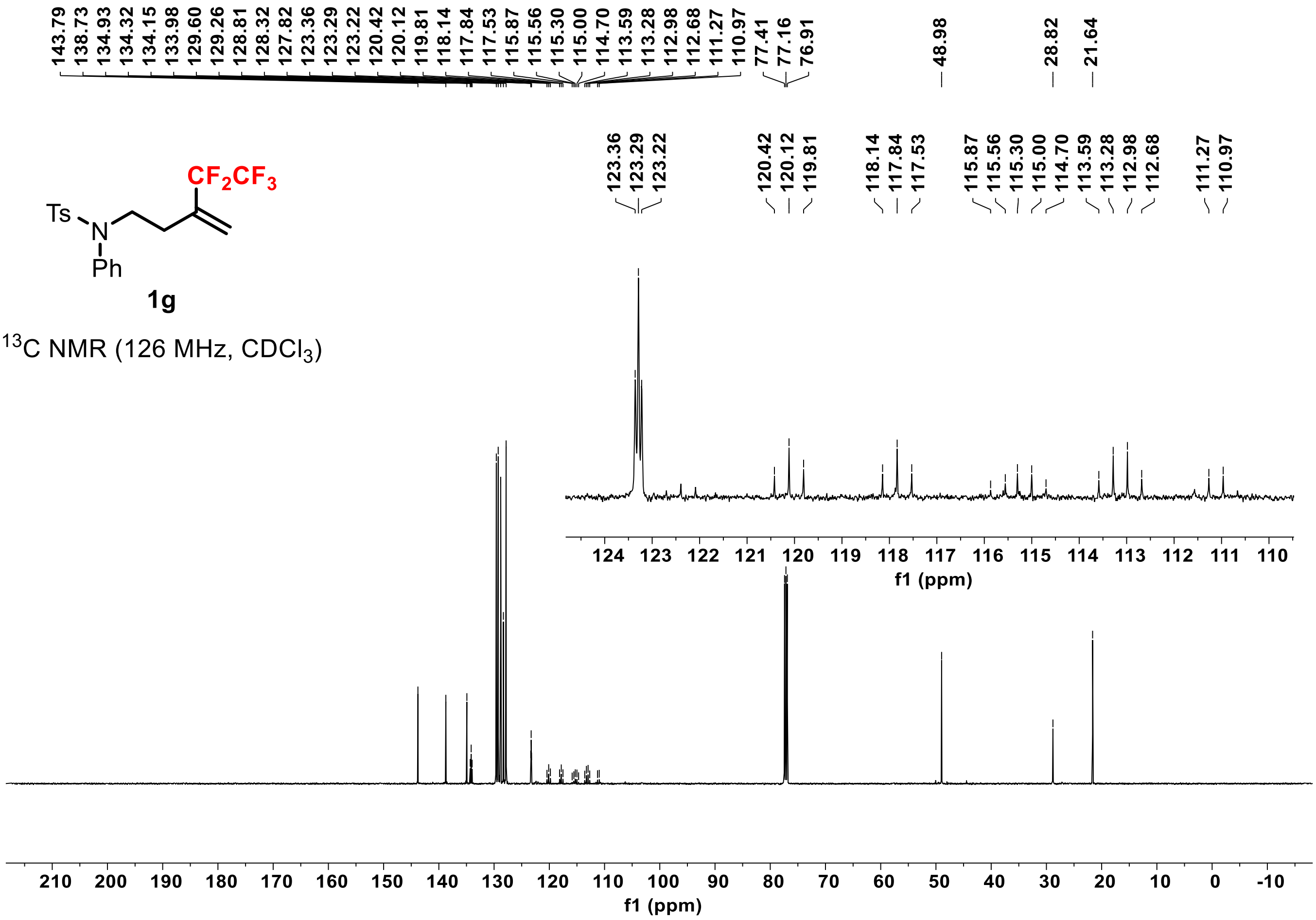

143.79
138.73
134.93
134.32
134.15
133.98
129.60
129.26
128.81
128.32
127.82
123.36
123.29
123.22
120.42
120.12
119.81
118.14
117.84
117.53
115.87
115.56
115.30
115.00
114.70
113.59
113.28
112.98
112.68
111.27
110.97
77.41
77.16
76.91
48.98
28.82
21.64
Ts
N
Ph
CF_2CF_3
1g
^{13}C NMR (126 MHz, $CDCl_3$)
f1 (ppm)

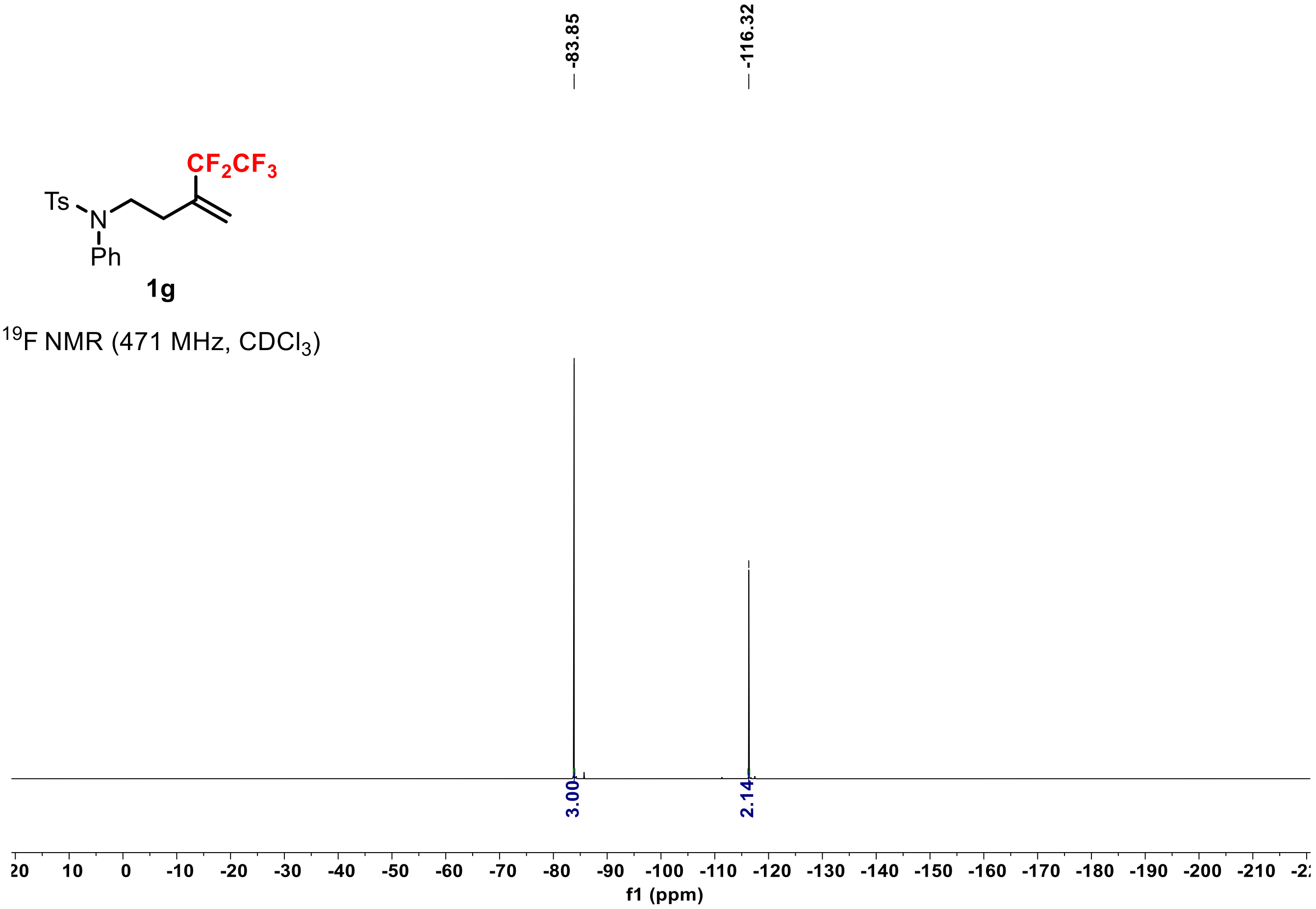

-83.85
-116.32
Ts
N
Ph
CF_2CF_3
1g
^{19}F NMR (471 MHz, $CDCl_3$)
3.00
2.14
f1 (ppm)

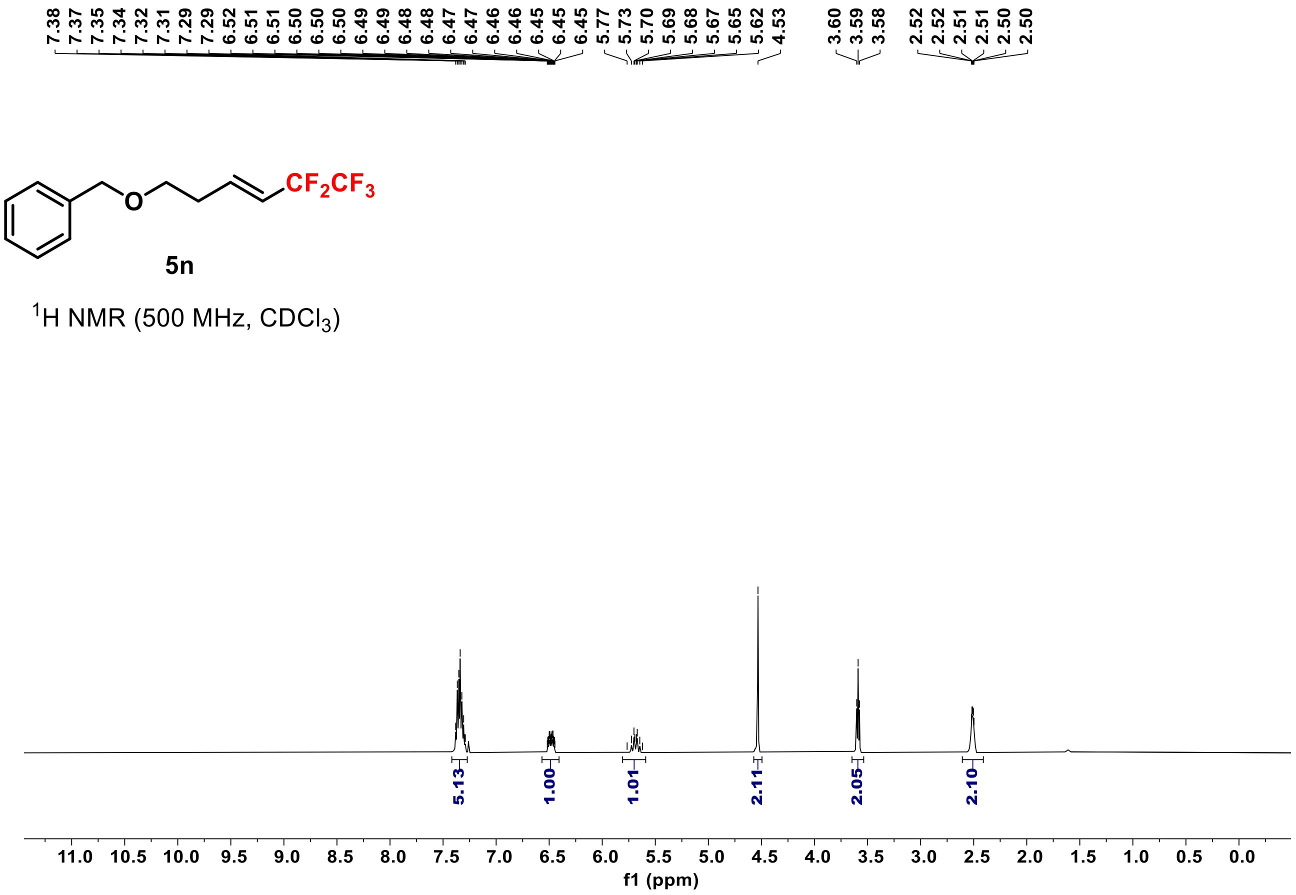

5n

^{1}H NMR (500 MHz, $CDCl_3$)

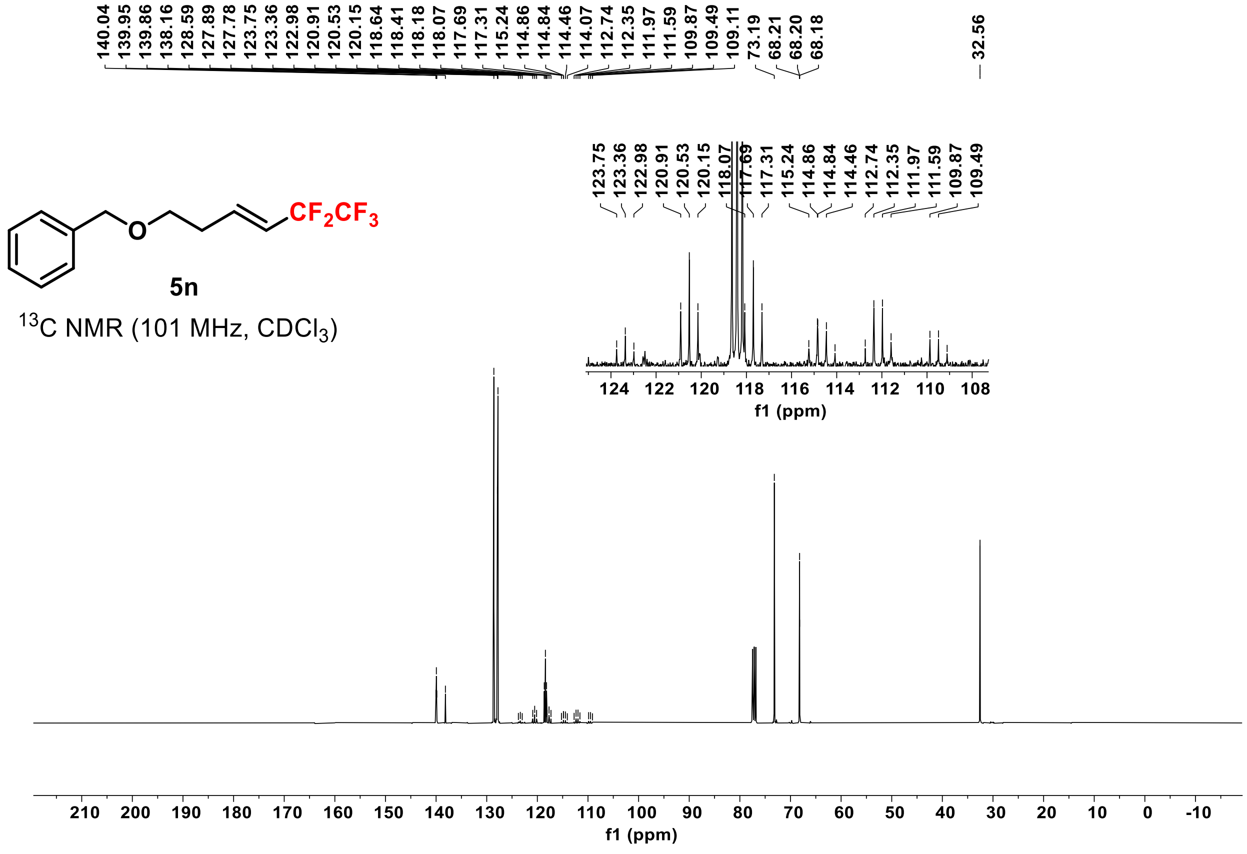

5n

^{13}C NMR (101 MHz, $CDCl_3$)

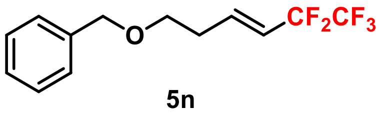

5n

^{19}F NMR (471 MHz, $CDCl_3$)

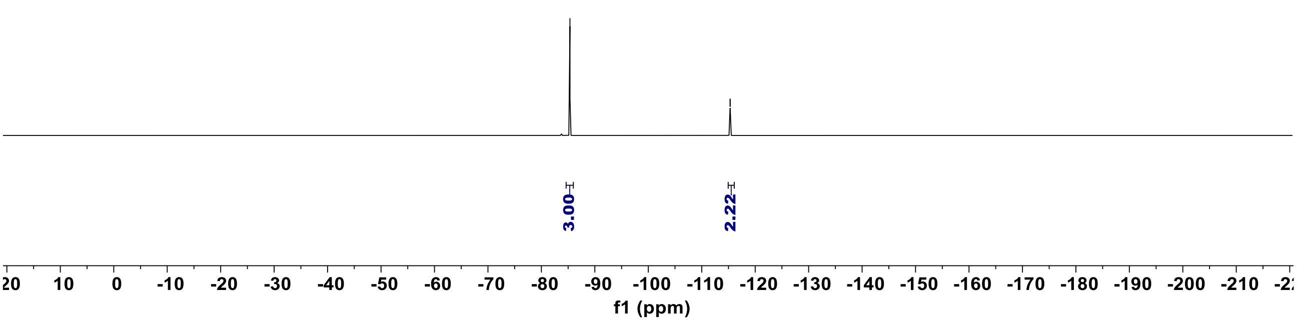

XI. Spectra of Products

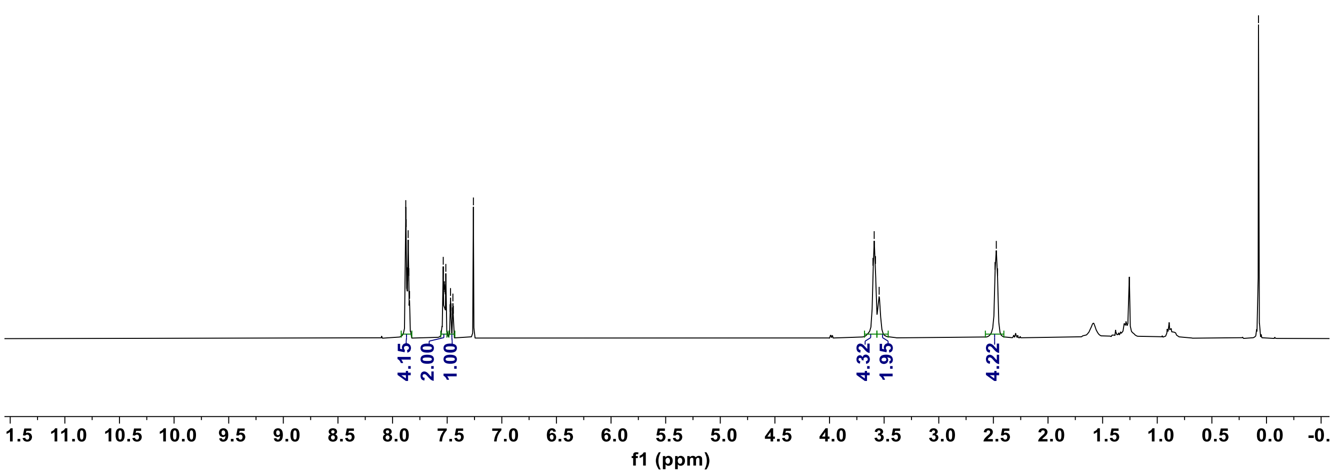

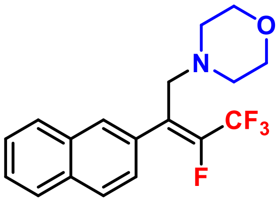

2a, *E/Z* >99:1, *E* isomer
(^{1}H NMR, 400 MHz, $CDCl_3$)

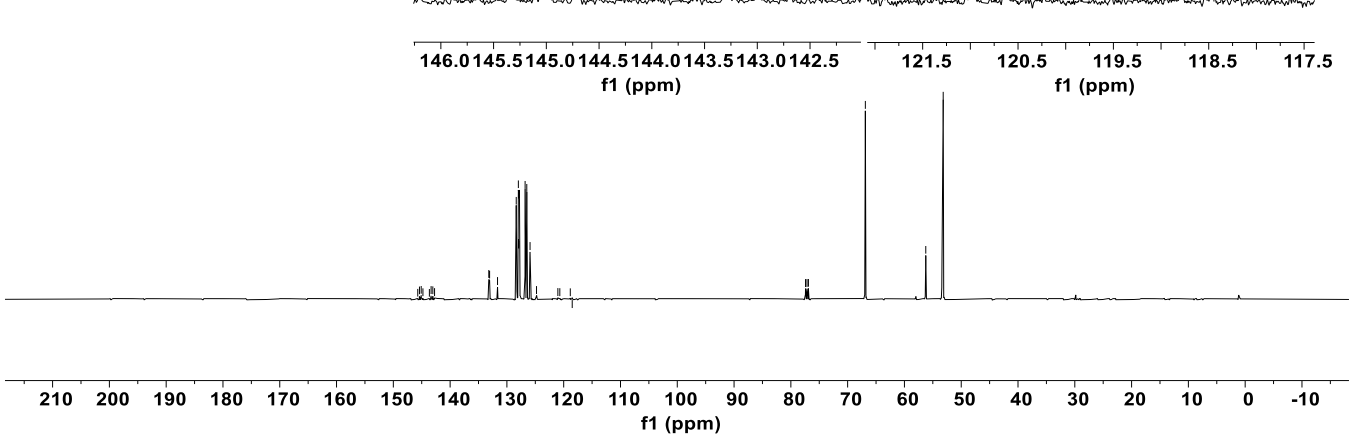

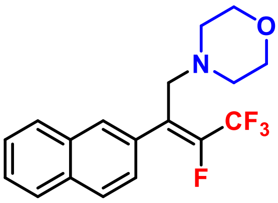

2a, *E/Z* >99:1, *E* isomer
(^{13}C NMR, 126 MHz, $CDCl_3$)

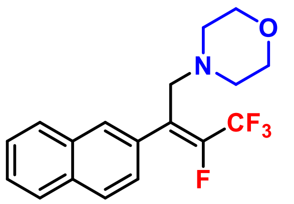

2a, *E/Z* >99:1, *E* isomer

(^{19}F NMR, 471 MHz, CDCl$_3$)

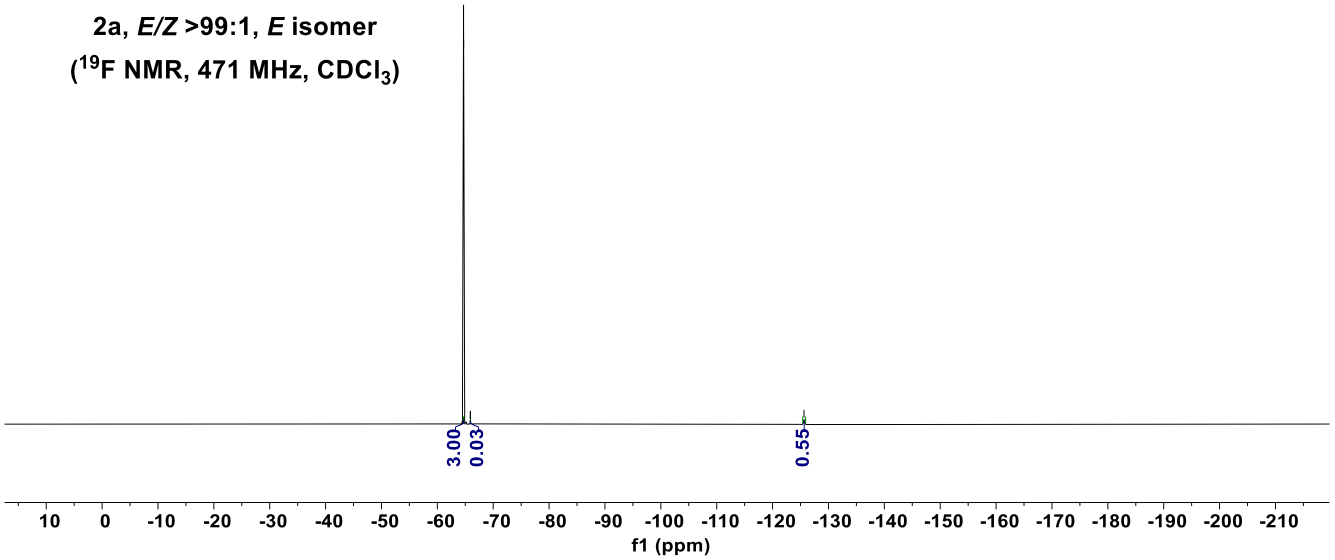

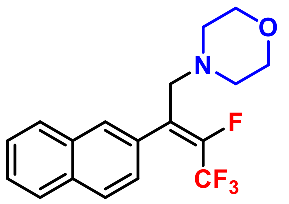

2a, *E/Z* = 2:98, *Z* isomer

(^{1}H NMR, 400 MHz, CDCl$_3$)

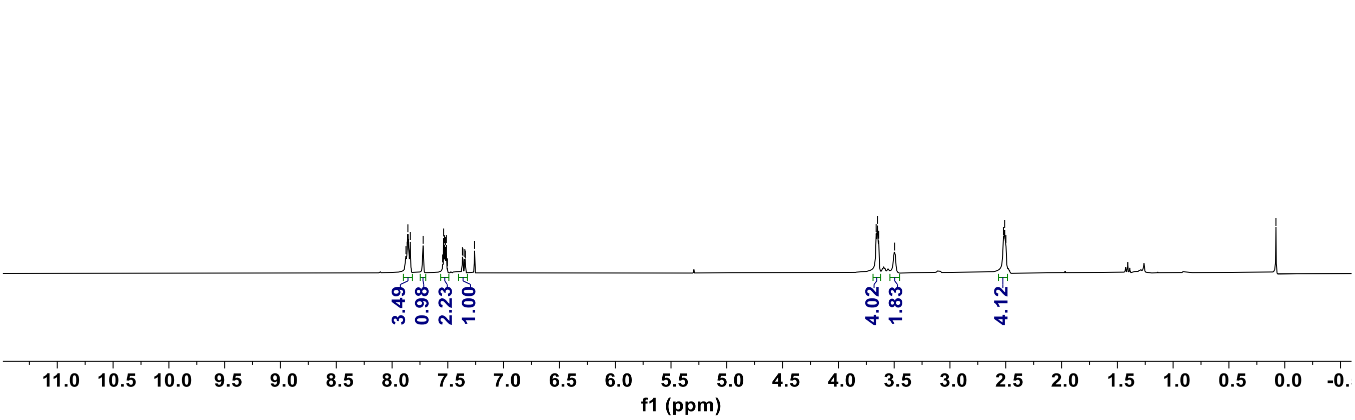

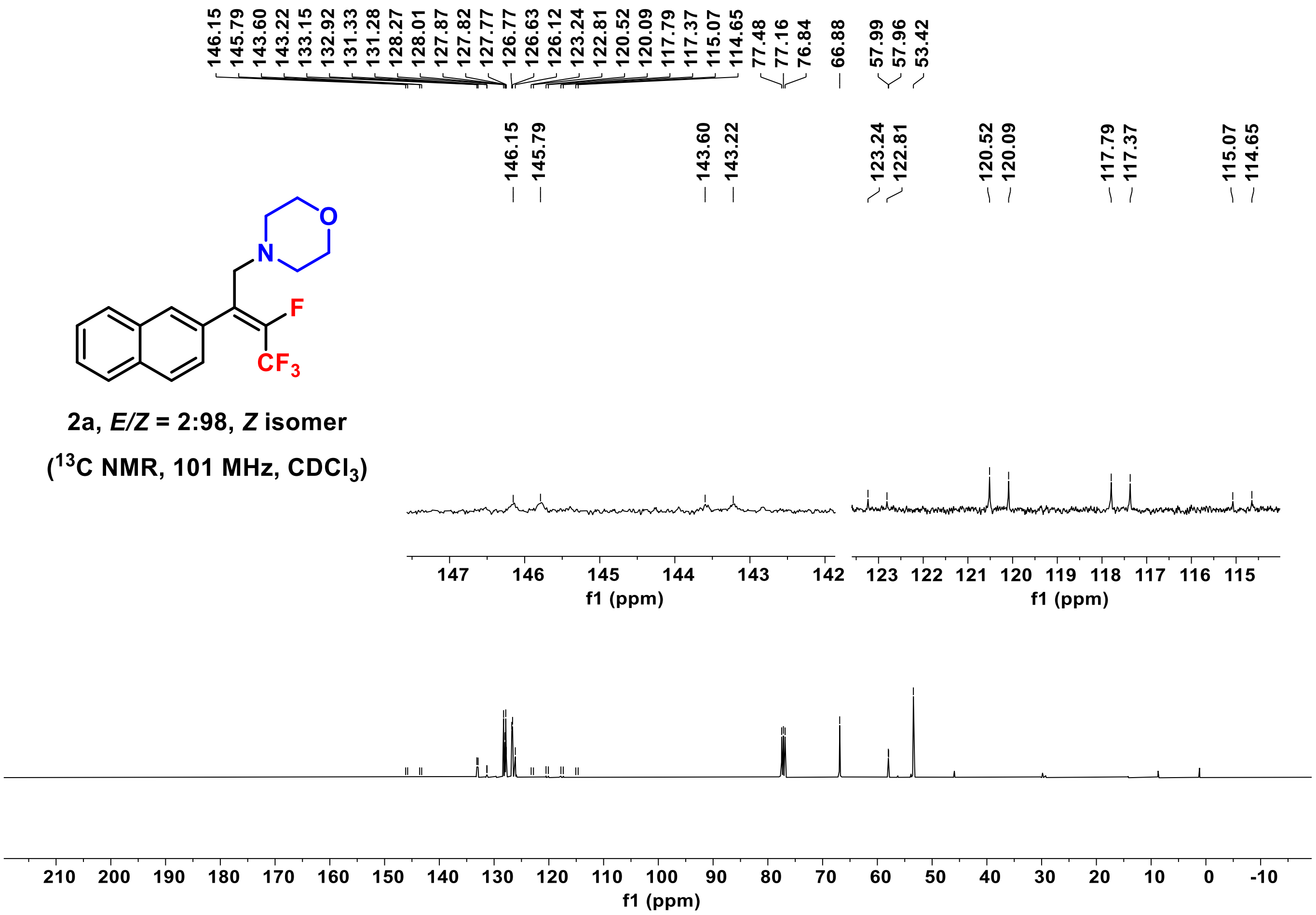

2a, *E/Z* = 2:98, *Z* isomer

(^{13}C NMR, 101 MHz, $CDCl_3$)

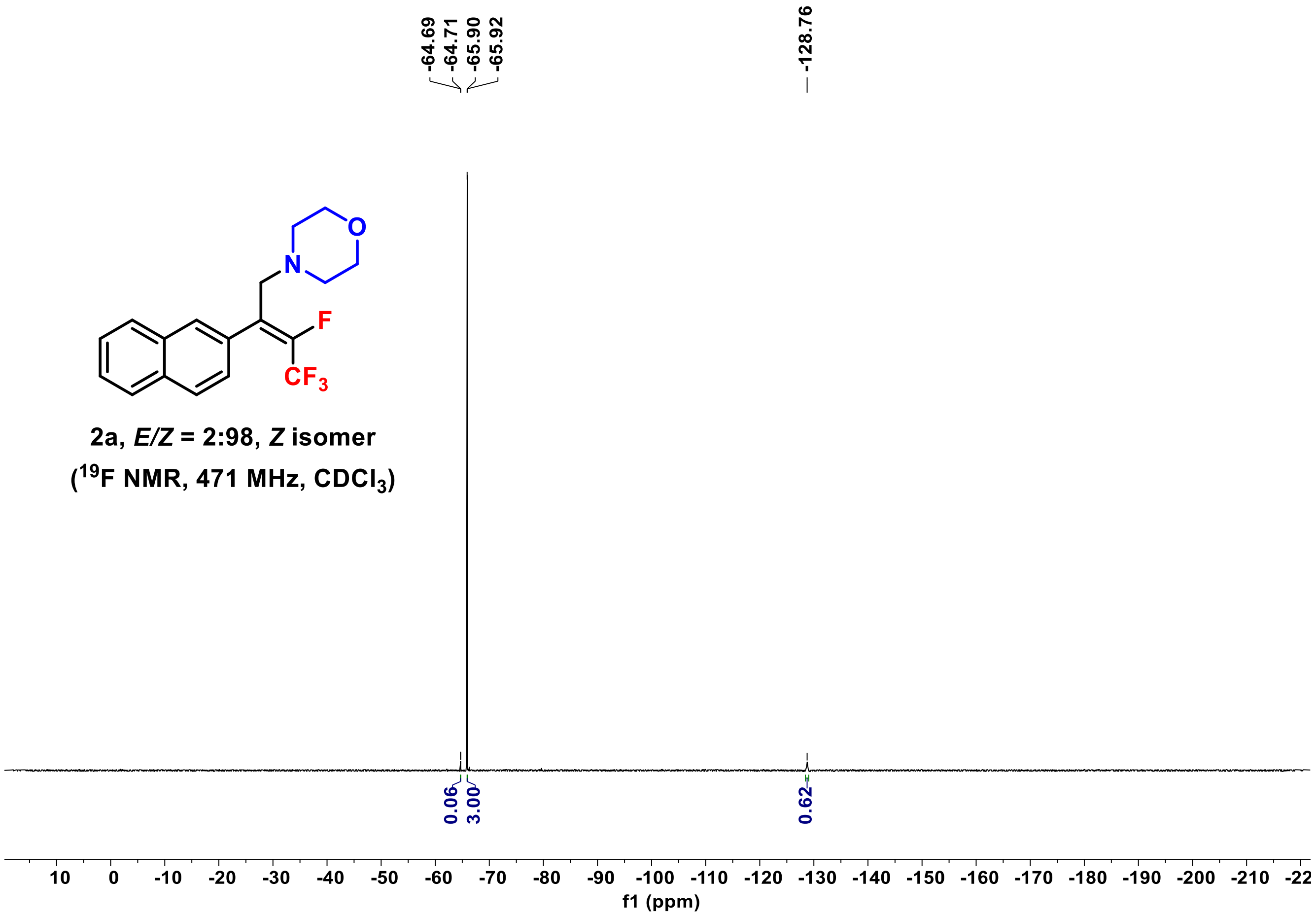

2a, *E/Z* = 2:98, *Z* isomer

(^{19}F NMR, 471 MHz, $CDCl_3$)

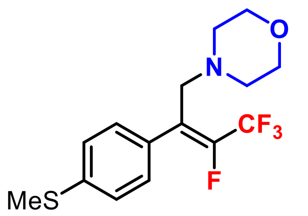

2b, *E/Z* >99:1, *E* isomer

(^{1}H NMR, 500 MHz, CDCl$_3$)

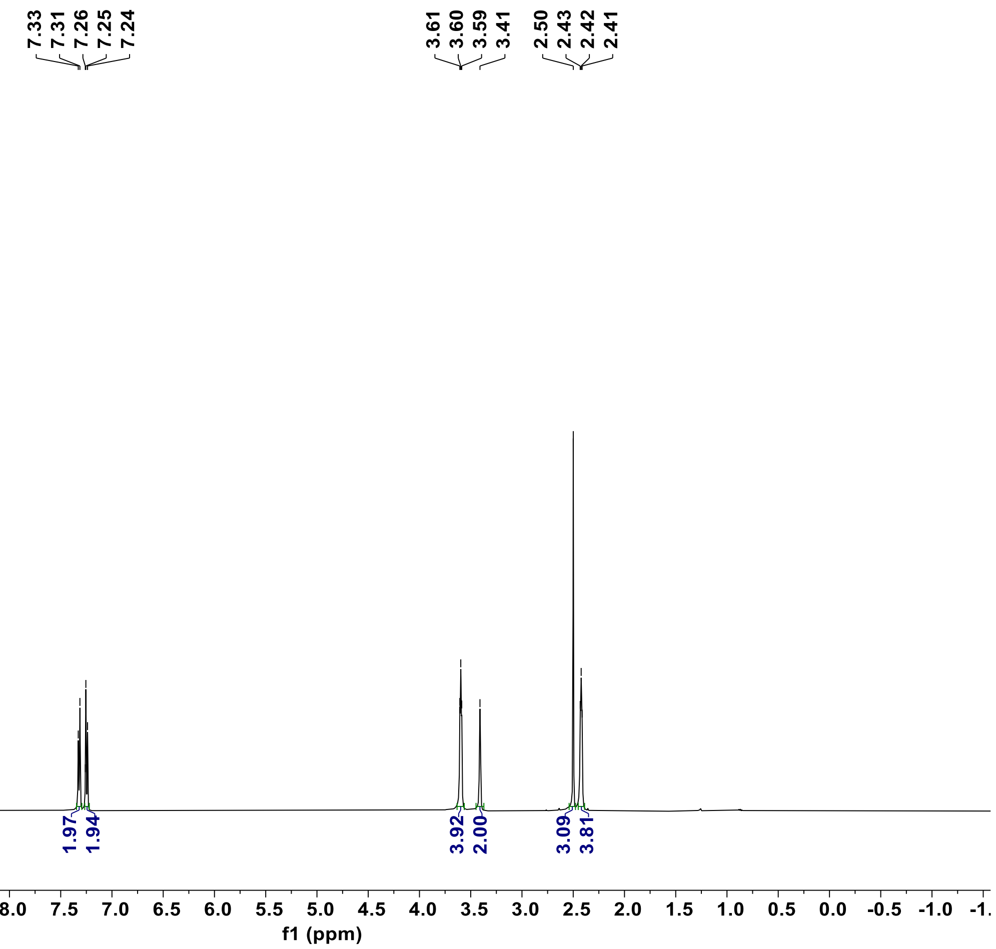

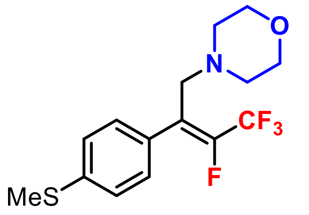

2b, *E/Z* >99:1, *E* isomer

(^{13}C NMR, 126 MHz, CDCl$_3$)

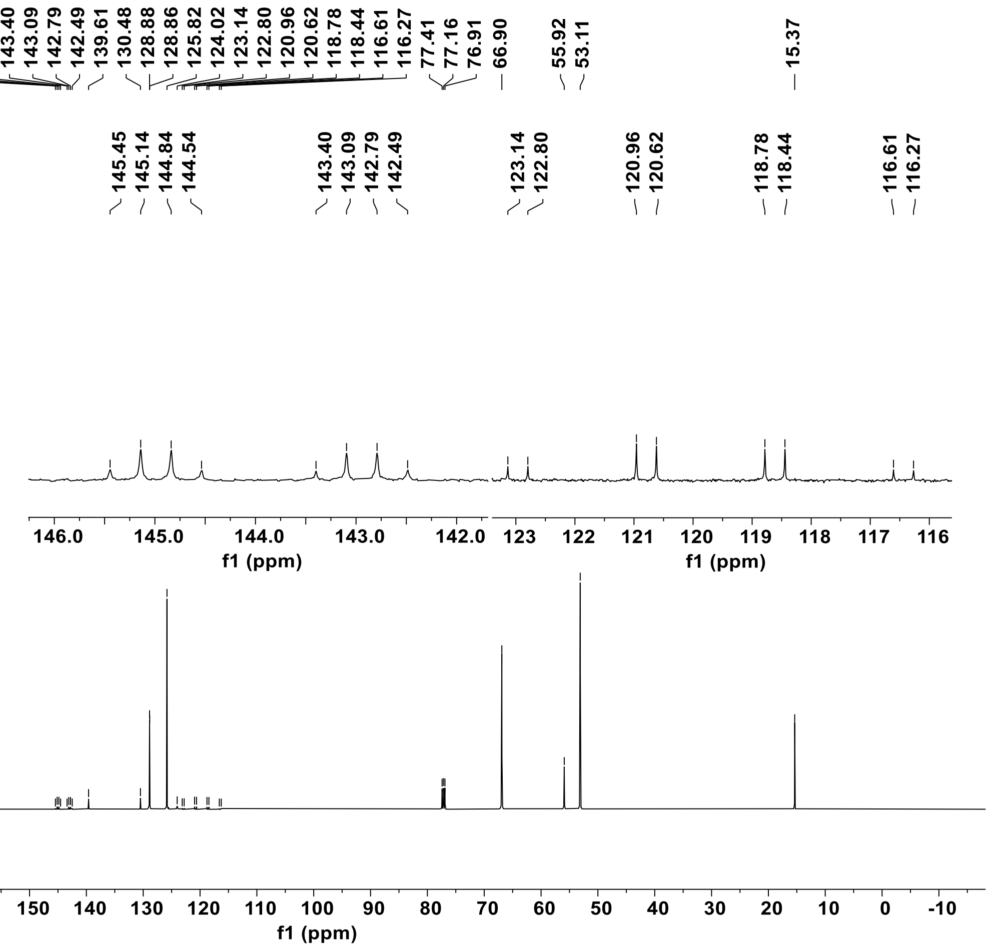

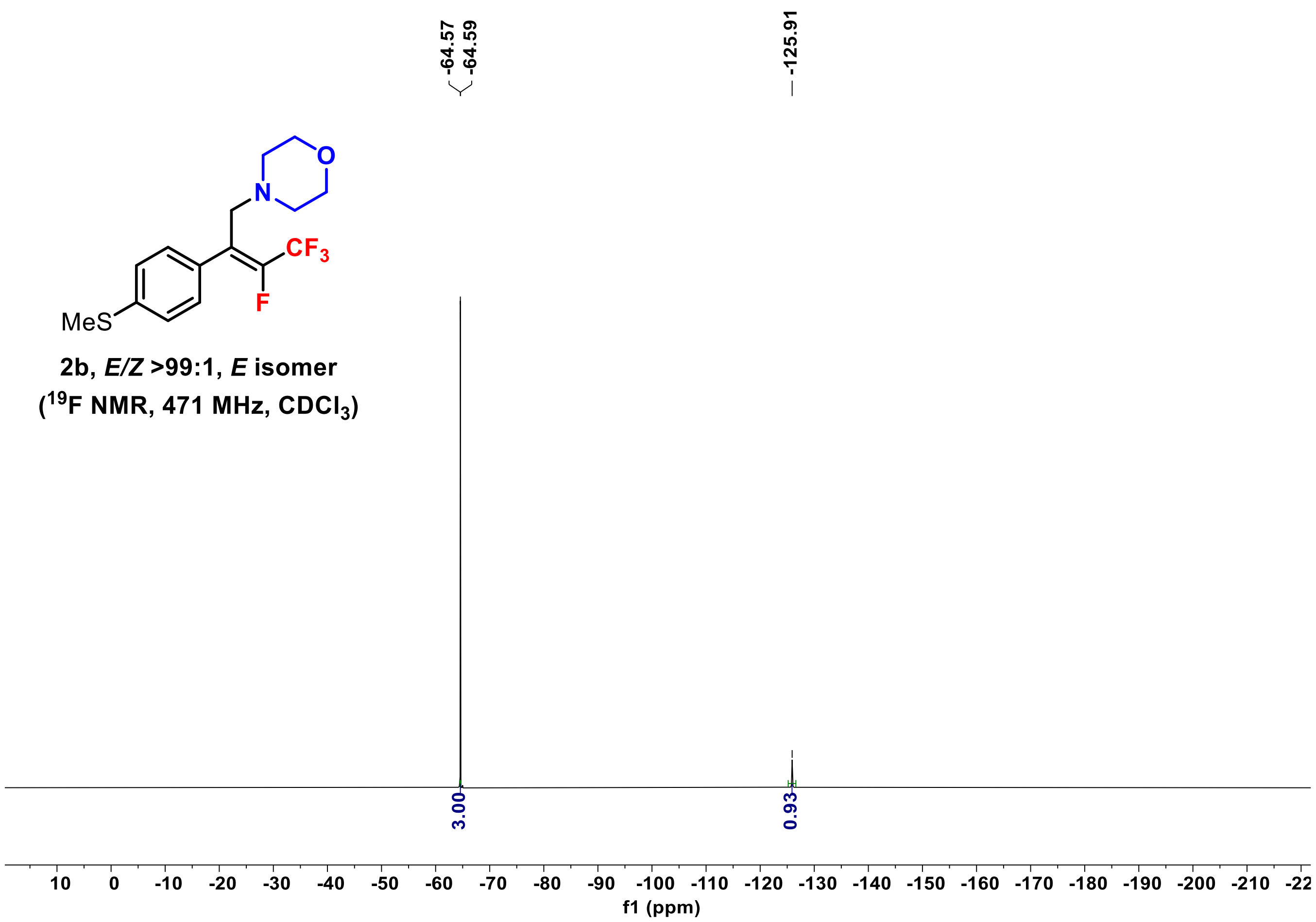

2b, *E/Z* >99:1, *E* isomer
(^{19}F NMR, 471 MHz, CDCl$_3$)

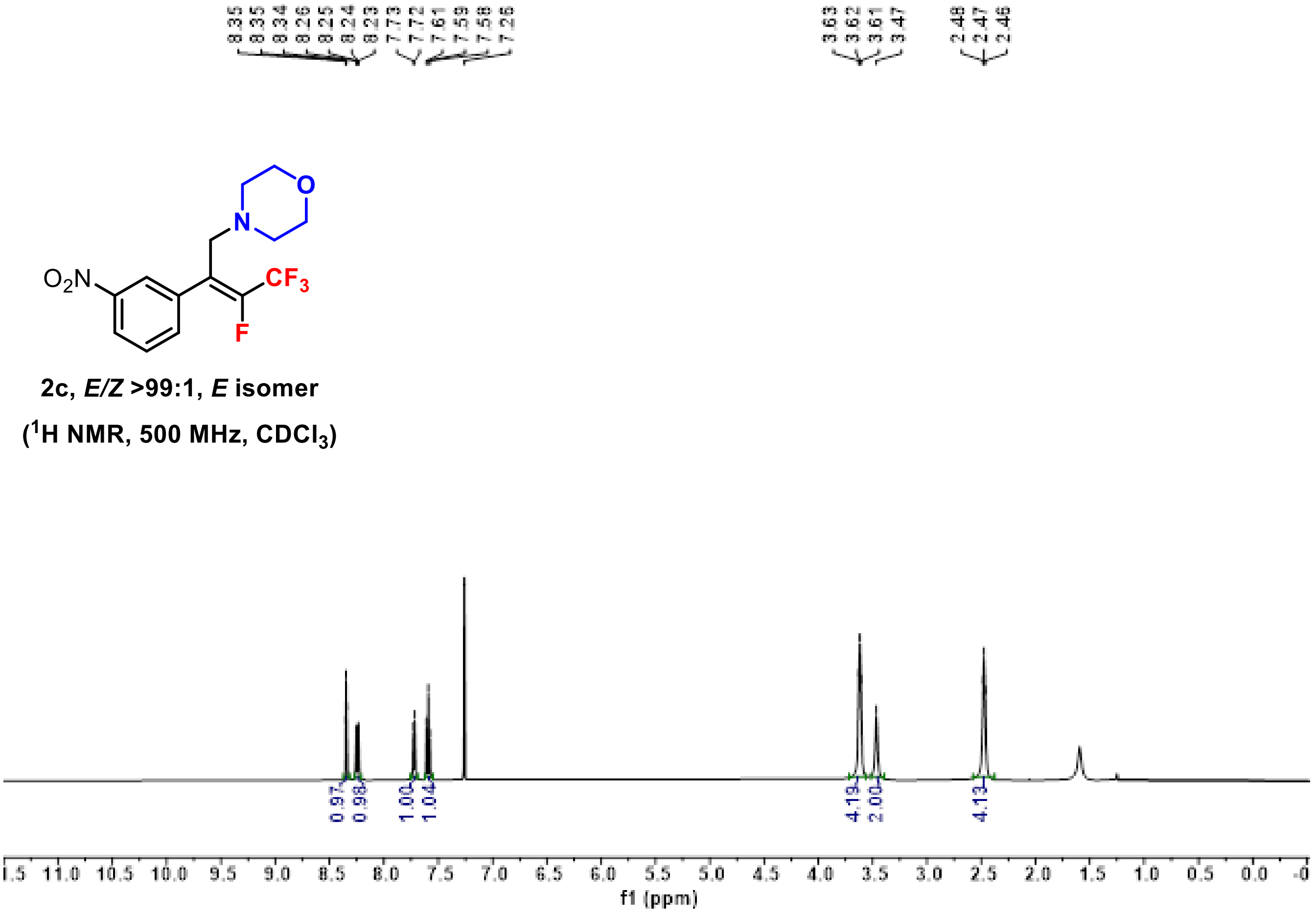

2c, *E/Z* >99:1, *E* isomer
(^{1}H NMR, 500 MHz, CDCl$_3$)

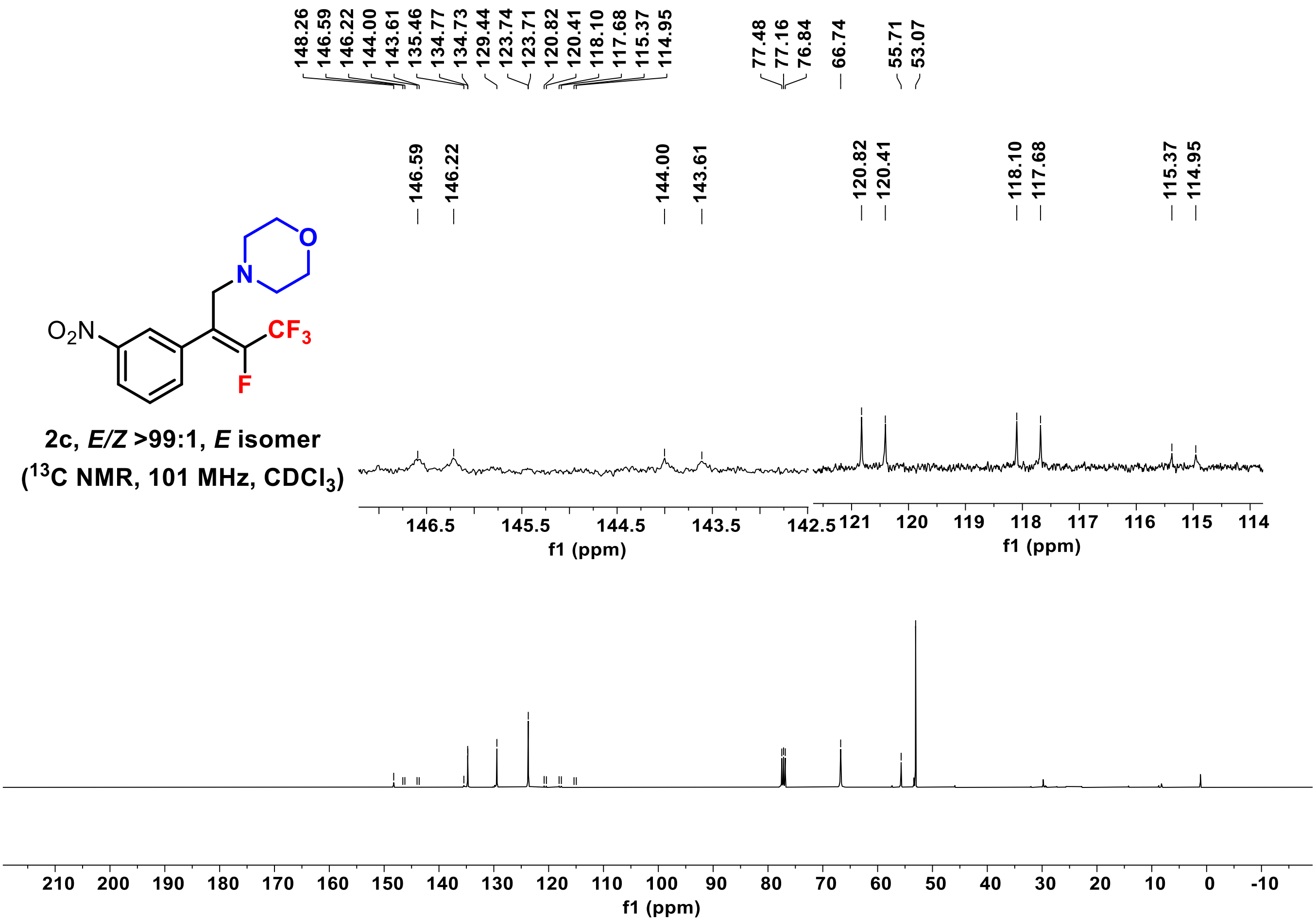
148.26
146.59
146.22
144.00
143.61
135.46
134.77
134.73
129.44
123.74
123.71
120.82
120.41
118.10
117.68
115.37
114.95
77.48
77.16
76.84
66.74
55.71
53.07
O2N
N
O
CF3
F
2c, E/Z >99:1, E isomer
(13C NMR, 101 MHz, CDCl3)
f1 (ppm)

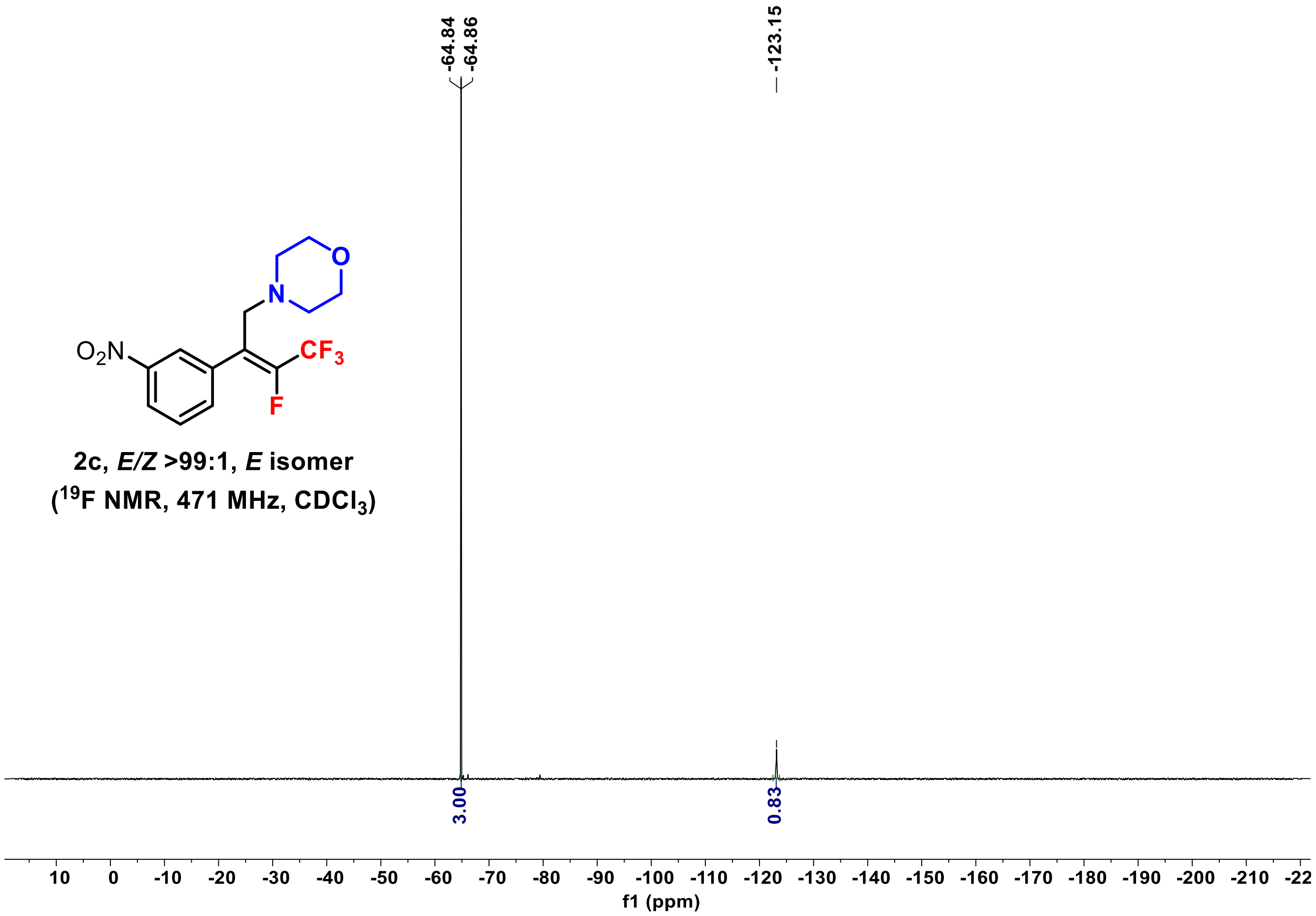
-64.84
-64.86
-123.15
O2N
N
O
CF3
F
2c, E/Z >99:1, E isomer
(19F NMR, 471 MHz, CDCl3)
3.00
0.83
f1 (ppm)

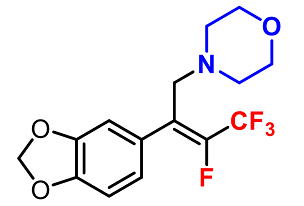

2d, *E/Z* = 96:4, *E* isomer

(^{1}H NMR, 500 MHz, $CDCl_3$)

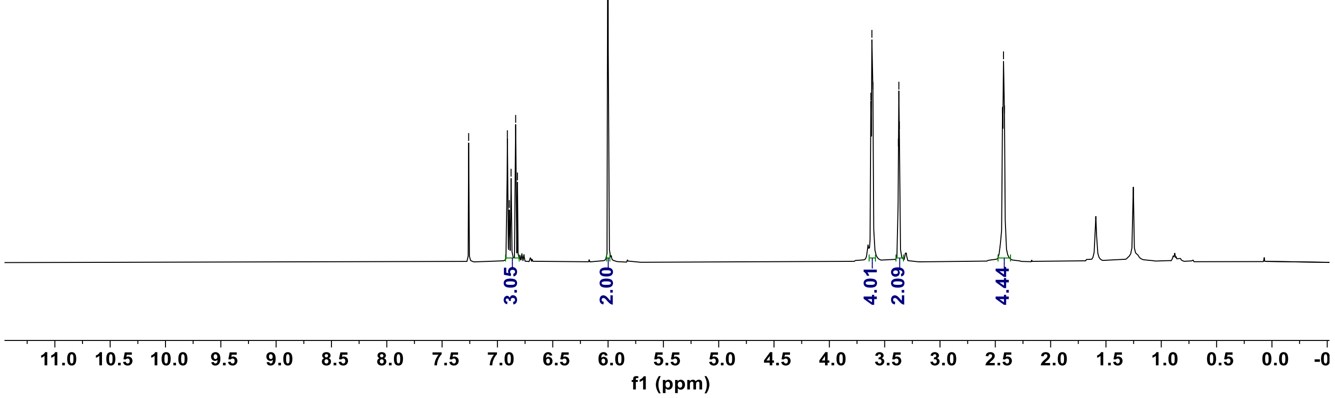

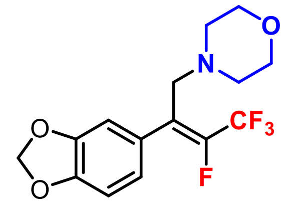

2d, *E/Z* = 96:4, *E* isomer

(^{13}C NMR, 126 MHz, $CDCl_3$)

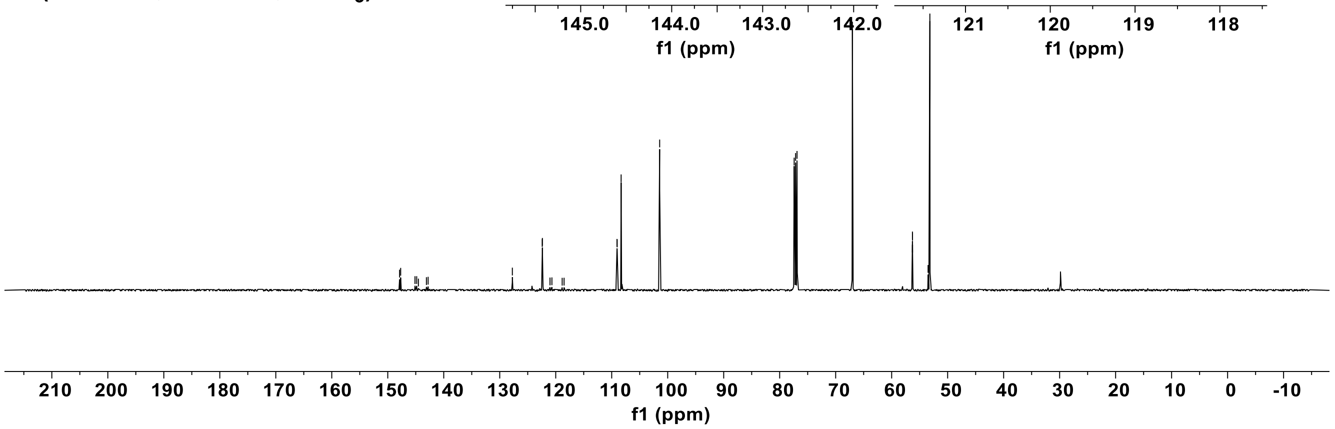

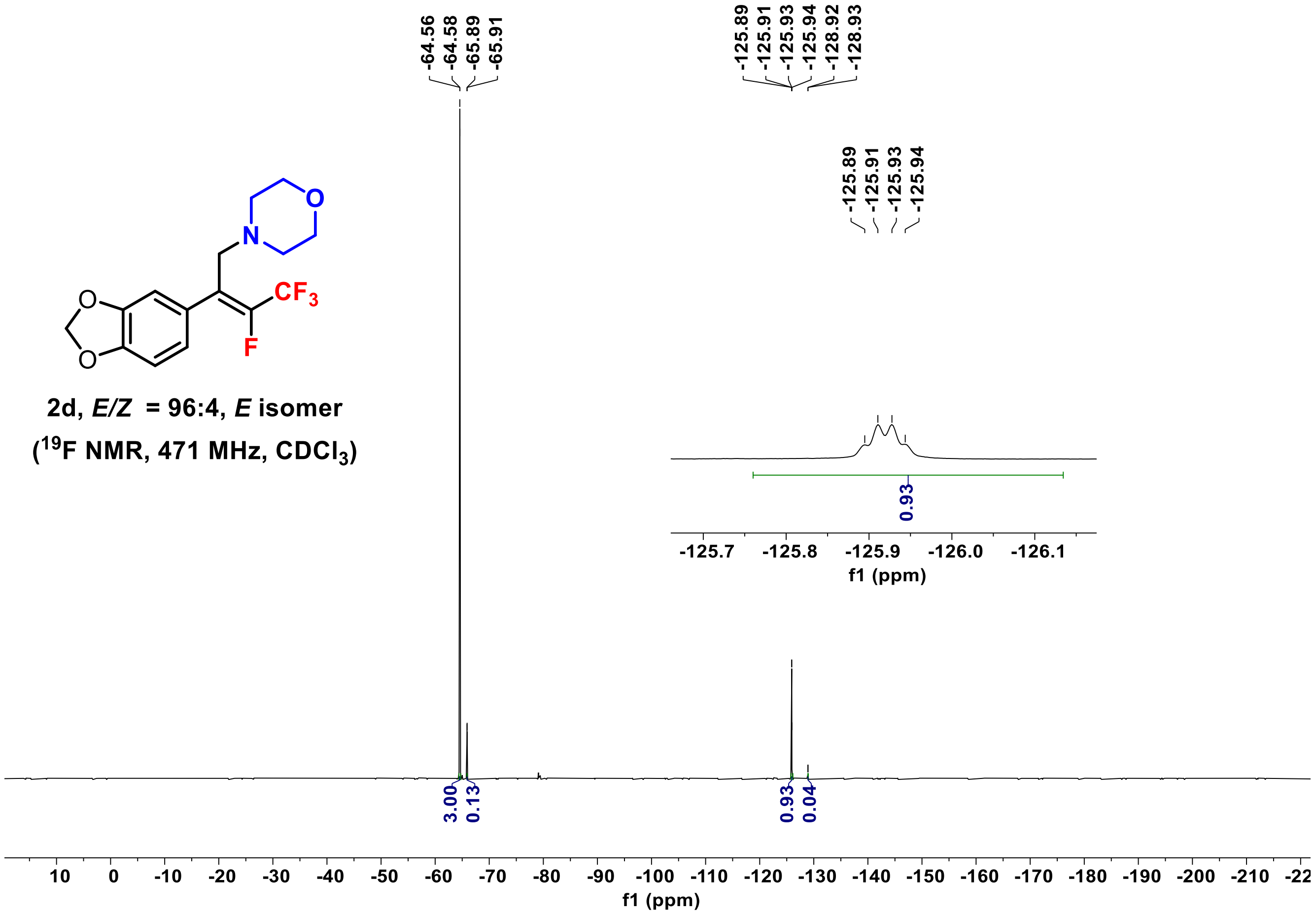

2d, ***E/Z*** **= 96:4,** ***E*** **isomer**

(^{19}F NMR, 471 MHz, CDCl$_3$)

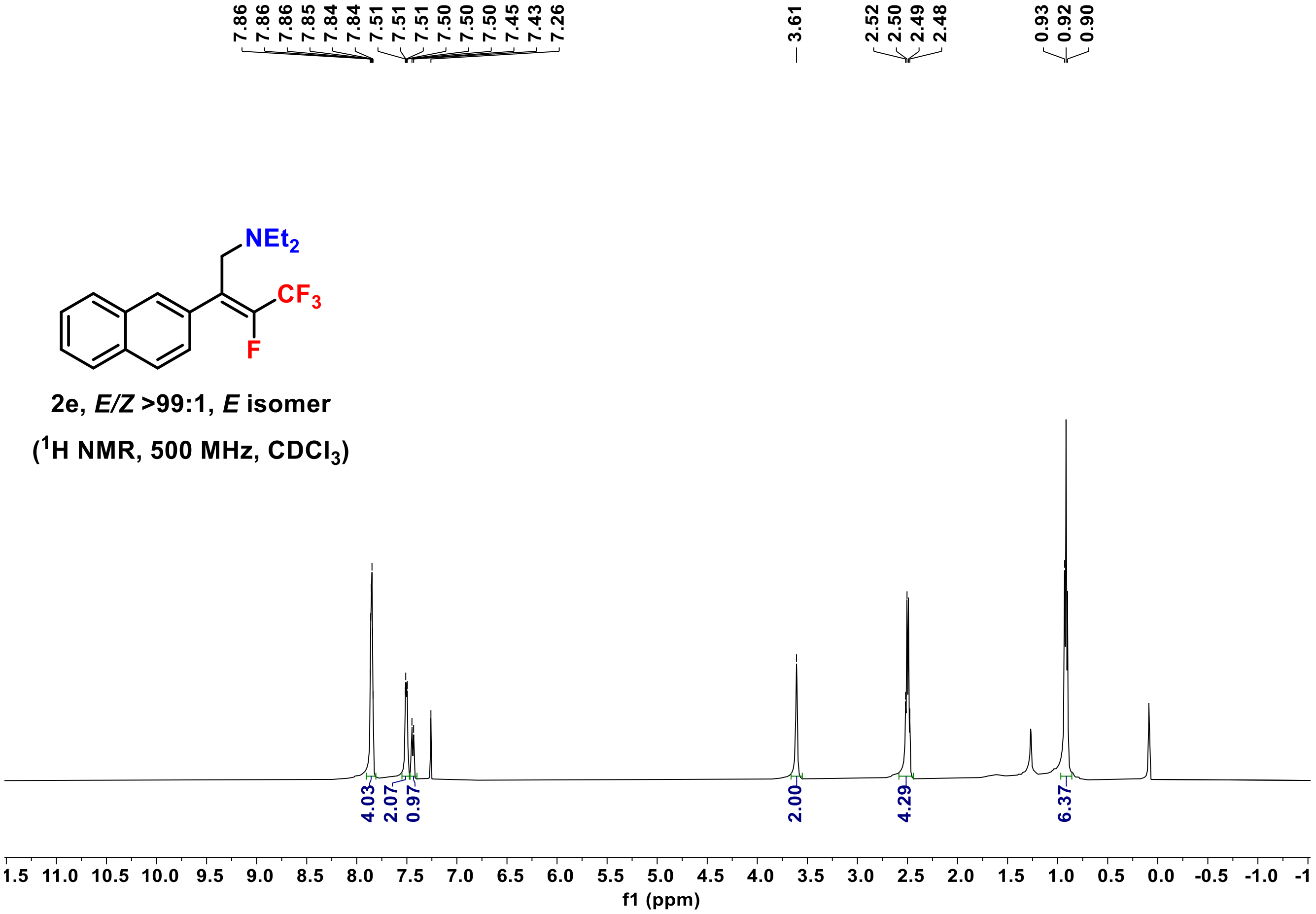

2e, ***E/Z*** **>99:1,** ***E*** **isomer**

(^{1}H NMR, 500 MHz, CDCl$_3$)

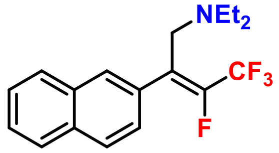

2e, *E/Z* >99:1, *E* isomer
(^{13}C NMR, 126 MHz, $CDCl_3$)

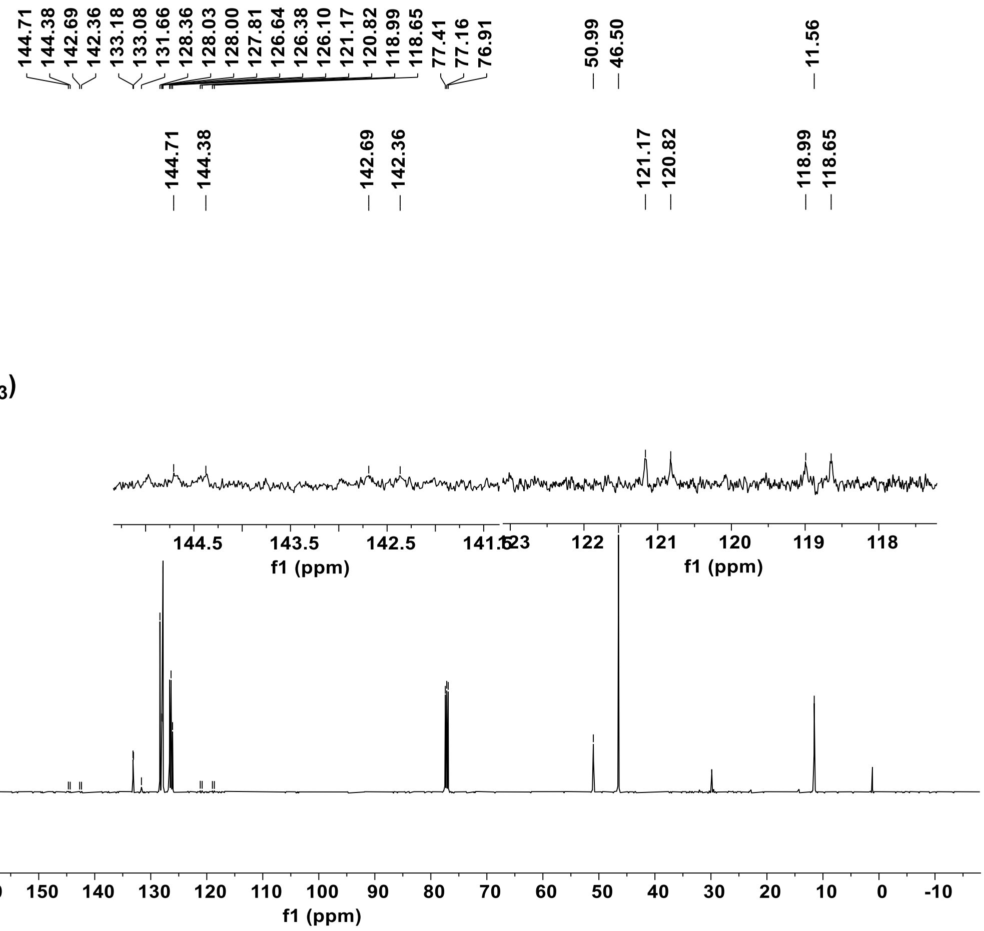

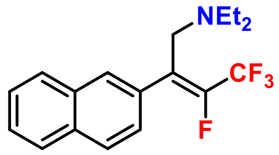

2e, *E/Z* >99:1, *E* isomer
(^{19}F NMR, 471 MHz, $CDCl_3$)

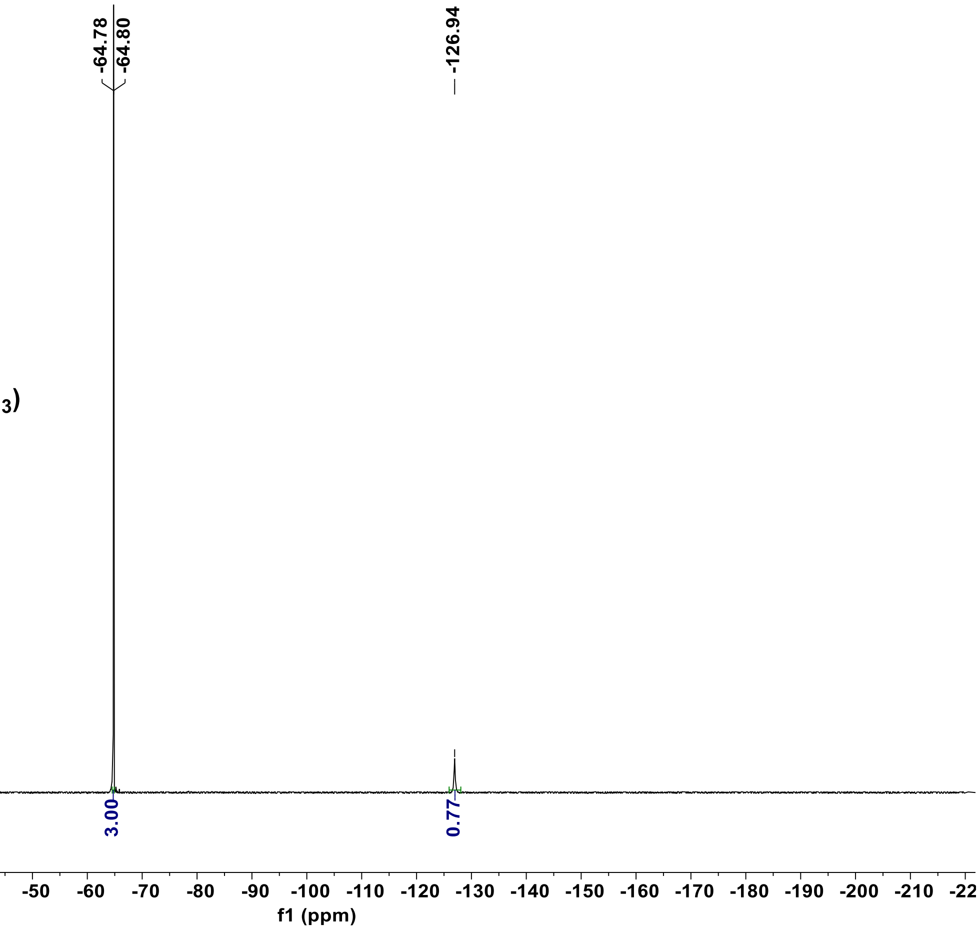

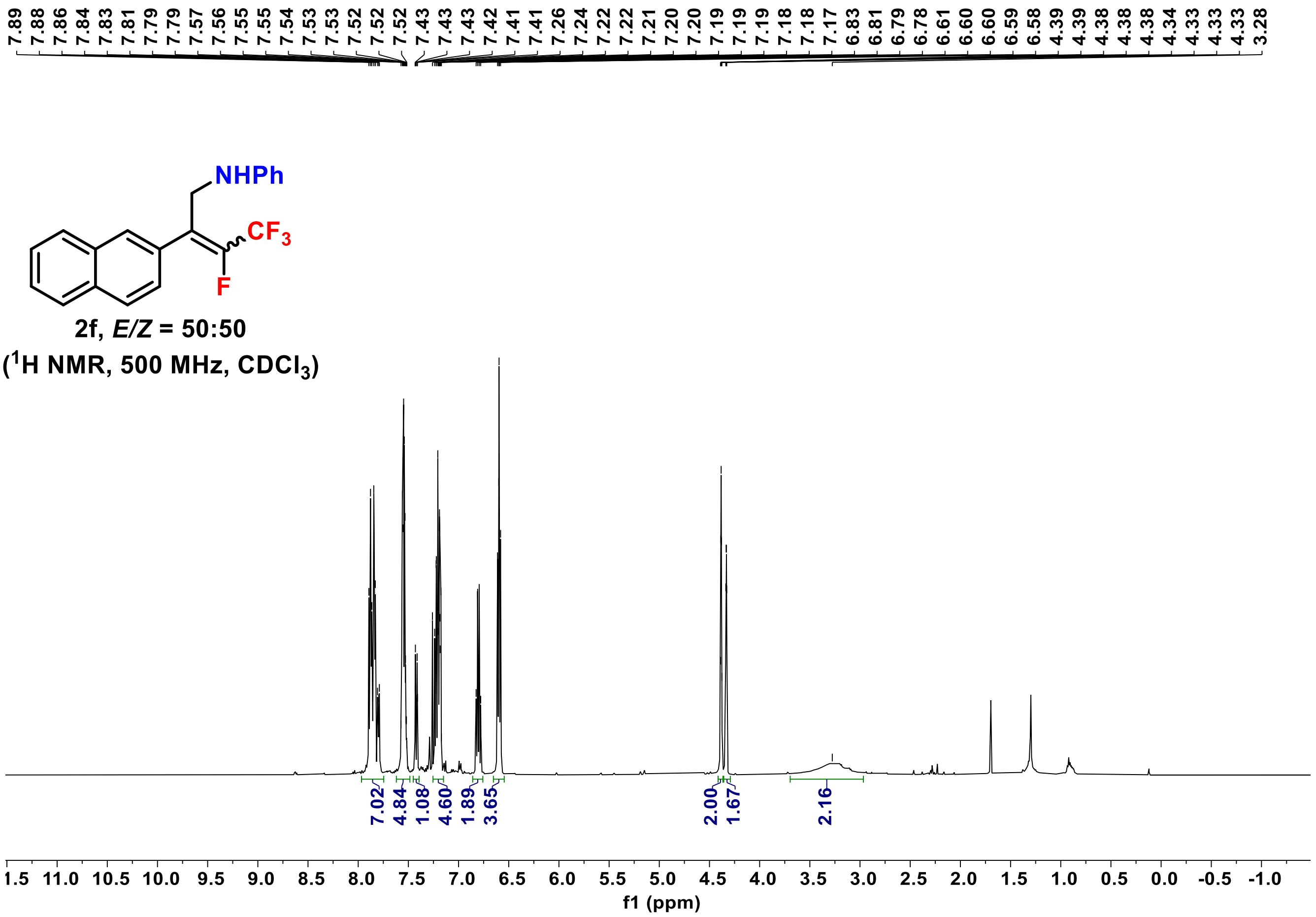
7.89 7.88 7.86 7.84 7.83 7.81 7.79 7.79 7.57 7.56 7.55 7.55 7.54 7.53 7.53 7.52 7.52 7.52 7.43 7.43 7.43 7.42 7.41 7.41 7.26 7.24 7.22 7.22 7.21 7.20 7.20 7.19 7.19 7.19 7.18 7.18 7.17 6.83 6.81 6.79 6.78 6.61 6.60 6.60 6.59 6.58 4.39 4.39 4.38 4.38 4.38 4.34 4.33 4.33 3.28
NHPh
CF3
F
2f, E/Z = 50:50
(1H NMR, 500 MHz, CDCl3)
7.02 4.84 1.08 4.60 1.89 3.65 2.00 1.67 2.16
11.5 11.0 10.5 10.0 9.5 9.0 8.5 8.0 7.5 7.0 6.5 6.0 5.5 5.0 4.5 4.0 3.5 3.0 2.5 2.0 1.5 1.0 0.5 0.0 -0.5 -1.0
f1 (ppm)

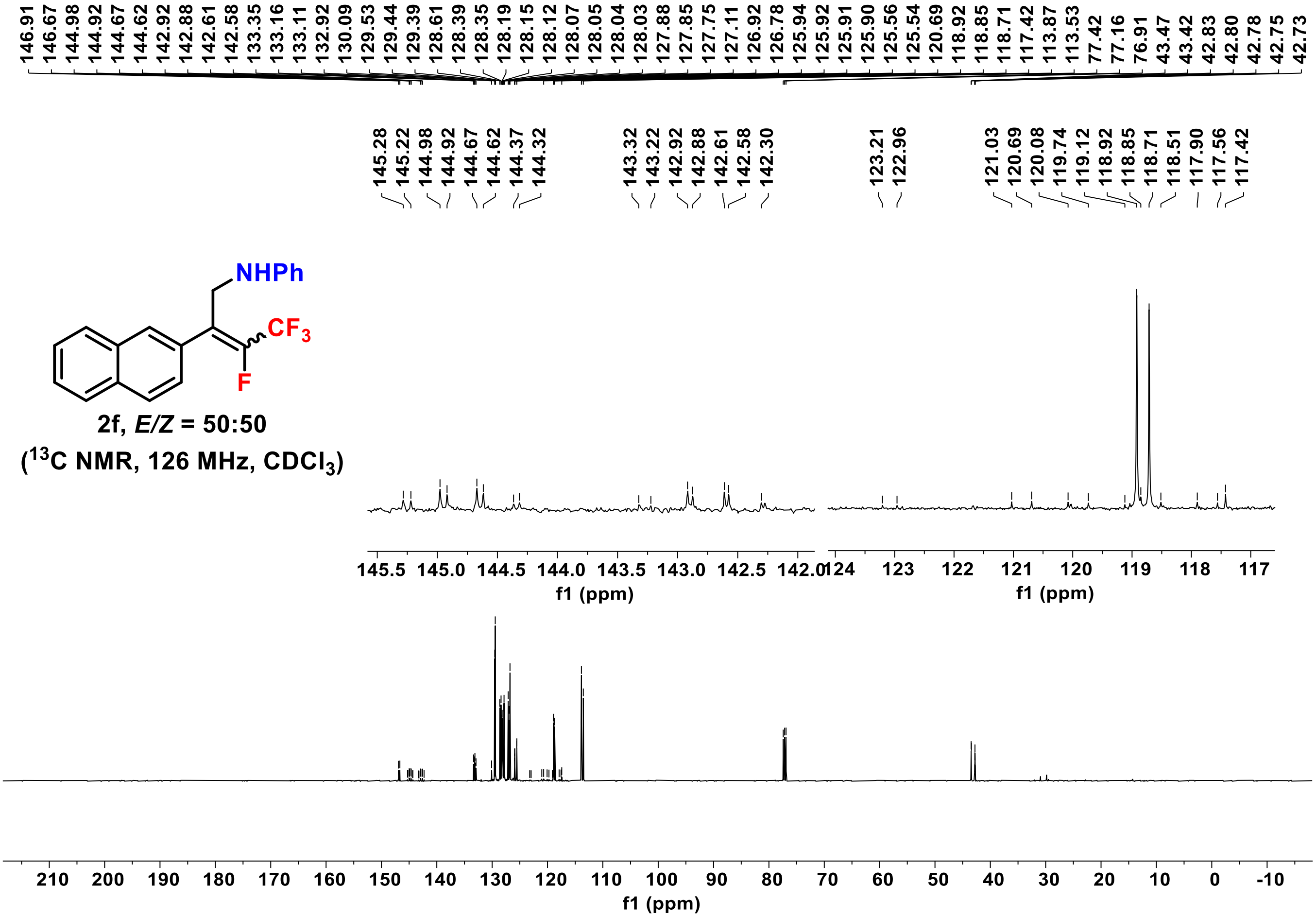
146.91 146.67 144.98 144.92 144.67 144.62 142.92 142.88 142.61 142.58 133.35 133.16 133.11 132.92 130.09 129.53 129.44 129.39 128.61 128.39 128.35 128.19 128.15 128.12 128.07 128.05 128.04 128.03 127.88 127.85 127.75 127.11 126.92 126.78 125.94 125.92 125.91 125.90 125.56 125.54 120.69 118.92 118.85 118.71 117.42 113.87 113.53 77.42 77.16 76.91 43.47 43.42 42.83 42.80 42.78 42.75 42.73
145.28 145.22 144.98 144.92 144.67 144.62 144.37 144.32
143.32 143.22 142.92 142.88 142.61 142.58 142.30
123.21 122.96
121.03 120.69 120.08 119.74 119.12 118.92 118.85 118.71 118.51 117.90 117.56 117.42
NHPh
CF3
F
2f, E/Z = 50:50
(13C NMR, 126 MHz, CDCl3)
145.5 145.0 144.5 144.0 143.5 143.0 142.5 142.0
f1 (ppm)
124 123 122 121 120 119 118 117
f1 (ppm)
210 200 190 180 170 160 150 140 130 120 110 100 90 80 70 60 50 40 30 20 10 0 -10
f1 (ppm)

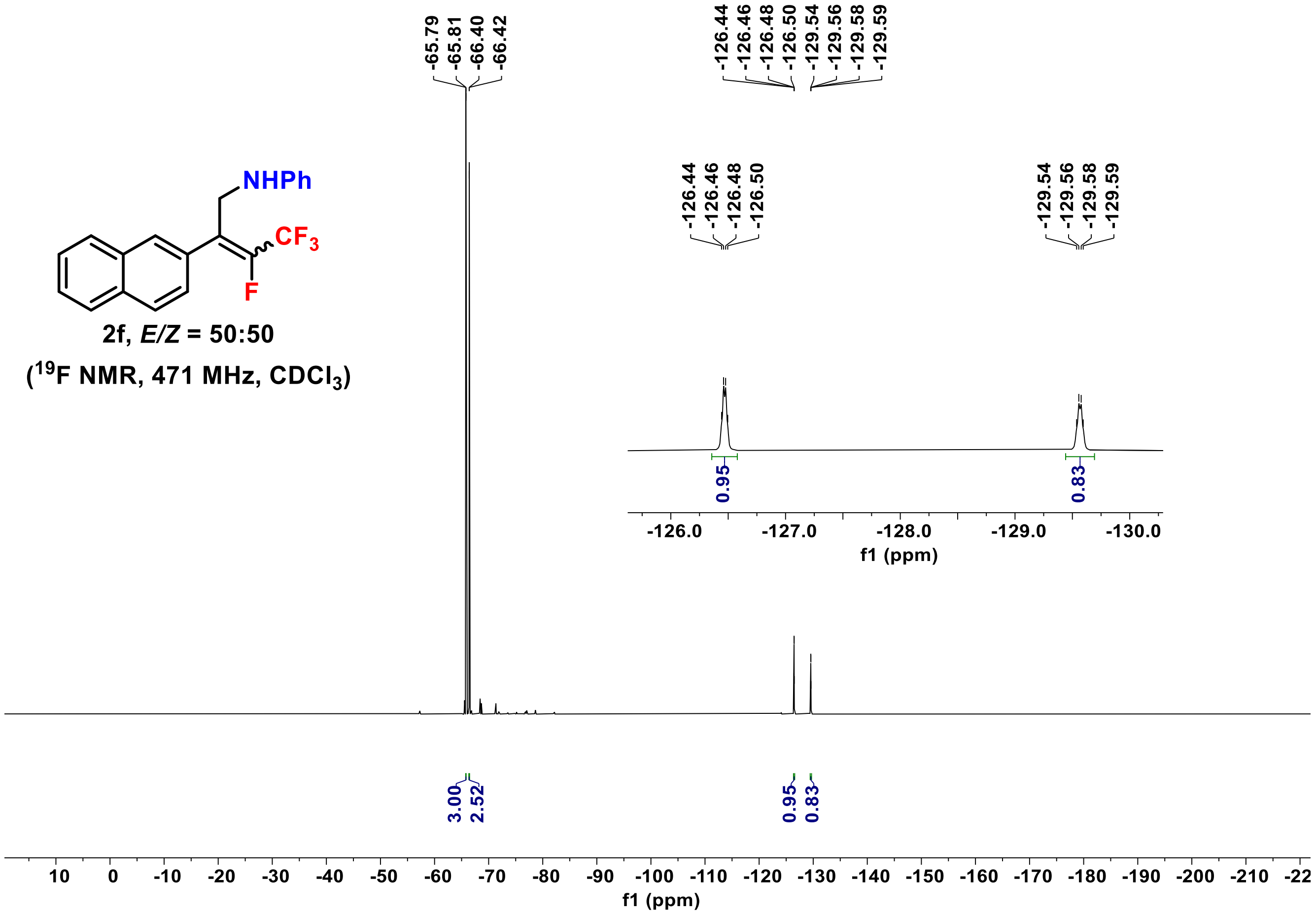

2f, *E/Z* = 50:50

(^{19}F NMR, 471 MHz, $CDCl_3$)

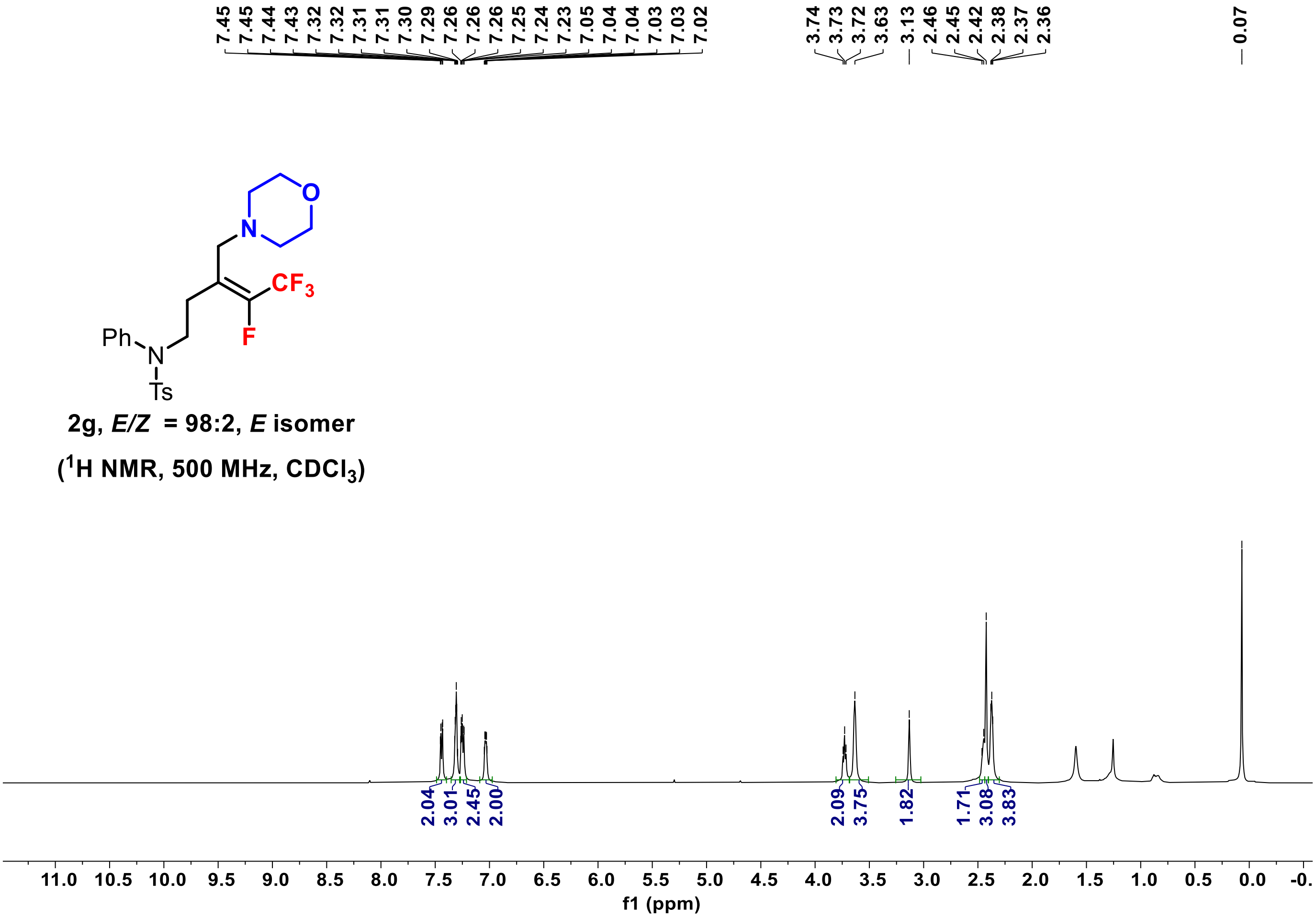

2g, *E/Z* = 98:2, *E* isomer

(^{1}H NMR, 500 MHz, $CDCl_3$)

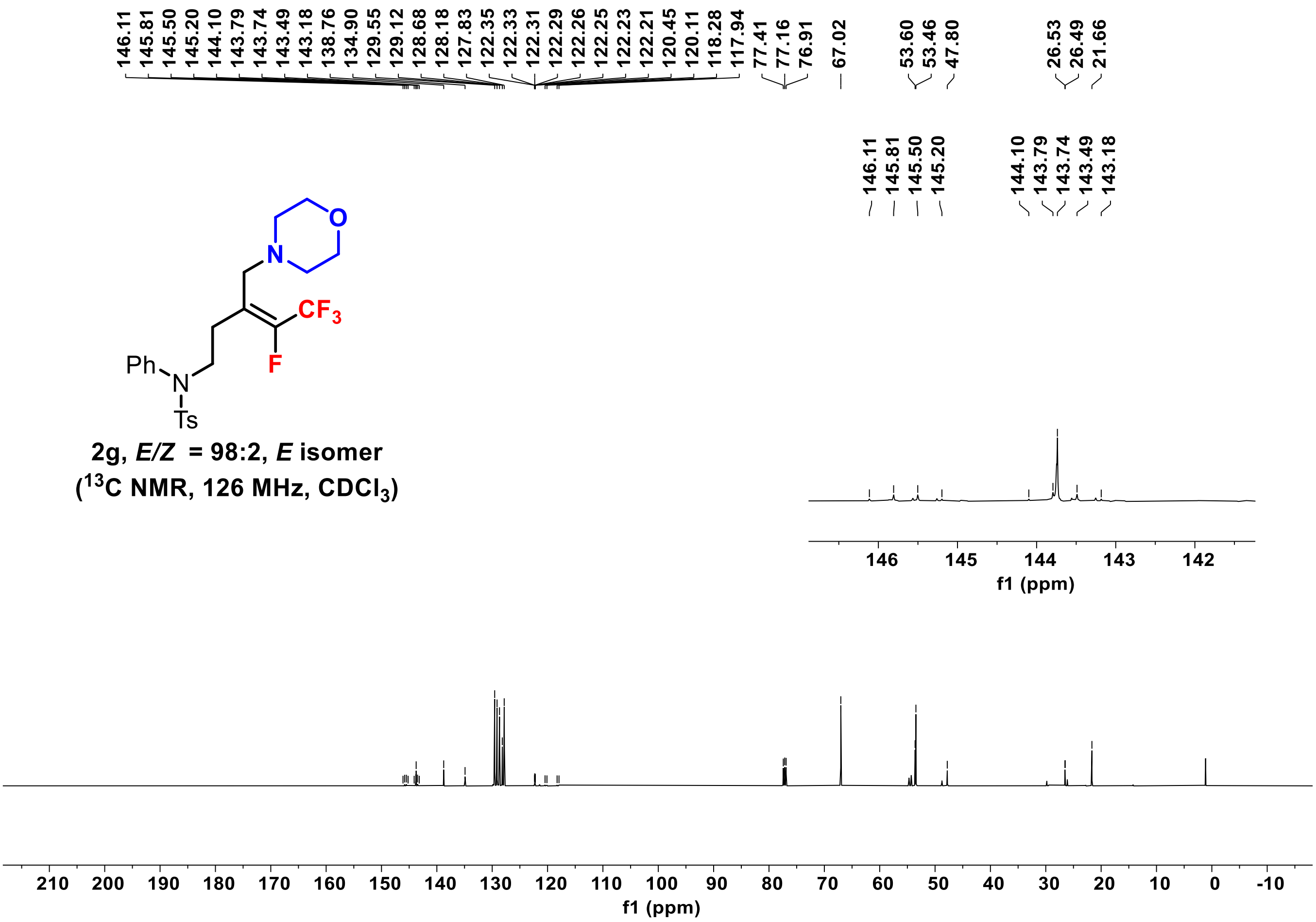

2g, *E/Z* = 98:2, *E* isomer

(^{13}C NMR, 126 MHz, $CDCl_3$)

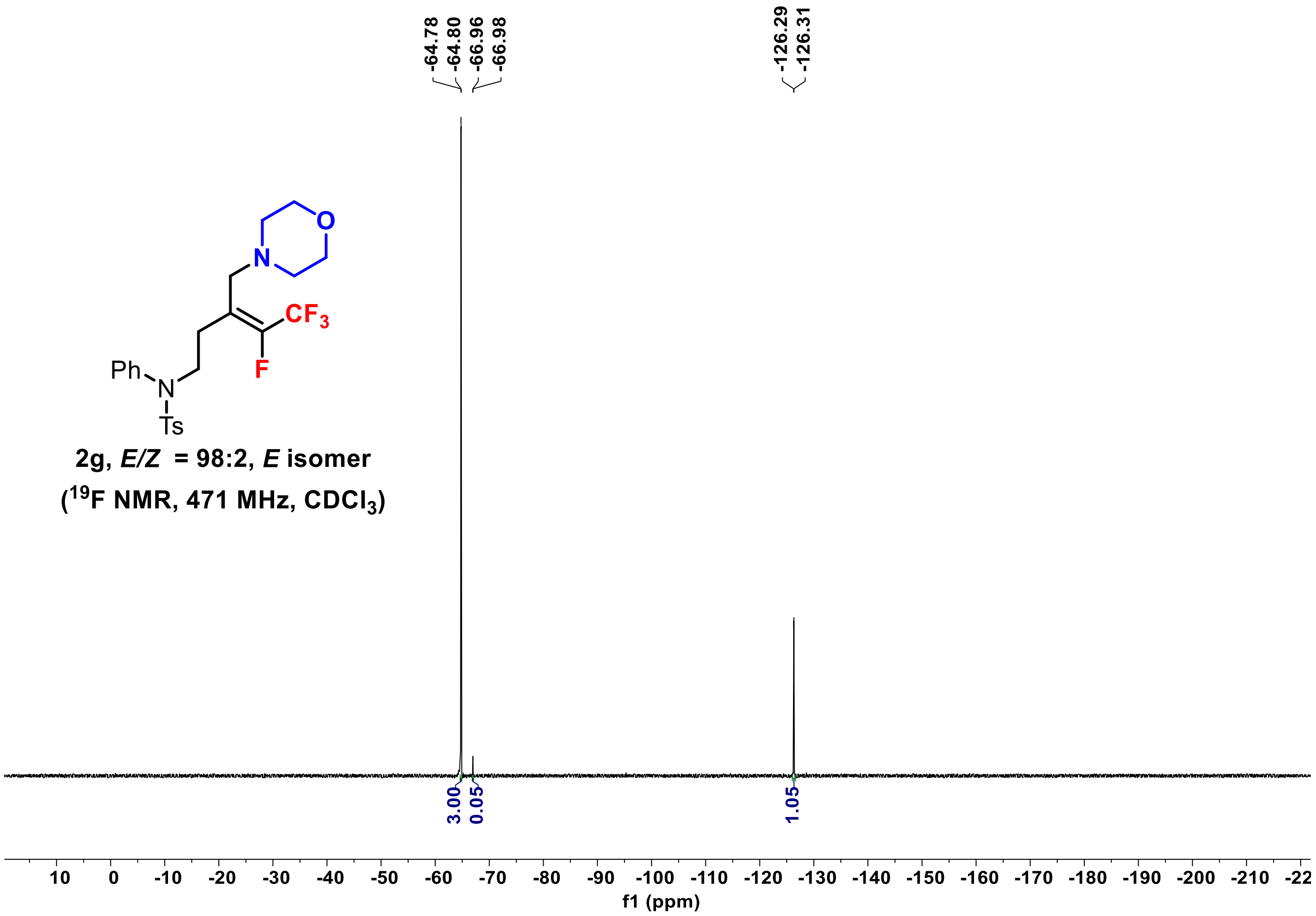

2g, *E/Z* = 98:2, *E* isomer

(^{19}F NMR, 471 MHz, $CDCl_3$)

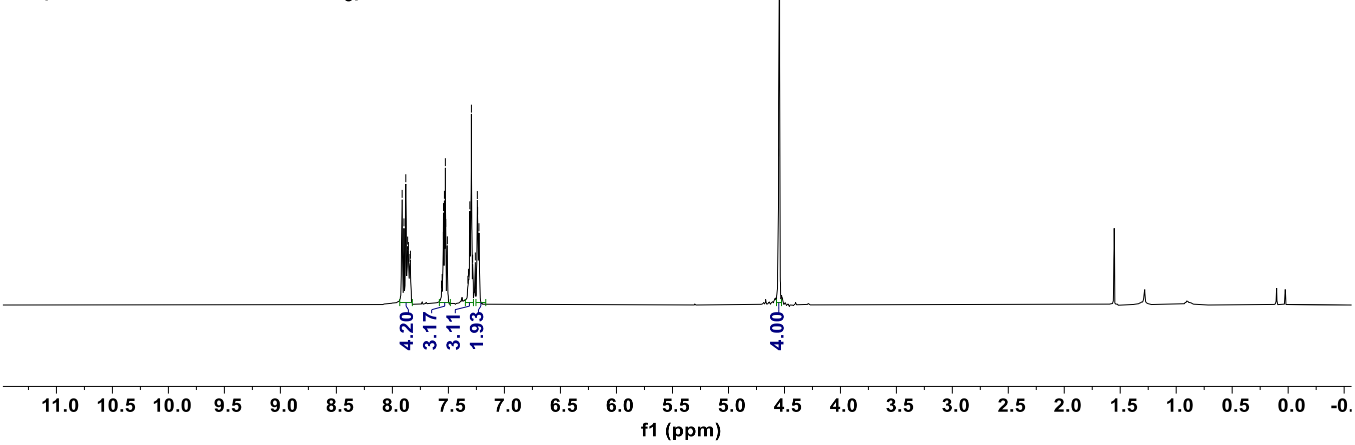

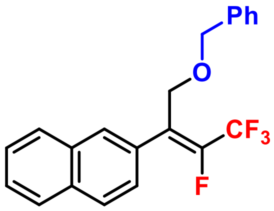

3a, *E/Z* >99:1, *E* isomer

(^{1}H NMR, 500 MHz, $CDCl_3$)

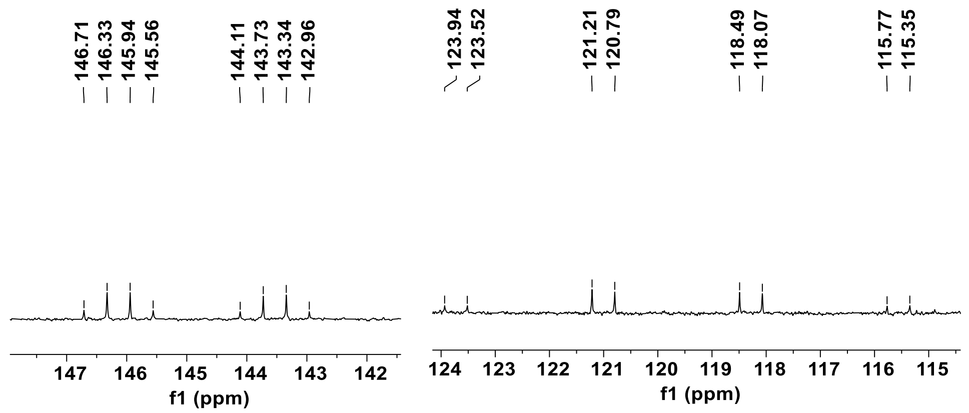

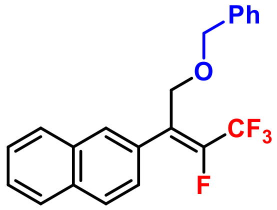

3a, *E/Z* >99:1, *E* isomer

(^{13}C NMR, 126 MHz, $CDCl_3$)

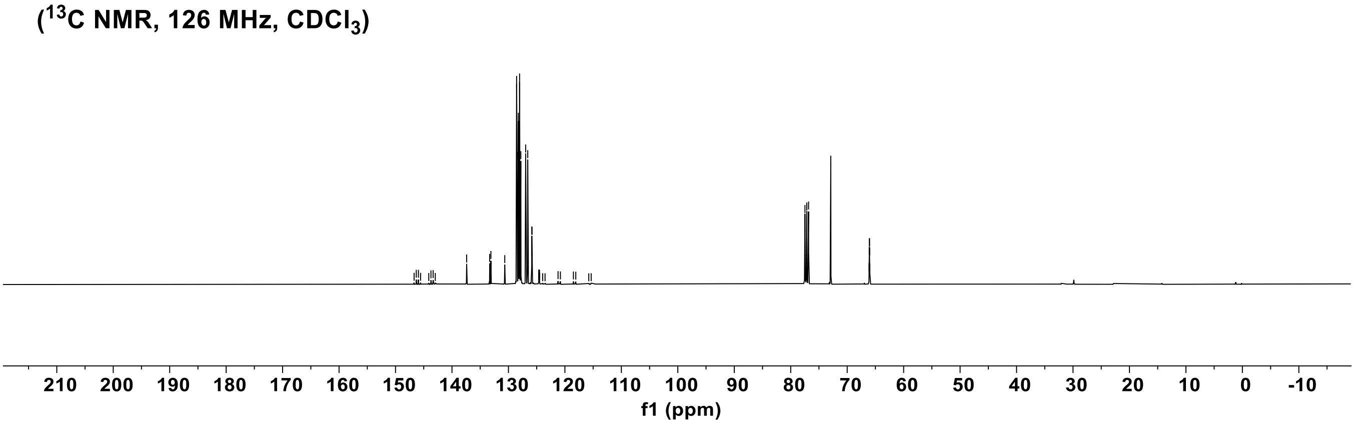

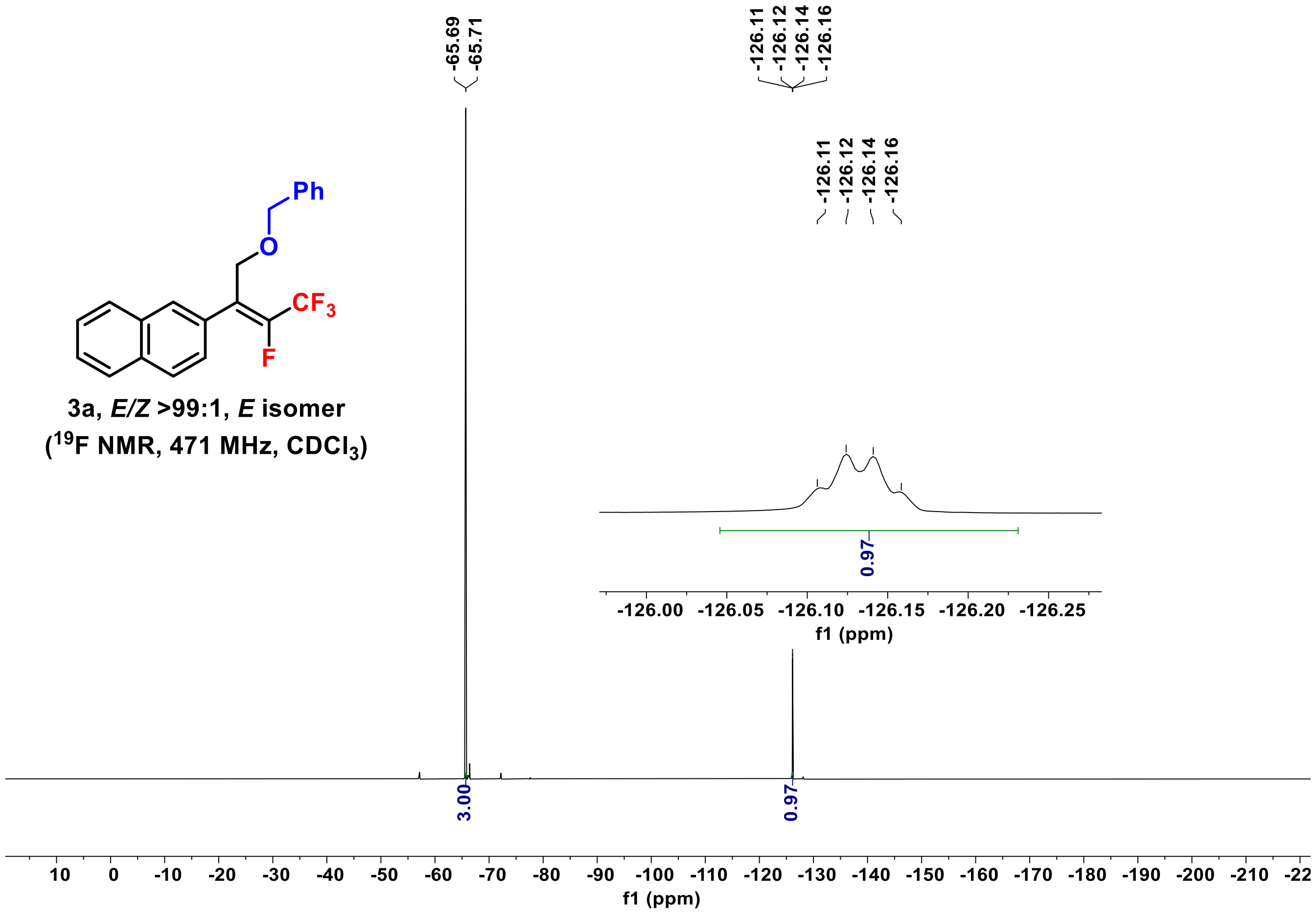

3a, *E/Z* >99:1, *E* isomer

(^{19}F NMR, 471 MHz, $CDCl_3$)

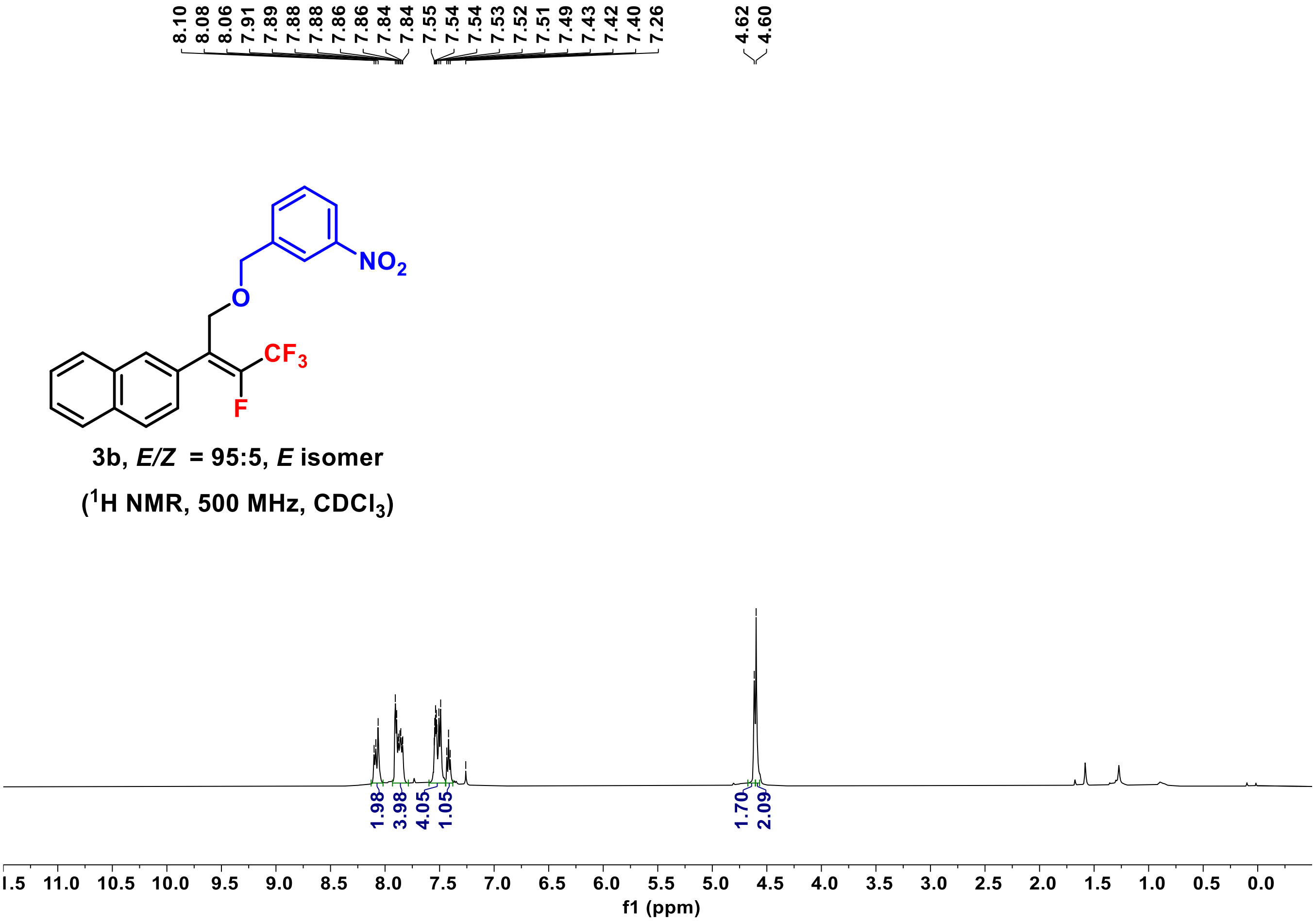

3b, *E/Z* = 95:5, *E* isomer

(^{1}H NMR, 500 MHz, $CDCl_3$)

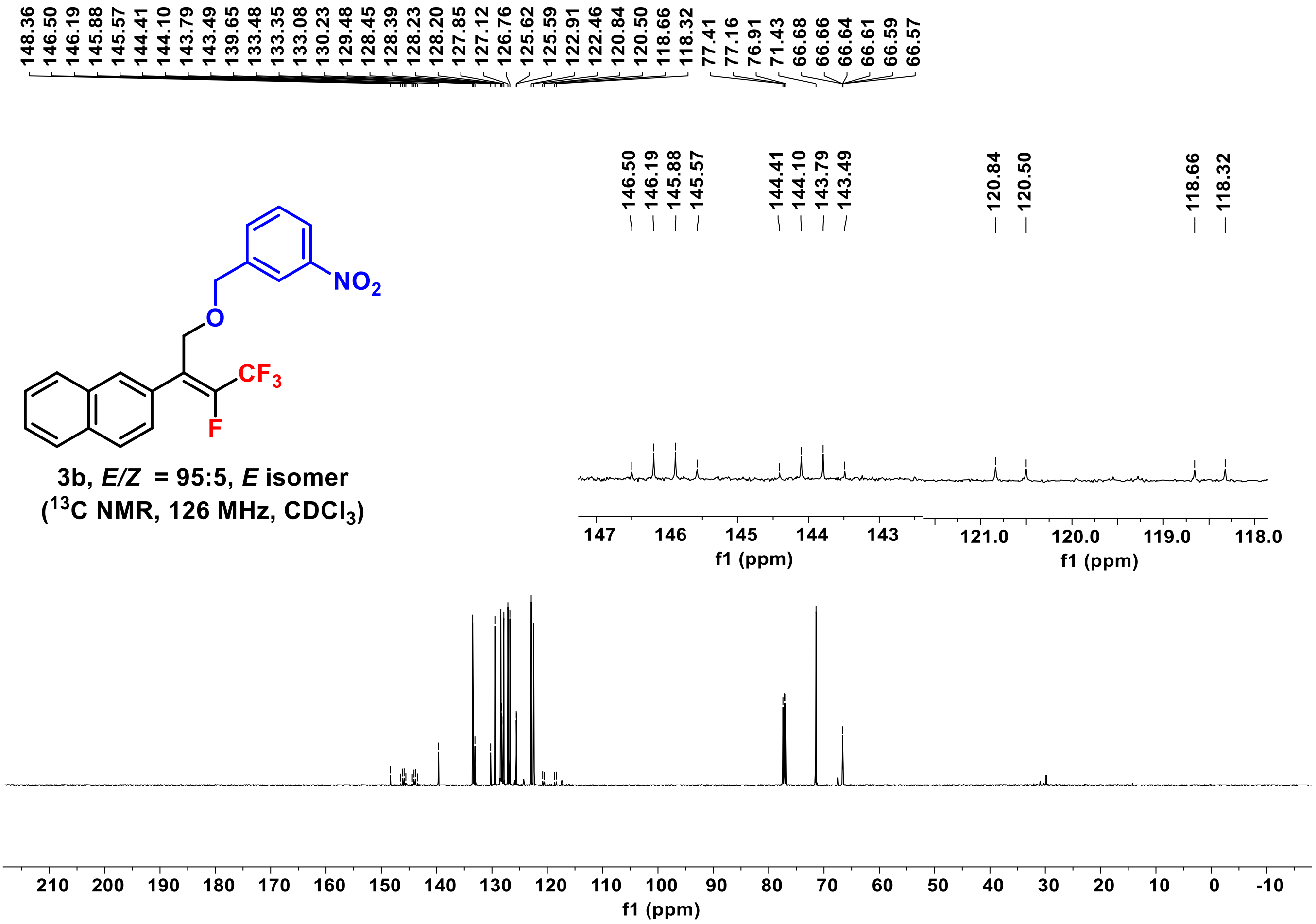

3b, *E/Z* = 95:5, *E* isomer
(^{13}C NMR, 126 MHz, $CDCl_3$)

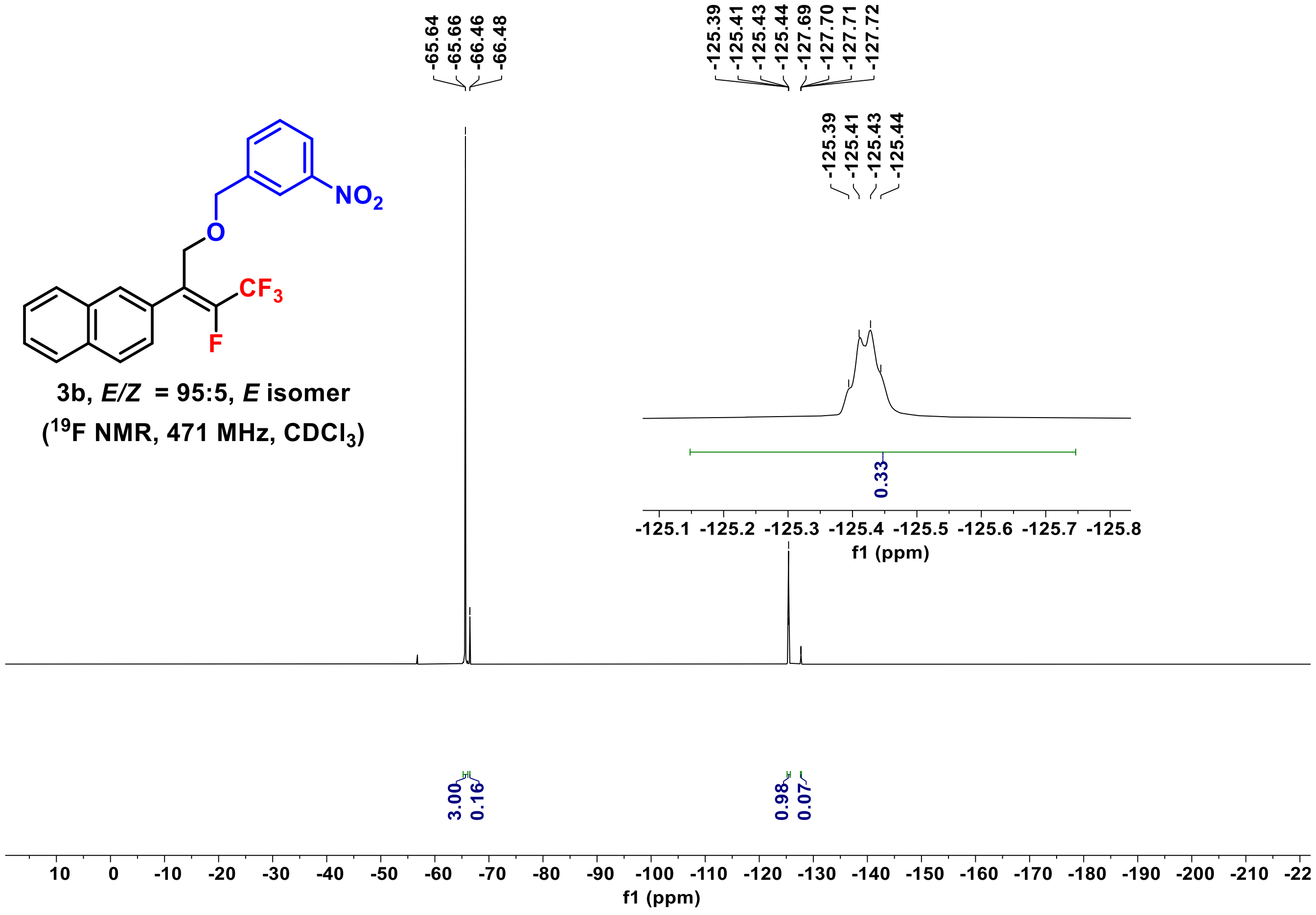

3b, *E/Z* = 95:5, *E* isomer
(^{19}F NMR, 471 MHz, $CDCl_3$)

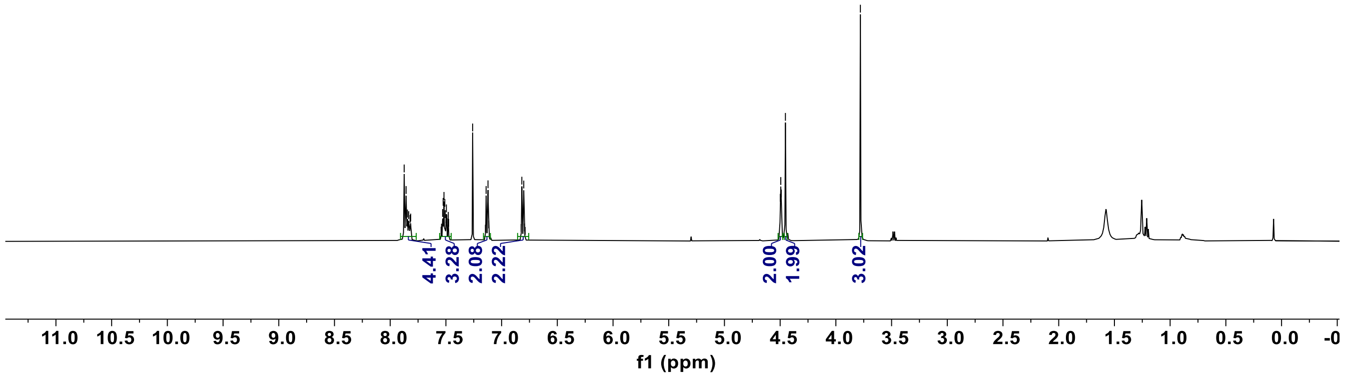

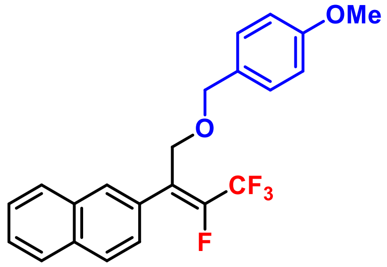

3c, *E/Z* = 93:7, *E* isomer

(^{1}H NMR, 500 MHz, $CDCl_3$)

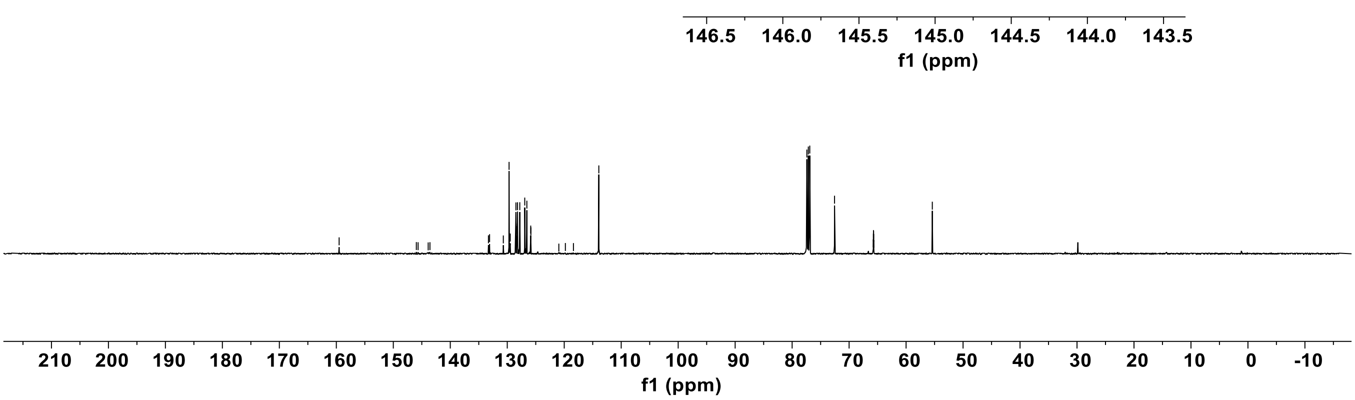

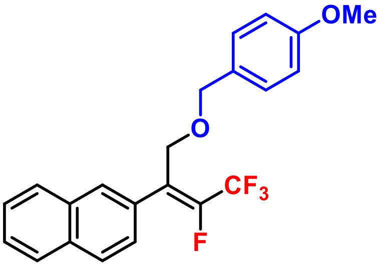

3c, *E/Z* = 93:7, *E* isomer

(^{13}C NMR, 126 MHz, $CDCl_3$)

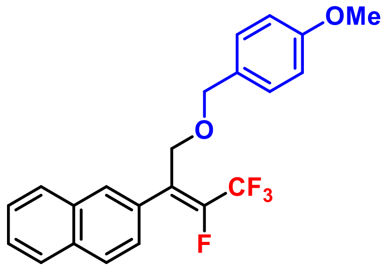

3c, *E/Z* = 93:7, *E* isomer

(^{19}F NMR, 471 MHz, $CDCl_3$)

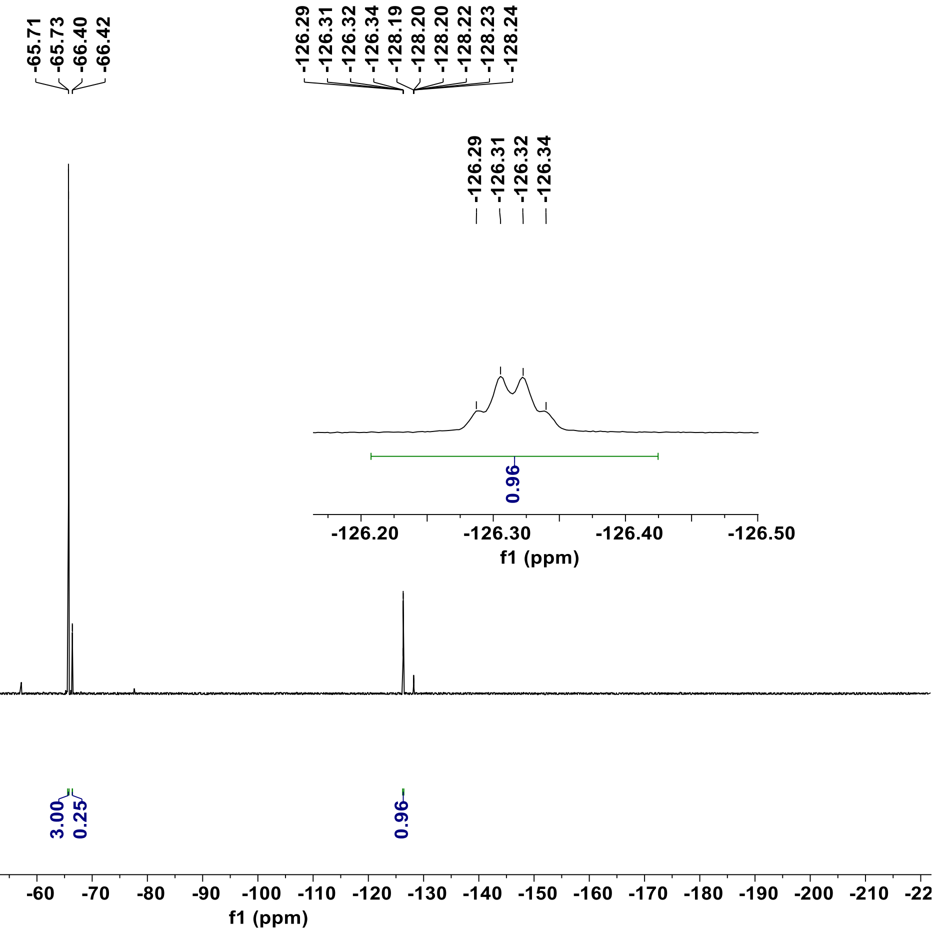

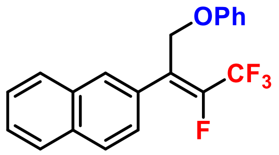

3d, *E/Z* = 60:40

(^{1}H NMR, 500 MHz, $CDCl_3$)

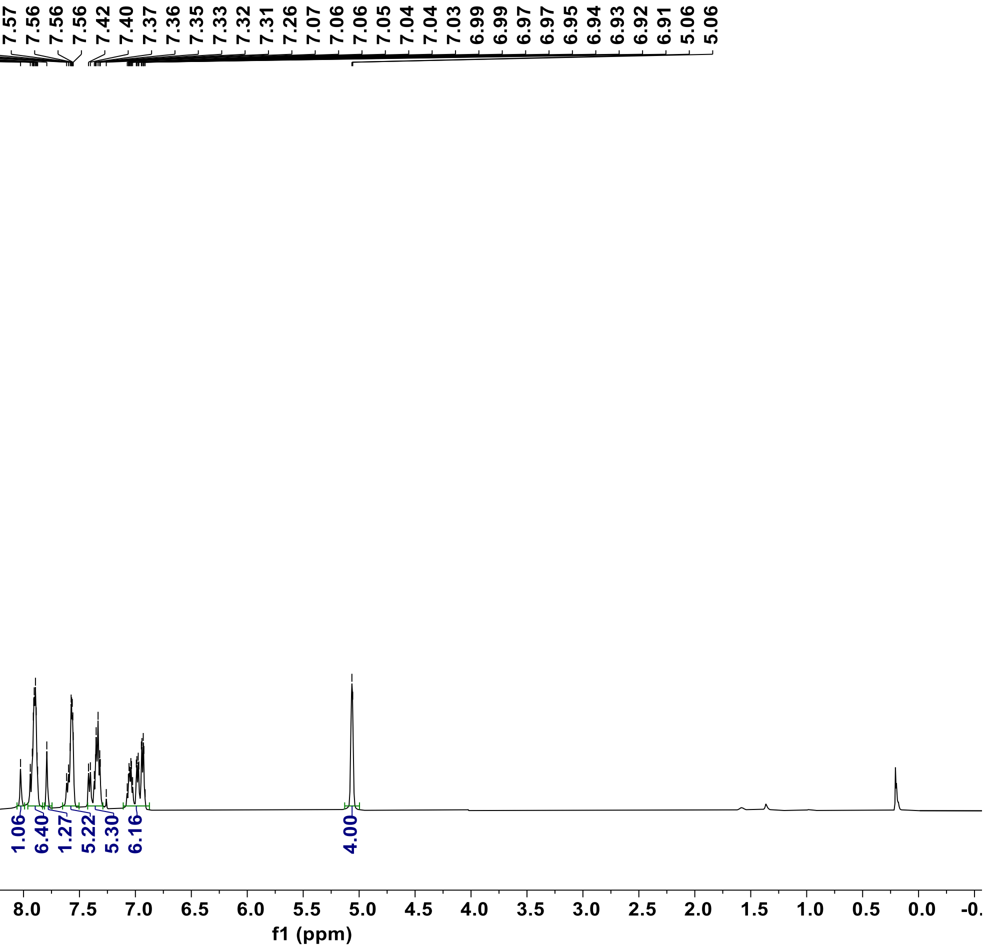

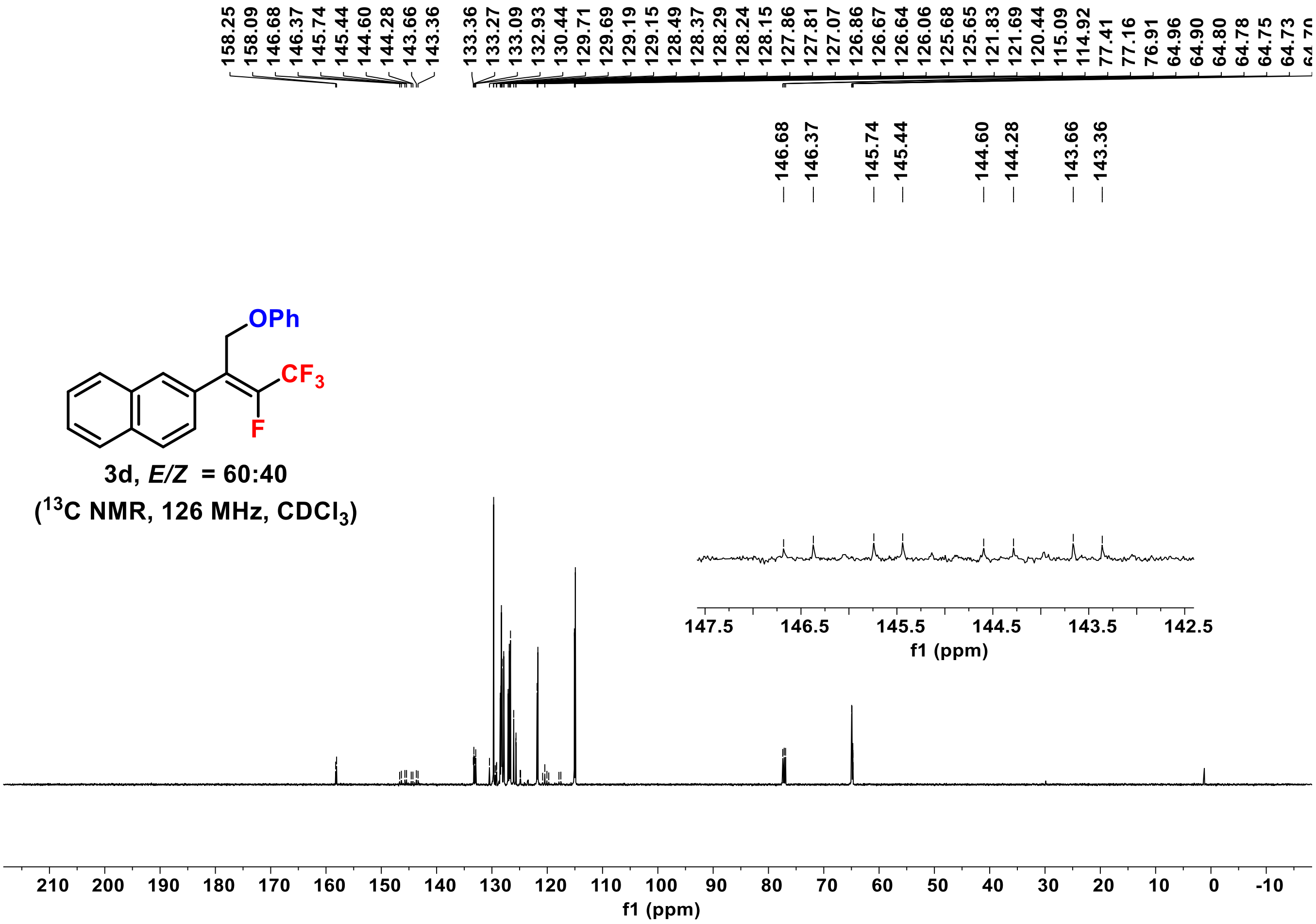
158.25
158.09
146.68
146.37
145.74
145.44
144.60
144.28
143.66
143.36
133.36
133.27
133.09
132.93
130.44
129.71
129.69
129.19
129.15
128.49
128.37
128.29
128.24
128.15
127.86
127.81
127.07
126.86
126.67
126.64
126.06
125.68
125.65
121.83
121.69
120.44
115.09
114.92
77.41
77.16
76.91
64.96
64.90
64.80
64.78
64.75
64.73
OPh
CF3
F
3d, E/Z = 60:40
(13C NMR, 126 MHz, CDCl3)
147.5
146.5
145.5
144.5
143.5
142.5
f1 (ppm)
210
200
190
180
170
160
150
140
130
120
110
100
90
80
70
60
50
40
30
20
10
0
-10
f1 (ppm)

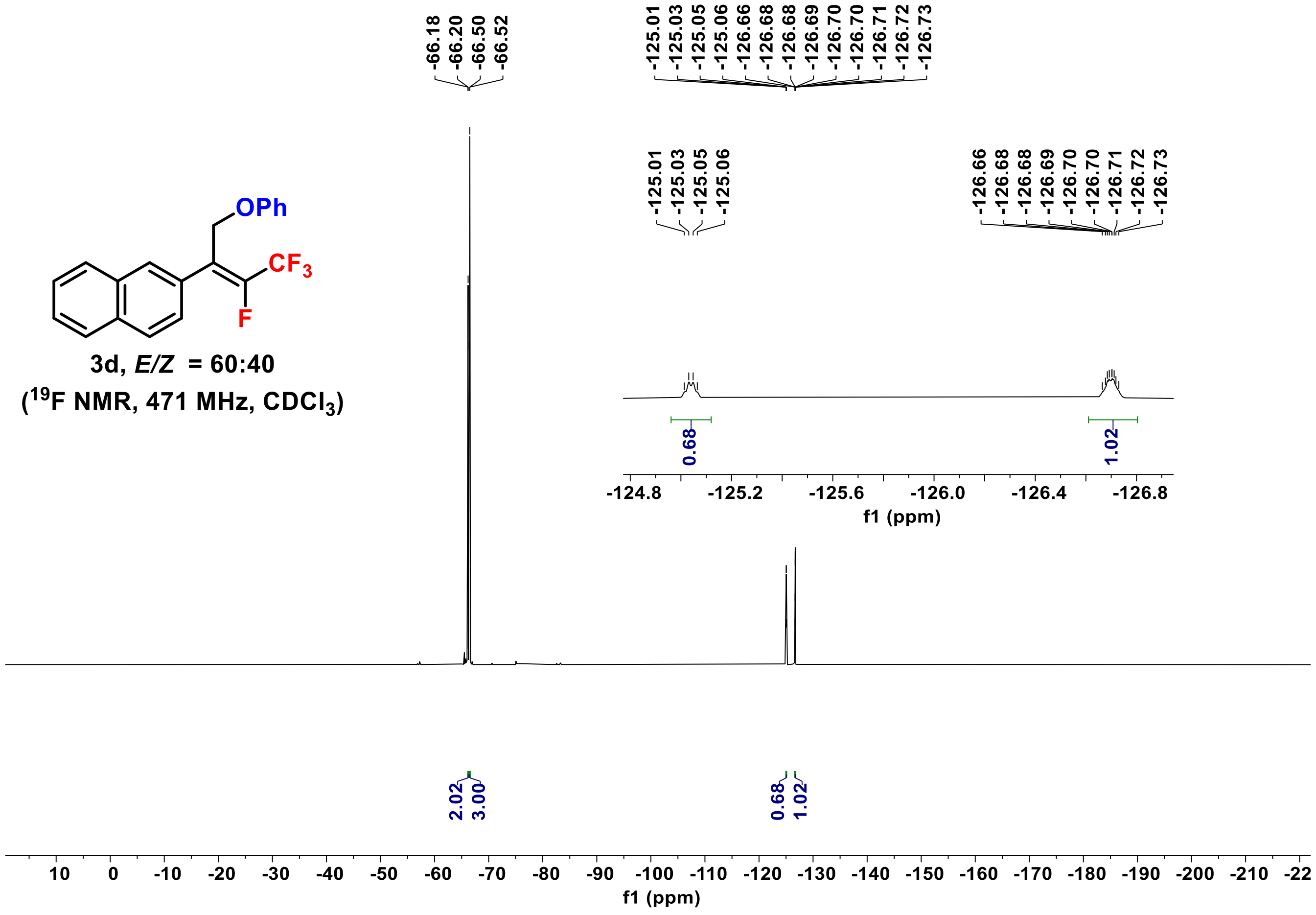
-66.18
-66.20
-66.50
-66.52
-125.01
-125.03
-125.05
-125.06
-126.66
-126.68
-126.68
-126.69
-126.70
-126.70
-126.71
-126.72
-126.73
OPh
CF3
F
3d, E/Z = 60:40
(19F NMR, 471 MHz, CDCl3)
0.68
1.02
-124.8
-125.2
-125.6
-126.0
-126.4
-126.8
f1 (ppm)
2.02
3.00
0.68
1.02
10
0
-10
-20
-30
-40
-50
-60
-70
-80
-90
-100
-110
-120
-130
-140
-150
-160
-170
-180
-190
-200
-210
-22
f1 (ppm)

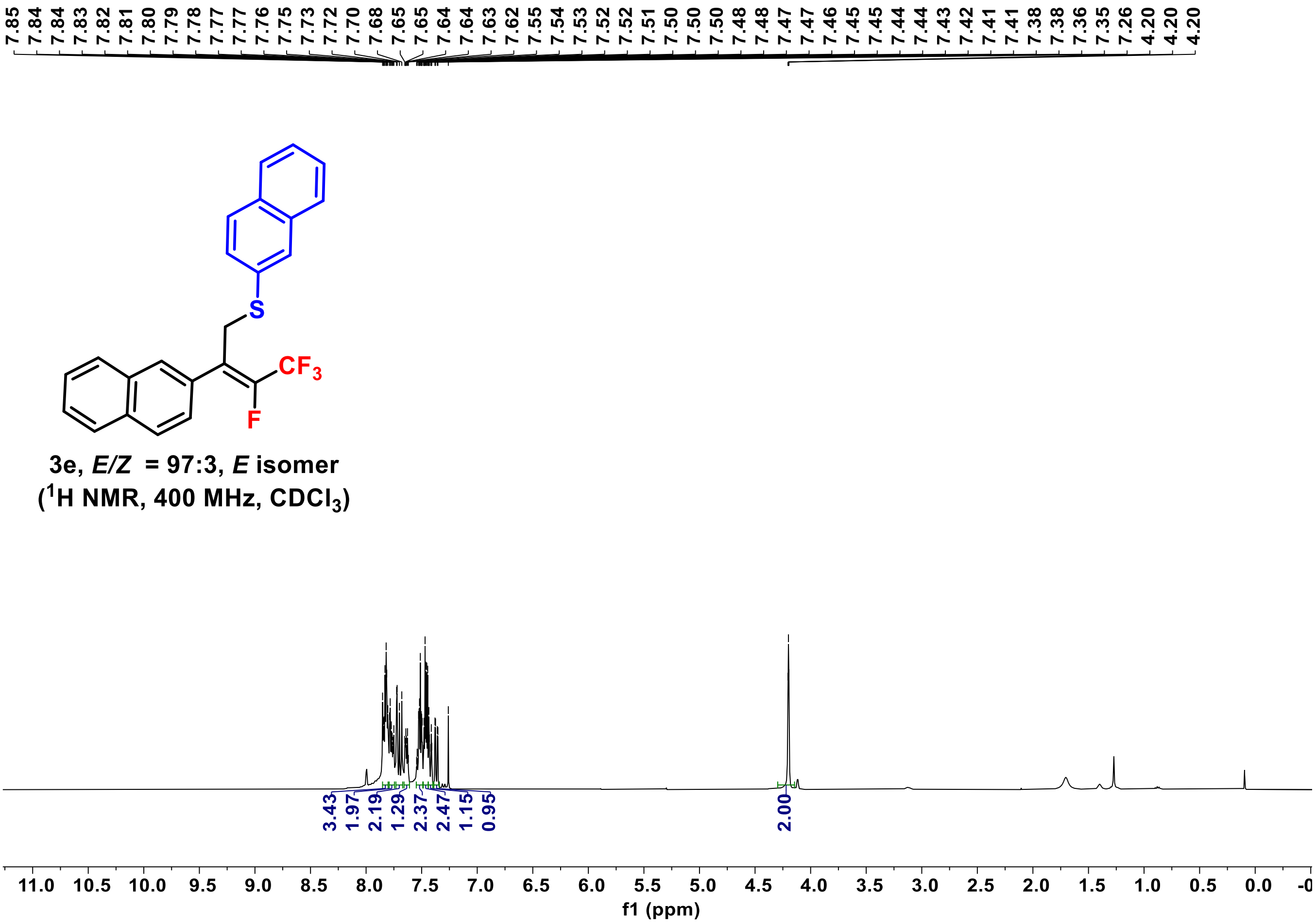

3e, *E/Z* = 97:3, *E* isomer
(^{1}H NMR, 400 MHz, $CDCl_3$)

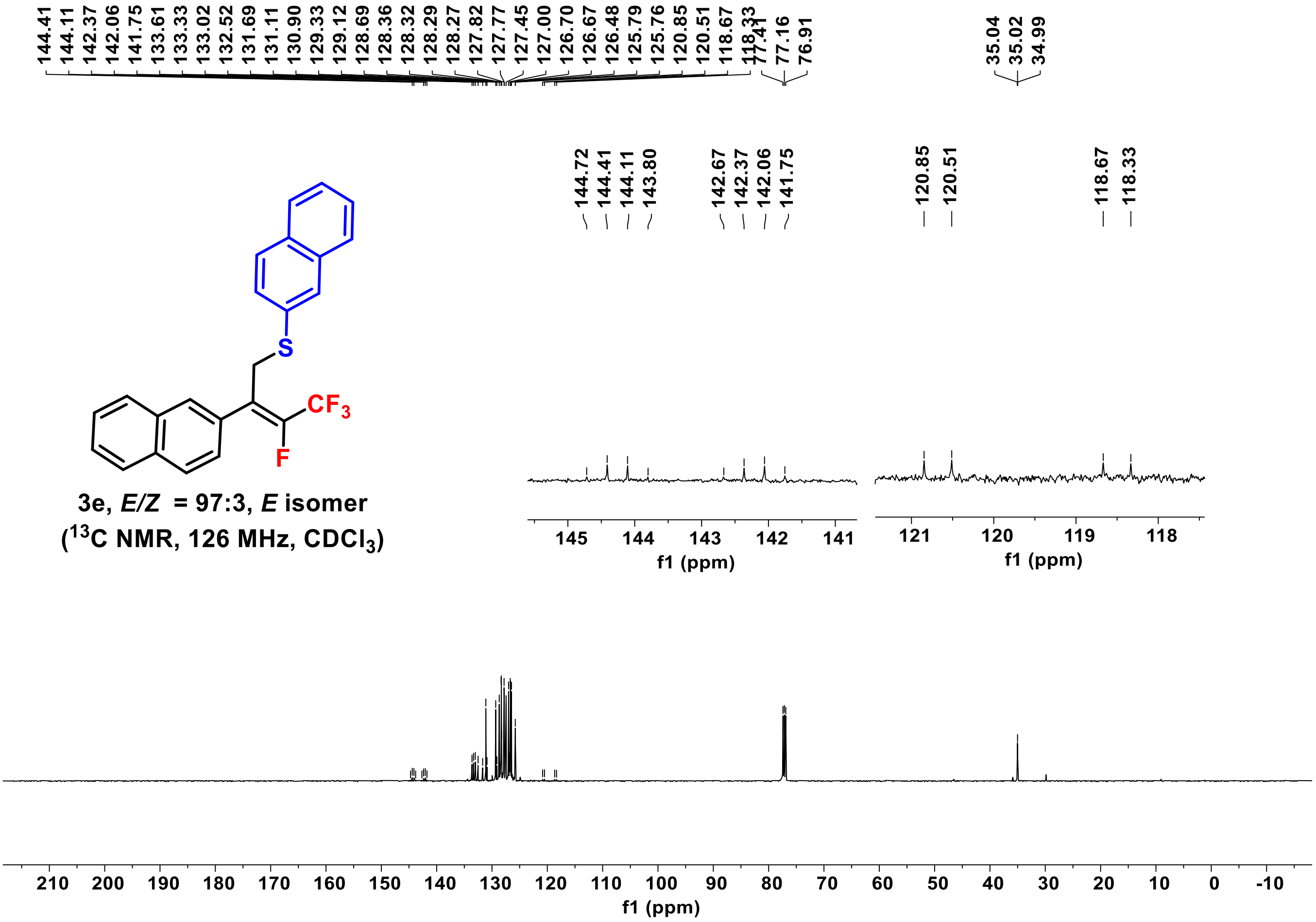

3e, *E/Z* = 97:3, *E* isomer
(^{13}C NMR, 126 MHz, $CDCl_3$)

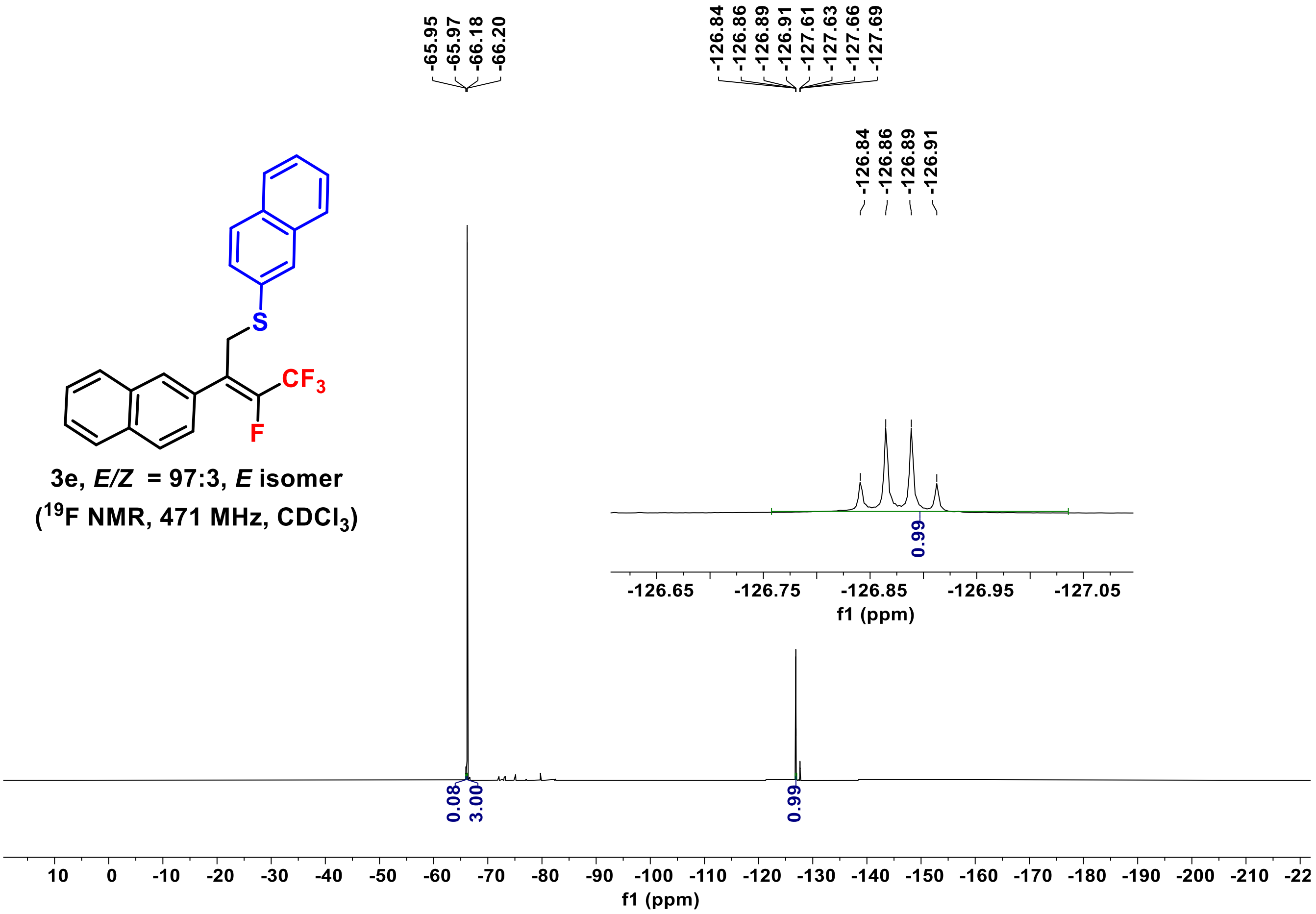

3e, *E/Z* = 97:3, *E* isomer
(^{19}F NMR, 471 MHz, $CDCl_3$)

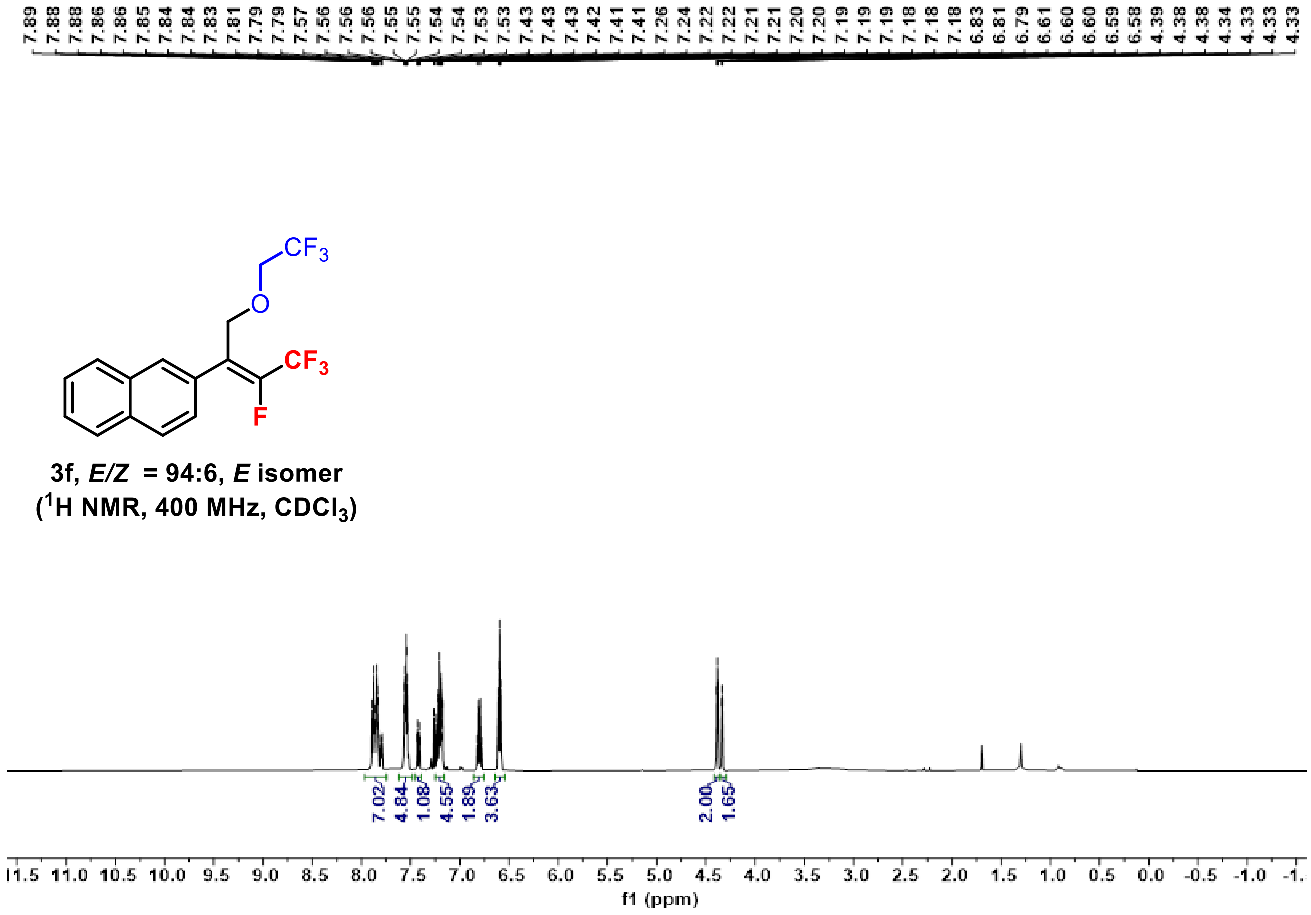

3f, *E/Z* = 94:6, *E* isomer
(^{1}H NMR, 400 MHz, $CDCl_3$)

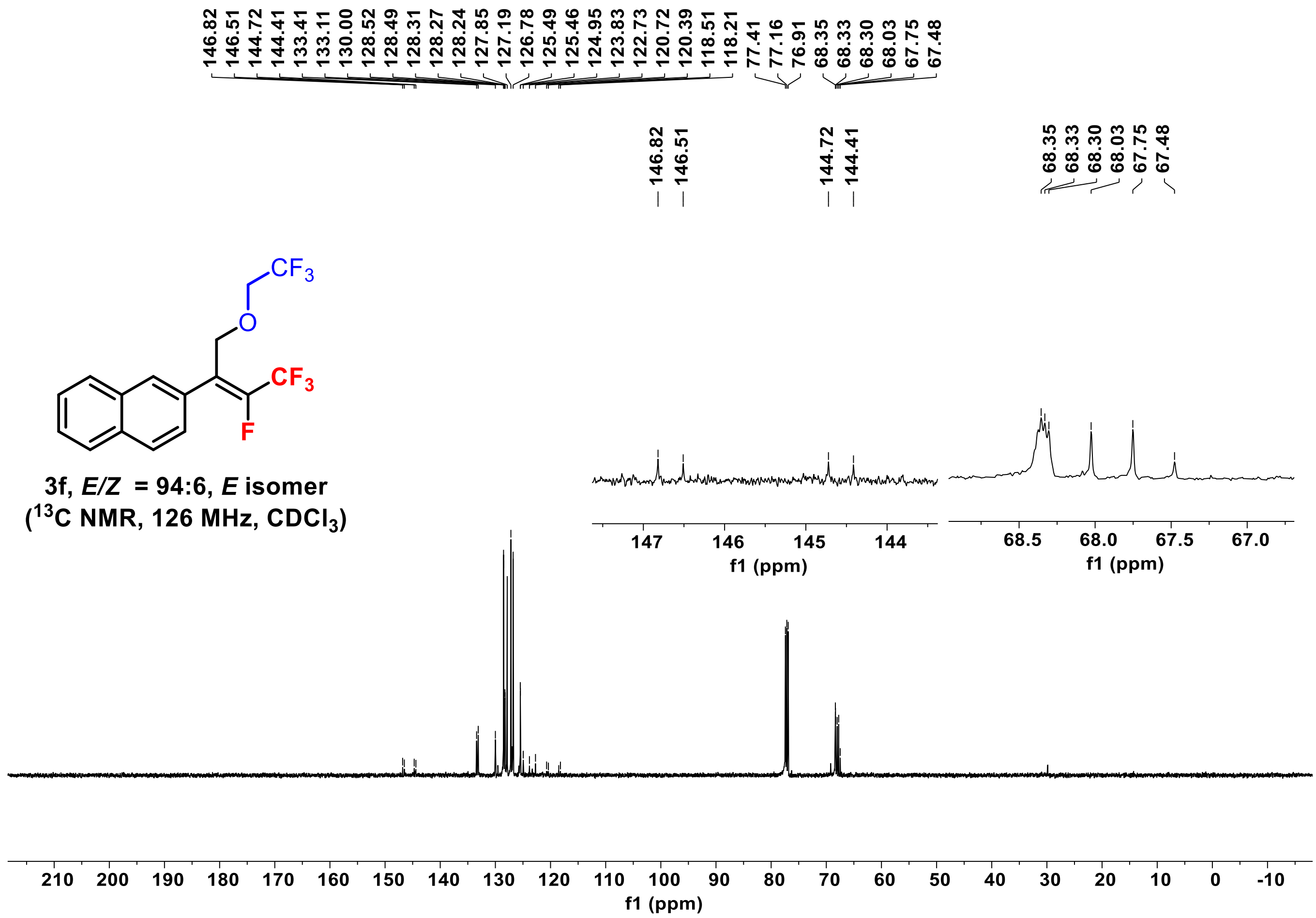
CF3
O
CF3
F
3f, E/Z = 94:6, E isomer
(13C NMR, 126 MHz, CDCl3)
146.82
146.51
144.72
144.41
133.41
133.11
130.00
128.52
128.49
128.31
128.27
128.24
127.85
127.19
126.78
125.49
125.46
124.95
123.83
122.73
120.72
120.39
118.51
118.21
77.41
77.16
76.91
68.35
68.33
68.30
68.03
67.75
67.48
147
146
145
144
f1 (ppm)
68.5
68.0
67.5
67.0
f1 (ppm)
210 200 190 180 170 160 150 140 130 120 110 100 90 80 70 60 50 40 30 20 10 0 -10
f1 (ppm)

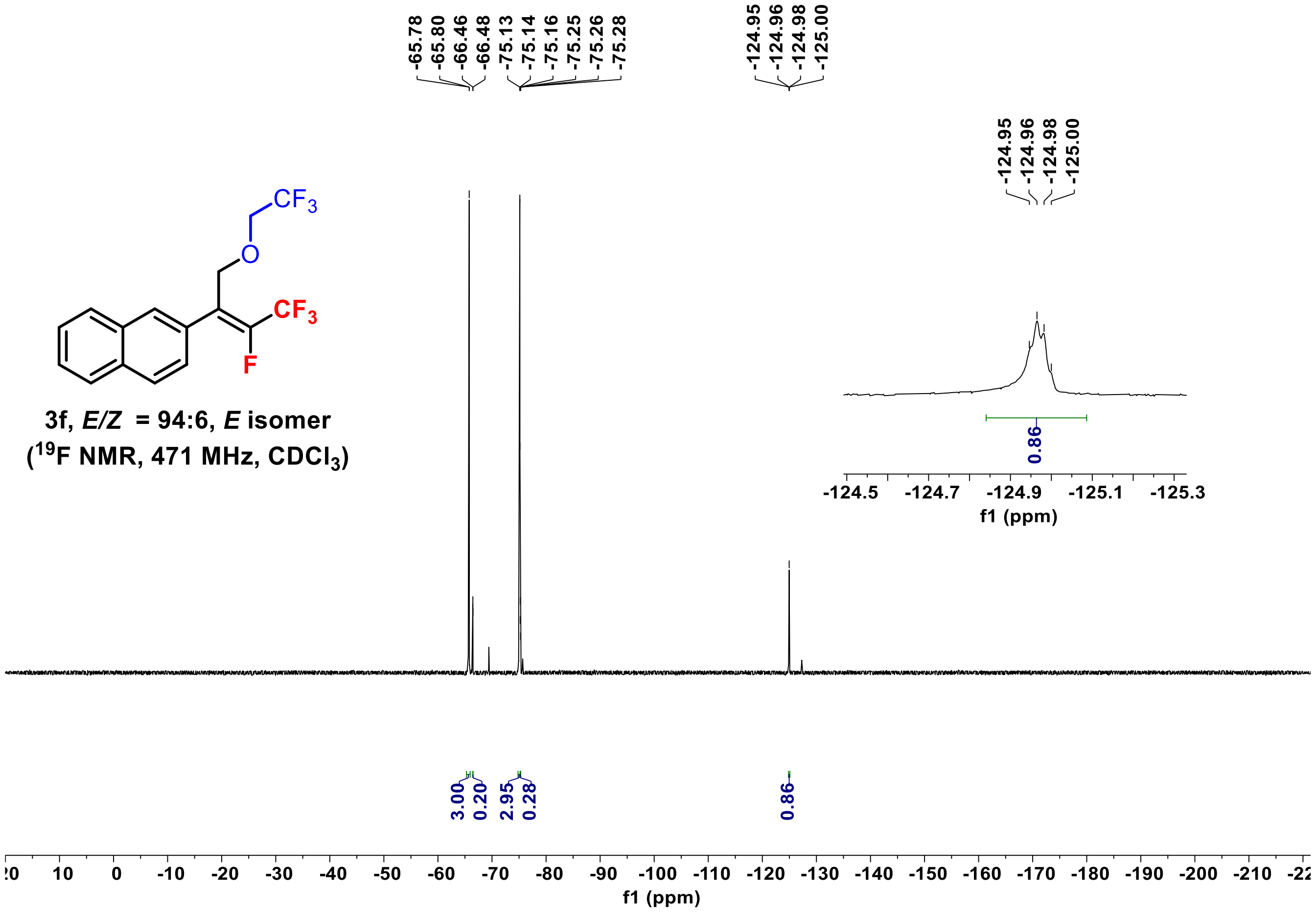
CF3
O
CF3
F
3f, E/Z = 94:6, E isomer
(19F NMR, 471 MHz, CDCl3)
-65.78
-65.80
-66.46
-66.48
-75.13
-75.14
-75.16
-75.25
-75.26
-75.28
-124.95
-124.96
-124.98
-125.00
0.86
-124.5 -124.7 -124.9 -125.1 -125.3
f1 (ppm)
3.00
0.20
2.95
0.28
0.86
f1 (ppm)

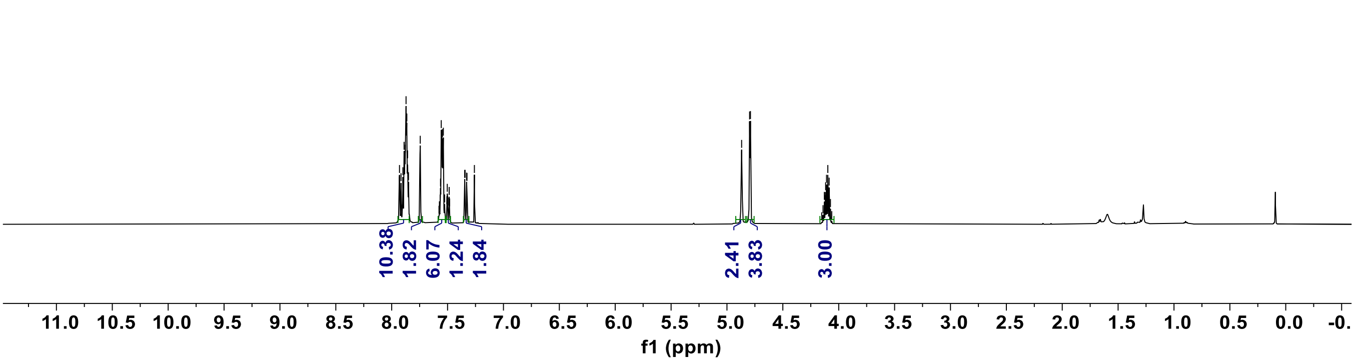

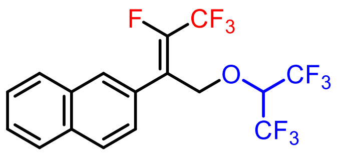

3g, *E/Z* = 63:37

(^{1}H NMR, 500 MHz, CDCl$_3$)

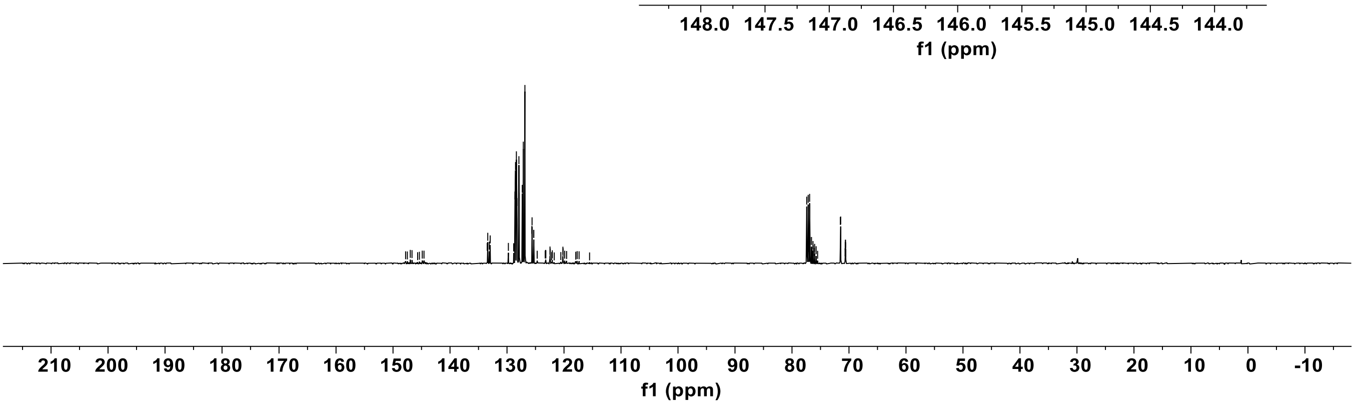

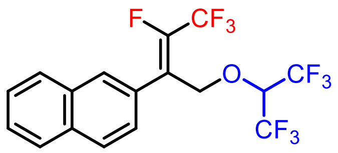

3g, *E/Z* = 63:37

(^{13}C NMR, 126 MHz, CDCl$_3$)

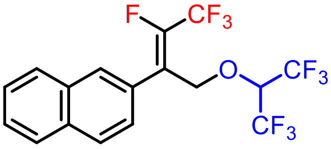

3g, *E/Z* = 63:37

(^{19}F NMR, 471 MHz, CDCl$_3$)

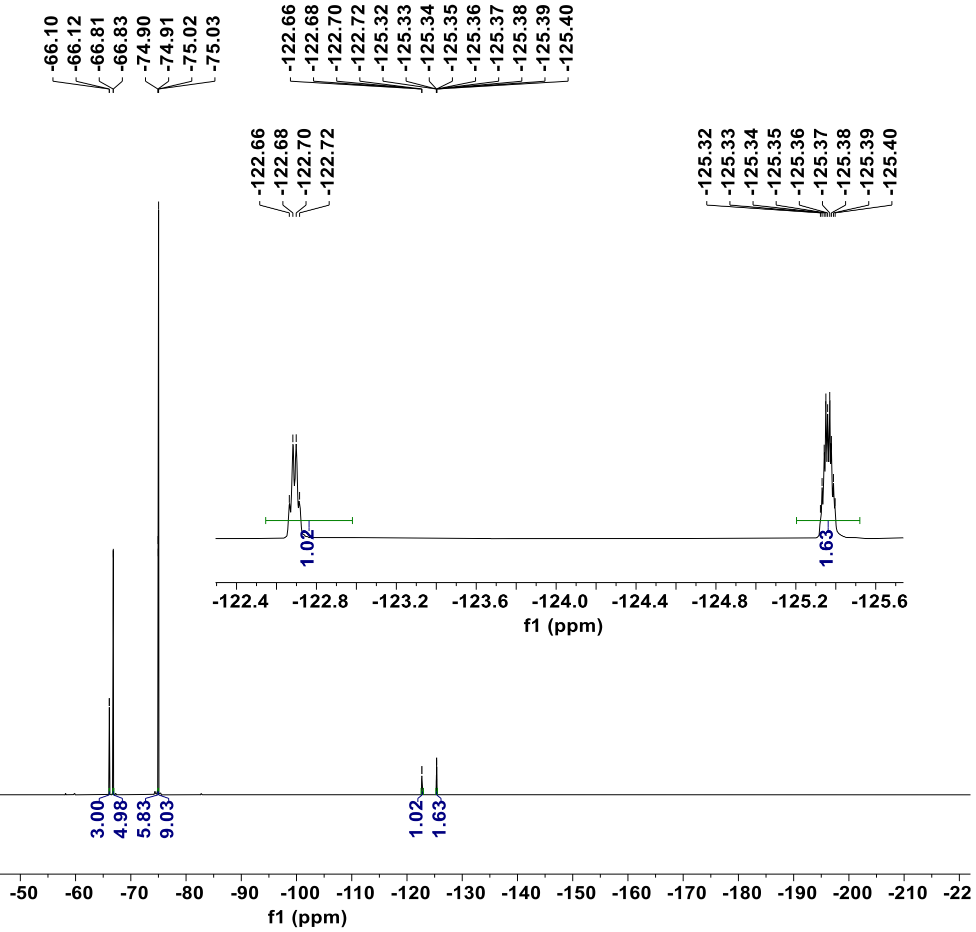

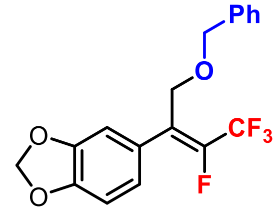

3h, *E/Z* = 93:7, *E* isomer

(^{1}H NMR, 500 MHz, CDCl$_3$)

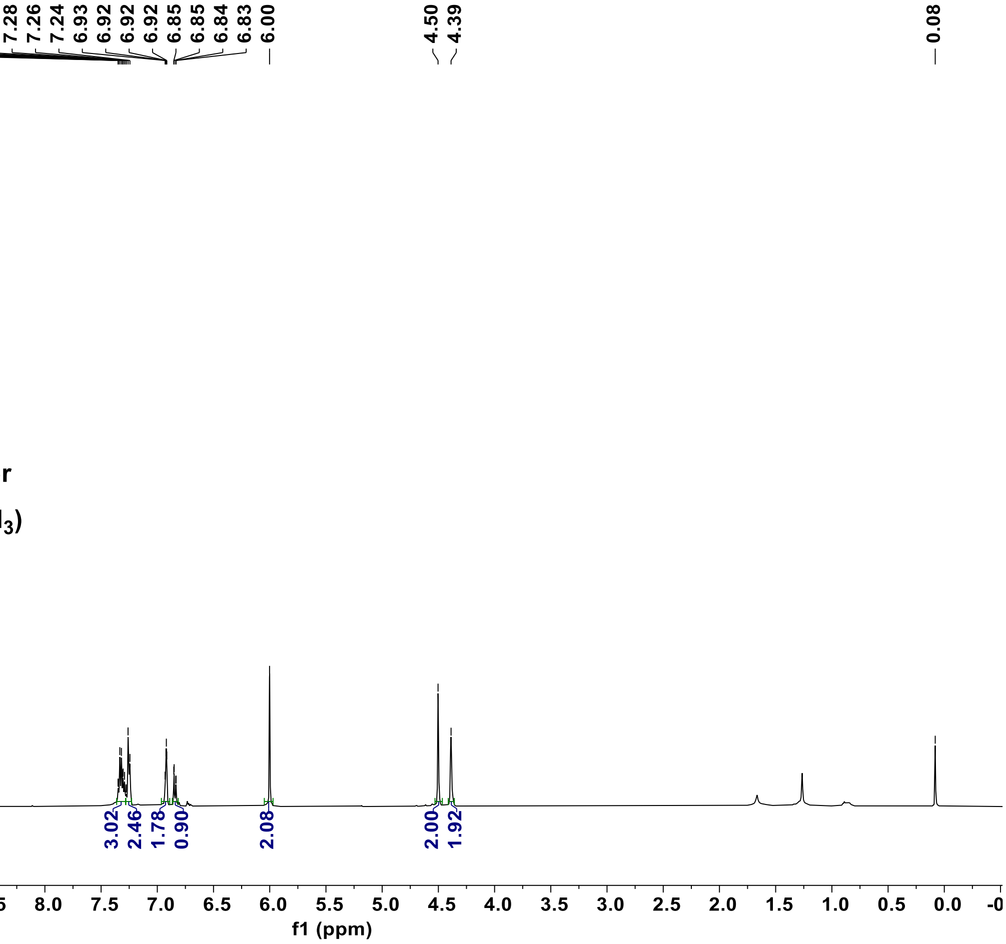

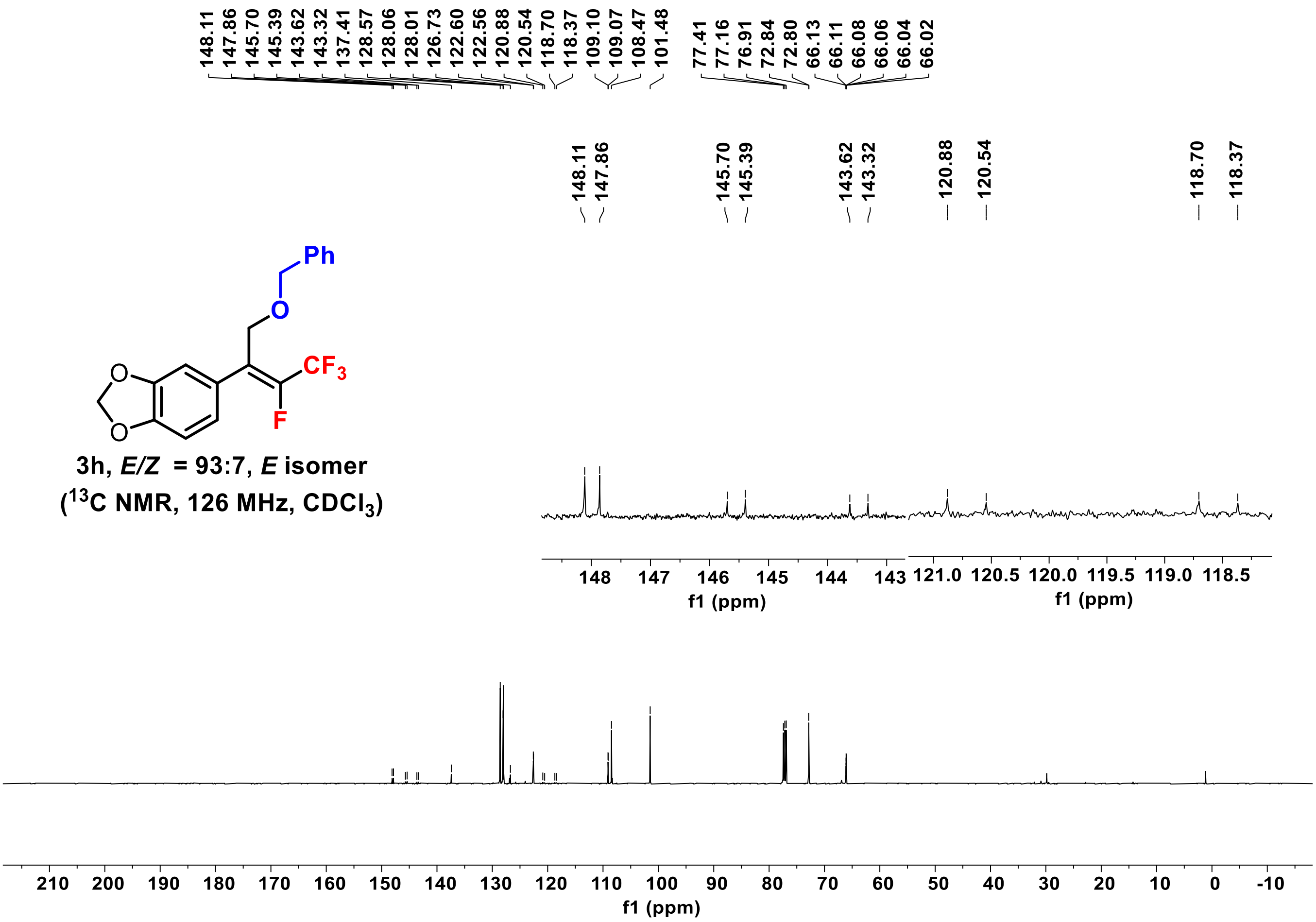
148.11 147.86 145.70 145.39 143.62 143.32 137.41 128.57 128.06 128.01 126.73 122.60 122.56 120.88 120.54 118.70 118.37 109.10 109.07 108.47 101.48 77.41 77.16 76.91 72.84 72.80 66.13 66.11 66.08 66.06 66.04 66.02
148.11 147.86 145.70 145.39 143.62 143.32 120.88 120.54 118.70 118.37
Ph
O
CF3
O
O
F
3h, E/Z = 93:7, E isomer
(13C NMR, 126 MHz, CDCl3)
148 147 146 145 144 143
f1 (ppm)
121.0 120.5 120.0 119.5 119.0 118.5
f1 (ppm)
210 200 190 180 170 160 150 140 130 120 110 100 90 80 70 60 50 40 30 20 10 0 -10
f1 (ppm)

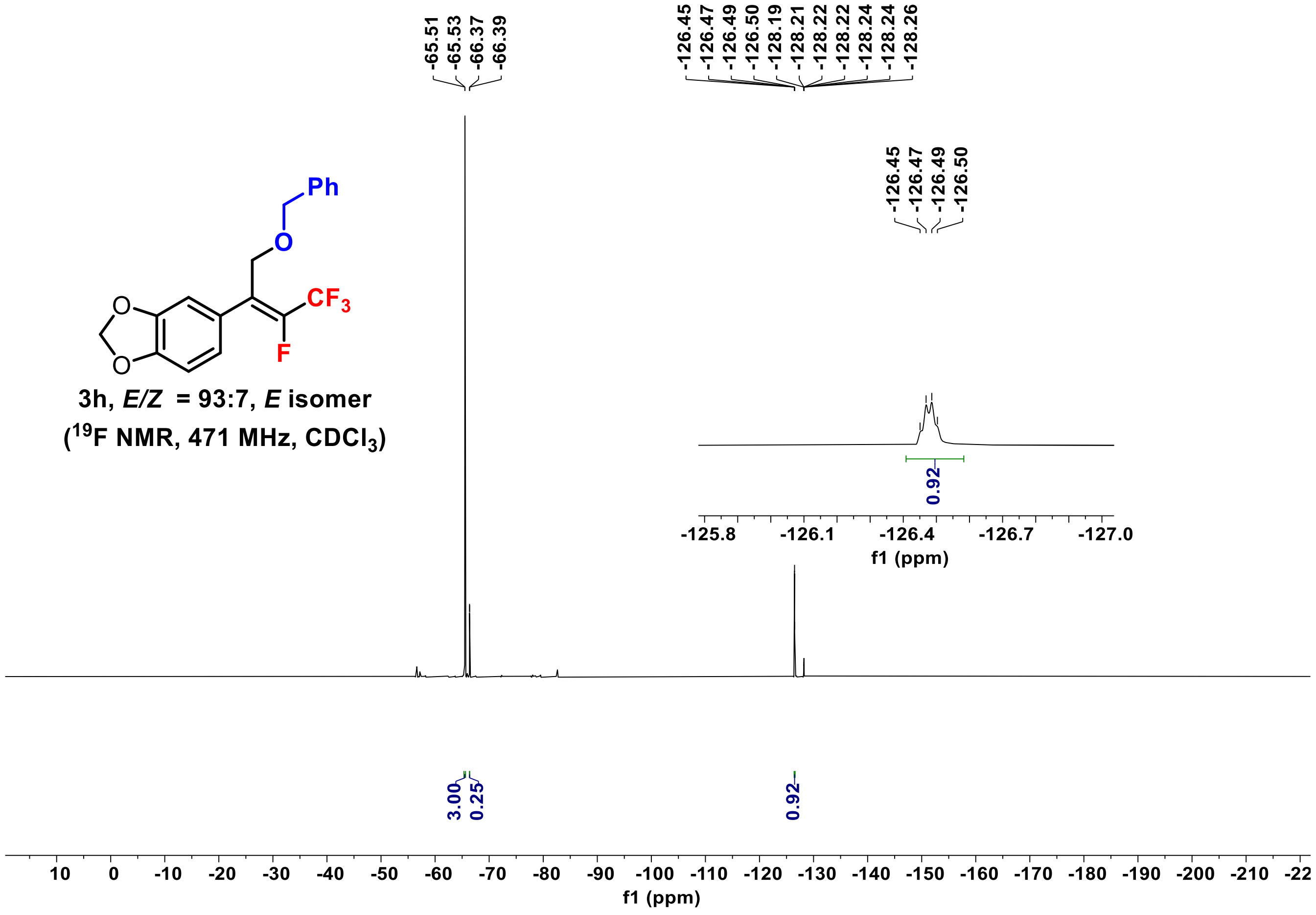
-65.51 -65.53 -66.37 -66.39
-126.45 -126.47 -126.49 -126.50 -128.19 -128.21 -128.22 -128.22 -128.24 -128.24 -128.26
-126.45 -126.47 -126.49 -126.50
Ph
O
CF3
O
O
F
3h, E/Z = 93:7, E isomer
(19F NMR, 471 MHz, CDCl3)
0.92
-125.8 -126.1 -126.4 -126.7 -127.0
f1 (ppm)
3.00 0.25 0.92
10 0 -10 -20 -30 -40 -50 -60 -70 -80 -90 -100 -110 -120 -130 -140 -150 -160 -170 -180 -190 -200 -210 -22
f1 (ppm)

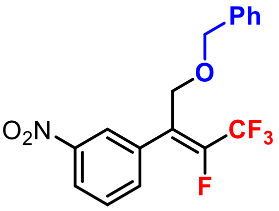

3i, *E/Z* = 97:3, *E* isomer
(^{1}H NMR, 400 MHz, $CDCl_3$)

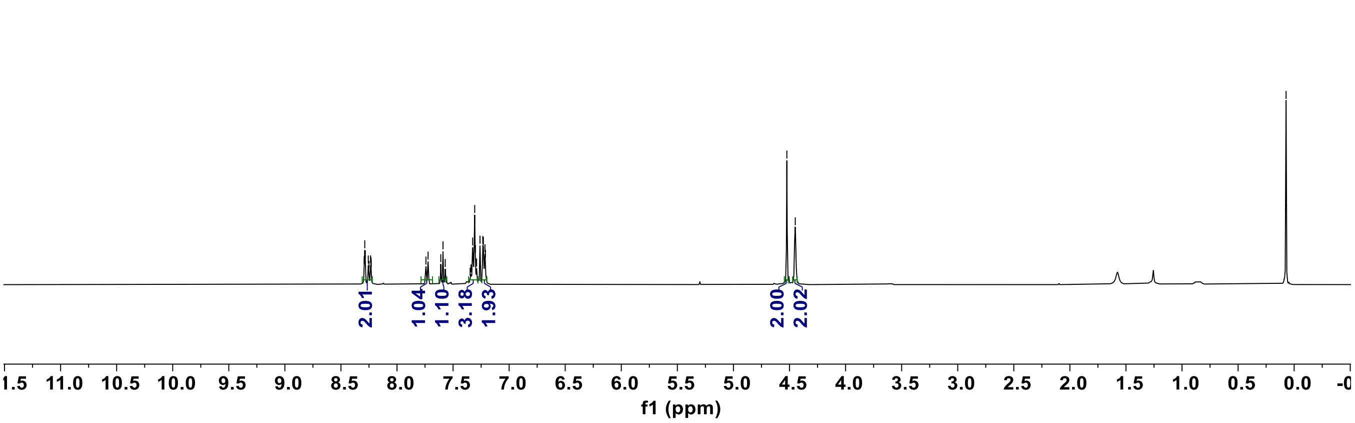

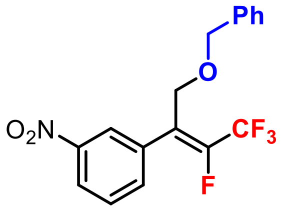

3i, *E/Z* = 97:3, *E* isomer
(^{13}C NMR, 101 MHz, $CDCl_3$)

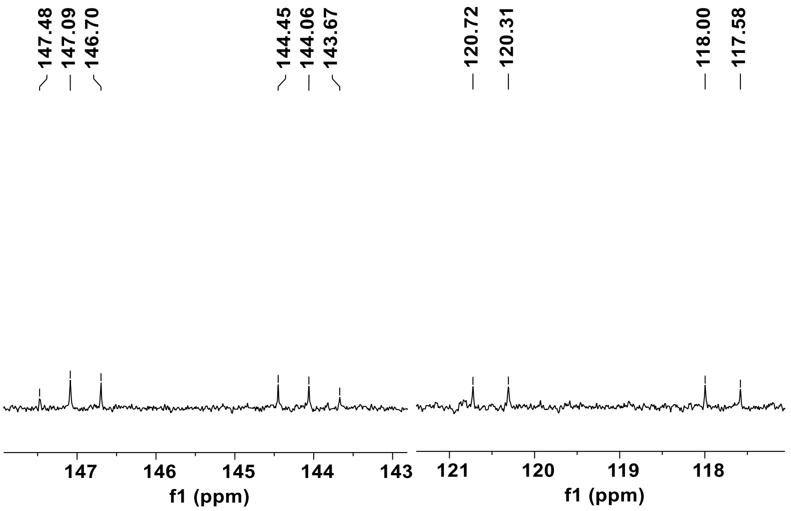

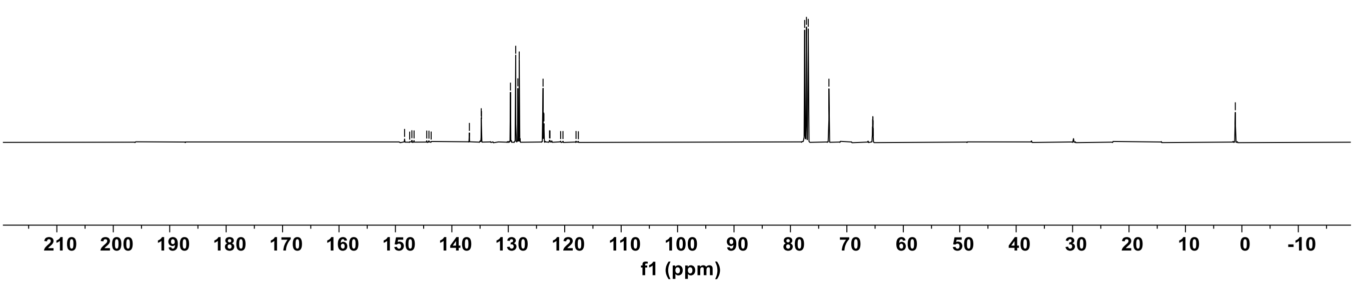

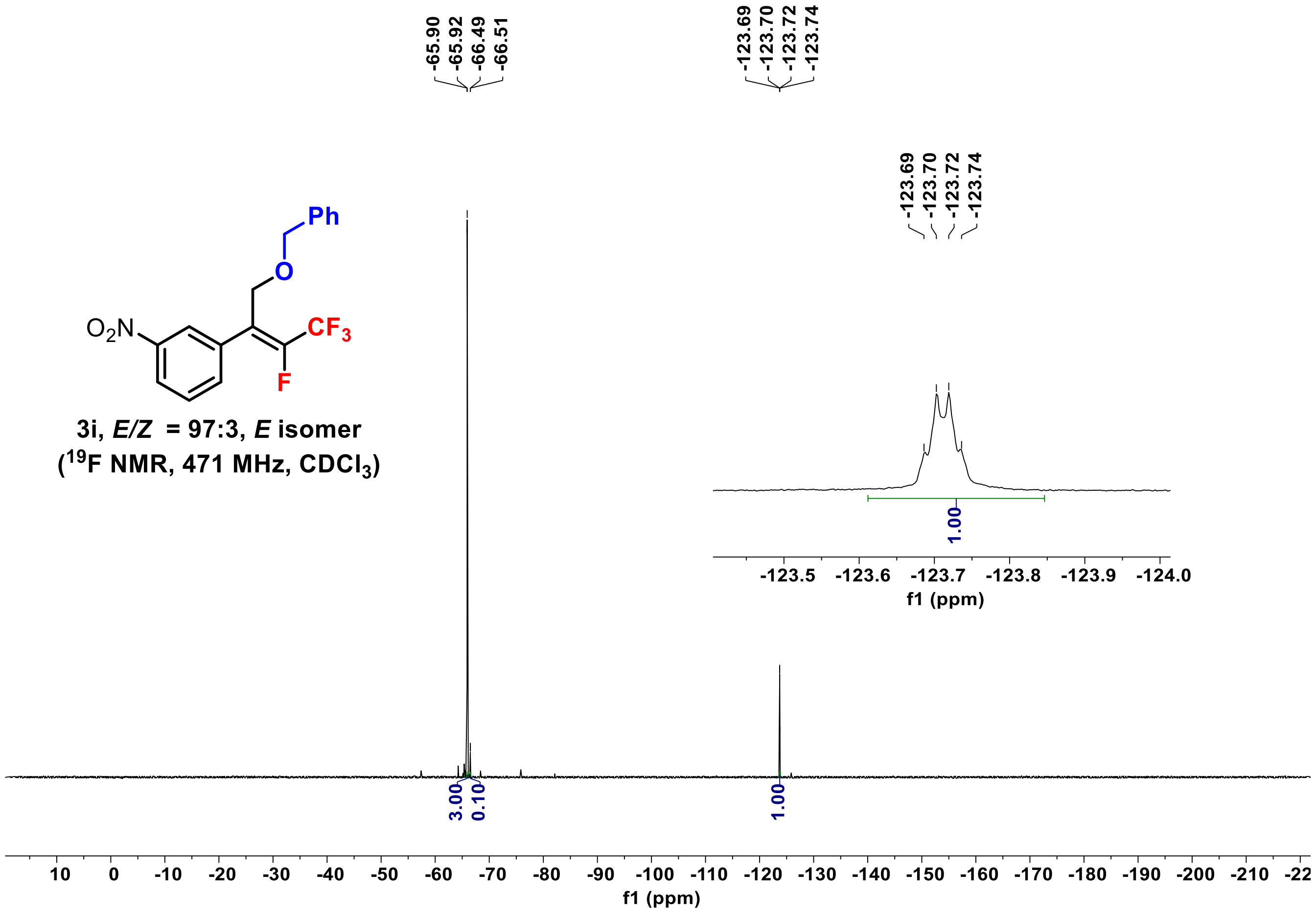
-65.90
-65.92
-66.49
-66.51
-123.69
-123.70
-123.72
-123.74
3i, E/Z = 97:3, E isomer
(19F NMR, 471 MHz, CDCl3)
3.00
0.10
1.00
f1 (ppm)

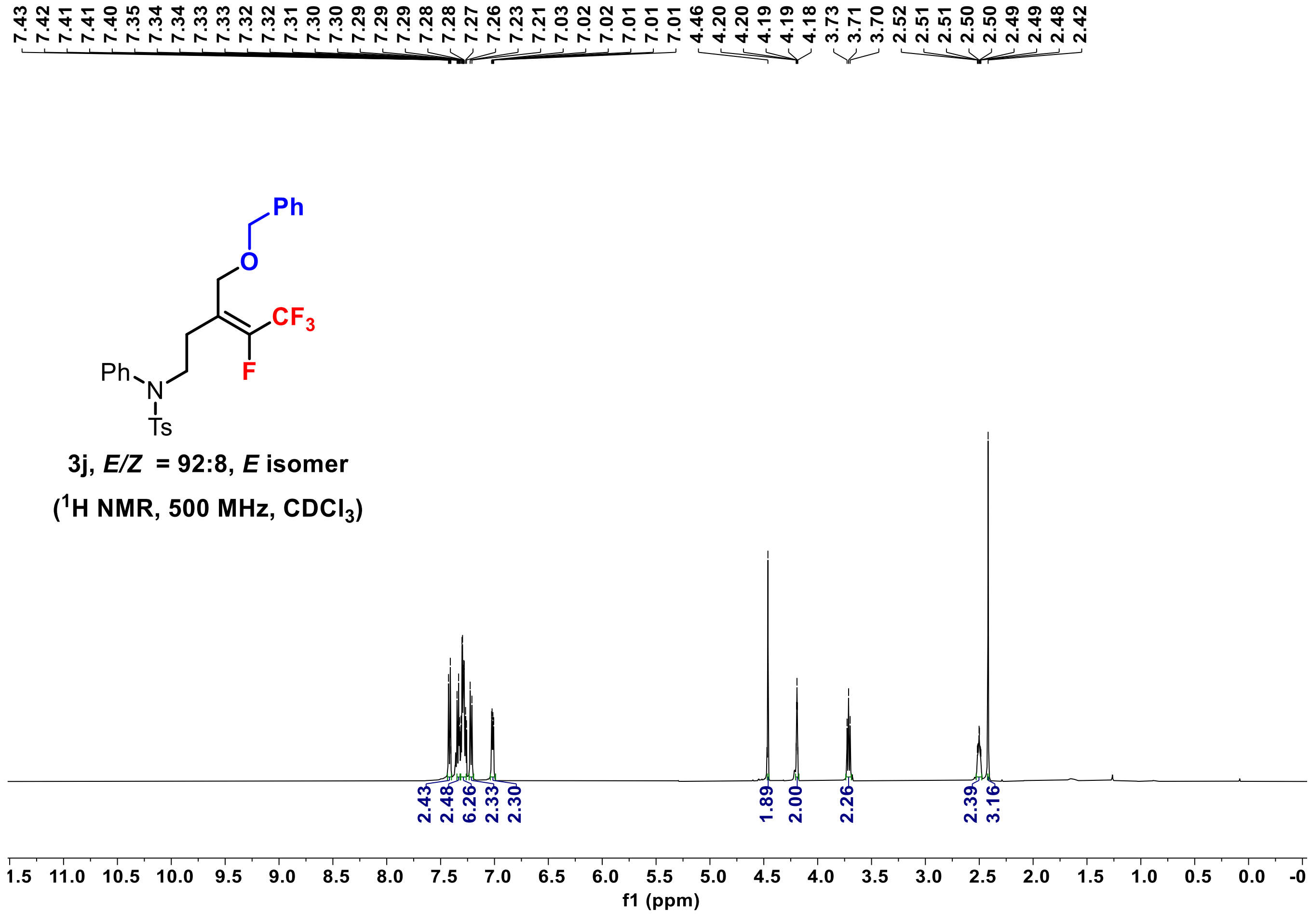
7.43 7.42 7.41 7.41 7.40 7.35 7.34 7.34 7.33 7.33 7.32 7.32 7.31 7.30 7.30 7.29 7.29 7.29 7.28 7.28 7.27 7.26 7.23 7.21 7.03 7.02 7.02 7.01 7.01 7.01 4.46 4.20 4.20 4.19 4.19 4.18 3.73 3.71 3.70 2.52 2.51 2.51 2.50 2.50 2.49 2.49 2.48 2.42
3j, E/Z = 92:8, E isomer
(1H NMR, 500 MHz, CDCl3)
2.43 2.48 6.26 2.33 2.30 1.89 2.00 2.26 2.39 3.16
f1 (ppm)

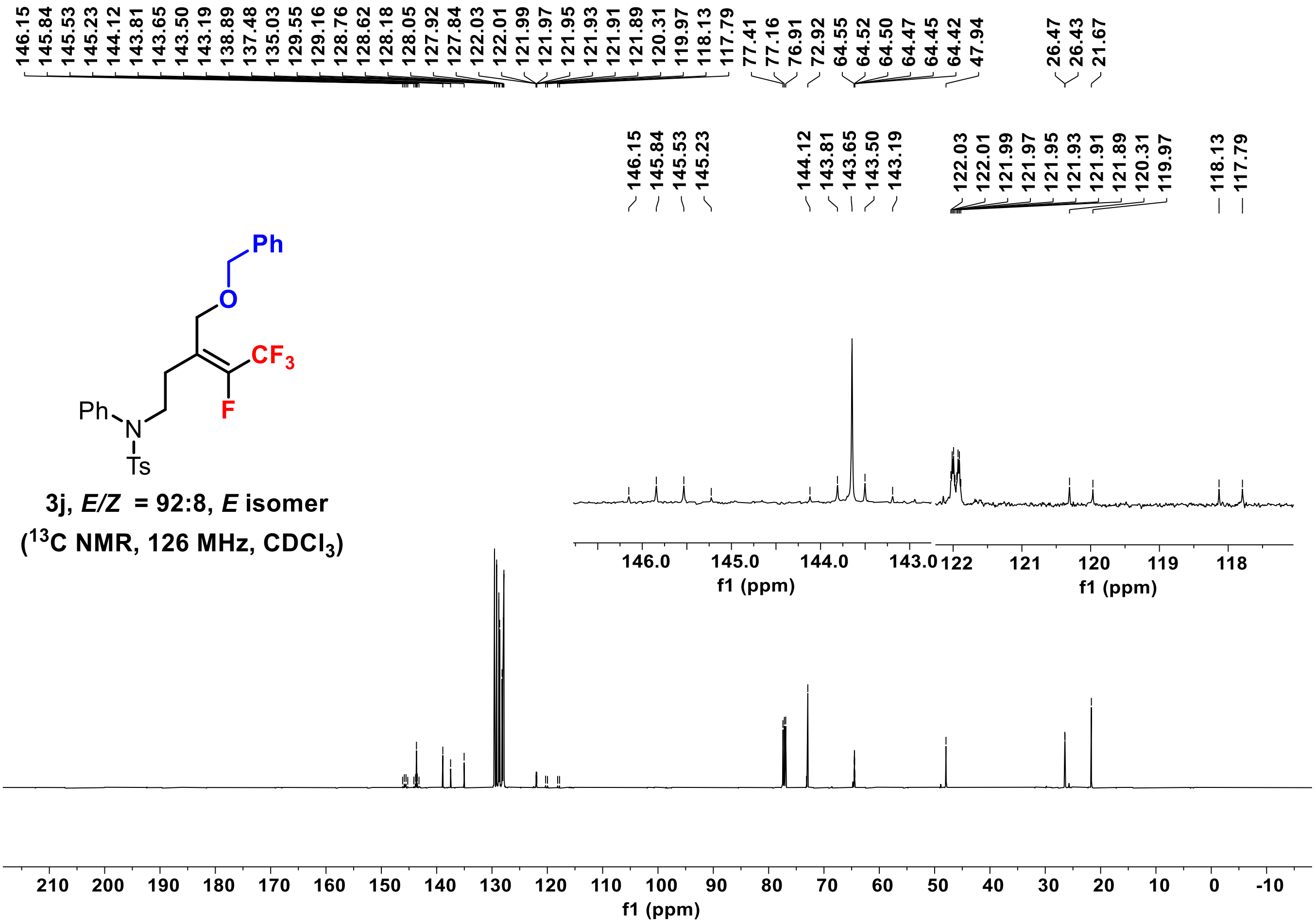

3j, *E/Z* = 92:8, *E* isomer
(^{13}C NMR, 126 MHz, $CDCl_3$)

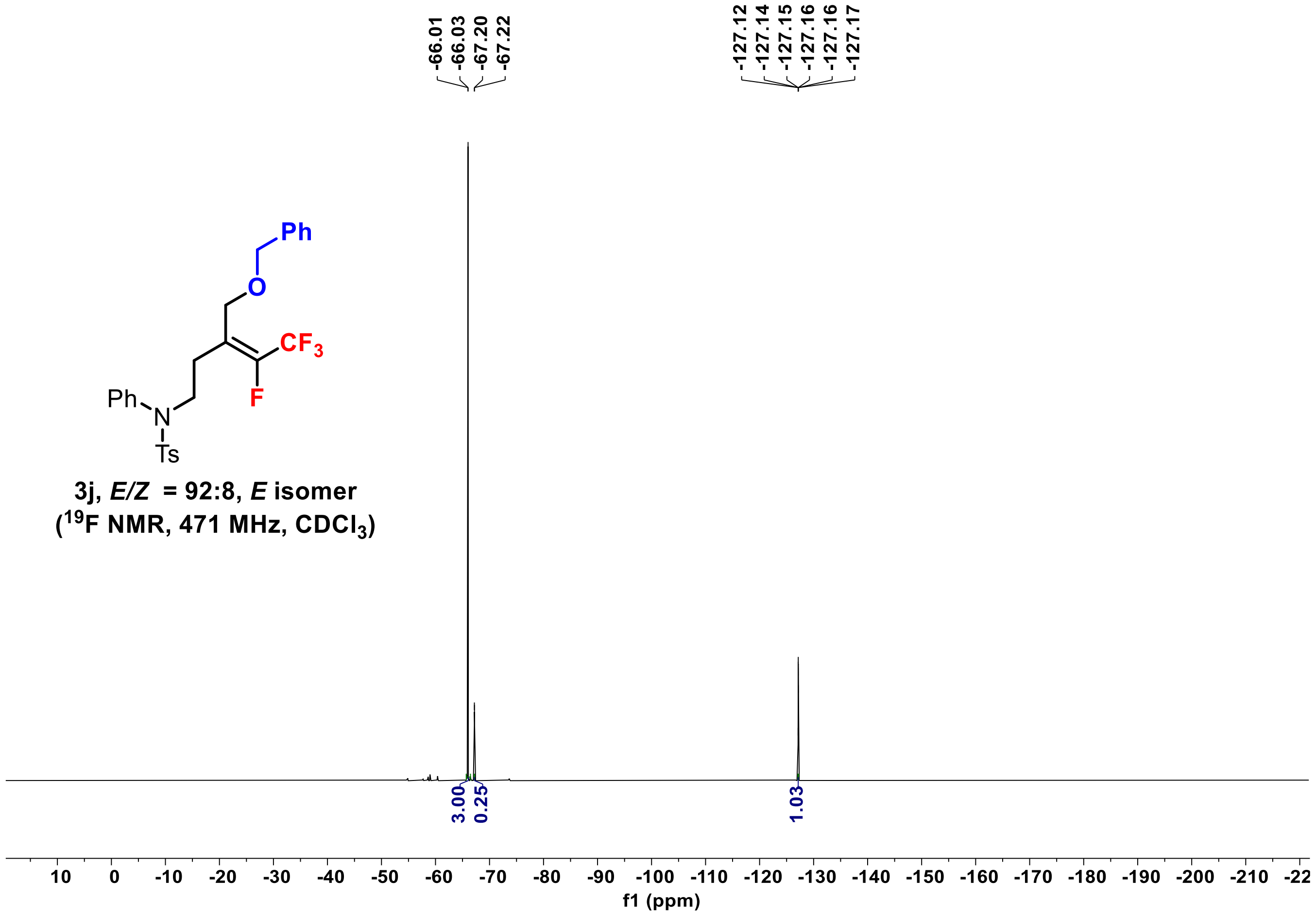

3j, *E/Z* = 92:8, *E* isomer
(^{19}F NMR, 471 MHz, $CDCl_3$)

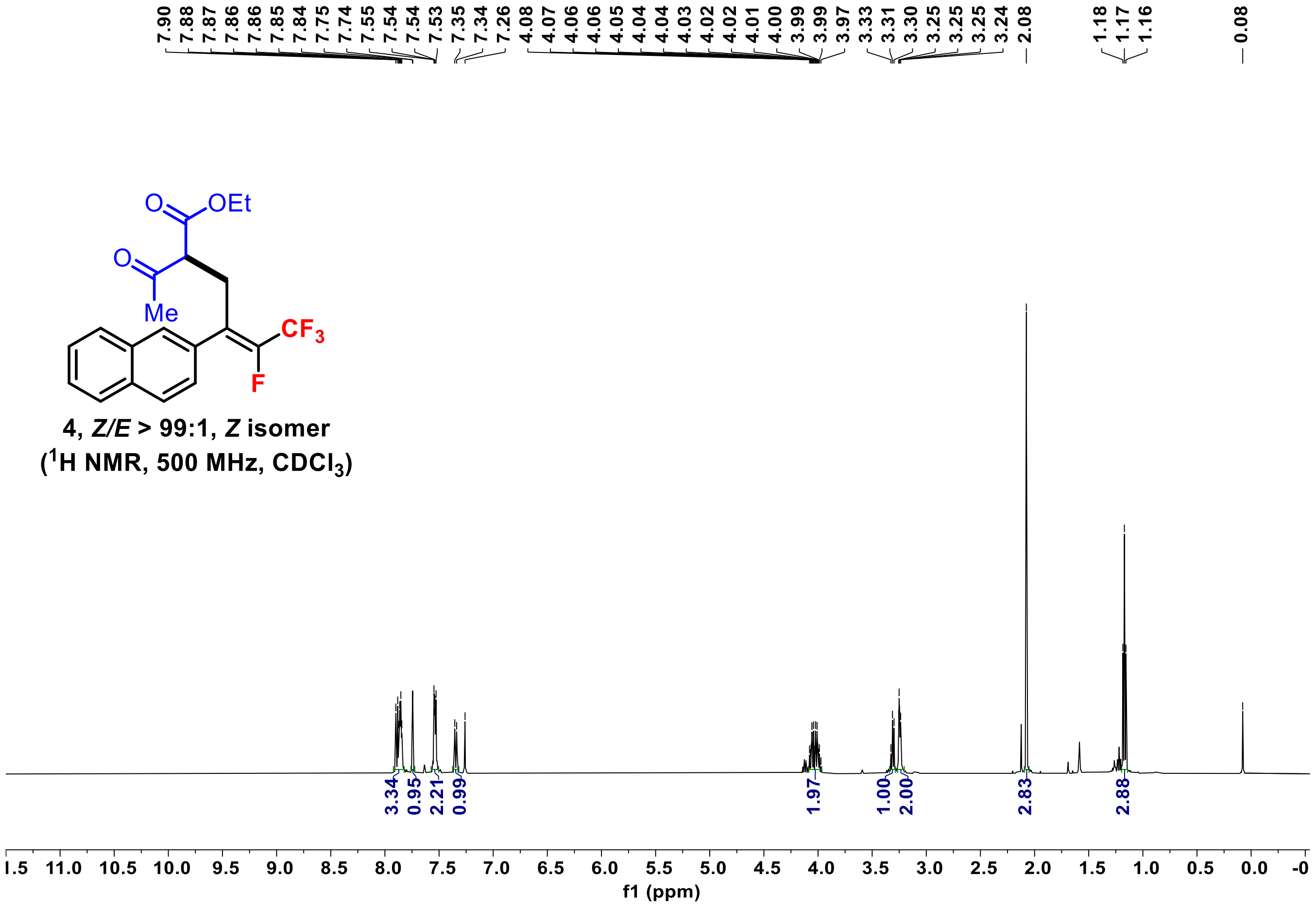

4, *Z/E* > 99:1, *Z* isomer
(^{1}H NMR, 500 MHz, CDCl$_3$)

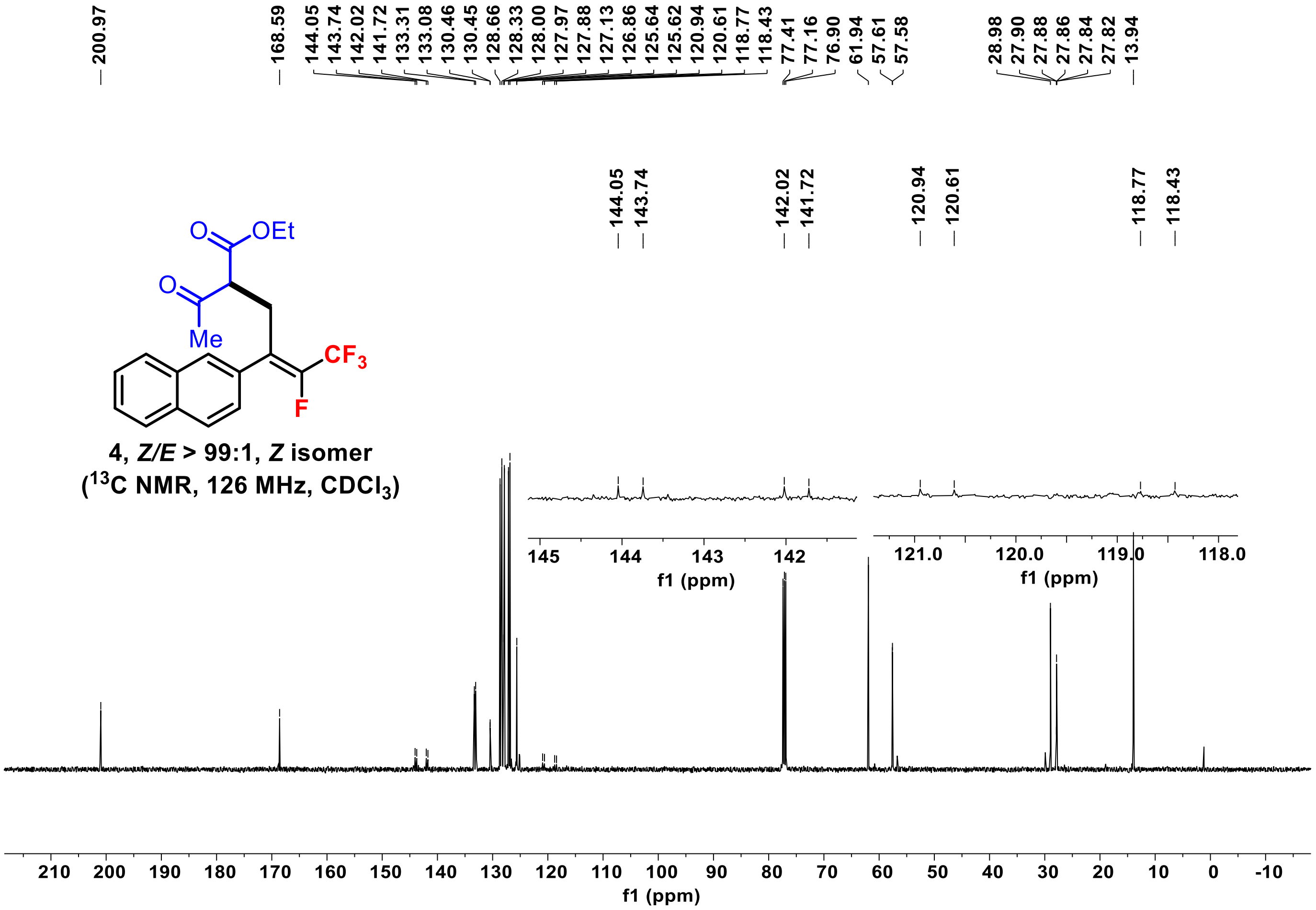

4, *Z/E* > 99:1, *Z* isomer
(^{13}C NMR, 126 MHz, CDCl$_3$)

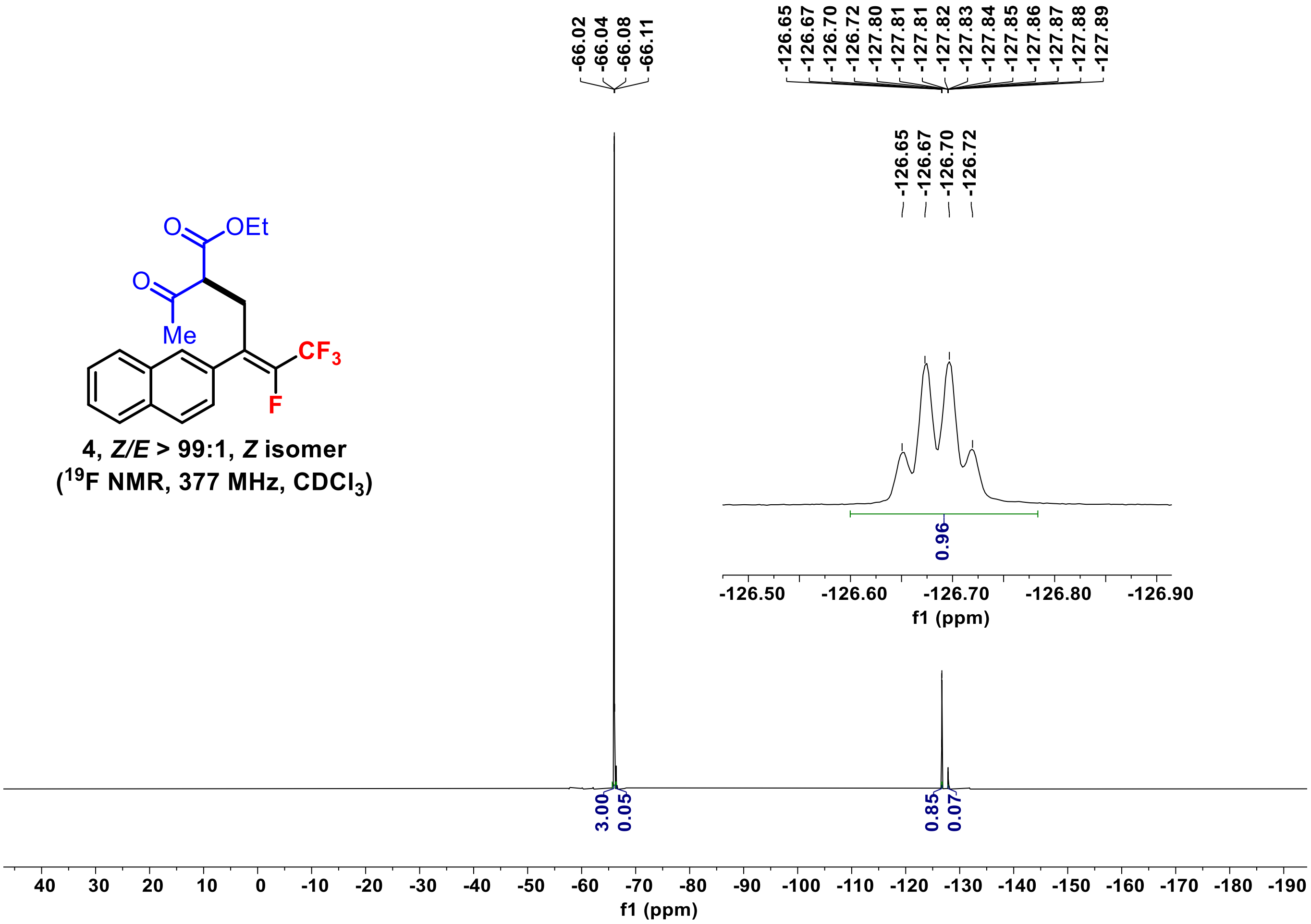
-66.02
-66.04
-66.08
-66.11
-126.65
-126.67
-126.70
-126.72
-127.80
-127.81
-127.81
-127.82
-127.83
-127.84
-127.85
-127.86
-127.87
-127.88
-127.89
OEt
O
O
Me
CF3
F
4, Z/E > 99:1, Z isomer
(19F NMR, 377 MHz, CDCl3)
0.96
-126.50 -126.60 -126.70 -126.80 -126.90
f1 (ppm)
3.00
0.05
0.85
0.07
40 30 20 10 0 -10 -20 -30 -40 -50 -60 -70 -80 -90 -100 -110 -120 -130 -140 -150 -160 -170 -180 -190
f1 (ppm)

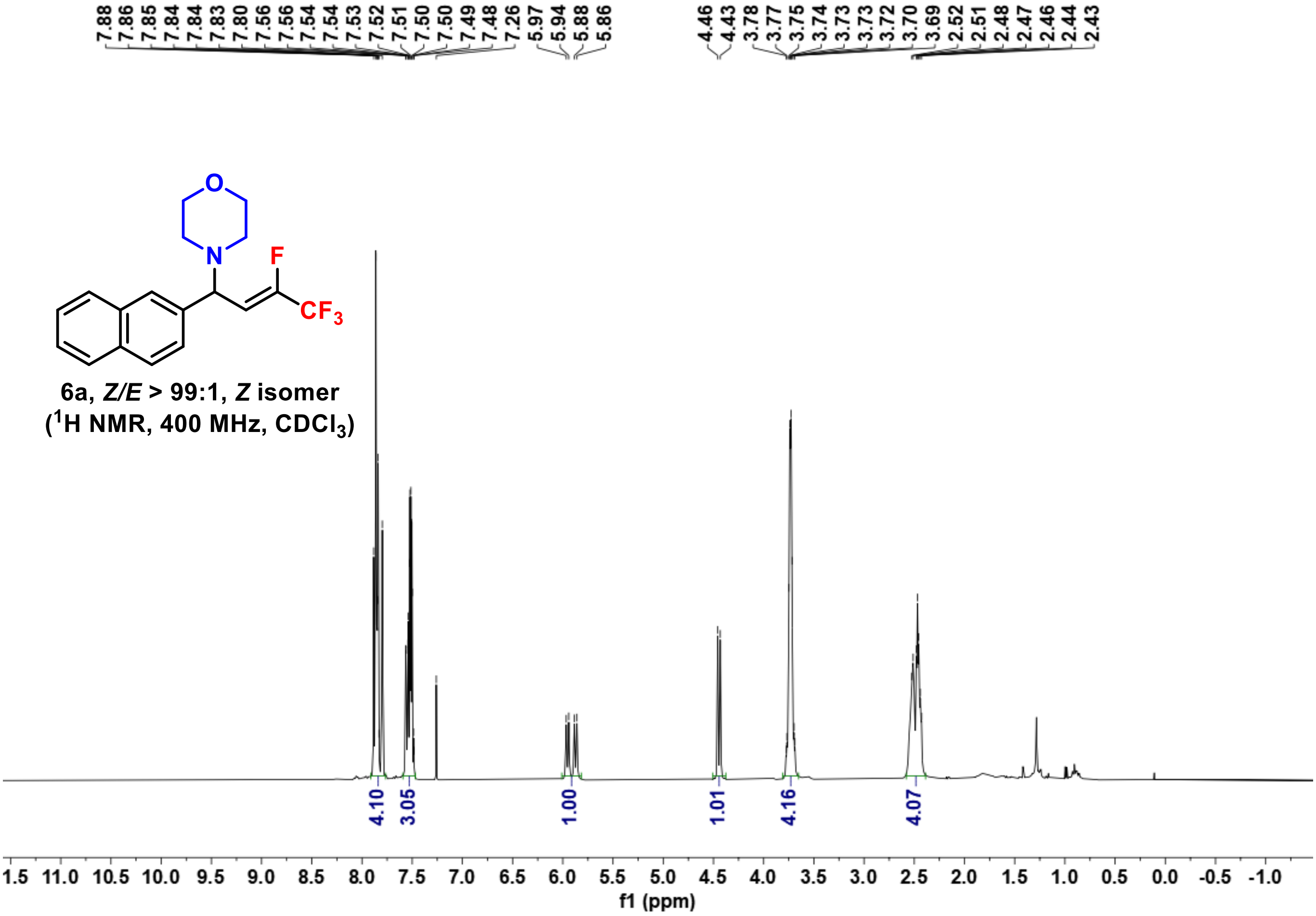
7.88
7.86
7.85
7.84
7.84
7.83
7.80
7.56
7.56
7.54
7.54
7.53
7.52
7.51
7.50
7.50
7.49
7.48
7.26
5.97
5.94
5.88
5.86
4.46
4.43
3.78
3.77
3.75
3.74
3.73
3.73
3.72
3.70
3.69
2.52
2.51
2.48
2.47
2.46
2.44
2.43
O
N
F
CF3
6a, Z/E > 99:1, Z isomer
(1H NMR, 400 MHz, CDCl3)
4.10
3.05
1.00
1.01
4.16
4.07
1.5 11.0 10.5 10.0 9.5 9.0 8.5 8.0 7.5 7.0 6.5 6.0 5.5 5.0 4.5 4.0 3.5 3.0 2.5 2.0 1.5 1.0 0.5 0.0 -0.5 -1.0
f1 (ppm)

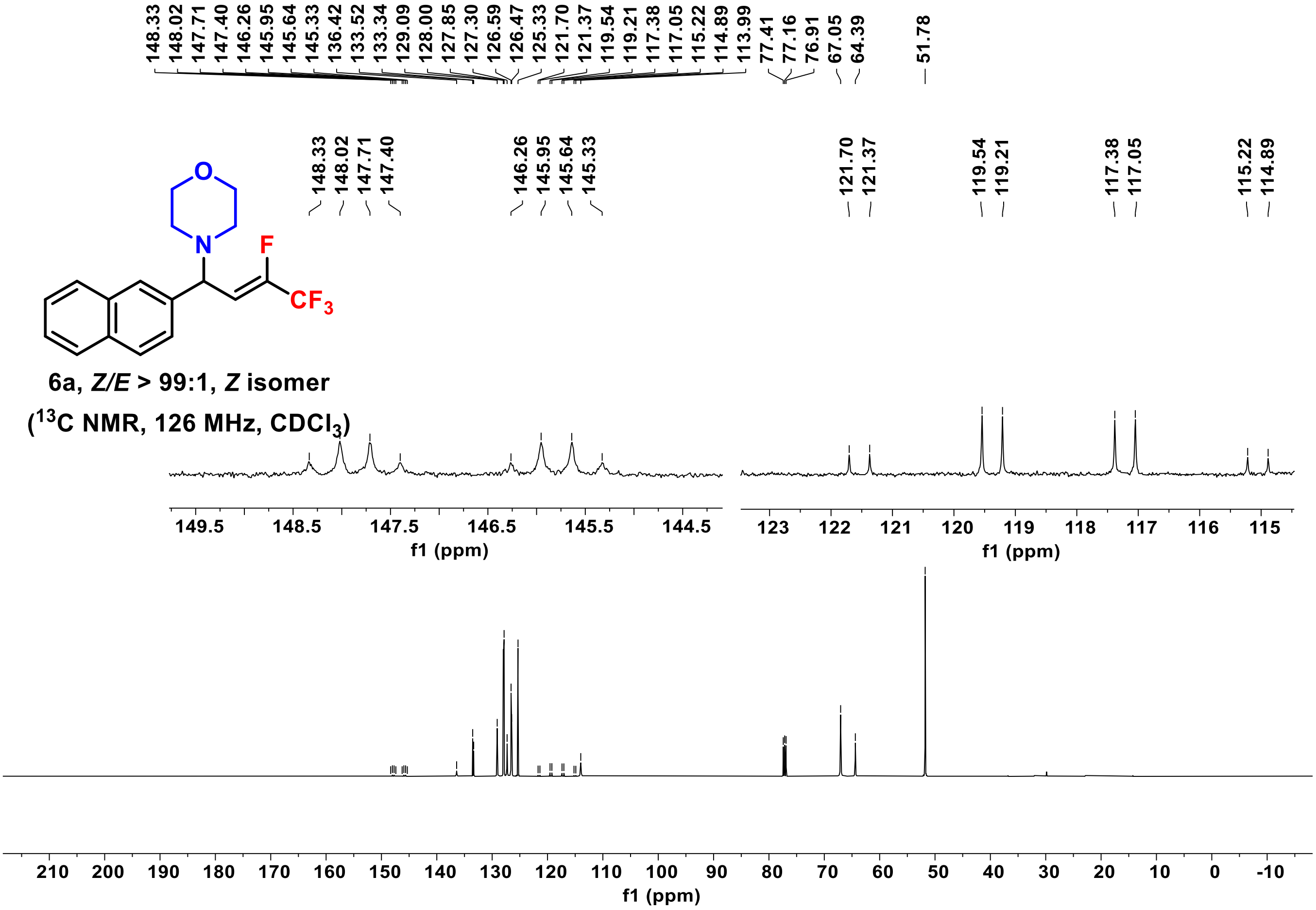
148.33
148.02
147.71
147.40
146.26
145.95
145.64
145.33
136.42
133.52
133.34
129.09
128.00
127.85
127.30
126.59
126.47
125.33
121.70
121.37
119.54
119.21
117.38
117.05
115.22
114.89
113.99
77.41
77.16
76.91
67.05
64.39
51.78
O
N
F
CF3
6a, Z/E > 99:1, Z isomer
(13C NMR, 126 MHz, CDCl3)
f1 (ppm)
f1 (ppm)
f1 (ppm)

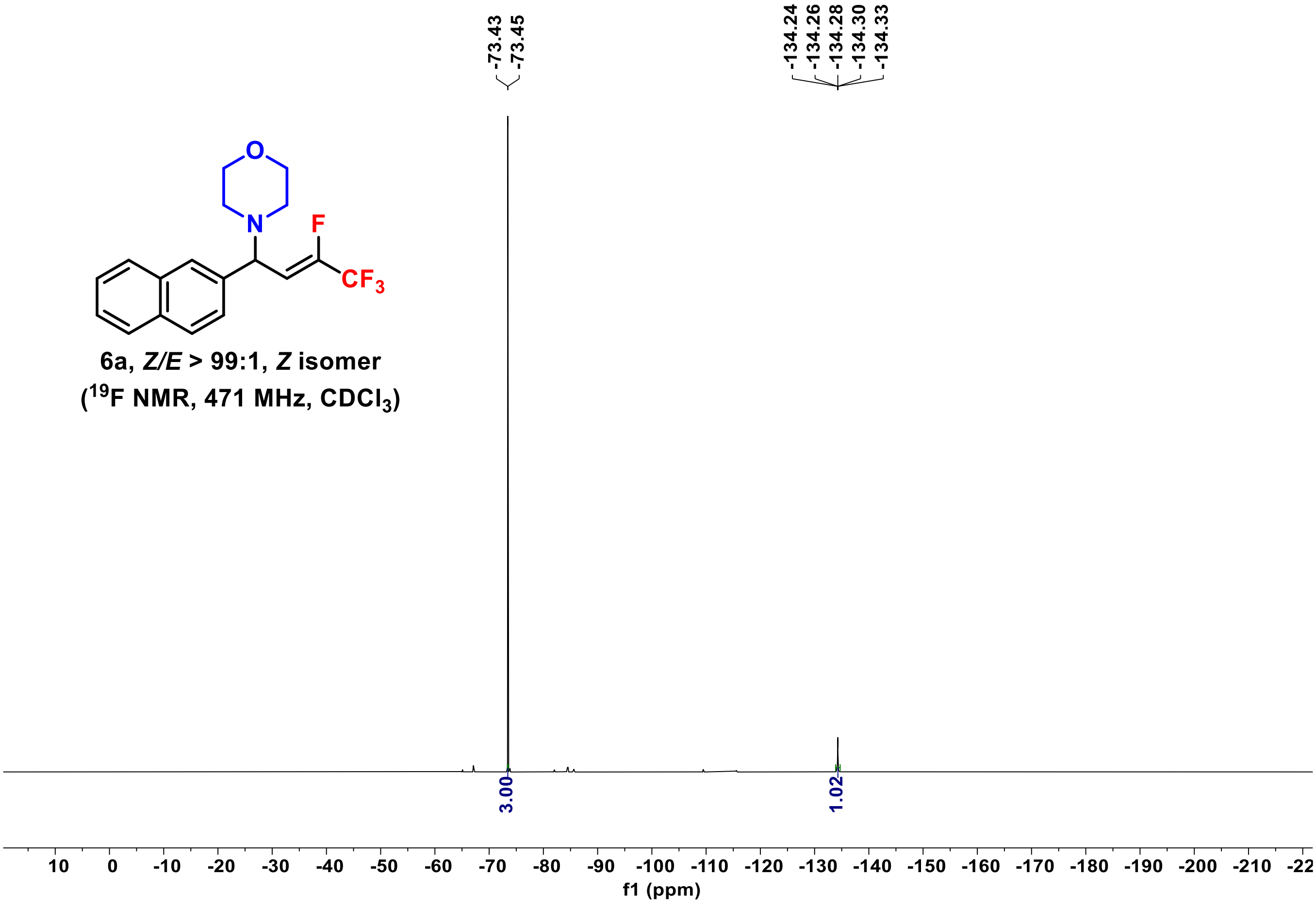
-73.43
-73.45
-134.24
-134.26
-134.28
-134.30
-134.33
O
N
F
CF3
6a, Z/E > 99:1, Z isomer
(19F NMR, 471 MHz, CDCl3)
3.00
1.02
f1 (ppm)

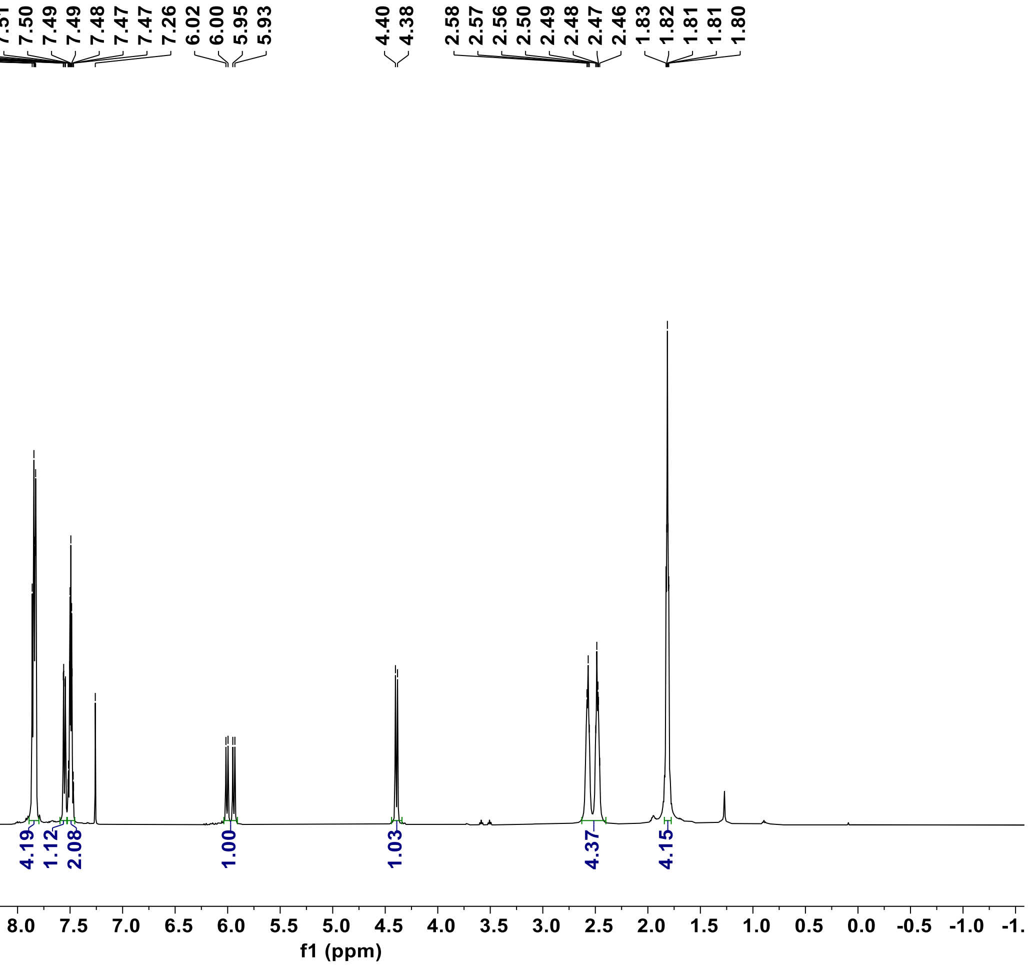

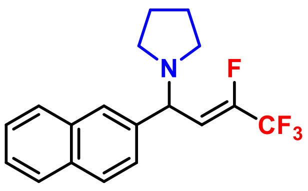

6b, *Z/E* > 99:1, *Z* isomer
(^{1}H NMR, 500 MHz, CDCl$_3$)

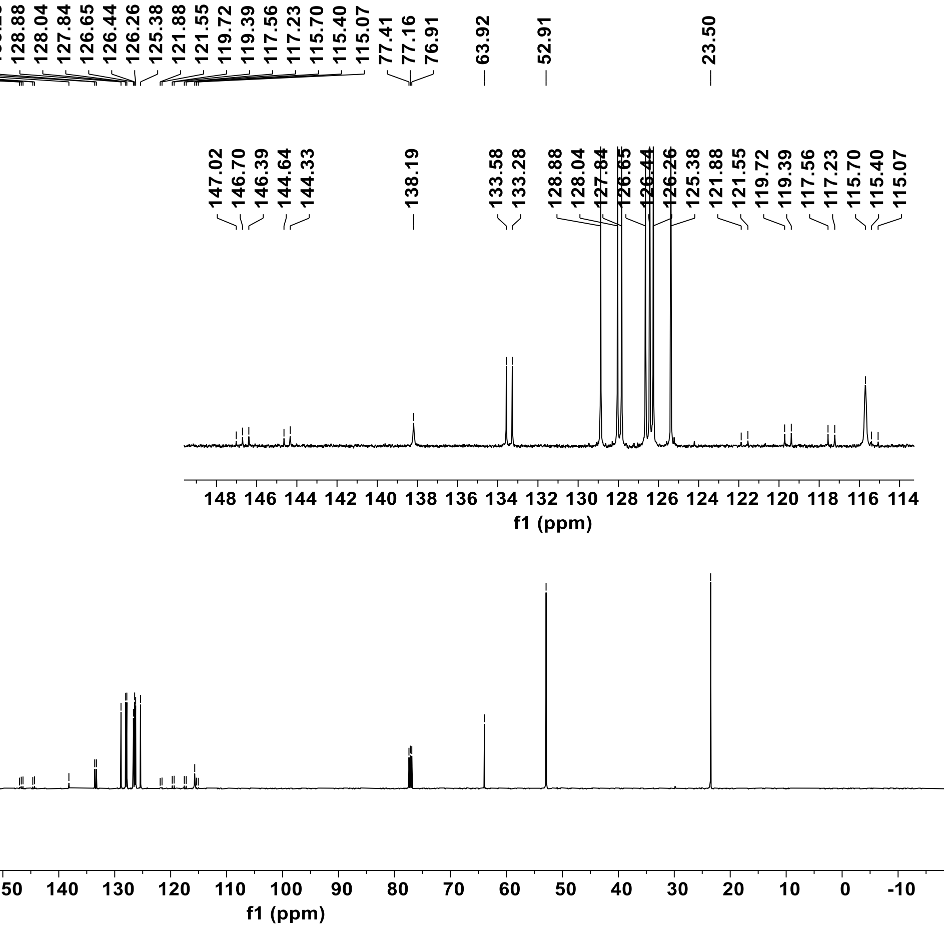

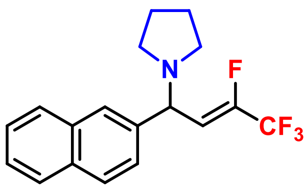

6b, *Z/E* > 99:1, *Z* isomer
(^{13}C NMR, 126 MHz, CDCl$_3$)

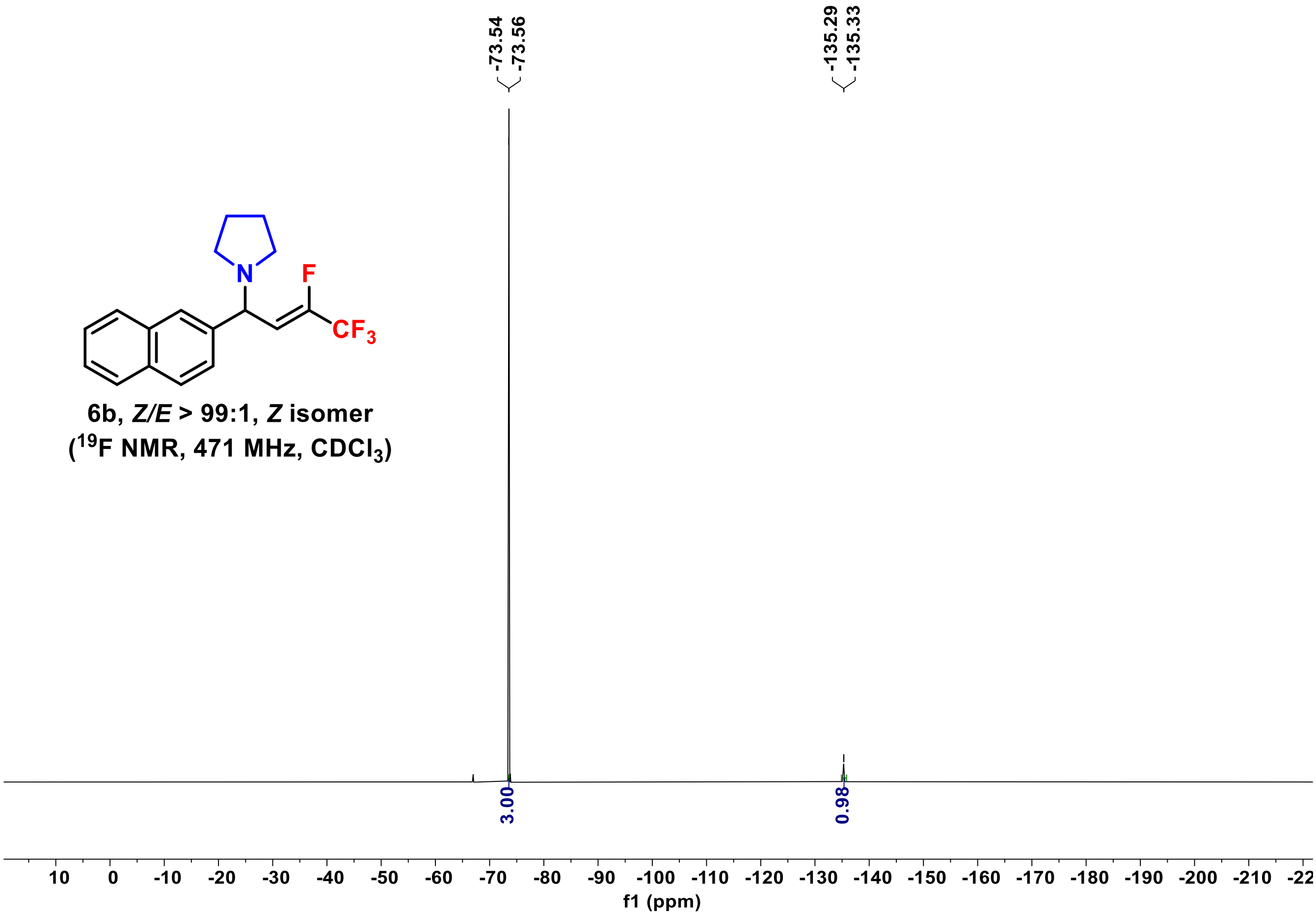

6b, *Z/E* > 99:1, *Z* isomer
(^{19}F NMR, 471 MHz, $CDCl_3$)

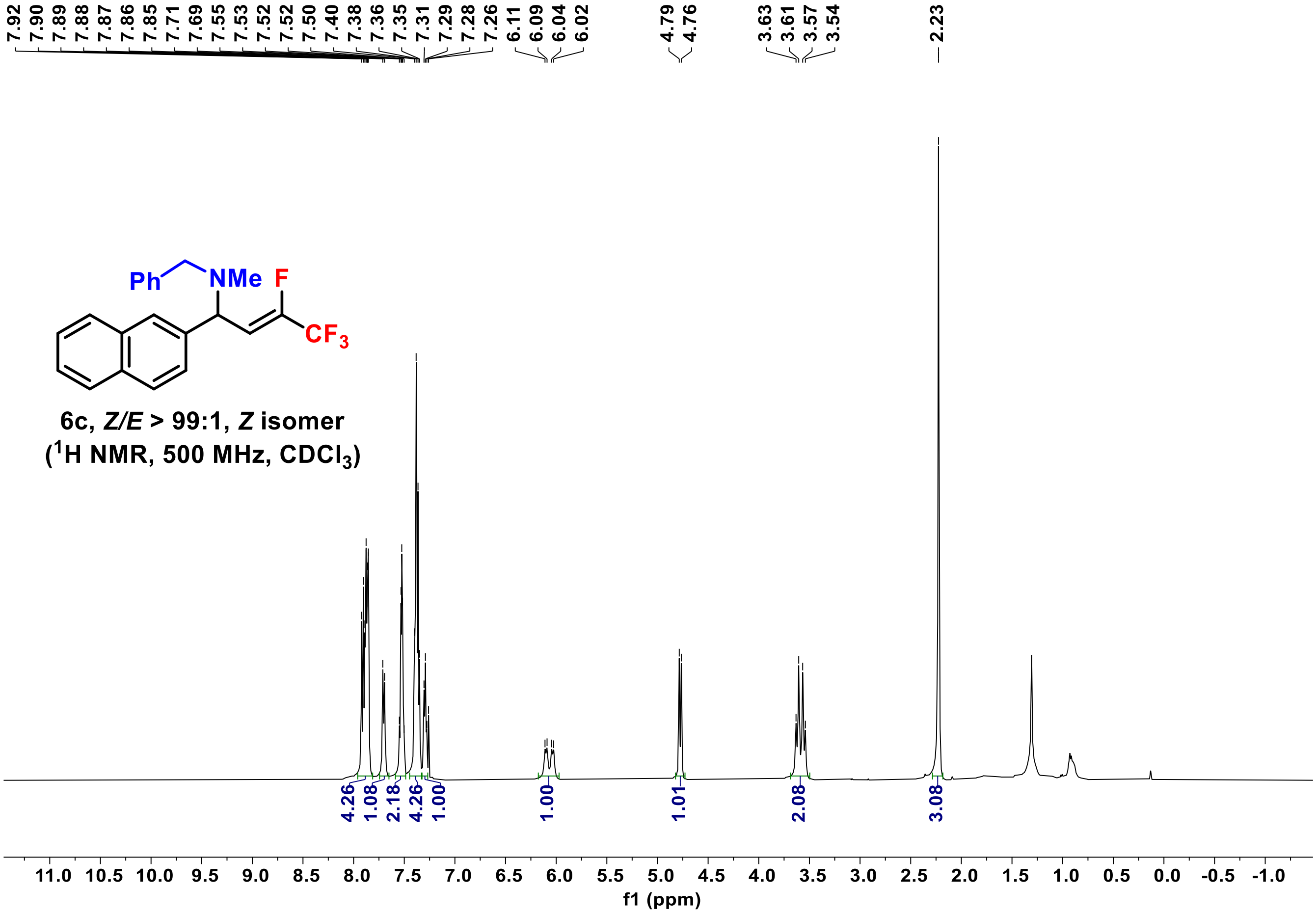

6c, *Z/E* > 99:1, *Z* isomer
(^{1}H NMR, 500 MHz, $CDCl_3$)

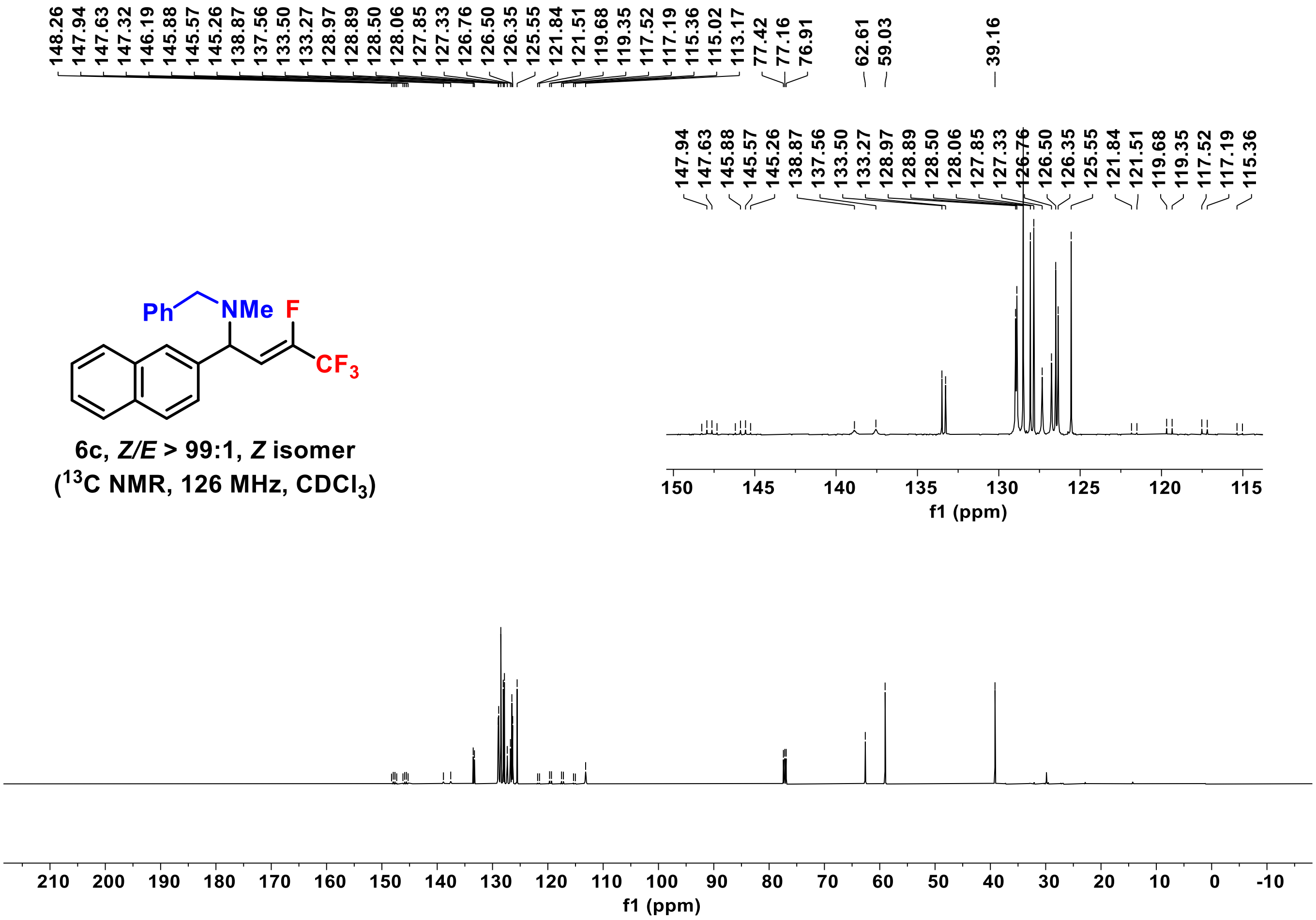

6c, *Z/E* > 99:1, *Z* isomer

(^{13}C NMR, 126 MHz, $CDCl_3$)

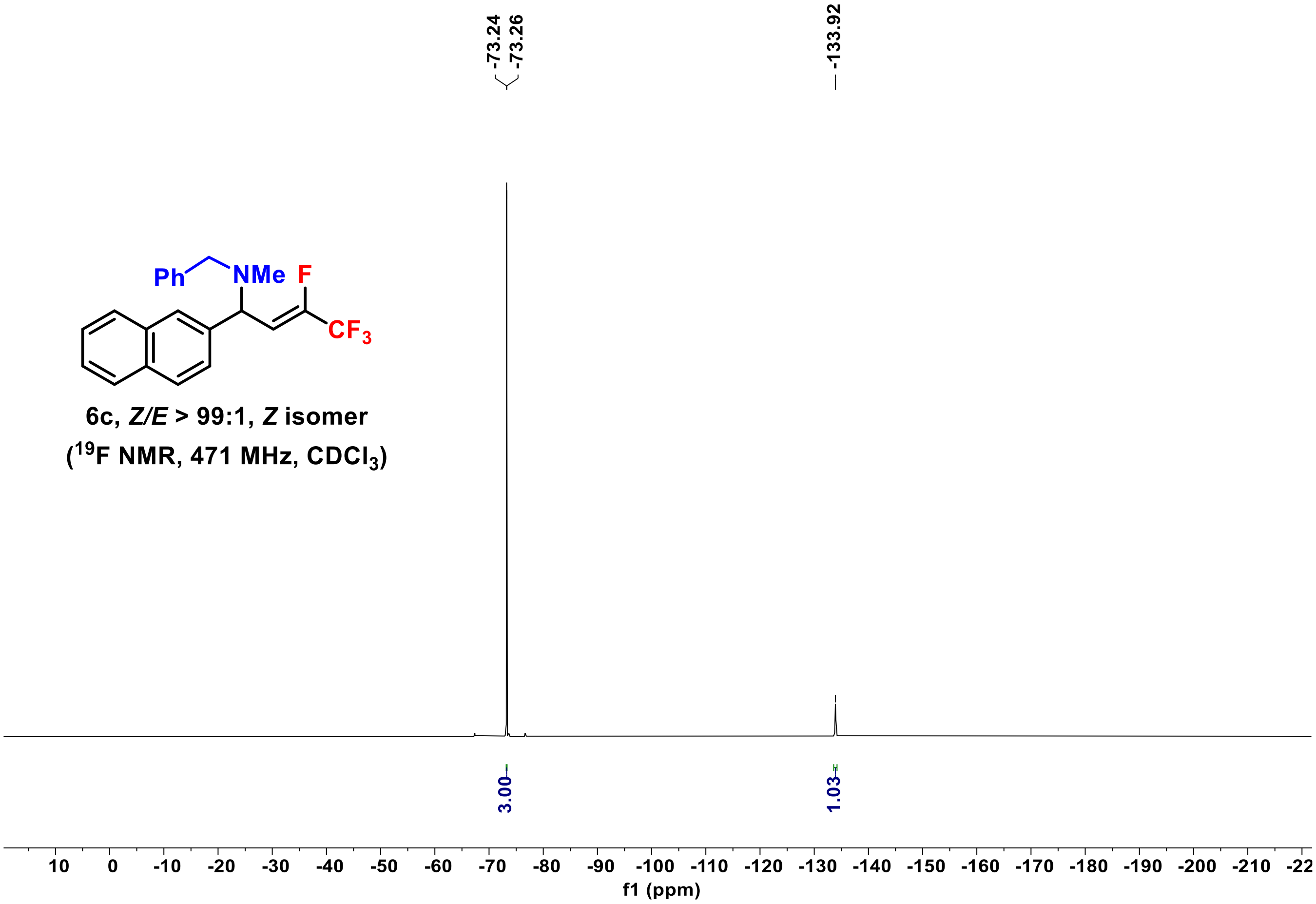

6c, *Z/E* > 99:1, *Z* isomer

(^{19}F NMR, 471 MHz, $CDCl_3$)

6d, *Z/E* > 99:1, *Z* isomer
(^{1}H NMR, 400 MHz, CDCl$_3$)

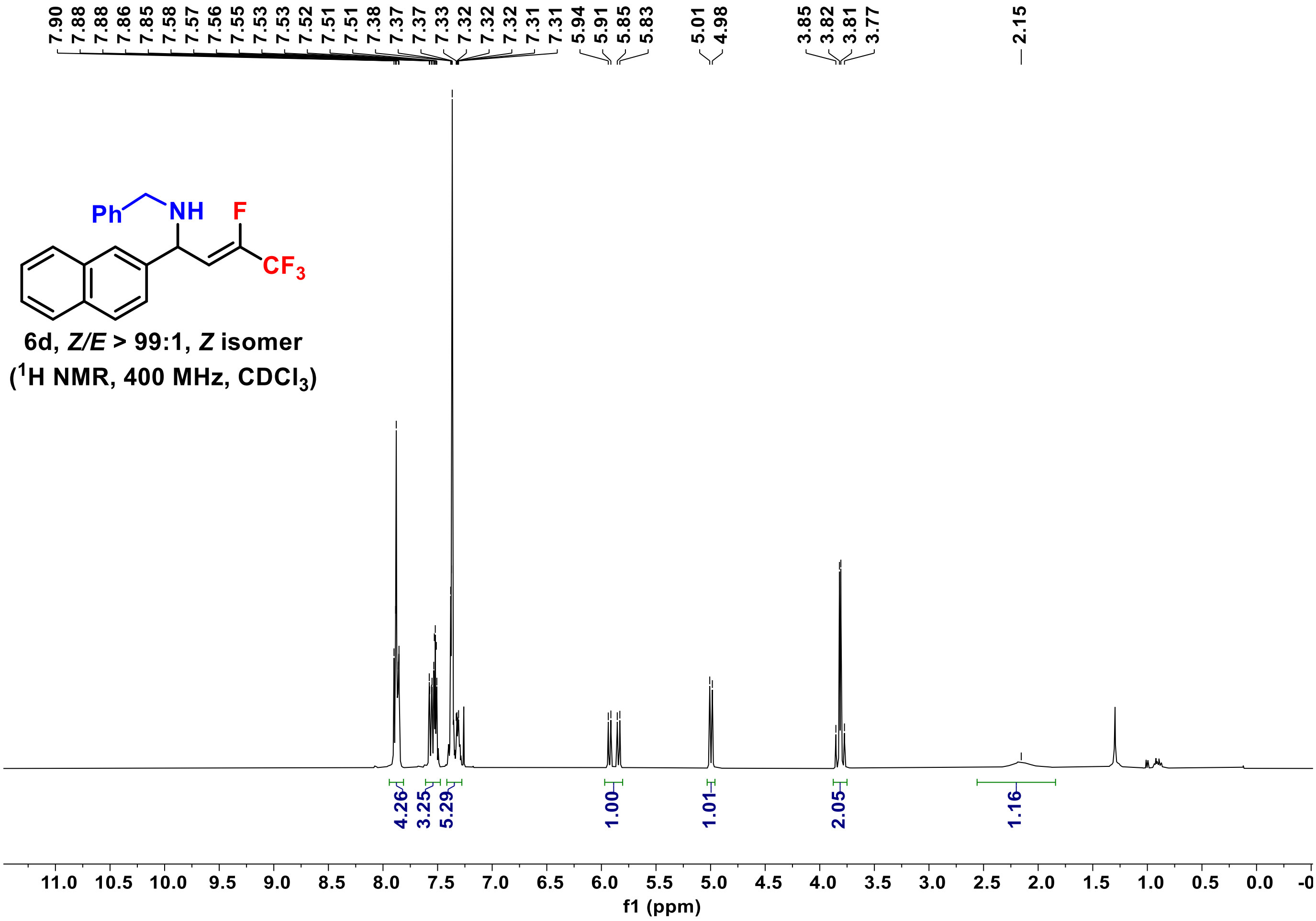

6d, *Z/E* > 99:1, *Z* isomer
(^{13}C NMR, 126 MHz, CDCl$_3$)

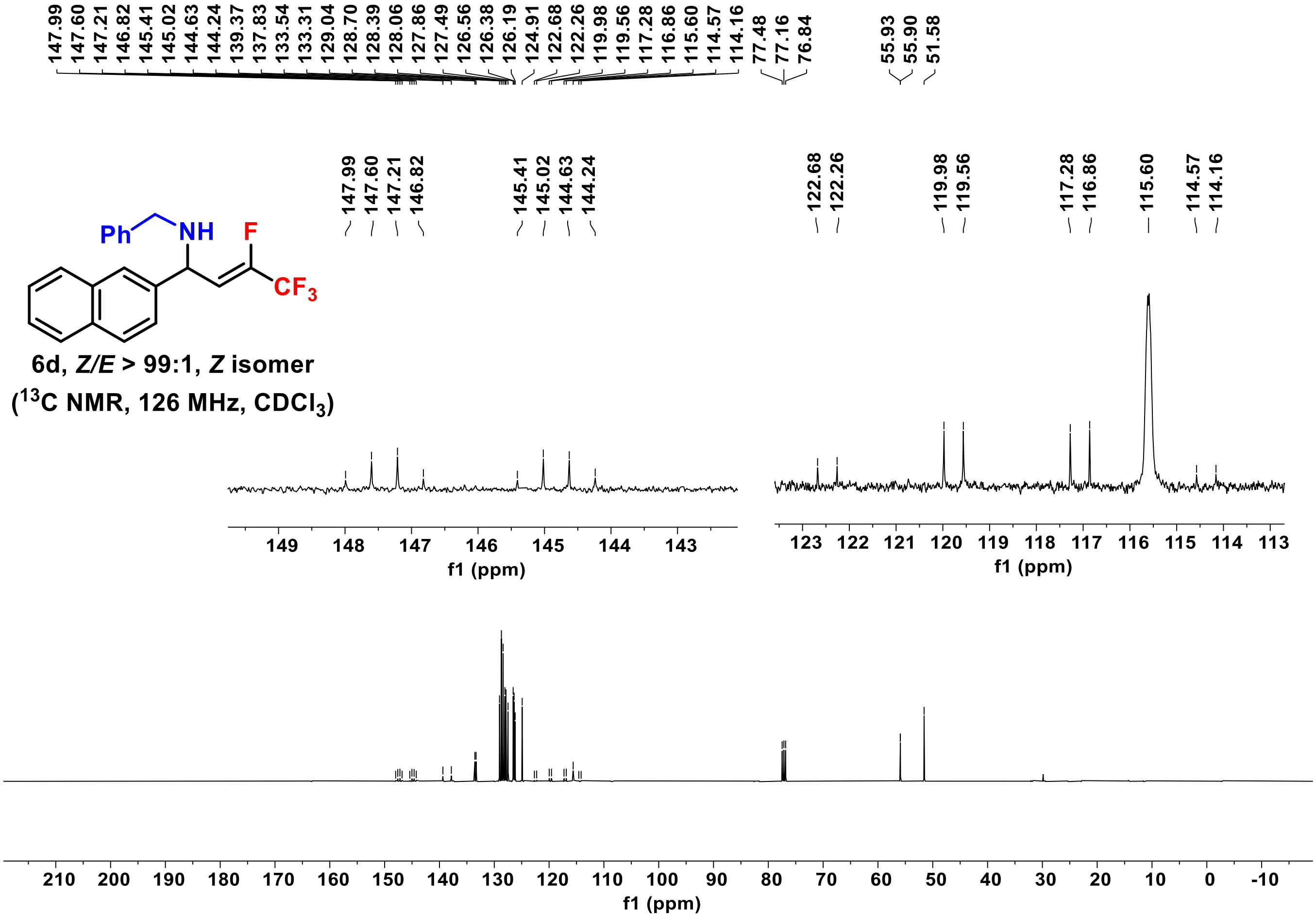

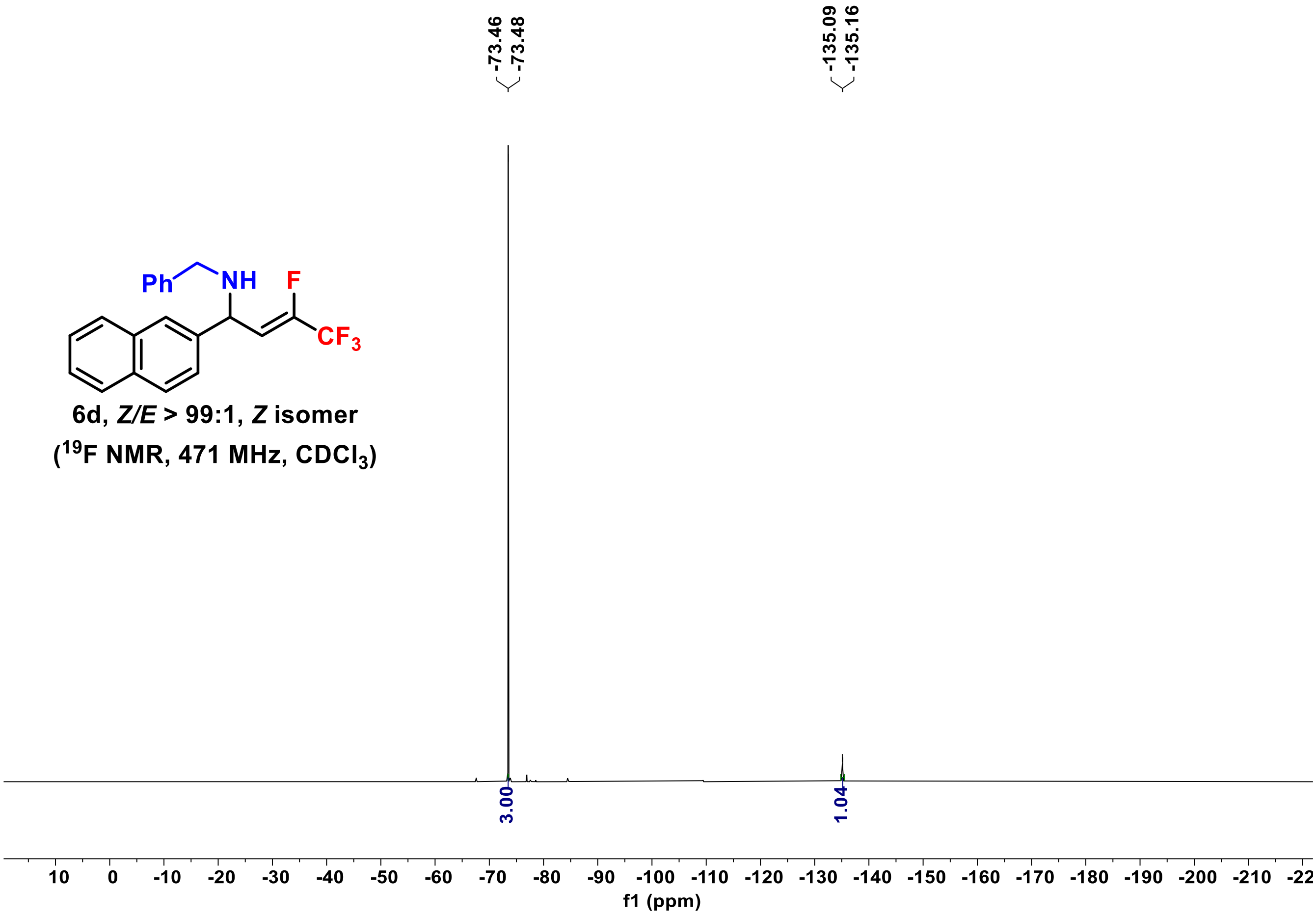

6d, *Z/E* > 99:1, *Z* isomer
(^{19}F NMR, 471 MHz, $CDCl_3$)

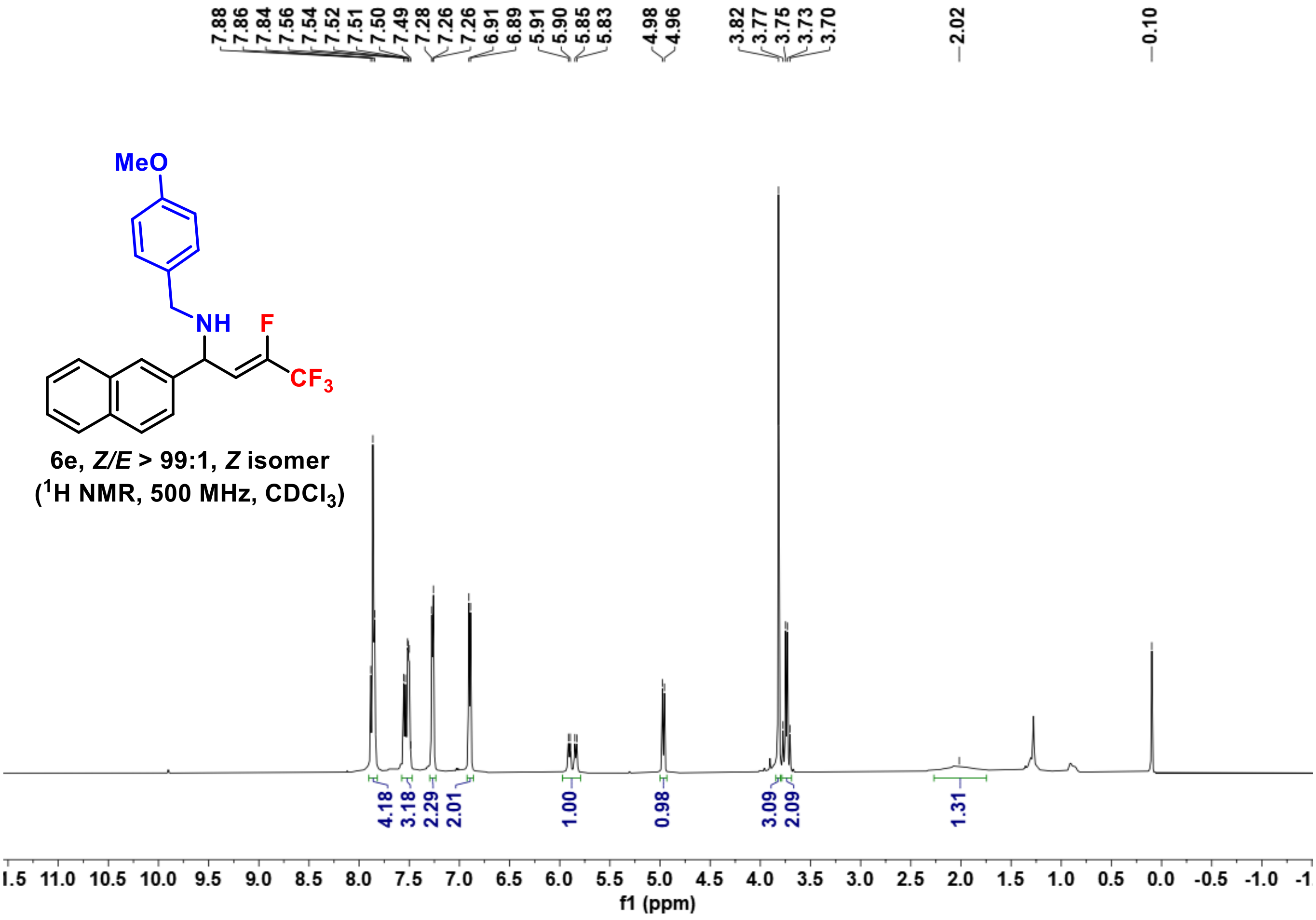

6e, *Z/E* > 99:1, *Z* isomer
(^{1}H NMR, 500 MHz, $CDCl_3$)

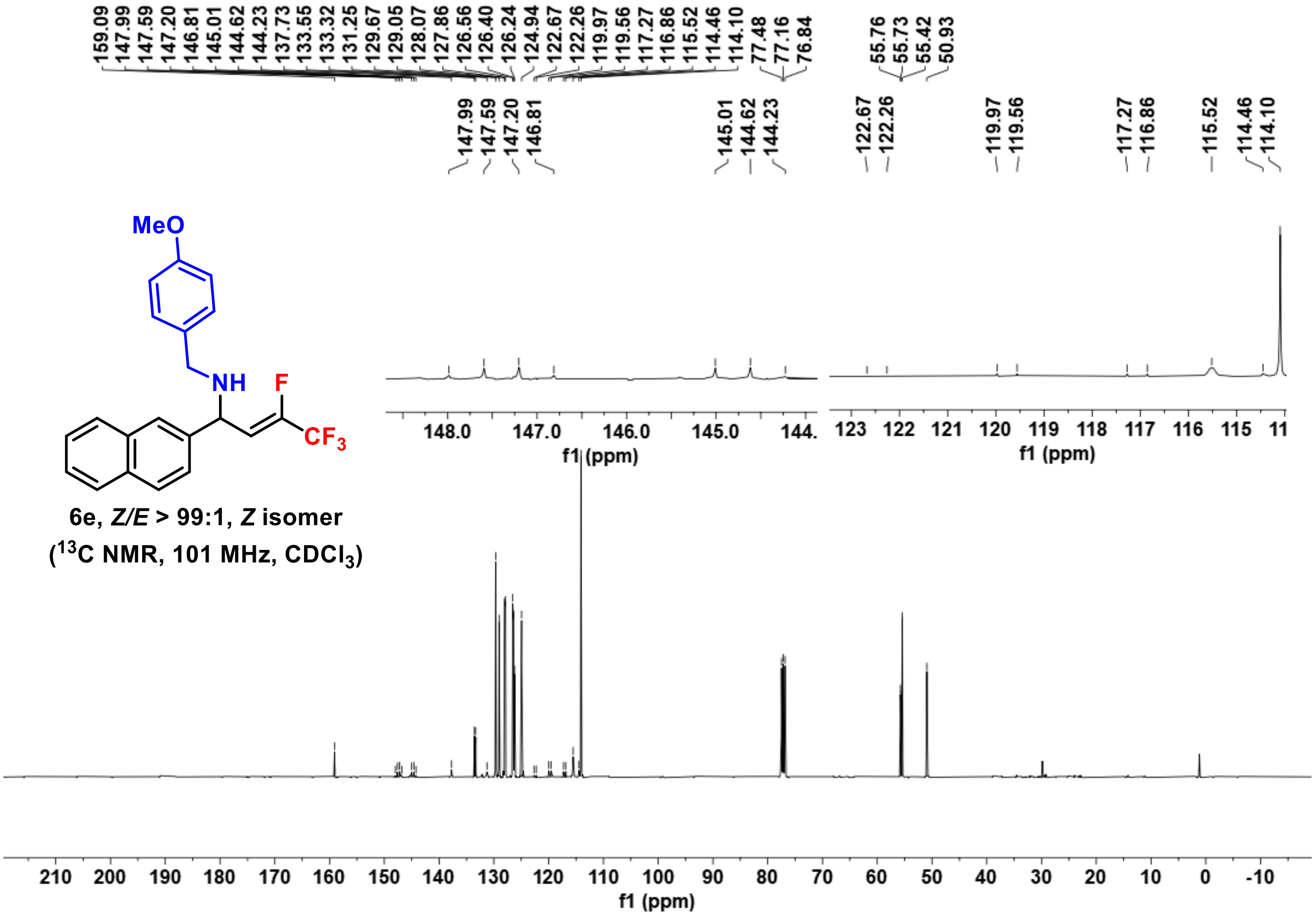

6e, *Z/E* > 99:1, *Z* isomer
(^{13}C NMR, 101 MHz, $CDCl_3$)

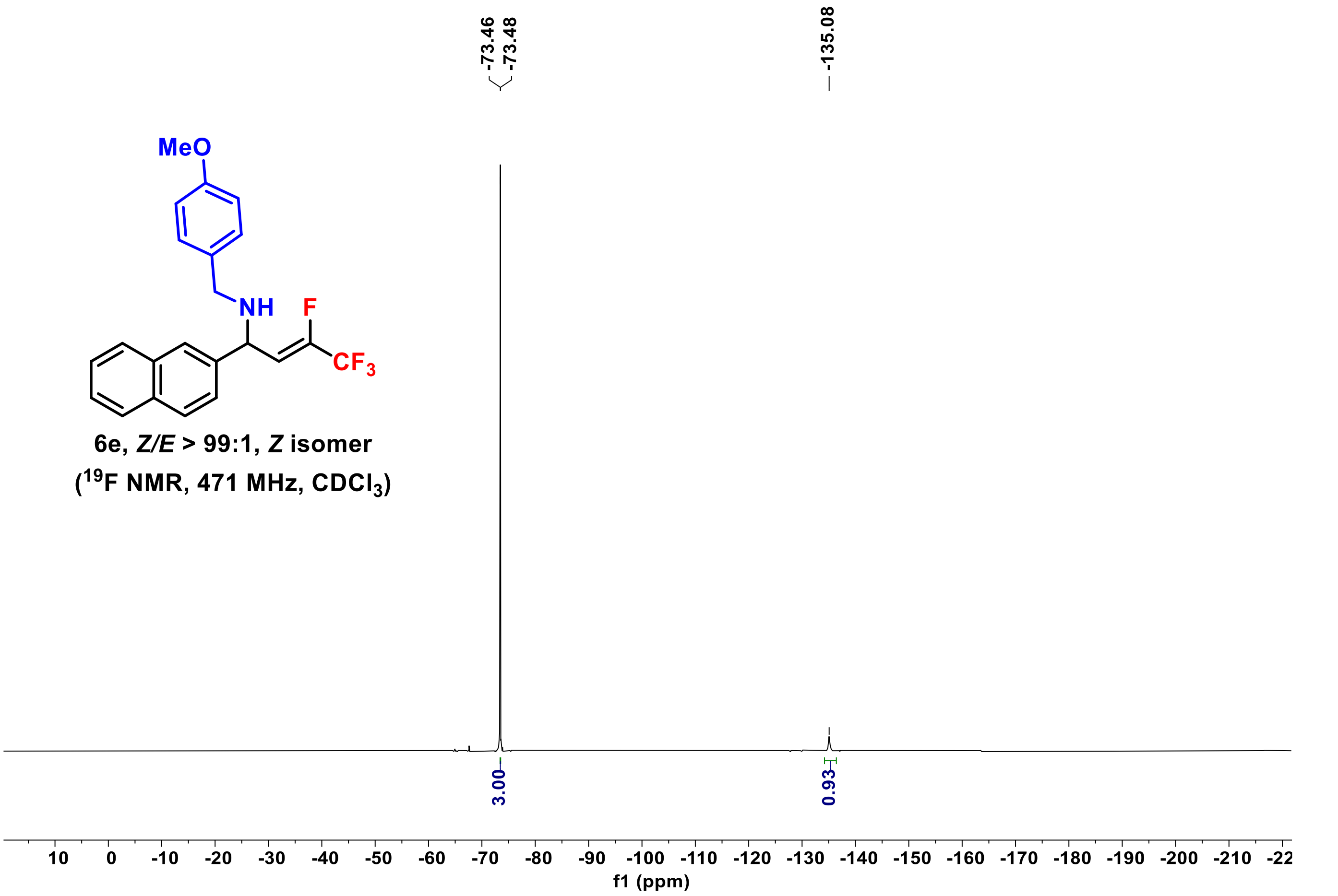

6e, *Z/E* > 99:1, *Z* isomer
(^{19}F NMR, 471 MHz, $CDCl_3$)

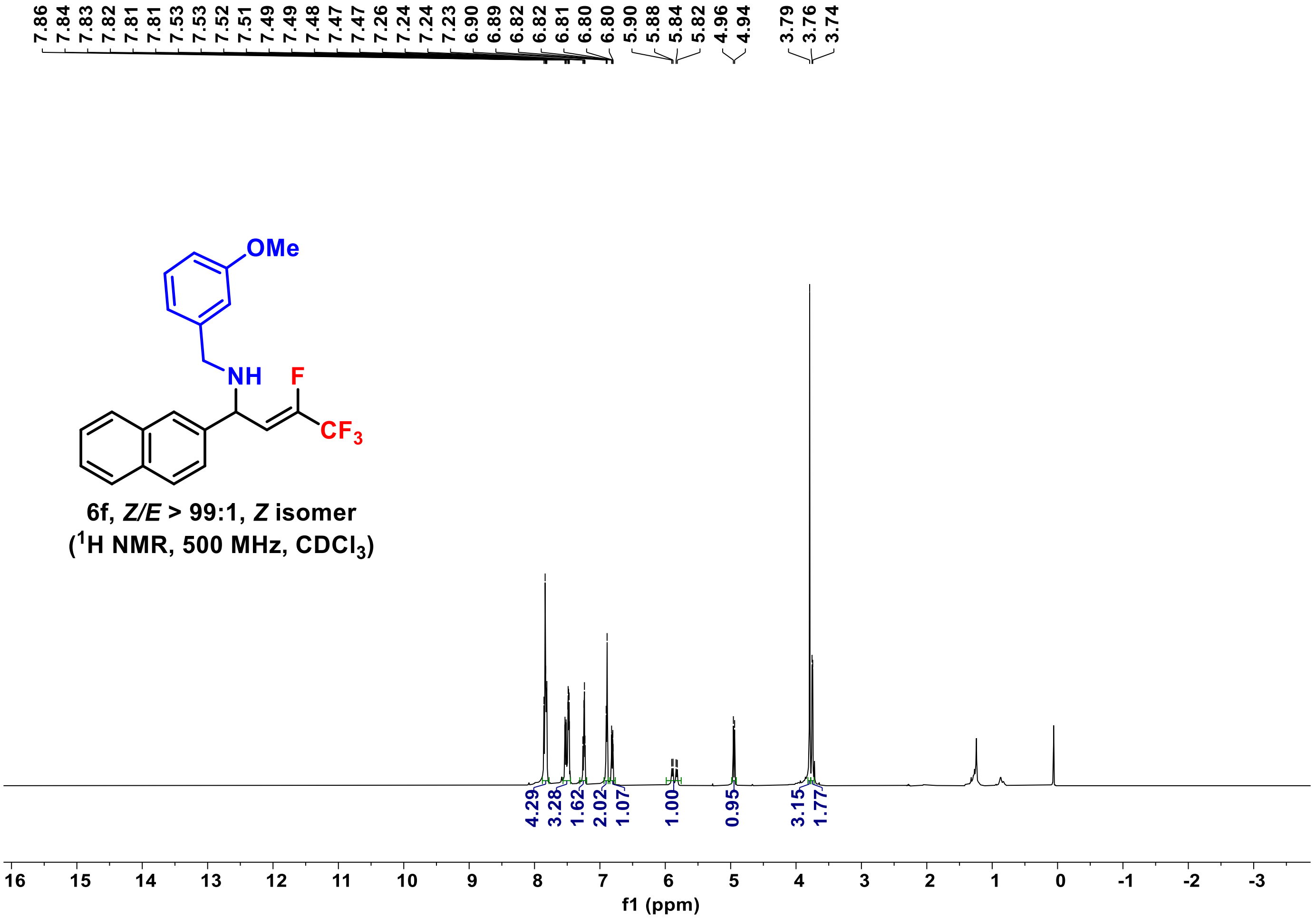
7.86 7.84 7.83 7.82 7.81 7.81 7.53 7.53 7.52 7.51 7.49 7.49 7.48 7.47 7.47 7.26 7.24 7.24 7.23 6.90 6.89 6.82 6.82 6.81 6.80 6.80 5.90 5.88 5.84 5.82 4.96 4.94 3.79 3.76 3.74
OMe
NH
F
CF3
6f, Z/E > 99:1, Z isomer
(1H NMR, 500 MHz, CDCl3)
4.29 3.28 1.62 2.02 1.07 1.00 0.95 3.15 1.77
f1 (ppm)

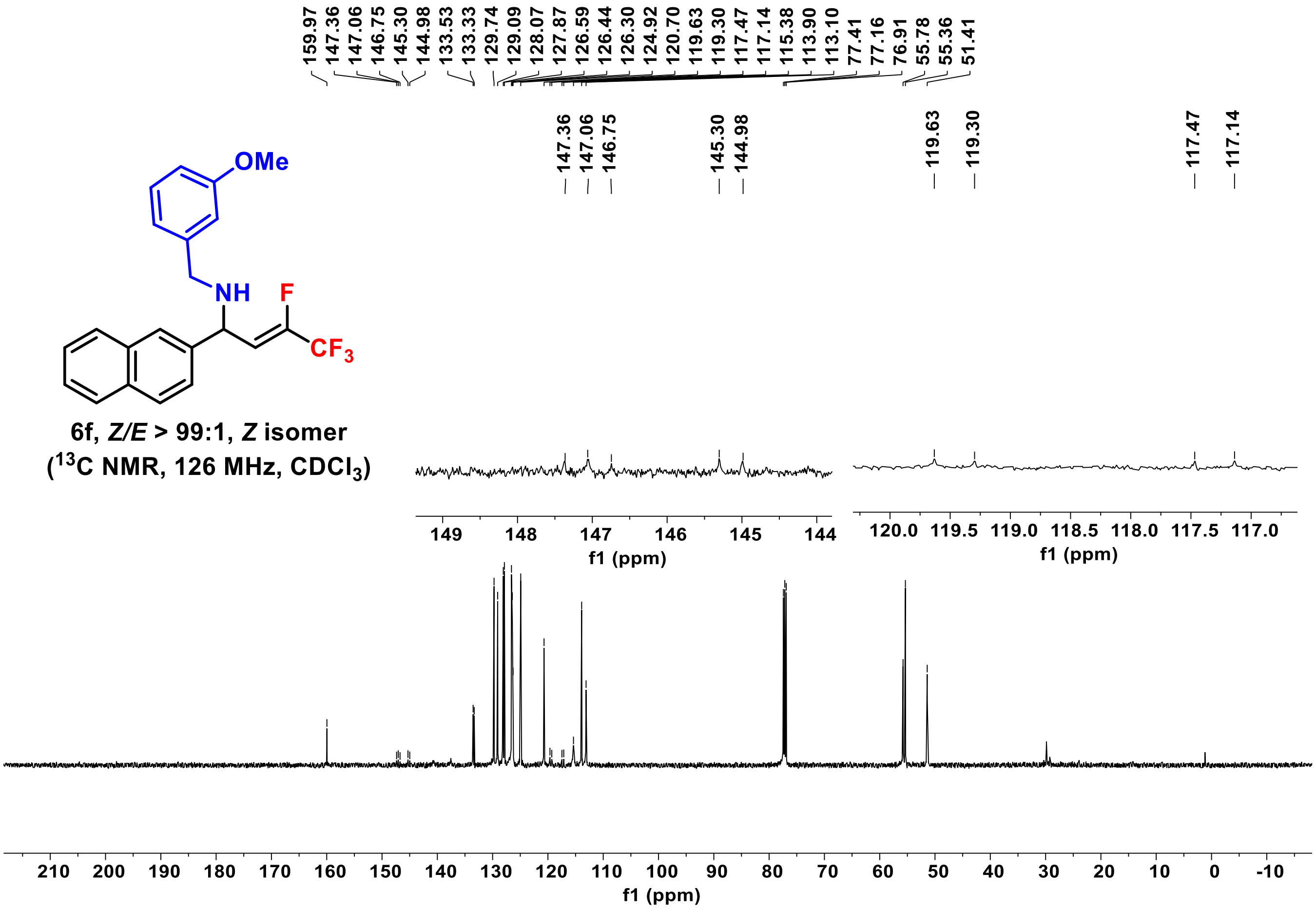
159.97 147.36 147.06 146.75 145.30 144.98 133.53 133.33 129.74 129.09 128.07 127.87 126.59 126.44 126.30 124.92 120.70 119.63 119.30 117.47 117.14 115.38 113.90 113.10 77.41 77.16 76.91 55.78 55.36 51.41
147.36 147.06 146.75 145.30 144.98
119.63 119.30 117.47 117.14
OMe
NH
F
CF3
6f, Z/E > 99:1, Z isomer
(13C NMR, 126 MHz, CDCl3)
f1 (ppm)
f1 (ppm)
f1 (ppm)

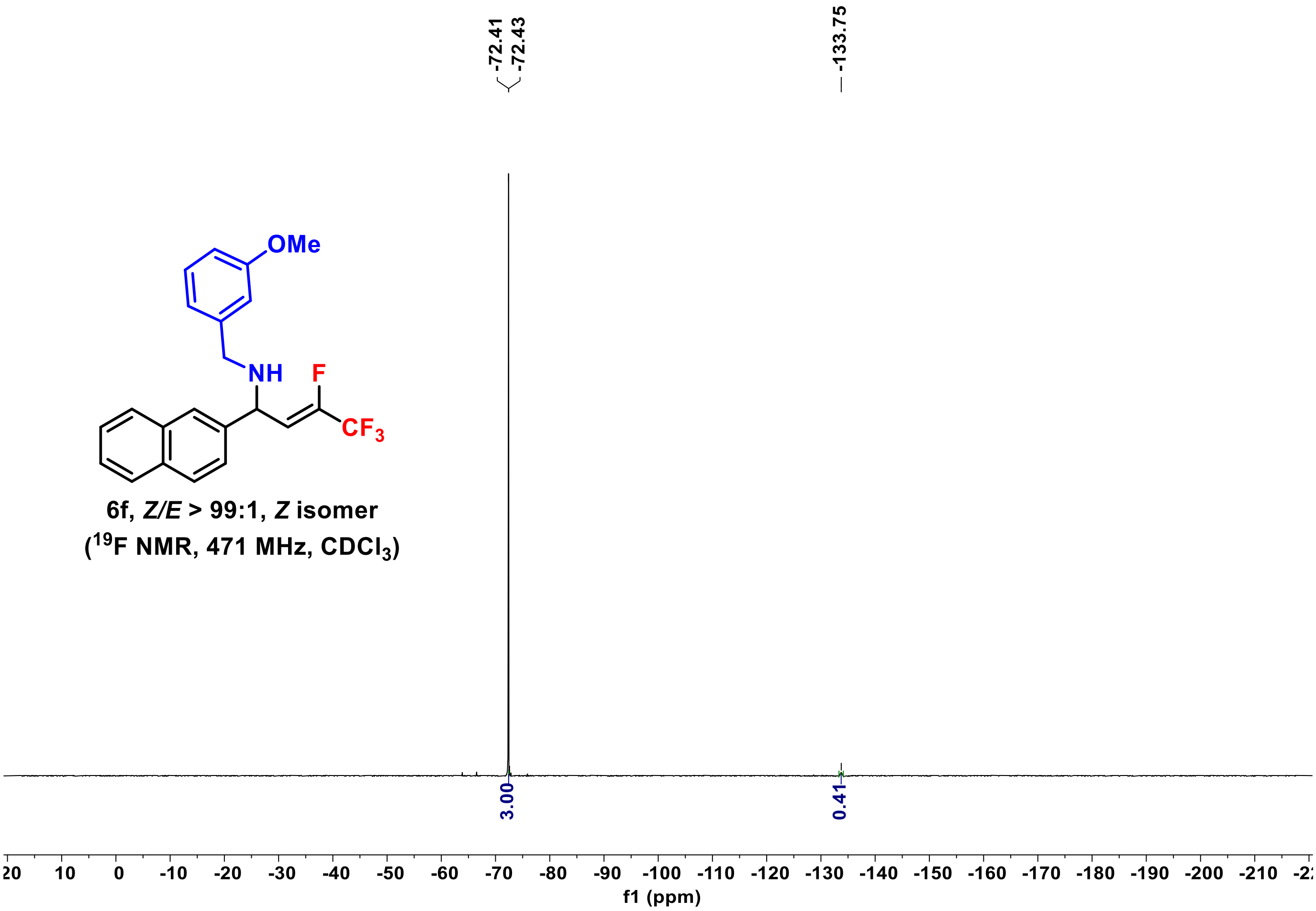

6f, *Z/E* > 99:1, *Z* isomer
(^{19}F NMR, 471 MHz, $CDCl_3$)

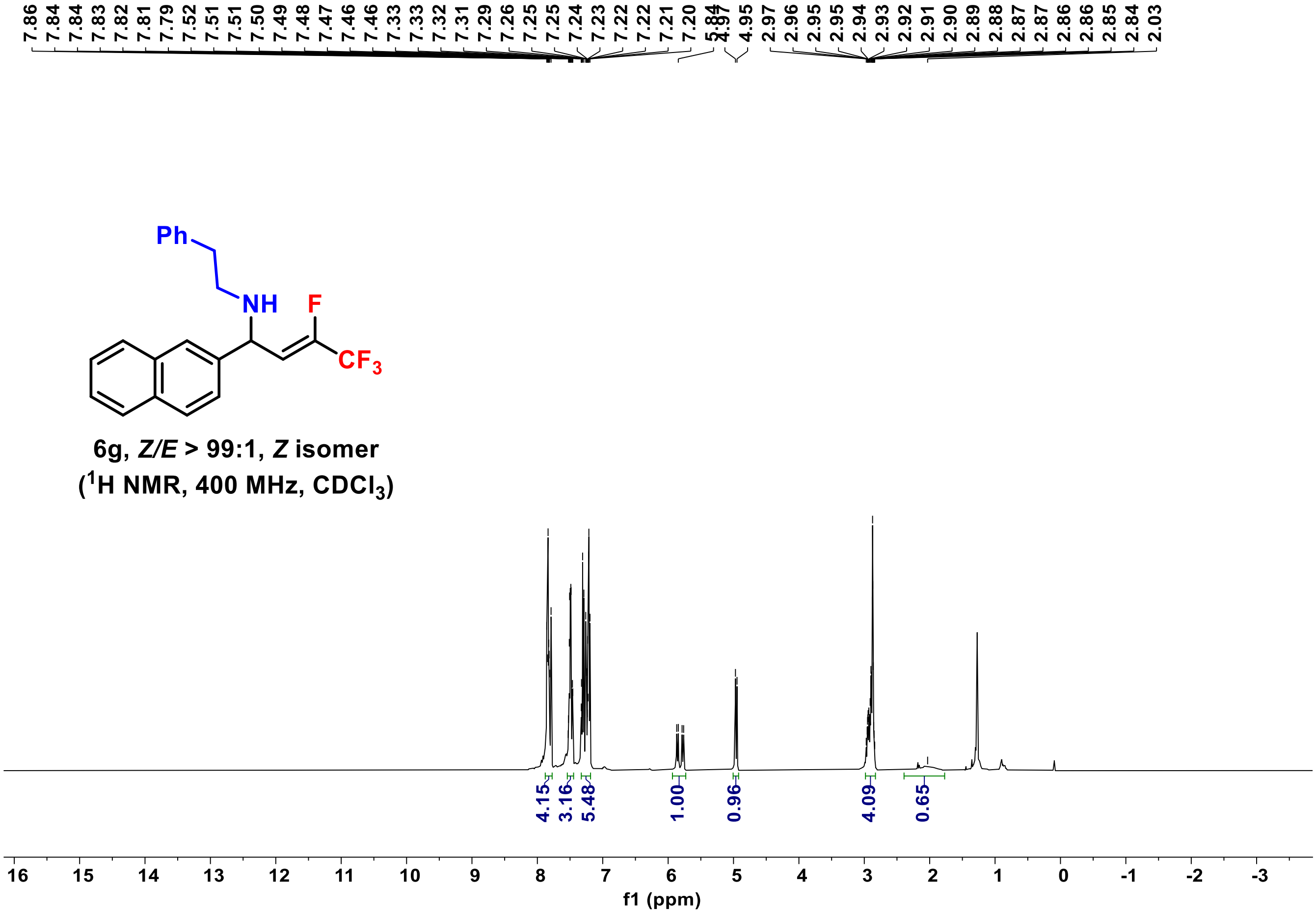

6g, *Z/E* > 99:1, *Z* isomer
(^{1}H NMR, 400 MHz, $CDCl_3$)

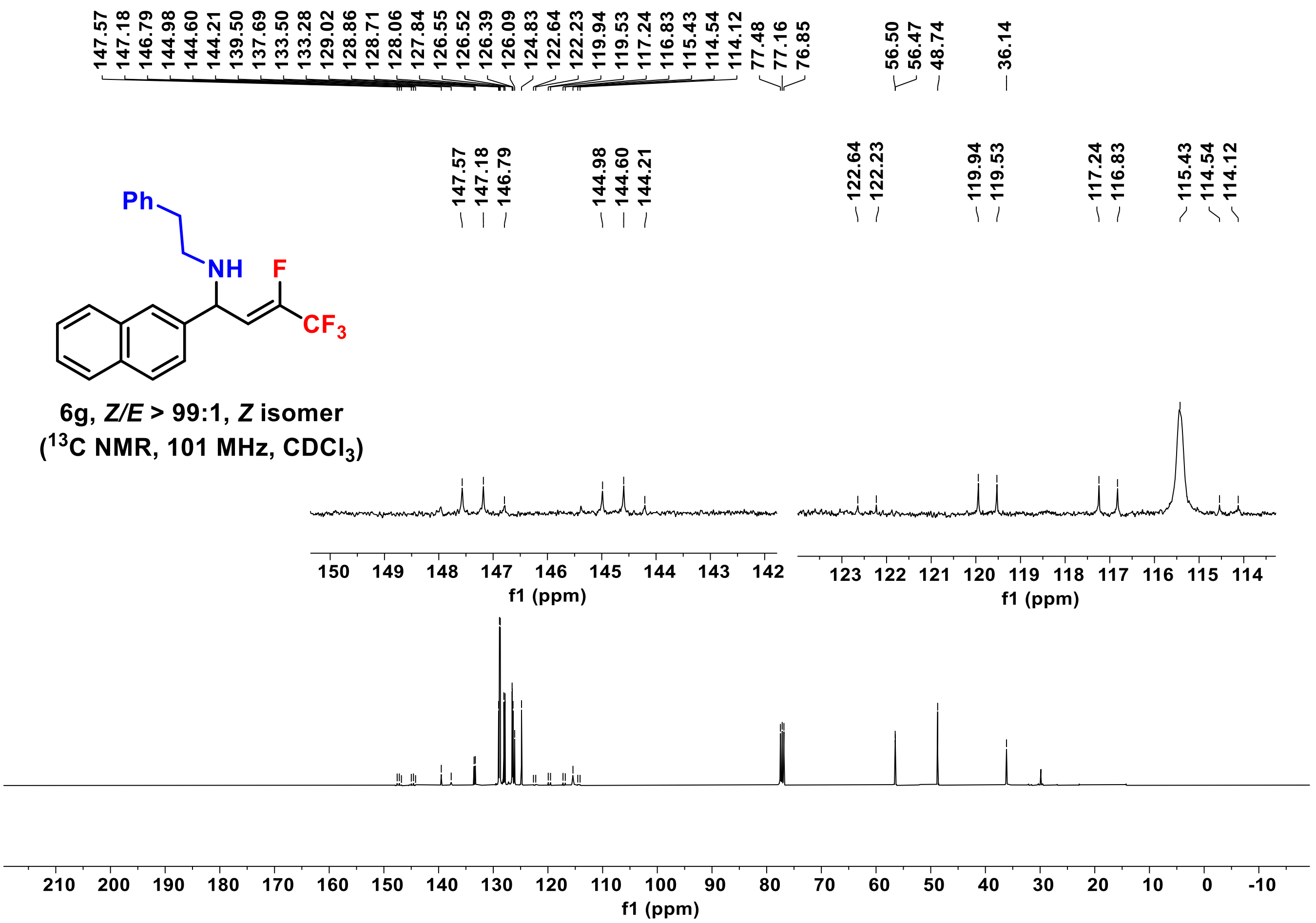
147.57 147.18 146.79 144.98 144.60 144.21 139.50 137.69 133.50 133.28 129.02 128.86 128.71 128.06 127.84 126.55 126.52 126.39 126.09 124.83 122.64 122.23 119.94 119.53 117.24 116.83 115.43 114.54 114.12 77.48 77.16 76.85 56.50 56.47 48.74 36.14
Ph
NH
F
CF_3
6g, *Z/E* > 99:1, *Z* isomer
(^{13}C NMR, 101 MHz, $CDCl_3$)
f1 (ppm)

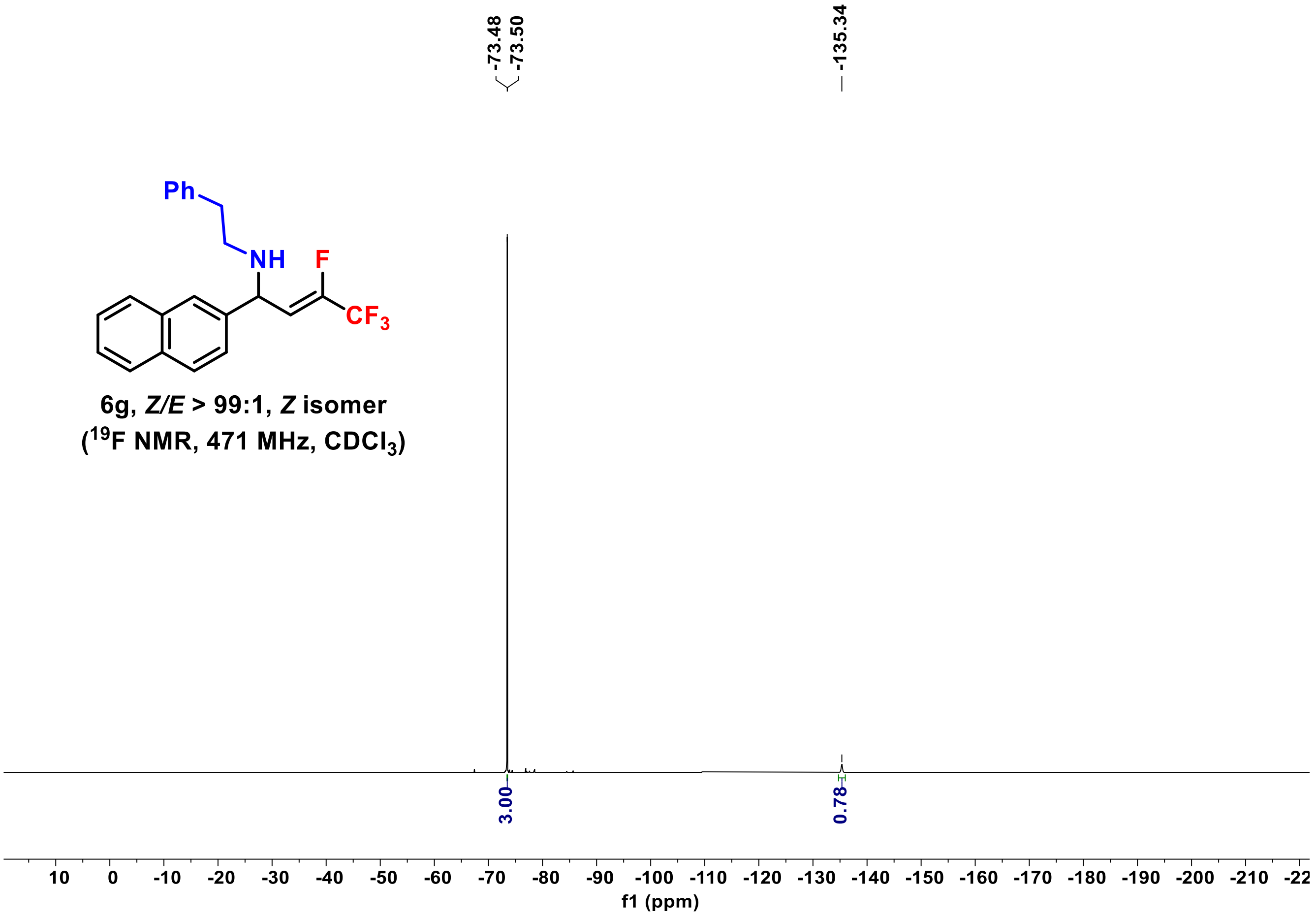
-73.48 -73.50 -135.34
Ph
NH
F
CF_3
6g, *Z/E* > 99:1, *Z* isomer
(^{19}F NMR, 471 MHz, $CDCl_3$)
3.00
0.78
f1 (ppm)

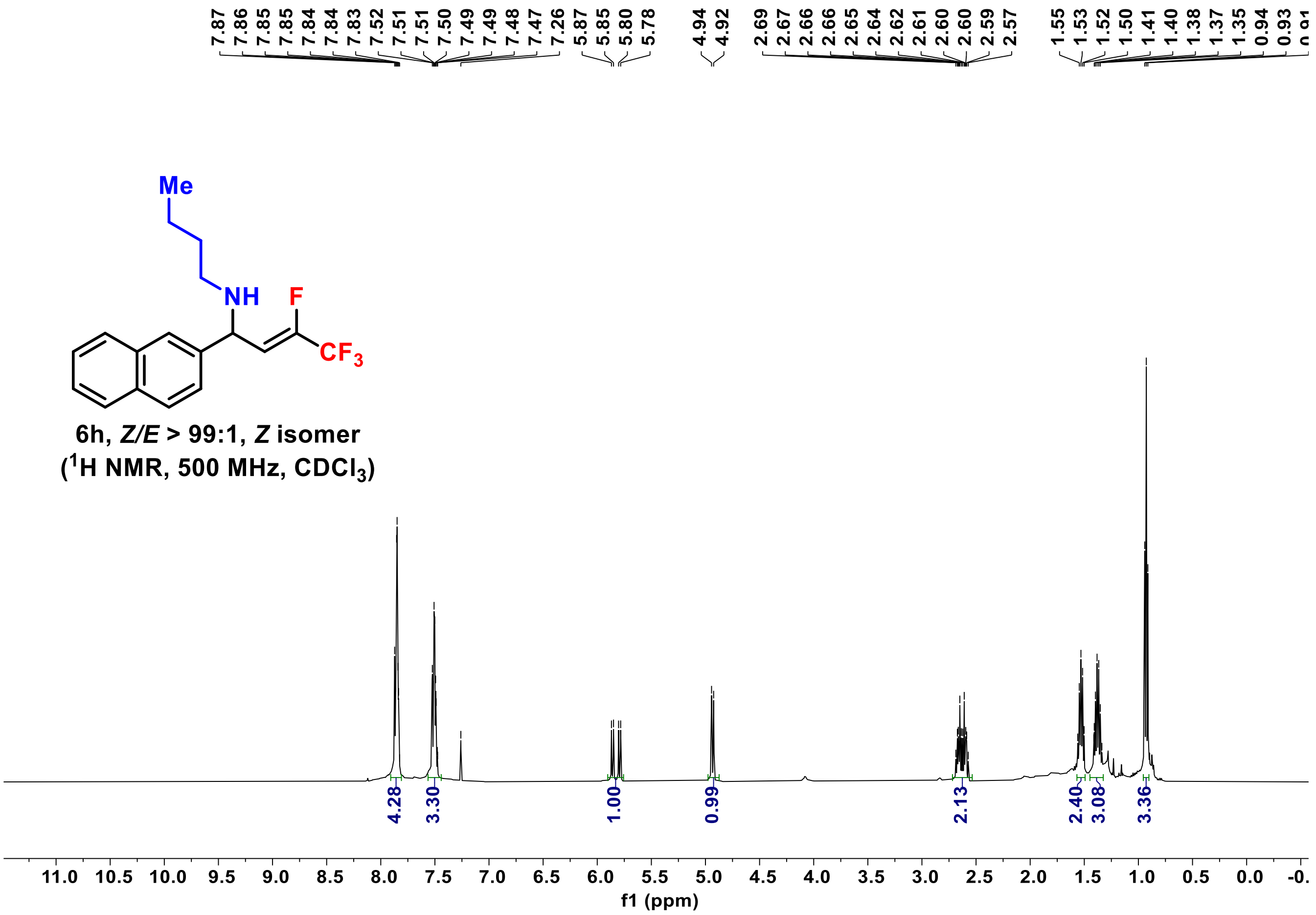

6h, *Z/E* > 99:1, *Z* isomer
(^{1}H NMR, 500 MHz, $CDCl_3$)

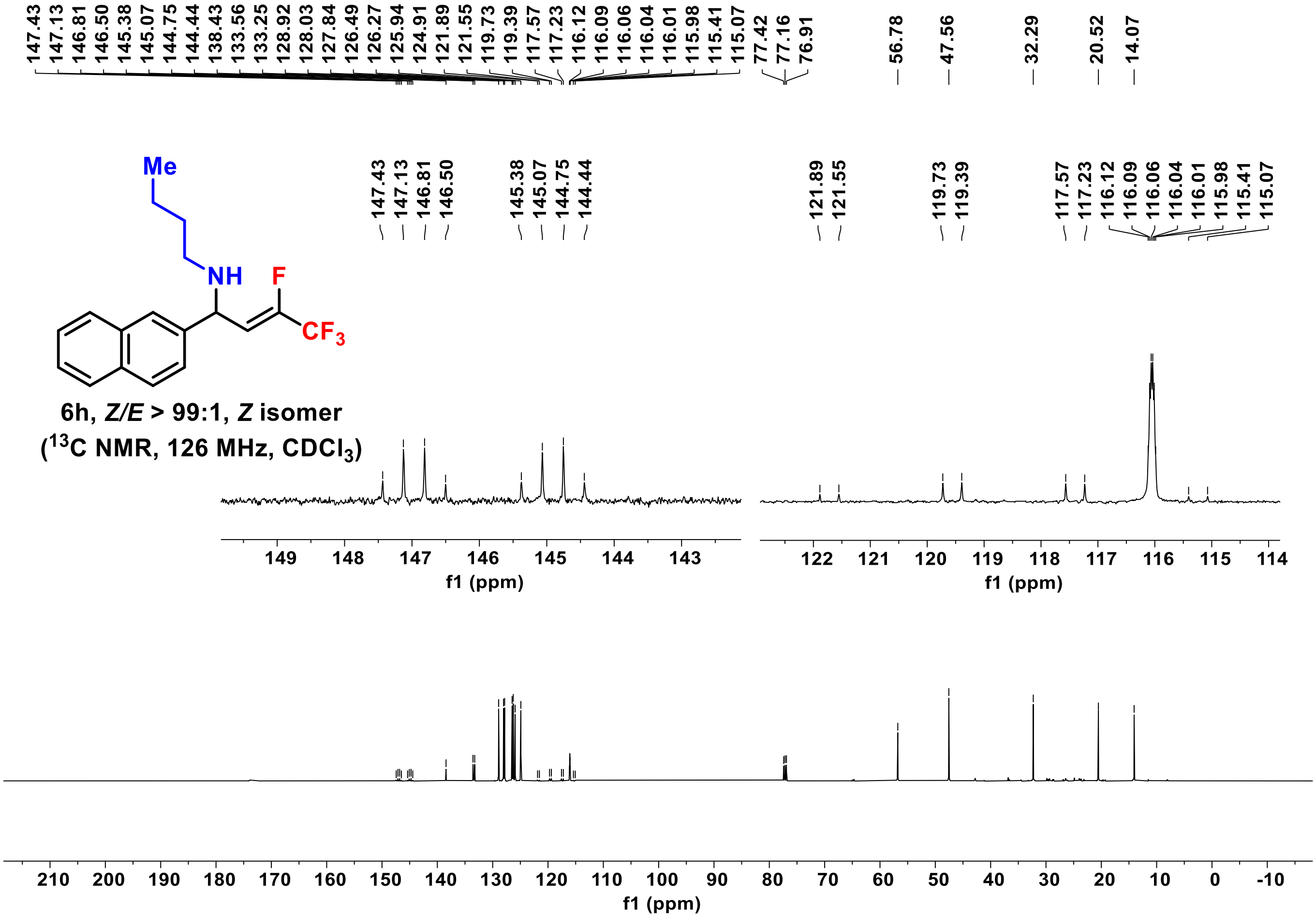

6h, *Z/E* > 99:1, *Z* isomer
(^{13}C NMR, 126 MHz, $CDCl_3$)

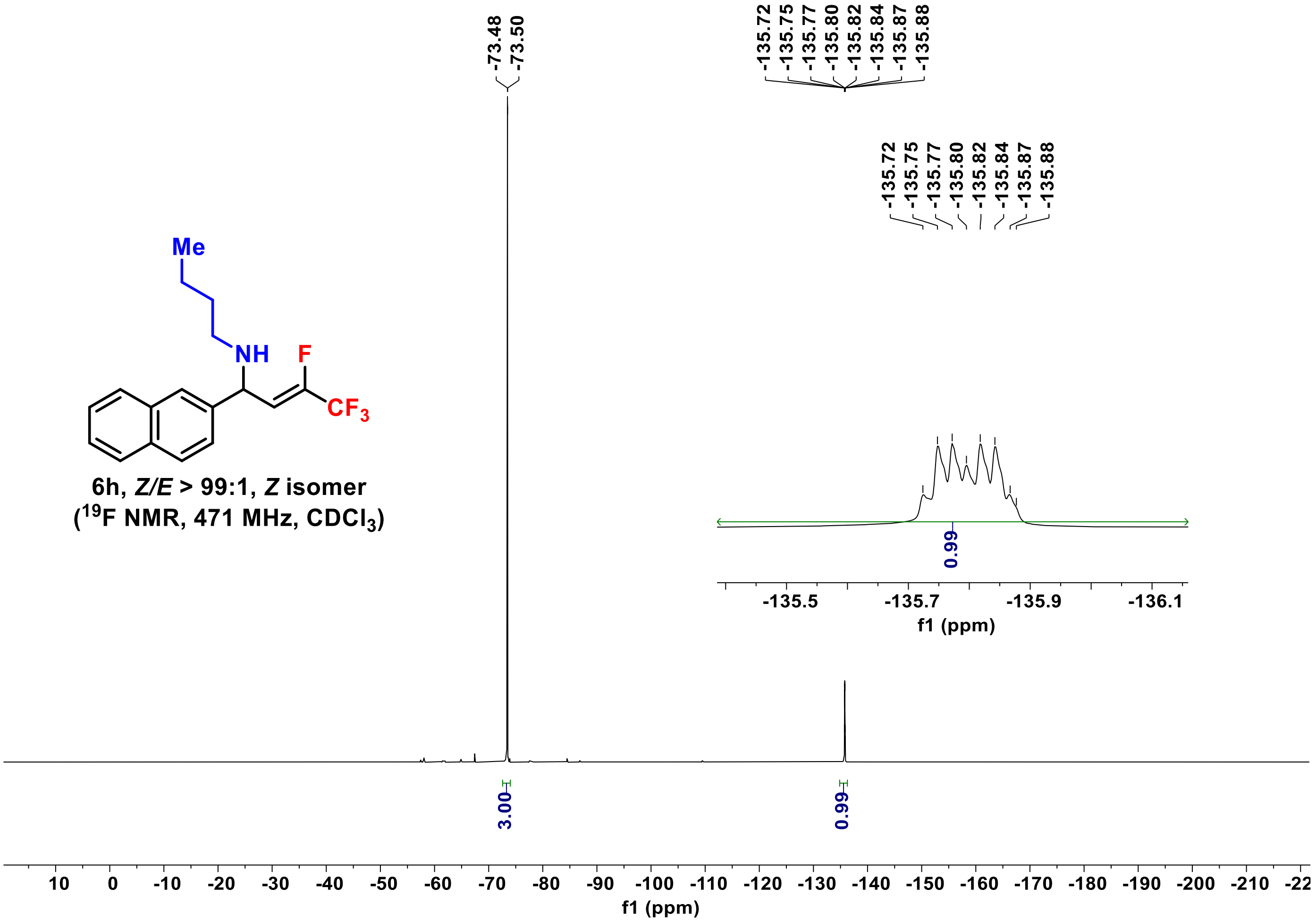

6h, *Z/E* > 99:1, *Z* isomer
(^{19}F NMR, 471 MHz, CDCl$_3$)

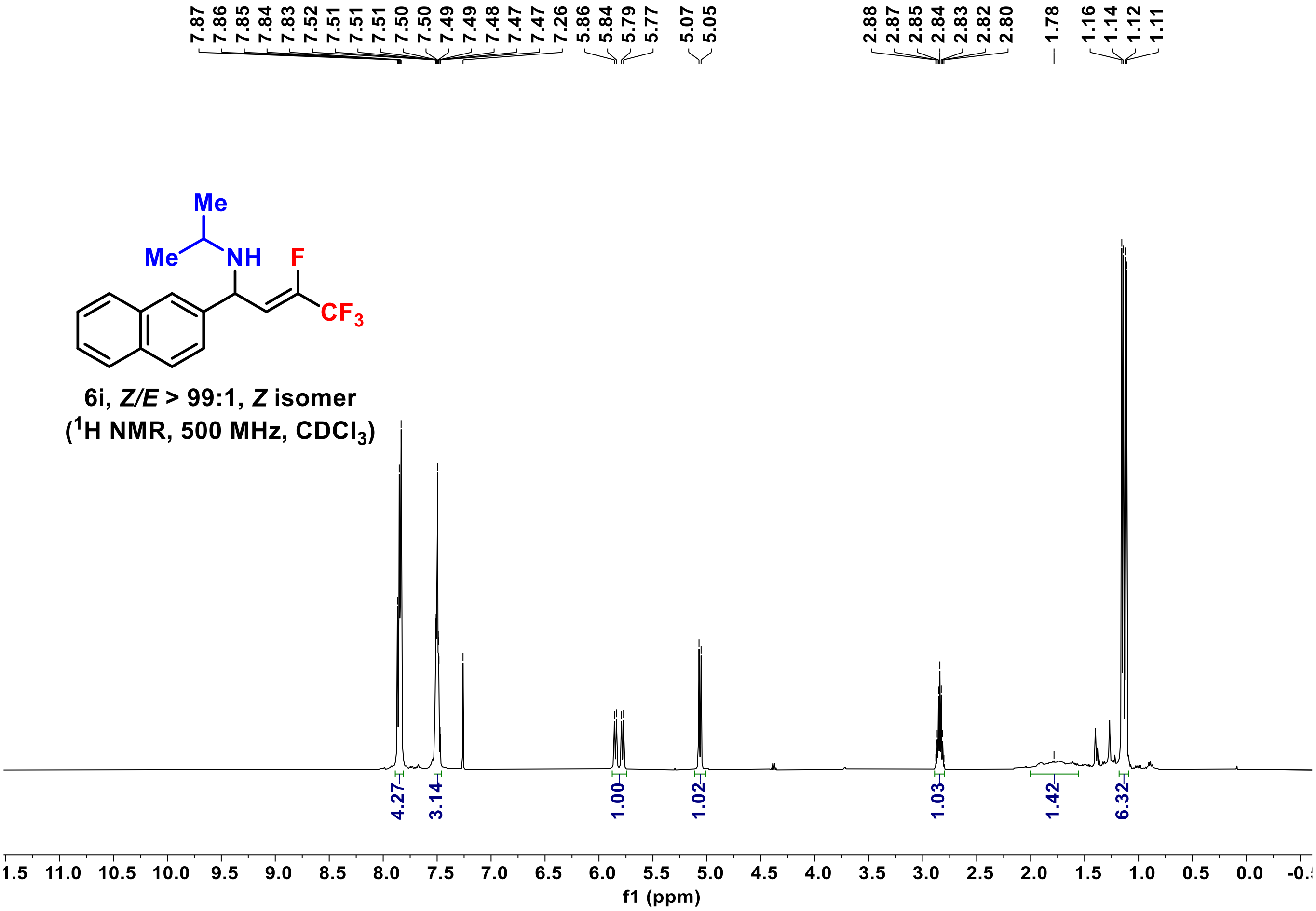

6i, *Z/E* > 99:1, *Z* isomer
(^{1}H NMR, 500 MHz, CDCl$_3$)

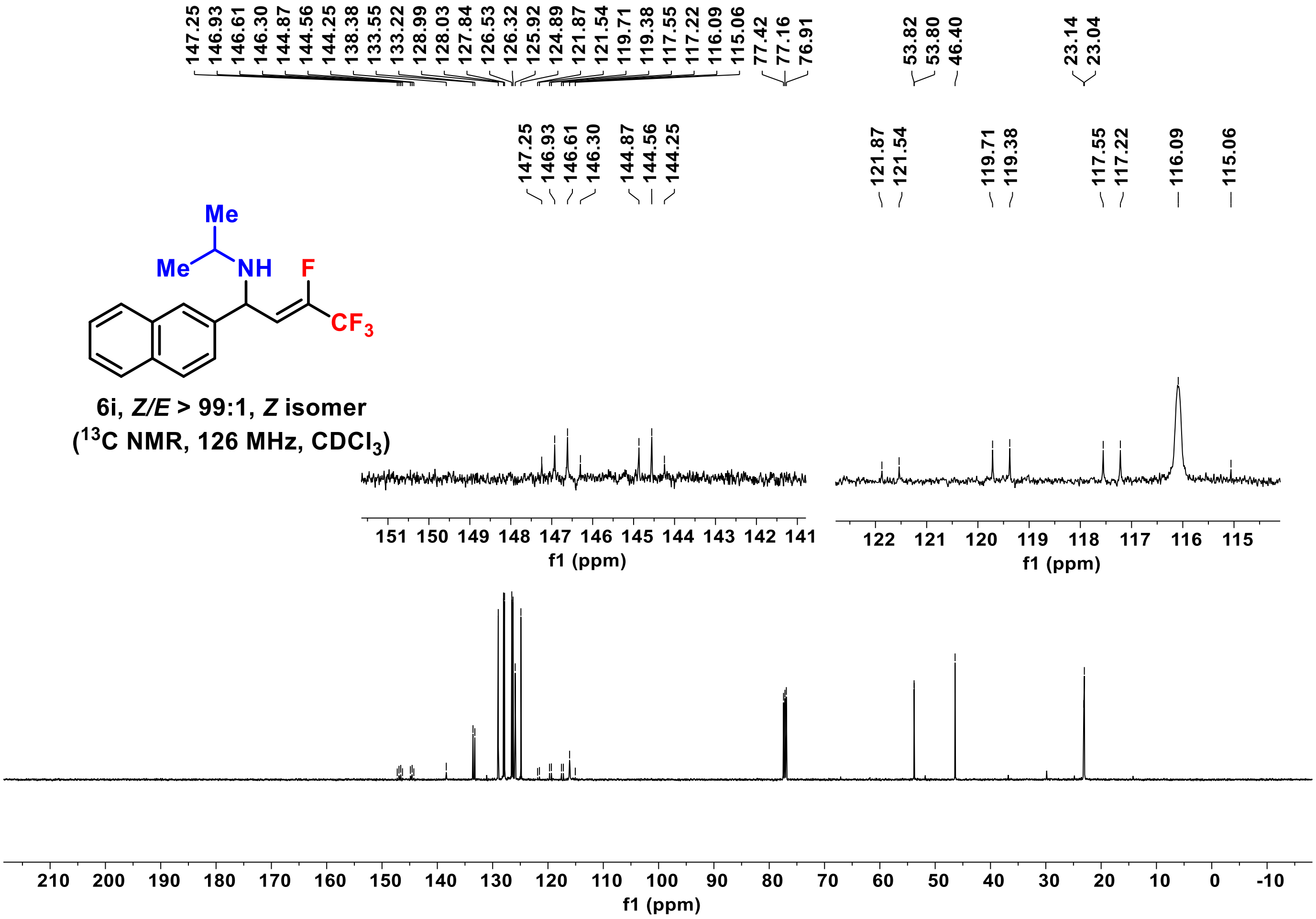

6i, *Z/E* > 99:1, *Z* isomer

(^{13}C NMR, 126 MHz, $CDCl_3$)

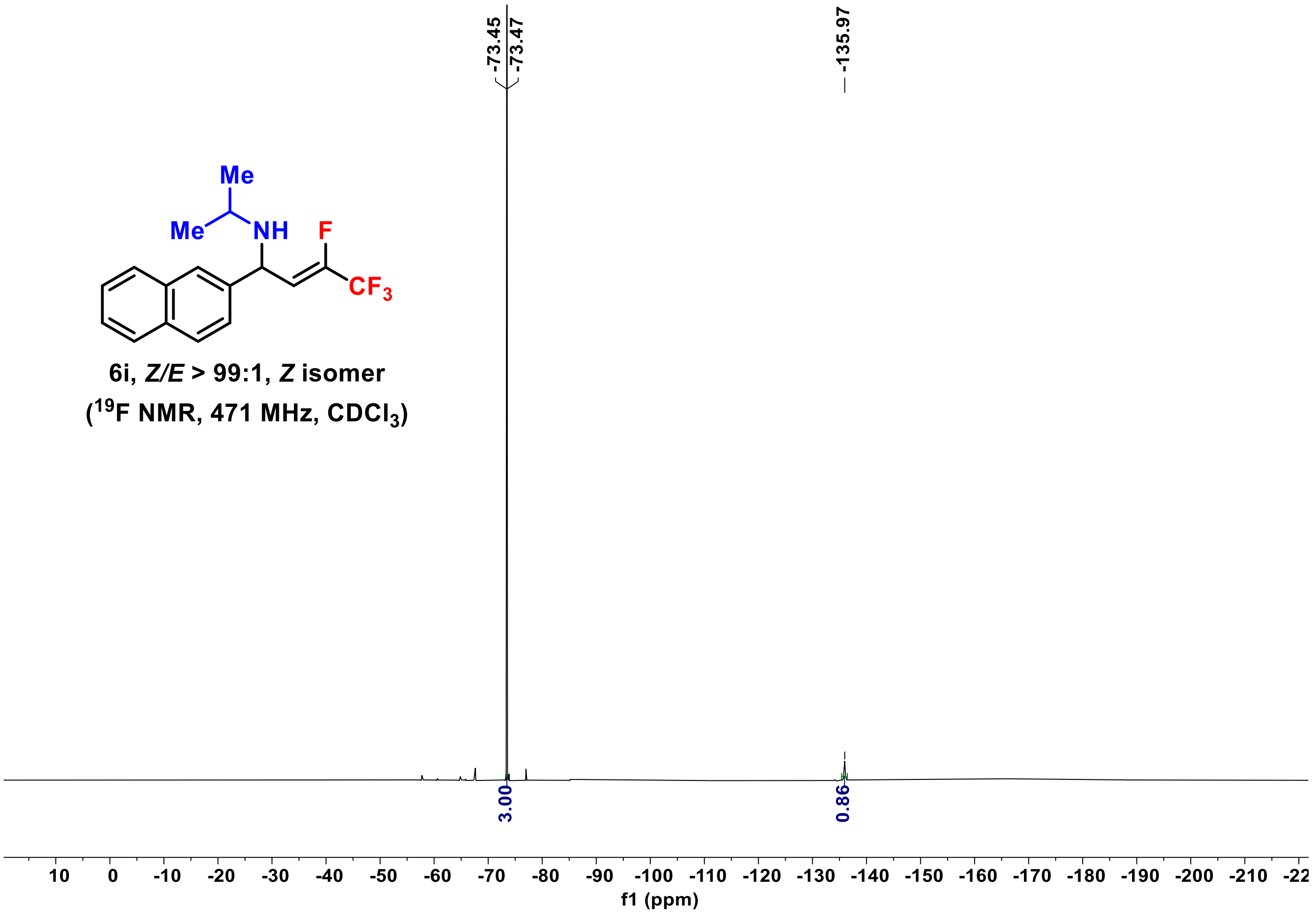

6i, *Z/E* > 99:1, *Z* isomer

(^{19}F NMR, 471 MHz, $CDCl_3$)

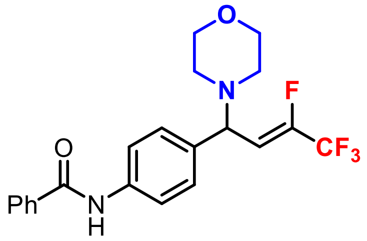

6j, *Z/E* > 99:1, *Z* isomer
(^{1}H NMR, 500 MHz, $CDCl_3$)

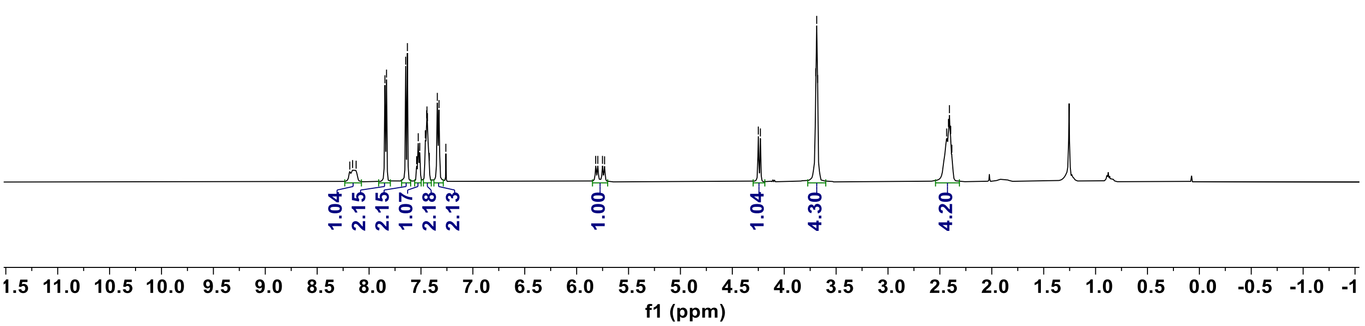

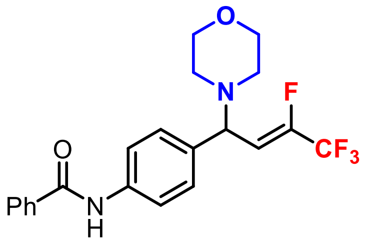

6j, *Z/E* > 99:1, *Z* isomer
(^{13}C NMR, 126 MHz, $CDCl_3$)

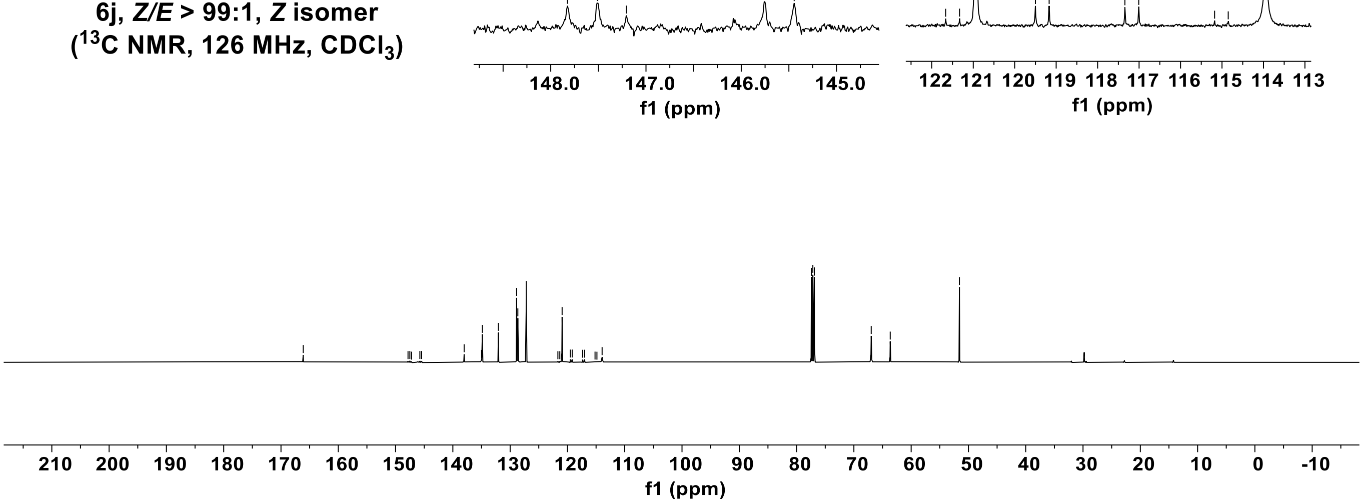

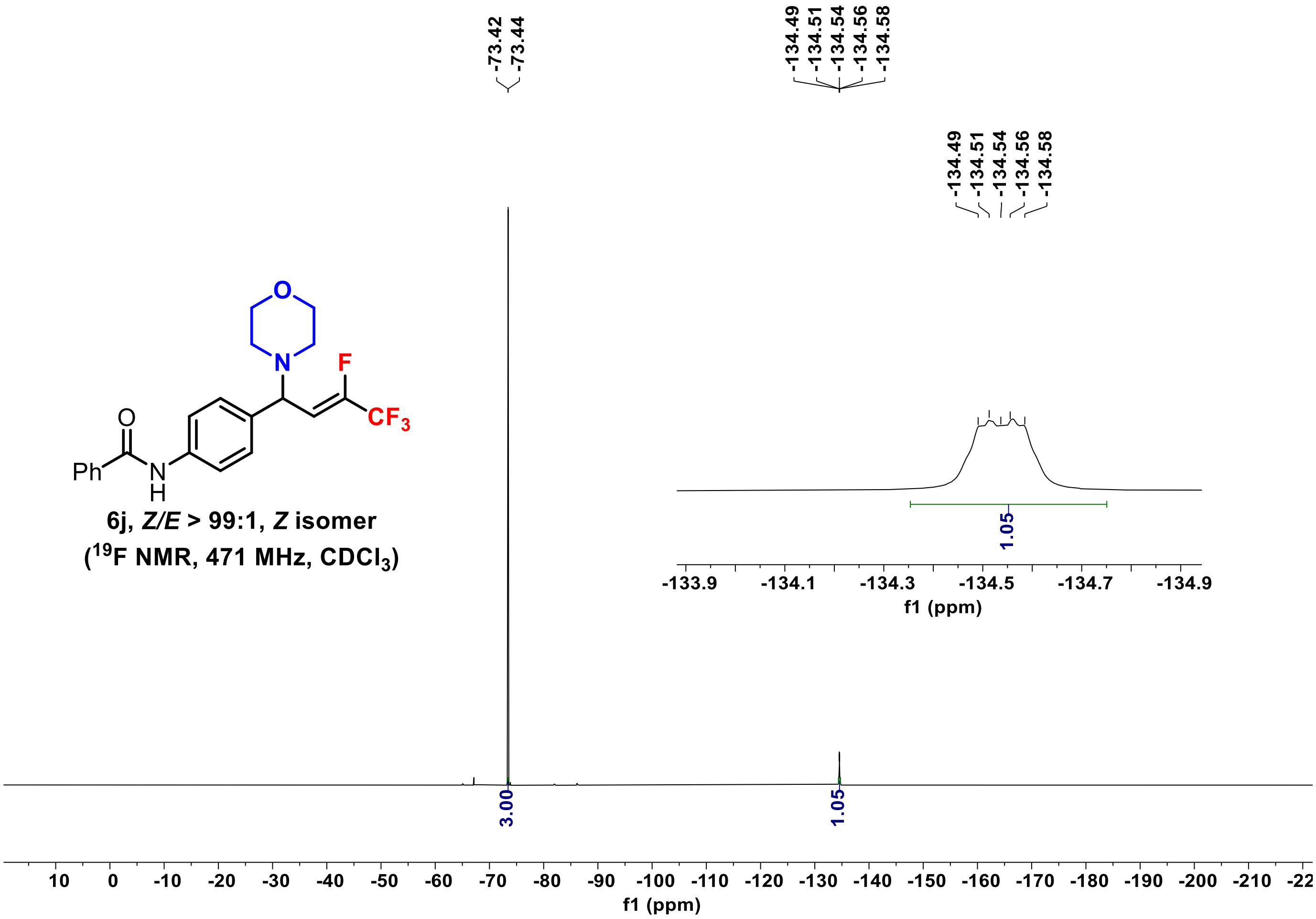
-73.42
-73.44
-134.49
-134.51
-134.54
-134.56
-134.58
O
N
F
CF3
Ph
N
H
6j, Z/E > 99:1, Z isomer
(19F NMR, 471 MHz, CDCl3)
1.05
-133.9 -134.1 -134.3 -134.5 -134.7 -134.9
f1 (ppm)
3.00
1.05
10 0 -10 -20 -30 -40 -50 -60 -70 -80 -90 -100 -110 -120 -130 -140 -150 -160 -170 -180 -190 -200 -210 -22
f1 (ppm)

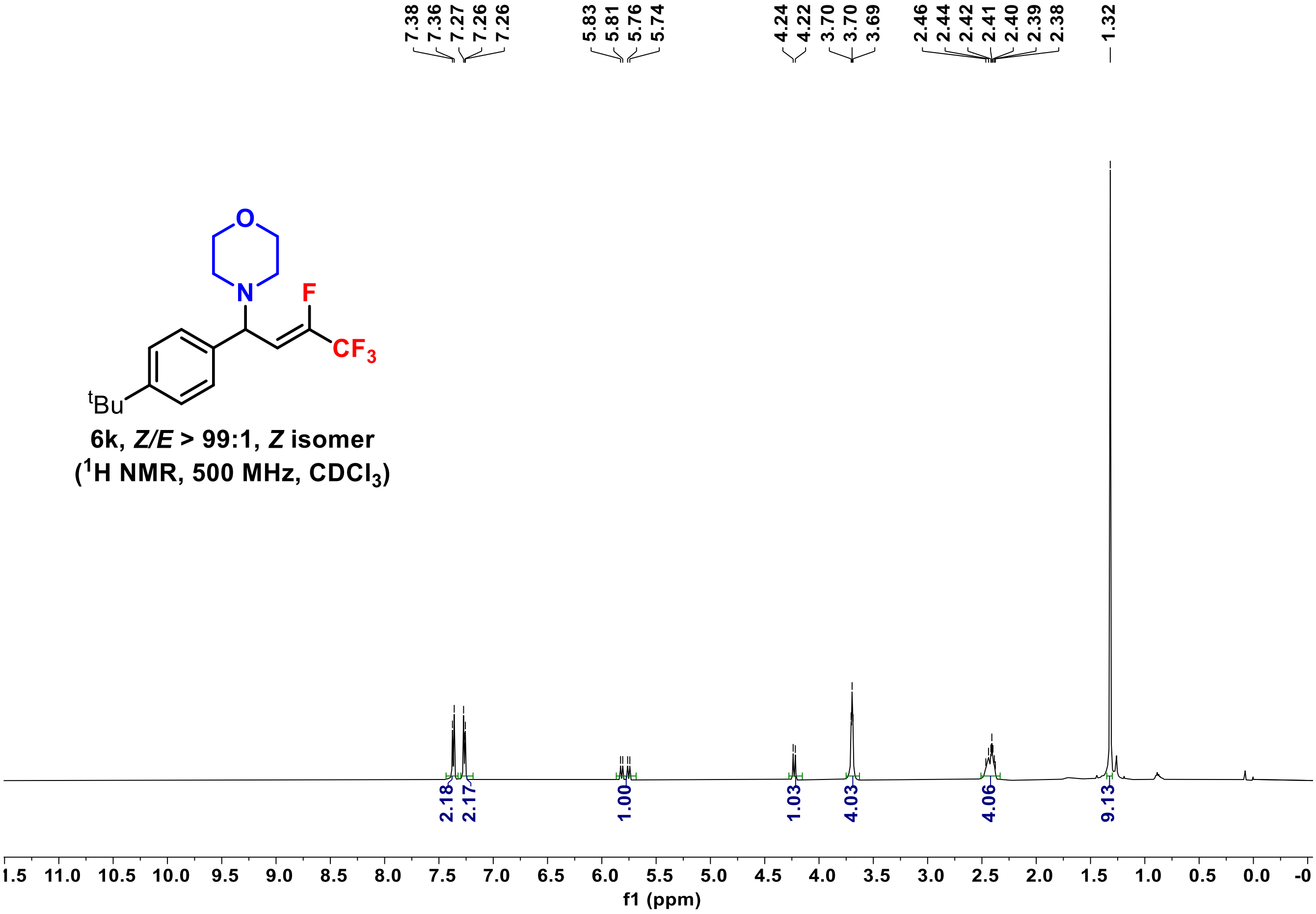
7.38
7.36
7.27
7.26
7.26
5.83
5.81
5.76
5.74
4.24
4.22
3.70
3.70
3.69
2.46
2.44
2.42
2.41
2.40
2.39
2.38
1.32
O
N
F
CF3
tBu
6k, Z/E > 99:1, Z isomer
(1H NMR, 500 MHz, CDCl3)
2.18
2.17
1.00
1.03
4.03
4.06
9.13
1.5 11.0 10.5 10.0 9.5 9.0 8.5 8.0 7.5 7.0 6.5 6.0 5.5 5.0 4.5 4.0 3.5 3.0 2.5 2.0 1.5 1.0 0.5 0.0 -0
f1 (ppm)

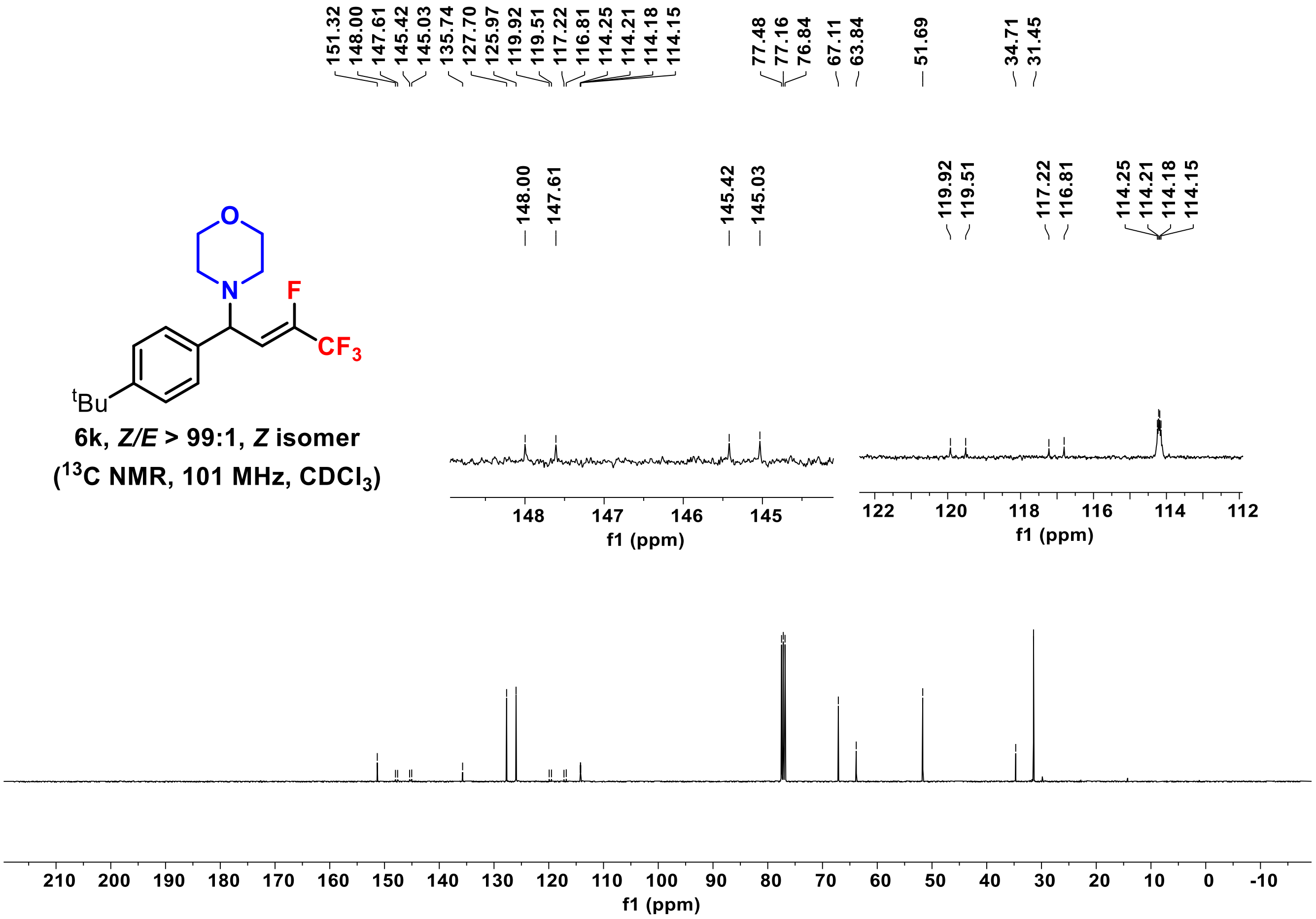
151.32
148.00
147.61
145.42
145.03
135.74
127.70
125.97
119.92
119.51
117.22
116.81
114.25
114.21
114.18
114.15
77.48
77.16
76.84
67.11
63.84
51.69
34.71
31.45
O
N
F
CF3
tBu
6k, Z/E > 99:1, Z isomer
(13C NMR, 101 MHz, CDCl3)
f1 (ppm)

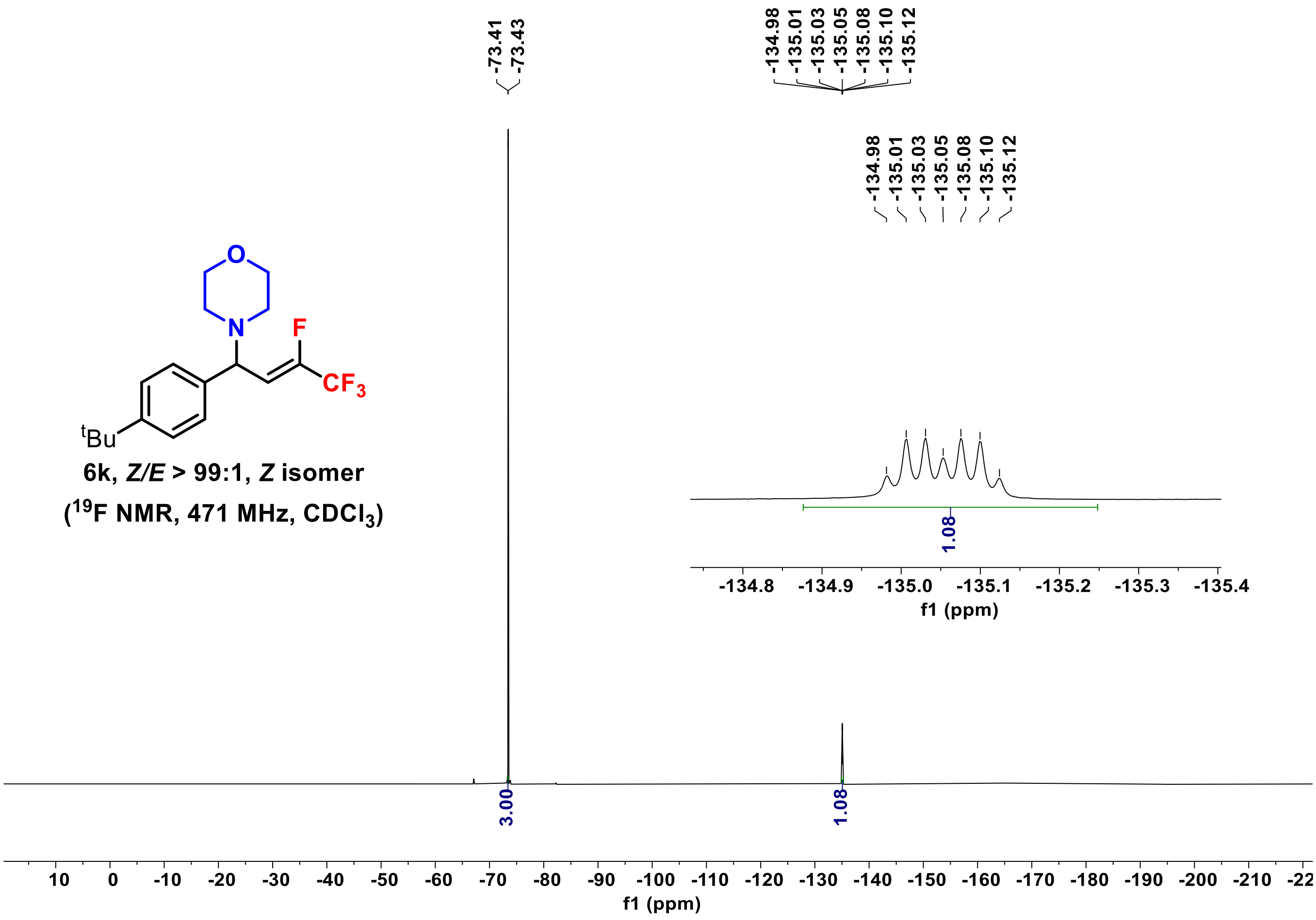
-73.41
-73.43
-134.98
-135.01
-135.03
-135.05
-135.08
-135.10
-135.12
O
N
F
CF3
tBu
6k, Z/E > 99:1, Z isomer
(19F NMR, 471 MHz, CDCl3)
1.08
3.00
f1 (ppm)

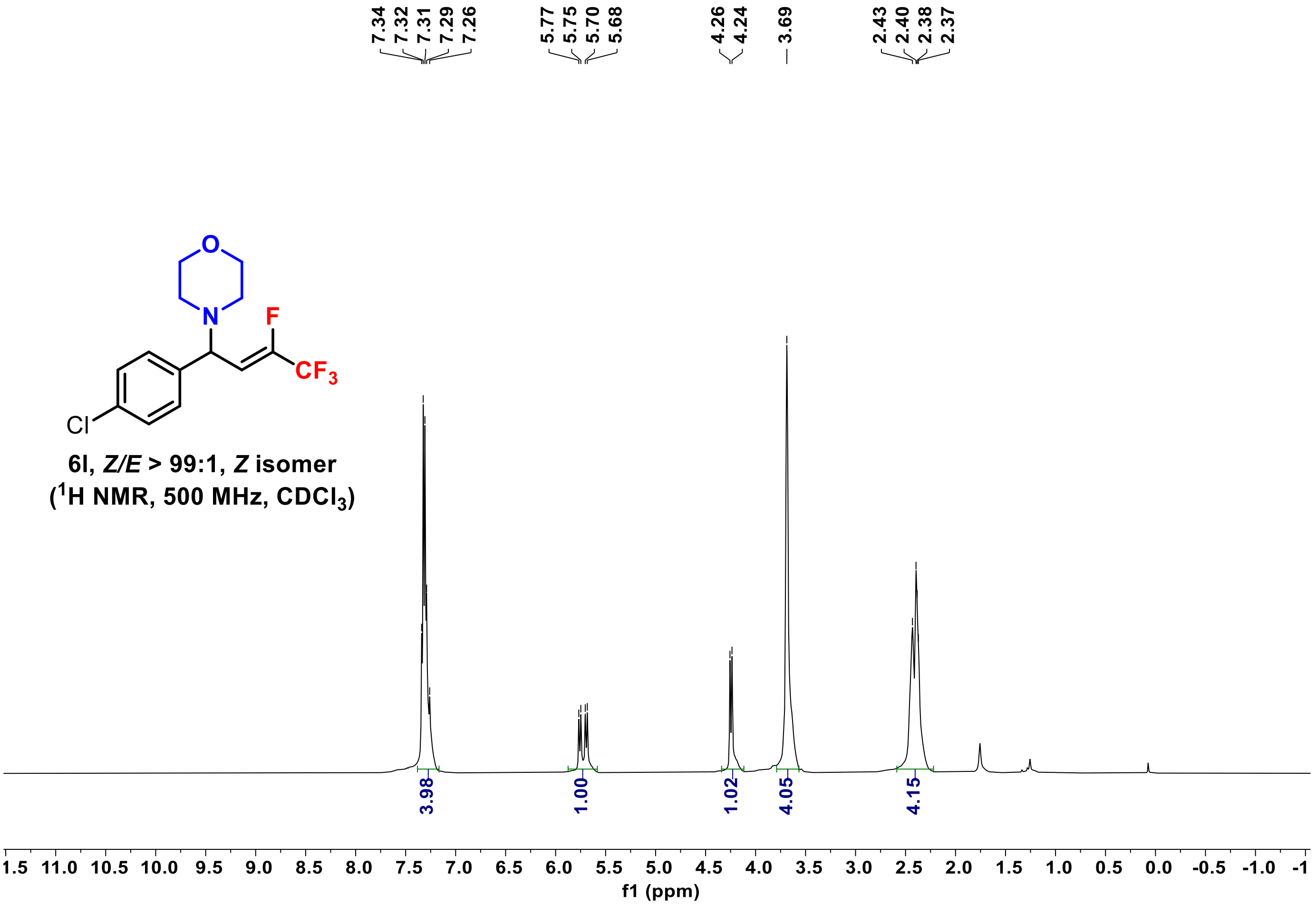
7.34 7.32 7.31 7.29 7.26
5.77 5.75 5.70 5.68
4.26 4.24
3.69
2.43 2.40 2.38 2.37
O
N
F
CF3
Cl
6l, Z/E > 99:1, Z isomer
(1H NMR, 500 MHz, CDCl3)
3.98
1.00
1.02
4.05
4.15
f1 (ppm)

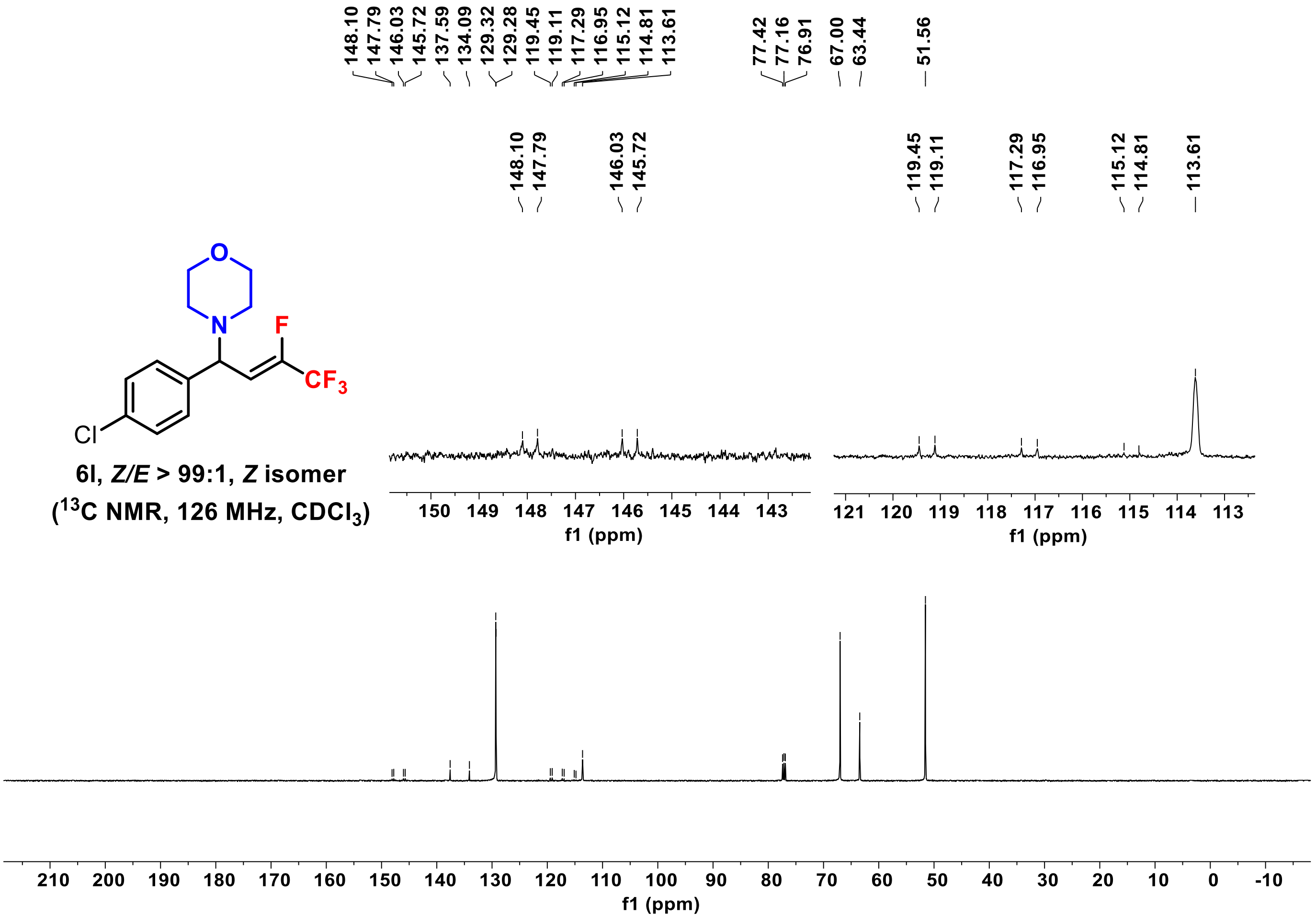
148.10 147.79 146.03 145.72 137.59 134.09 129.32 129.28 119.45 119.11 117.29 116.95 115.12 114.81 113.61
77.42 77.16 76.91
67.00
63.44
51.56
O
N
F
CF3
Cl
6l, Z/E > 99:1, Z isomer
(13C NMR, 126 MHz, CDCl3)
f1 (ppm)

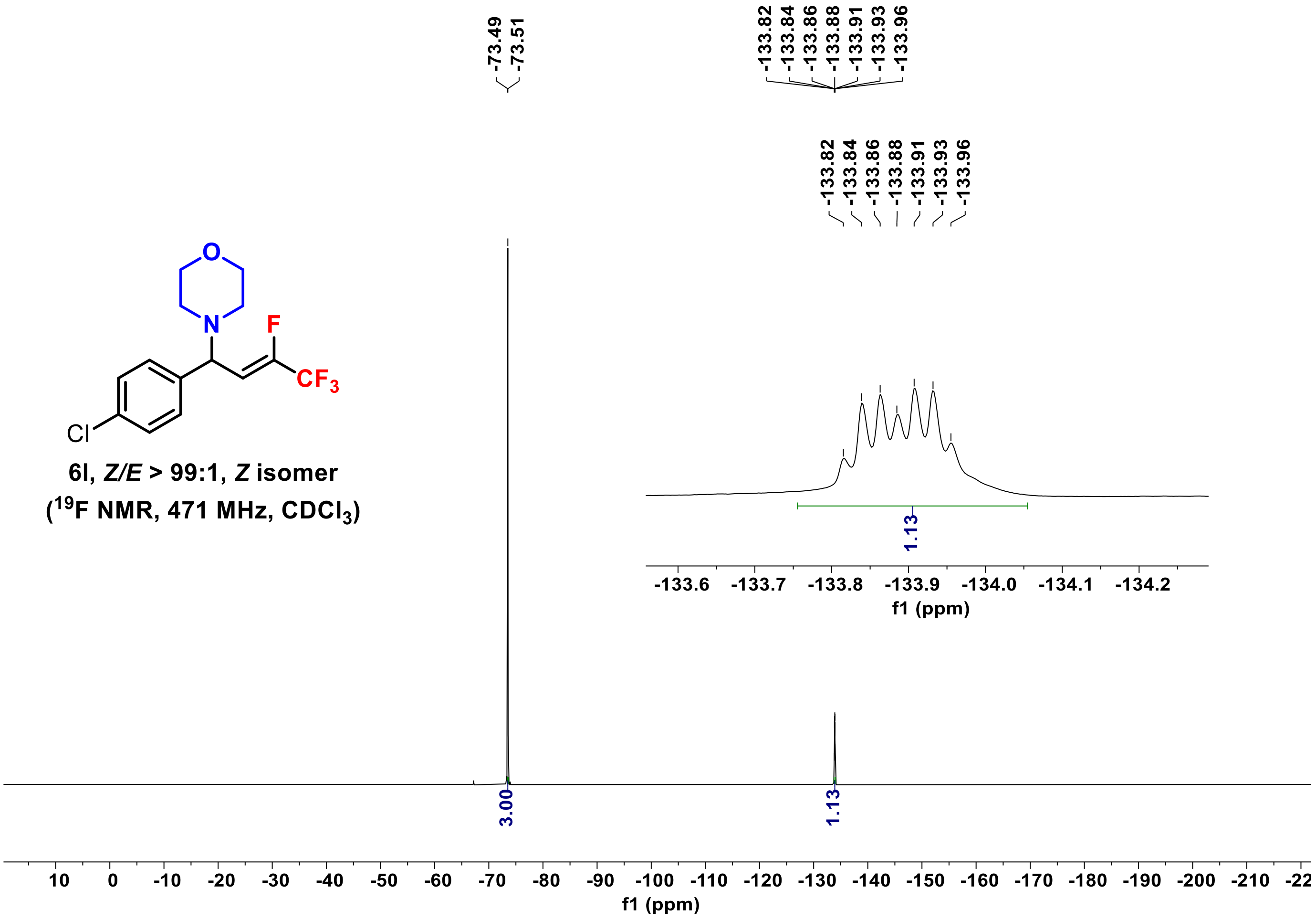

6l, *Z/E* > 99:1, *Z* isomer
(^{19}F NMR, 471 MHz, $CDCl_3$)

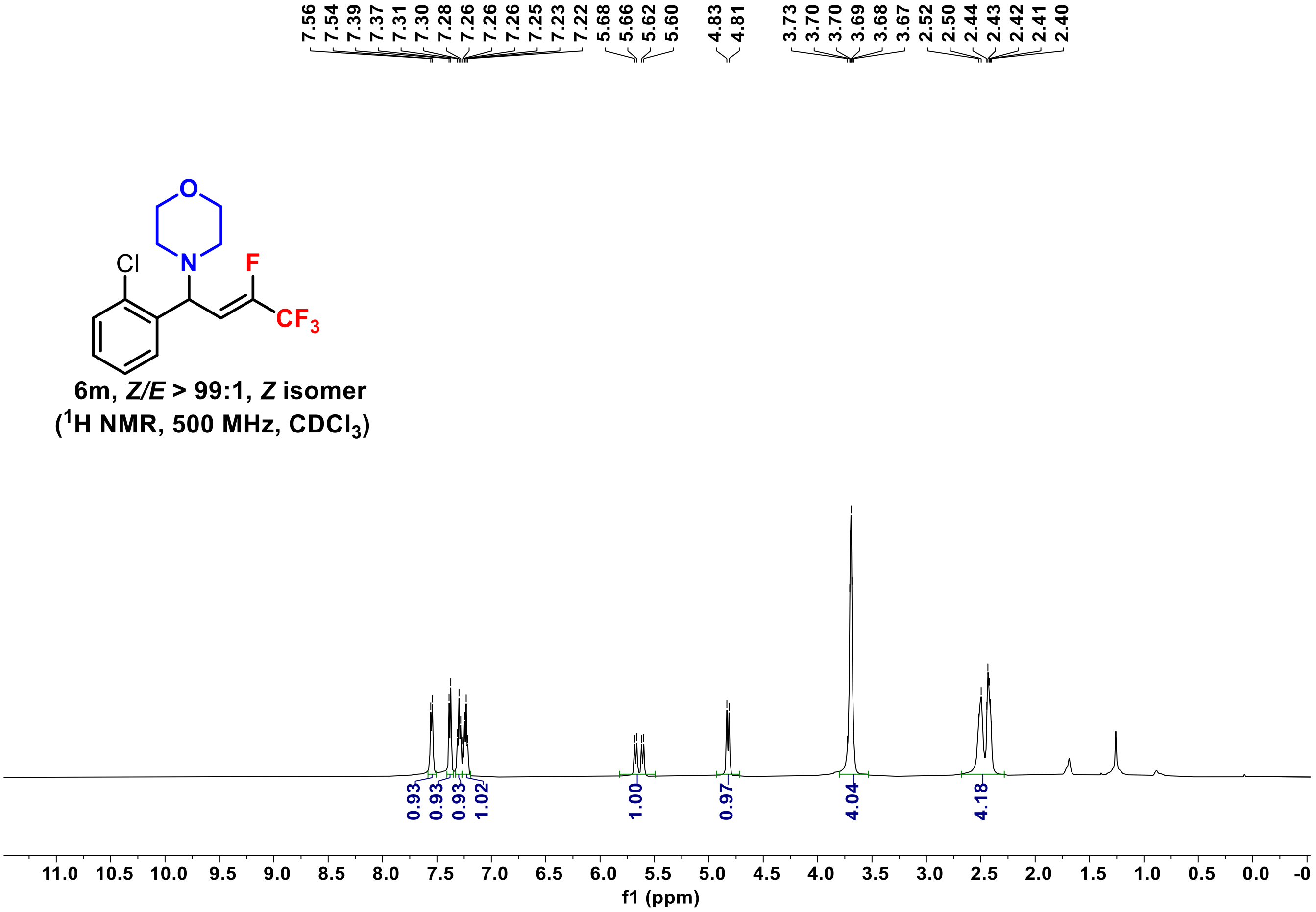

6m, *Z/E* > 99:1, *Z* isomer
(^{1}H NMR, 500 MHz, $CDCl_3$)

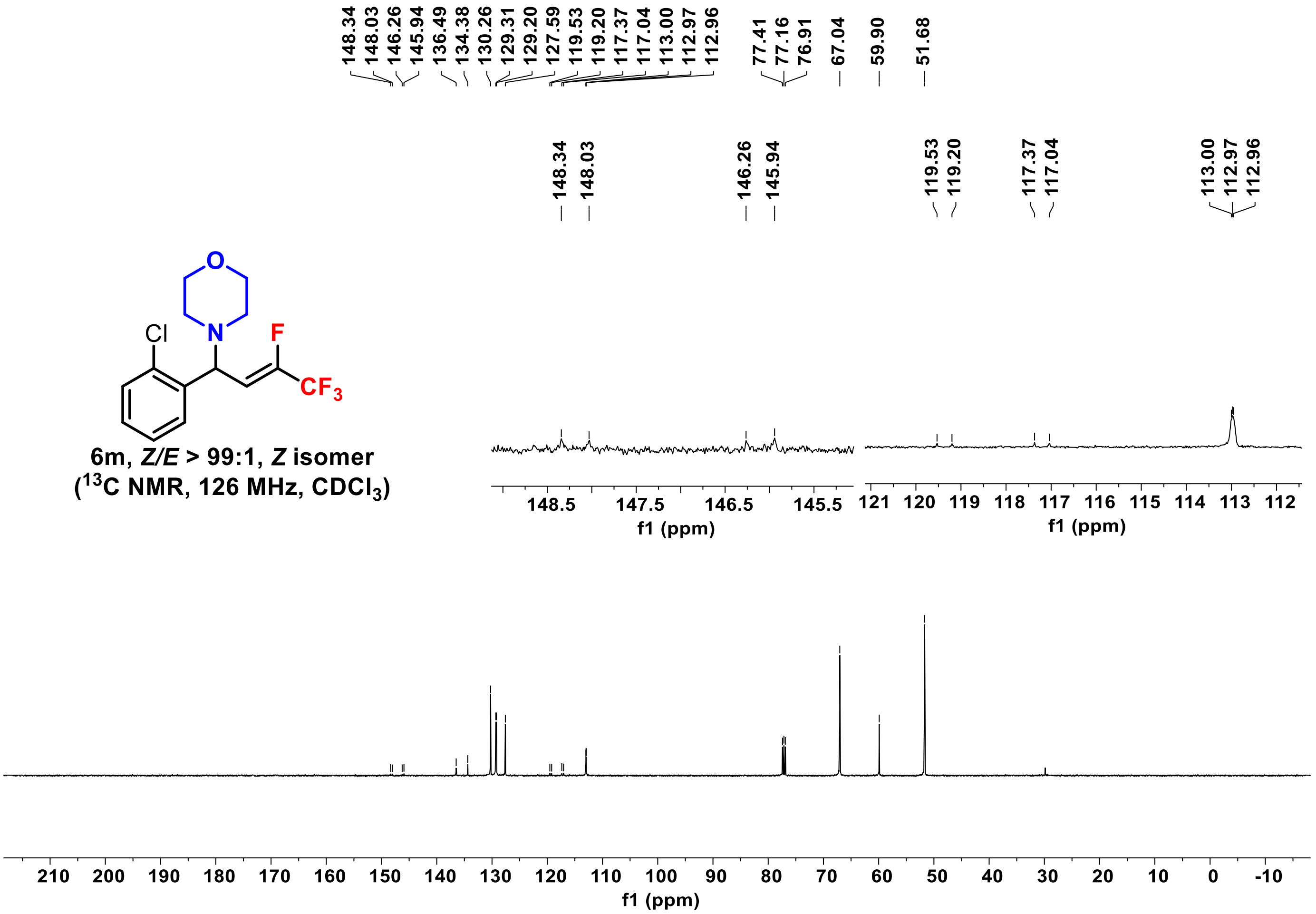
148.34
148.03
146.26
145.94
136.49
134.38
130.26
129.31
129.20
127.59
119.53
119.20
117.37
117.04
113.00
112.97
112.96
77.41
77.16
76.91
67.04
59.90
51.68
6m, Z/E > 99:1, Z isomer
(13C NMR, 126 MHz, CDCl3)
f1 (ppm)

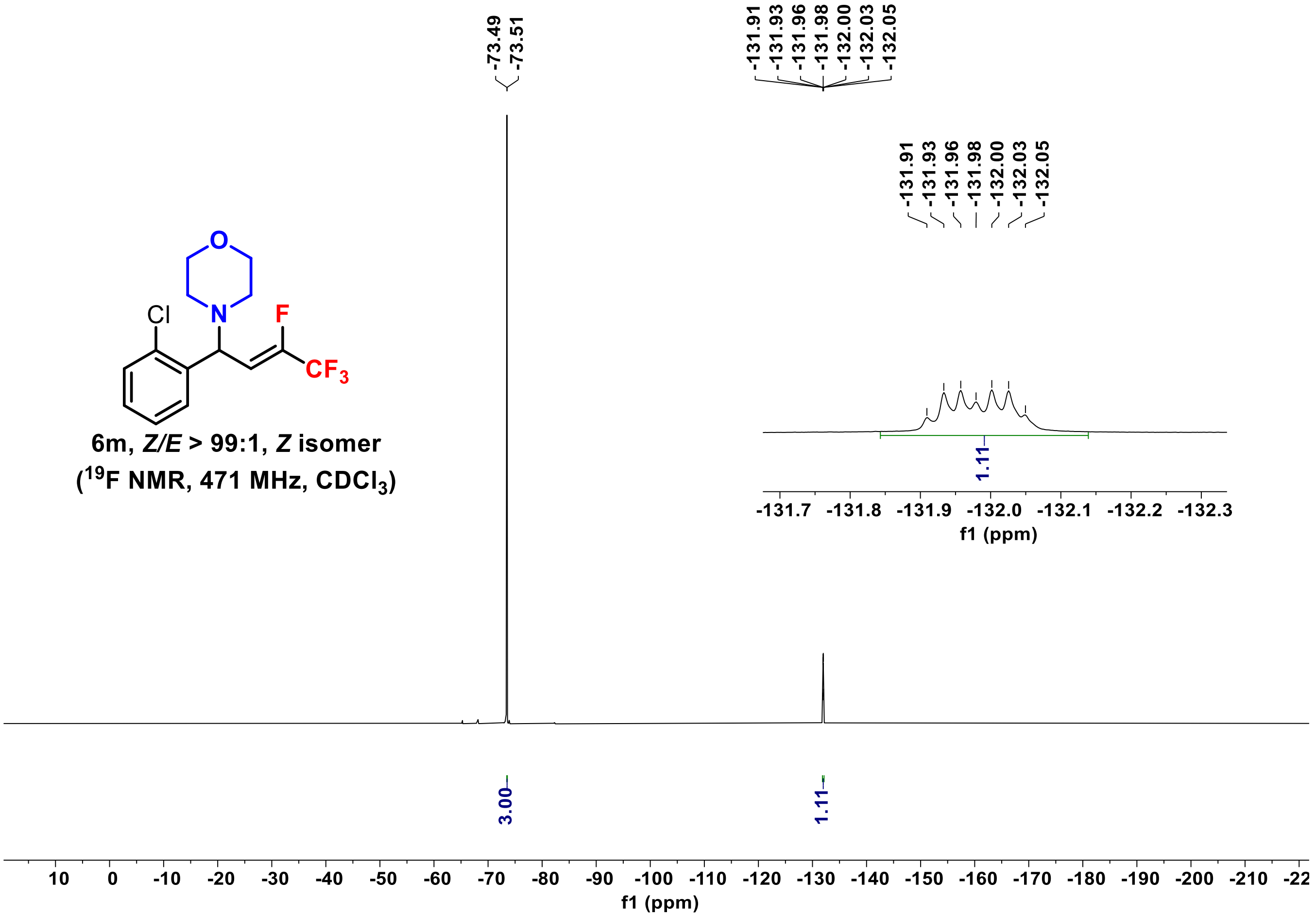
-73.49
-73.51
-131.91
-131.93
-131.96
-131.98
-132.00
-132.03
-132.05
6m, Z/E > 99:1, Z isomer
(19F NMR, 471 MHz, CDCl3)
1.11
3.00
f1 (ppm)

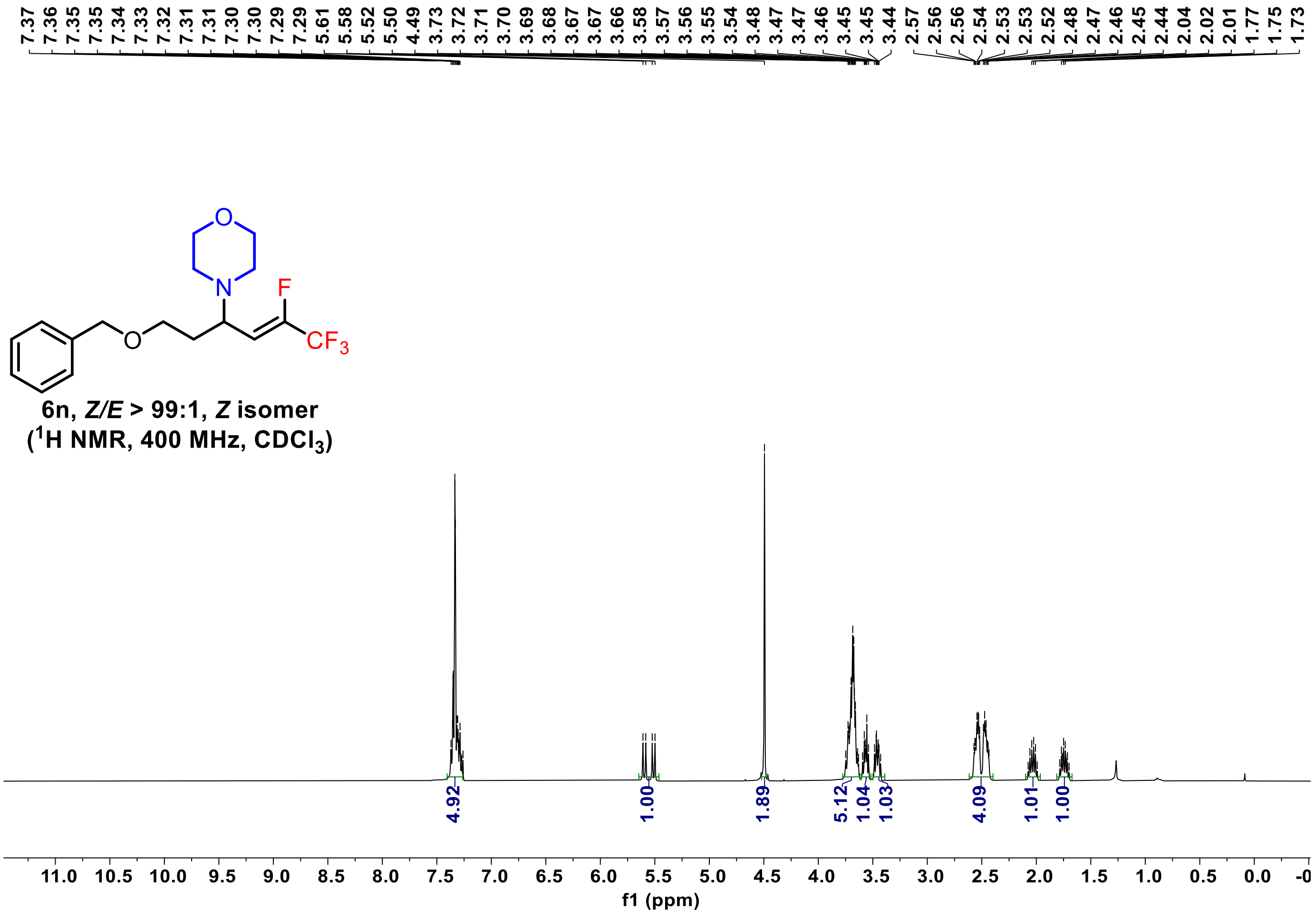
7.37 7.36 7.35 7.35 7.34 7.33 7.32 7.31 7.31 7.30 7.30 7.29 7.29 5.61 5.58 5.52 5.50 4.49 3.73 3.72 3.71 3.70 3.69 3.68 3.67 3.67 3.66 3.58 3.57 3.56 3.55 3.54 3.48 3.47 3.47 3.46 3.45 3.45 3.44 2.57 2.56 2.56 2.54 2.53 2.53 2.52 2.48 2.47 2.46 2.45 2.44 2.04 2.02 2.01 1.77 1.75 1.73
O
N
F
O
CF3
6n, Z/E > 99:1, Z isomer
(1H NMR, 400 MHz, CDCl3)
4.92 1.00 1.89 5.12 1.04 1.03 4.09 1.01 1.00
f1 (ppm)

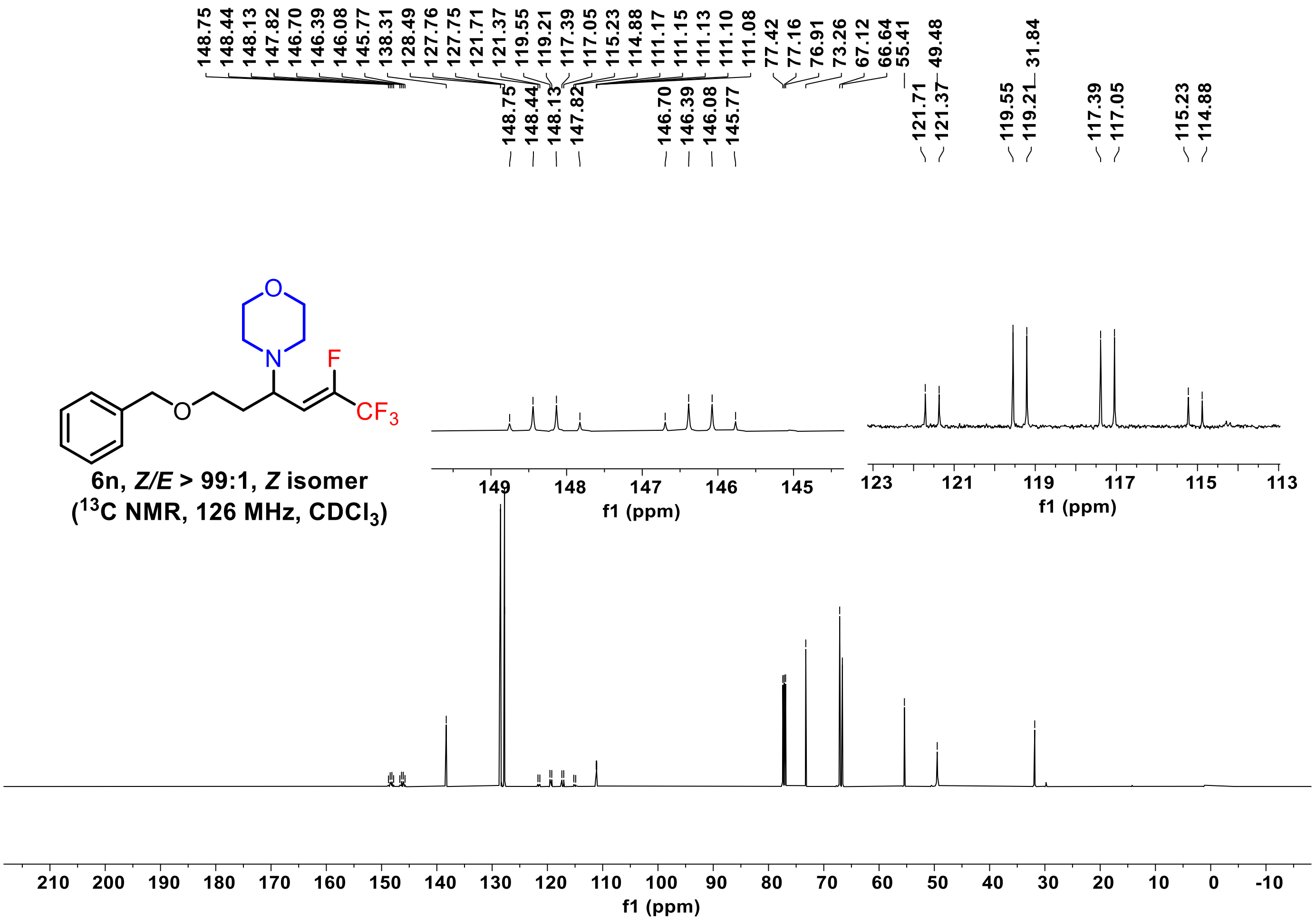
148.75 148.44 148.13 147.82 146.70 146.39 146.08 145.77 138.31 128.49 127.76 127.75 121.71 121.37 119.55 119.21 117.39 117.05 115.23 114.88 111.17 111.15 111.13 111.10 111.08 77.42 77.16 76.91 73.26 67.12 66.64 55.41 49.48 31.84
148.75 148.44 148.13 147.82 146.70 146.39 146.08 145.77
121.71 121.37 119.55 119.21 117.39 117.05 115.23 114.88
O
N
F
O
CF3
6n, Z/E > 99:1, Z isomer
(13C NMR, 126 MHz, CDCl3)
f1 (ppm)

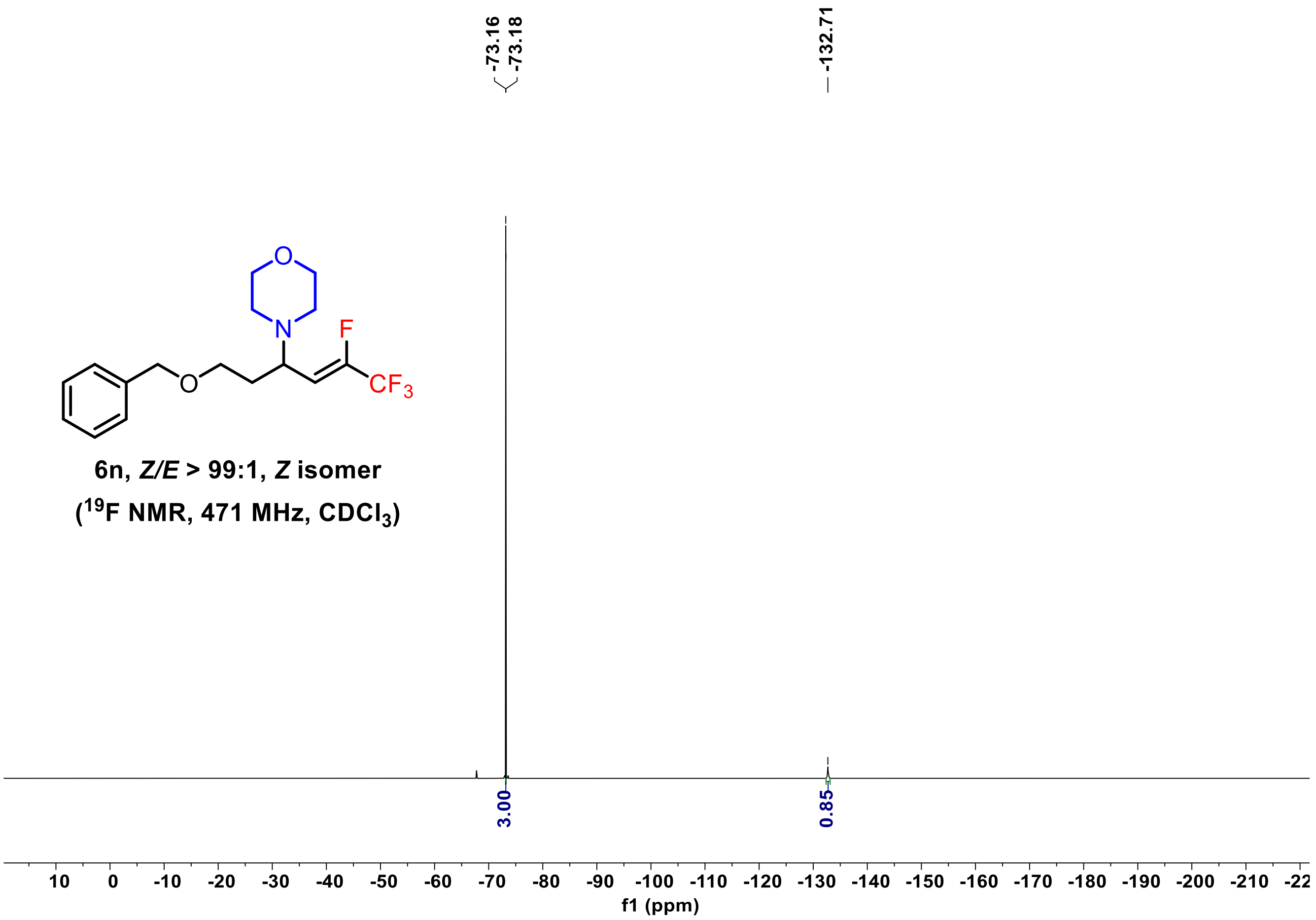
-73.16
-73.18
-132.71
6n, Z/E > 99:1, Z isomer
(19F NMR, 471 MHz, CDCl3)
3.00
0.85
f1 (ppm)

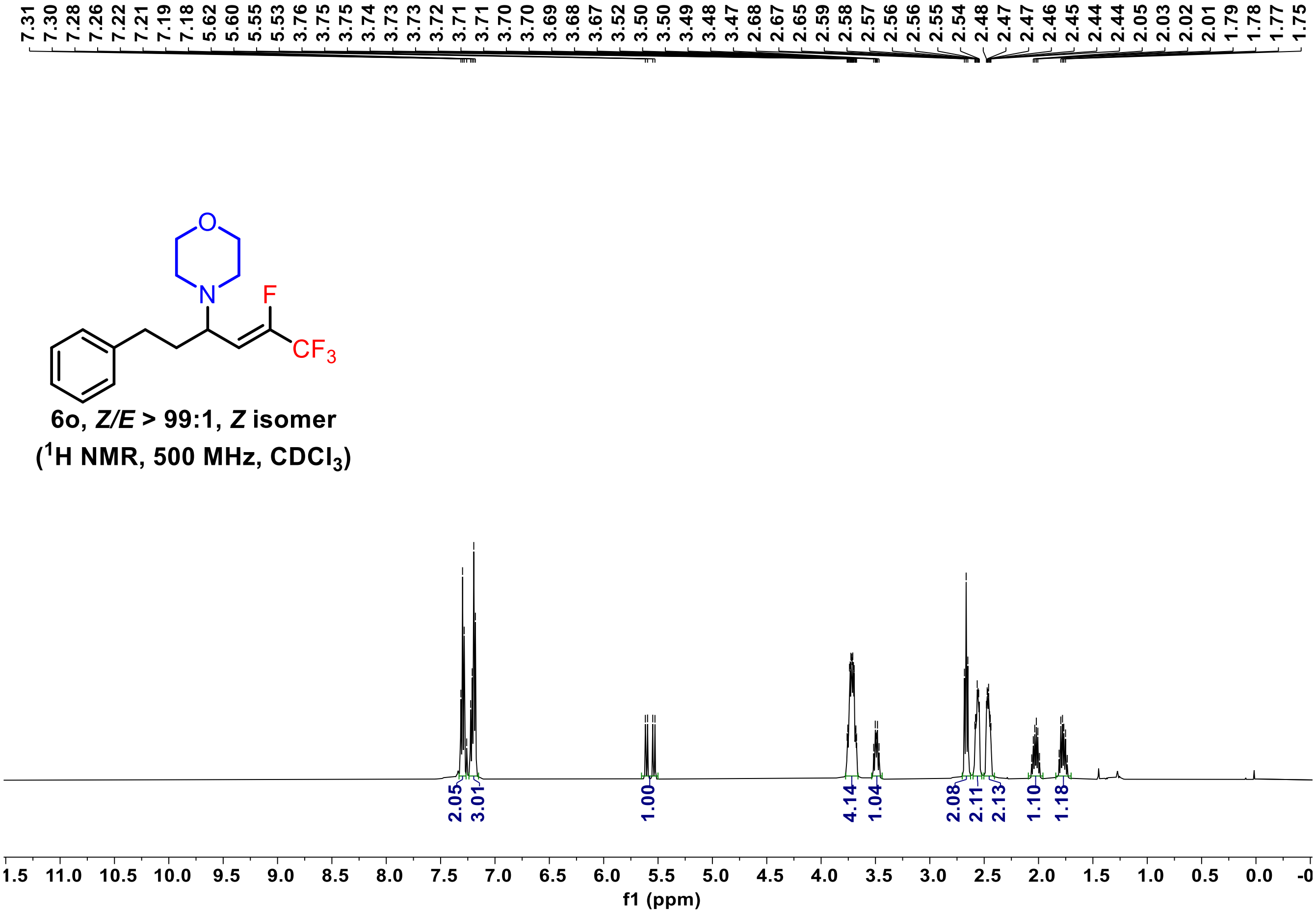
7.31 7.30 7.28 7.26 7.22 7.21 7.19 7.18 5.62 5.60 5.55 5.53 3.76 3.75 3.75 3.74 3.73 3.73 3.72 3.71 3.71 3.70 3.70 3.69 3.68 3.67 3.52 3.50 3.50 3.49 3.48 3.47 2.68 2.67 2.65 2.59 2.58 2.57 2.56 2.56 2.55 2.54 2.48 2.47 2.47 2.46 2.45 2.44 2.44 2.05 2.03 2.02 2.01 1.79 1.78 1.77 1.75
6o, Z/E > 99:1, Z isomer
(1H NMR, 500 MHz, CDCl3)
2.05
3.01
1.00
4.14
1.04
2.08
2.11
2.13
1.10
1.18
f1 (ppm)

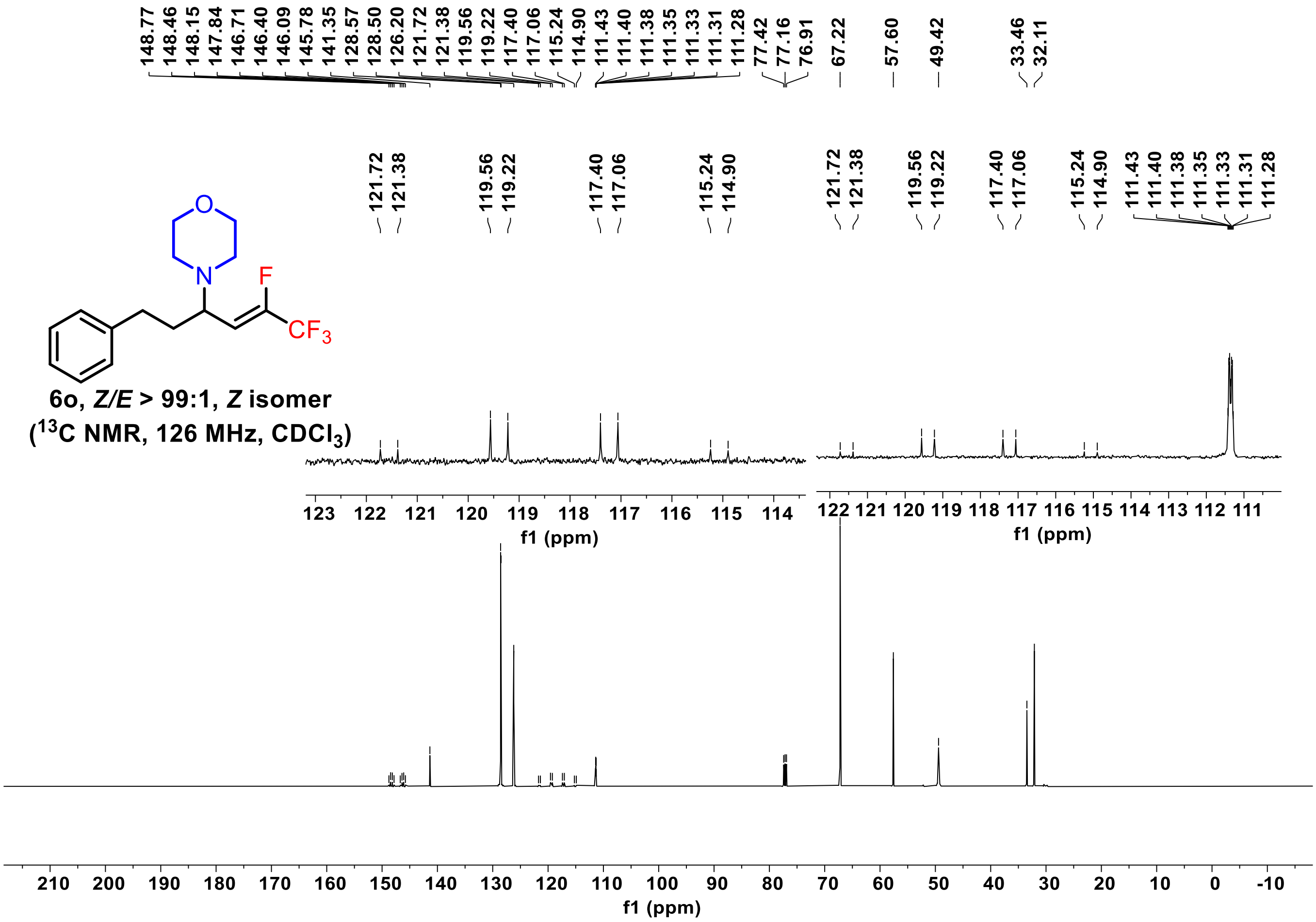
148.77 148.46 148.15 147.84 146.71 146.40 146.09 145.78 141.35 128.57 128.50 126.20 121.72 121.38 119.56 119.22 117.40 117.06 115.24 114.90 111.43 111.40 111.38 111.35 111.33 111.31 111.28 77.42 77.16 76.91 67.22 57.60 49.42 33.46 32.11
6o, Z/E > 99:1, Z isomer
(^{13}C NMR, 126 MHz, $CDCl_3$)
f1 (ppm)

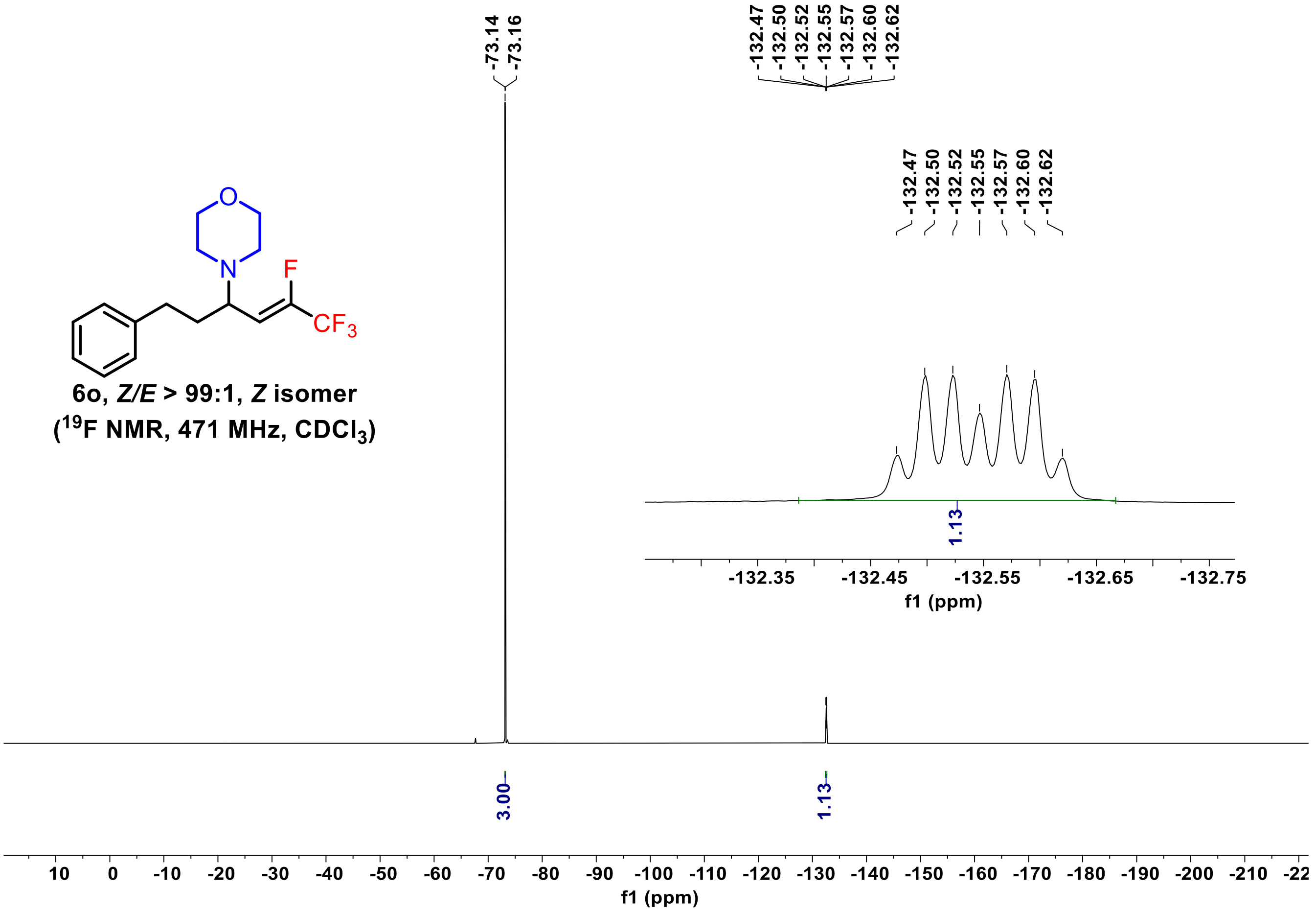
-73.14 -73.16
-132.47 -132.50 -132.52 -132.55 -132.57 -132.60 -132.62
6o, Z/E > 99:1, Z isomer
(^{19}F NMR, 471 MHz, $CDCl_3$)
3.00
1.13
f1 (ppm)

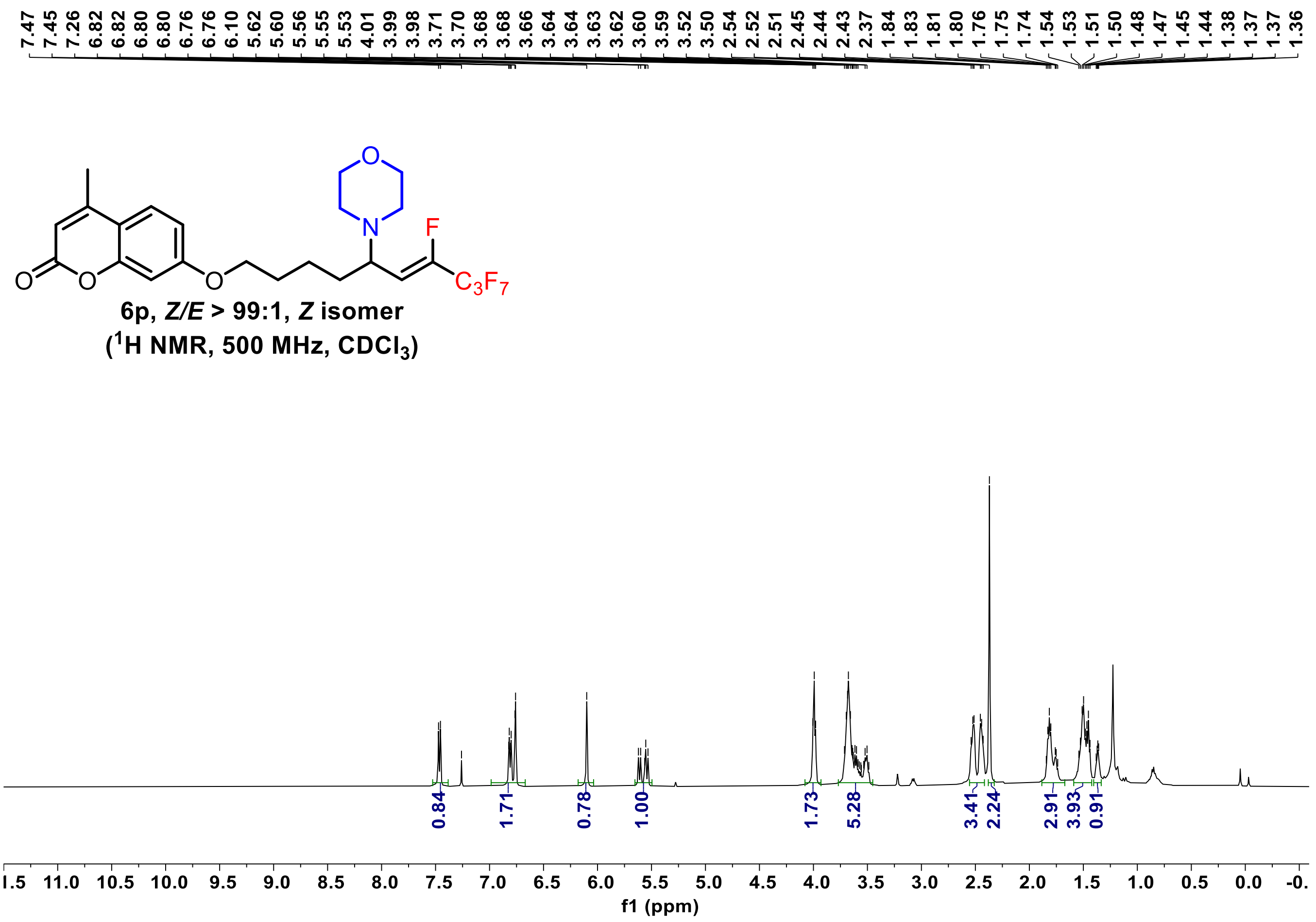
7.47 7.45 7.26 6.82 6.82 6.80 6.80 6.76 6.76 6.10 5.62 5.60 5.56 5.55 5.53 4.01 3.99 3.98 3.71 3.70 3.68 3.68 3.66 3.64 3.64 3.63 3.62 3.60 3.59 3.52 3.50 2.54 2.52 2.51 2.45 2.44 2.43 2.37 1.84 1.83 1.81 1.80 1.76 1.75 1.74 1.54 1.53 1.51 1.50 1.48 1.47 1.45 1.44 1.38 1.37 1.37 1.36
O
N
F
O
O
O
C3F7
6p, Z/E > 99:1, Z isomer
(1H NMR, 500 MHz, CDCl3)
0.84
1.71
0.78
1.00
1.73
5.28
3.41
2.24
2.91
3.93
0.91
f1 (ppm)

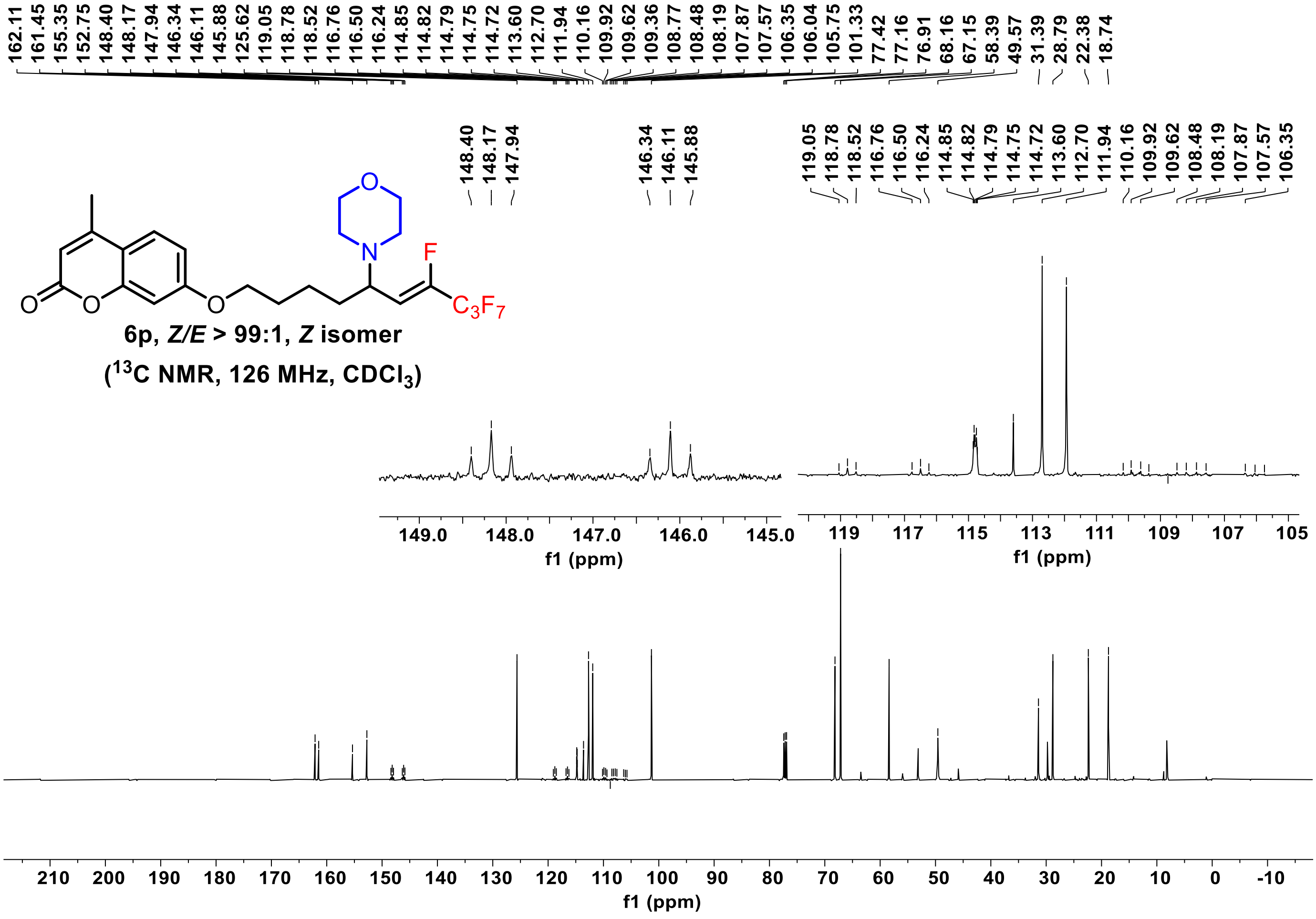
162.11 161.45 155.35 152.75 148.40 148.17 147.94 146.34 146.11 145.88 125.62 119.05 118.78 118.52 116.76 116.50 116.24 114.85 114.82 114.79 114.75 114.72 113.60 112.70 111.94 110.16 109.92 109.62 109.36 108.77 108.48 108.19 107.87 107.57 106.35 106.04 105.75 101.33 77.42 77.16 76.91 68.16 67.15 58.39 49.57 31.39 28.79 22.38 18.74
148.40 148.17 147.94
146.34 146.11 145.88
119.05 118.78 118.52 116.76 116.50 116.24 114.85 114.82 114.79 114.75 114.72 113.60 112.70 111.94 110.16 109.92 109.62 108.48 108.19 107.87 107.57 106.35
O
N
F
O
O
O
C3F7
6p, Z/E > 99:1, Z isomer
(13C NMR, 126 MHz, CDCl3)
f1 (ppm)
f1 (ppm)
f1 (ppm)

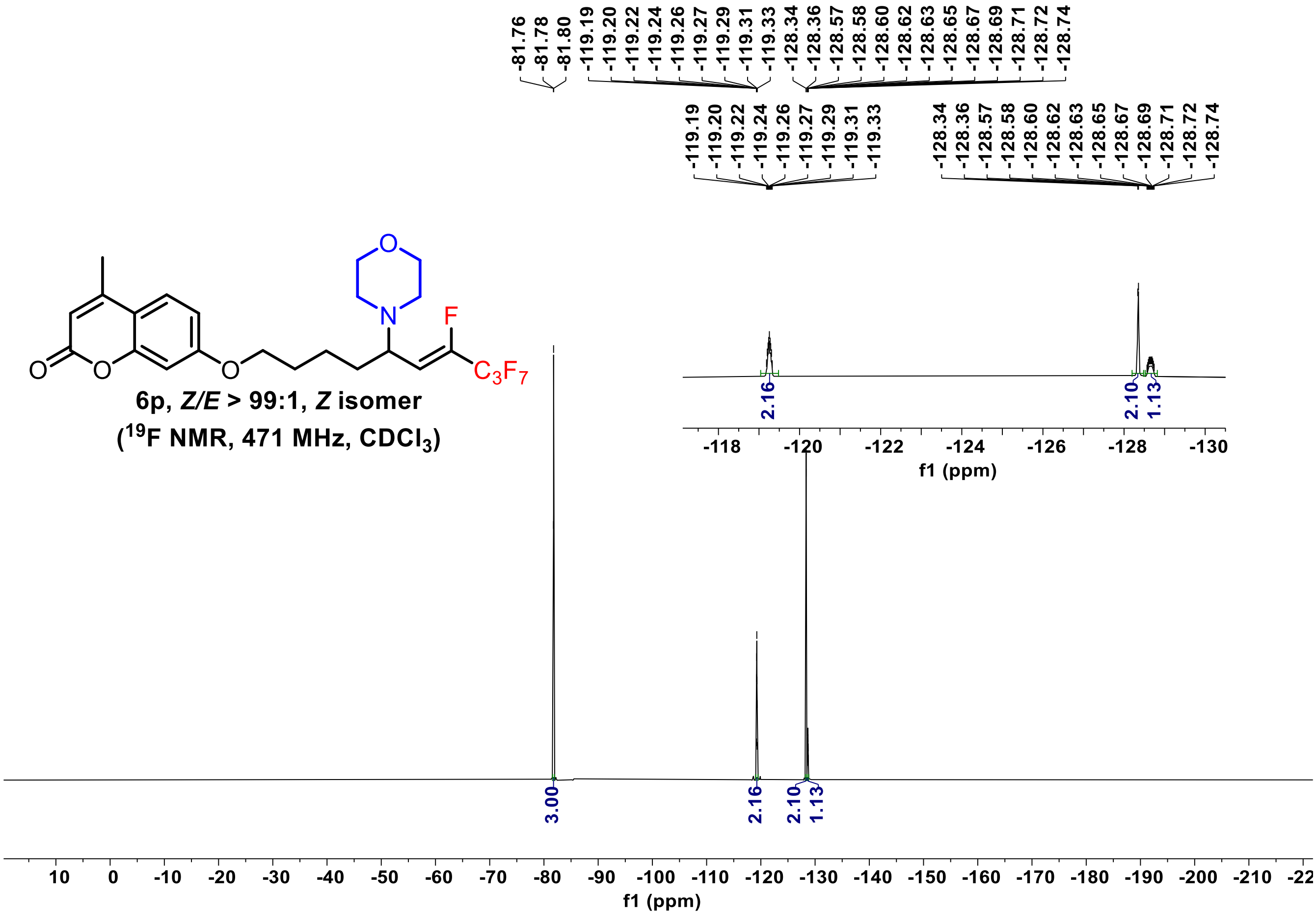
-81.76
-81.78
-81.80
-119.19
-119.20
-119.22
-119.24
-119.26
-119.27
-119.29
-119.31
-119.33
-128.34
-128.36
-128.57
-128.58
-128.60
-128.62
-128.63
-128.65
-128.67
-128.69
-128.71
-128.72
-128.74
6p, Z/E > 99:1, Z isomer
(19F NMR, 471 MHz, CDCl3)
3.00
2.16
2.10
1.13
f1 (ppm)

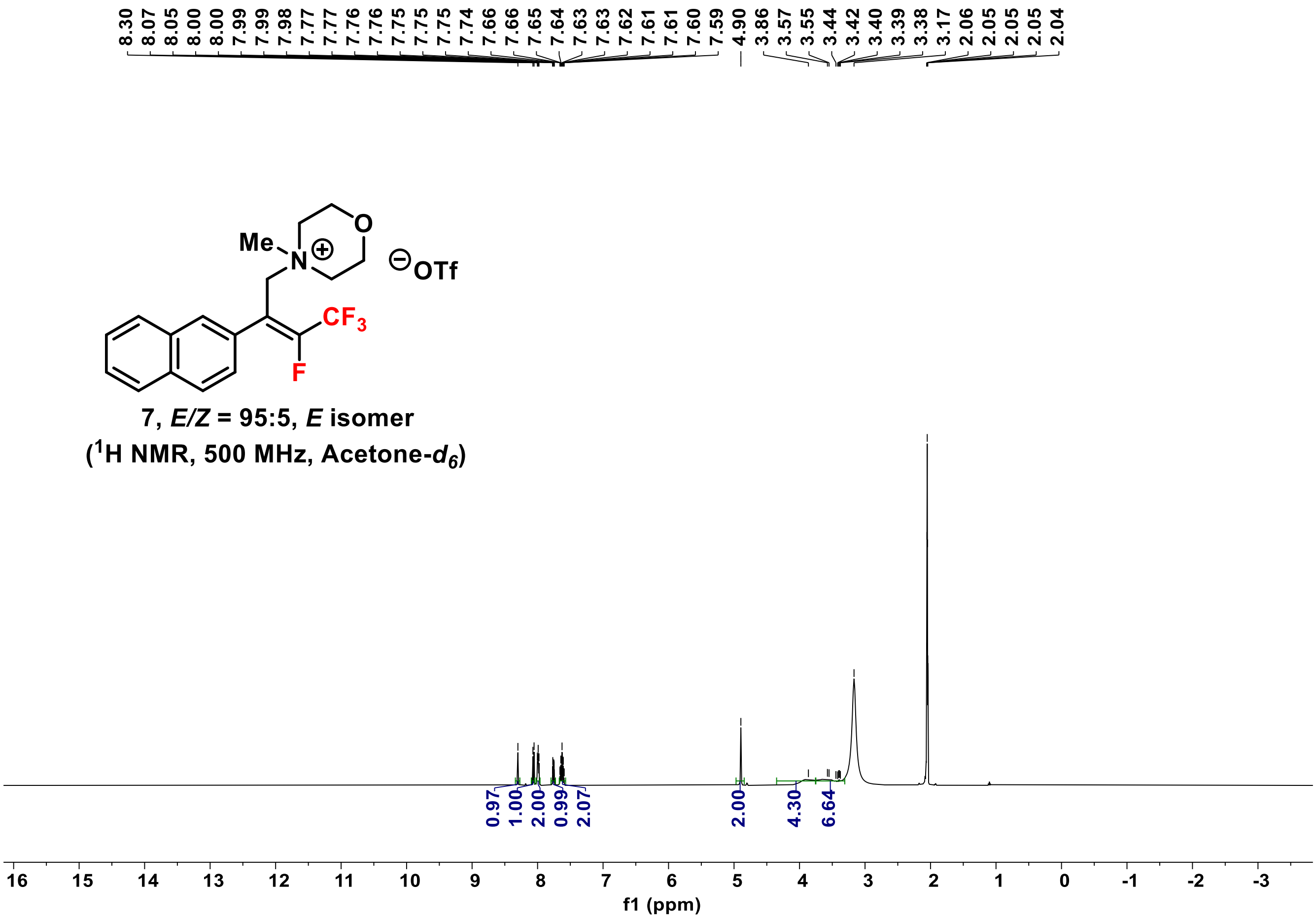
8.30
8.07
8.05
8.00
8.00
7.99
7.99
7.98
7.77
7.77
7.76
7.76
7.75
7.75
7.74
7.66
7.66
7.65
7.64
7.63
7.62
7.61
7.61
7.60
7.59
4.90
3.86
3.57
3.55
3.44
3.42
3.40
3.39
3.38
3.17
2.06
2.05
2.05
2.05
2.04
Me
OTf
CF3
F
7, E/Z = 95:5, E isomer
(1H NMR, 500 MHz, Acetone-d6)
0.97
1.00
2.00
0.99
2.07
2.00
4.30
6.64
f1 (ppm)

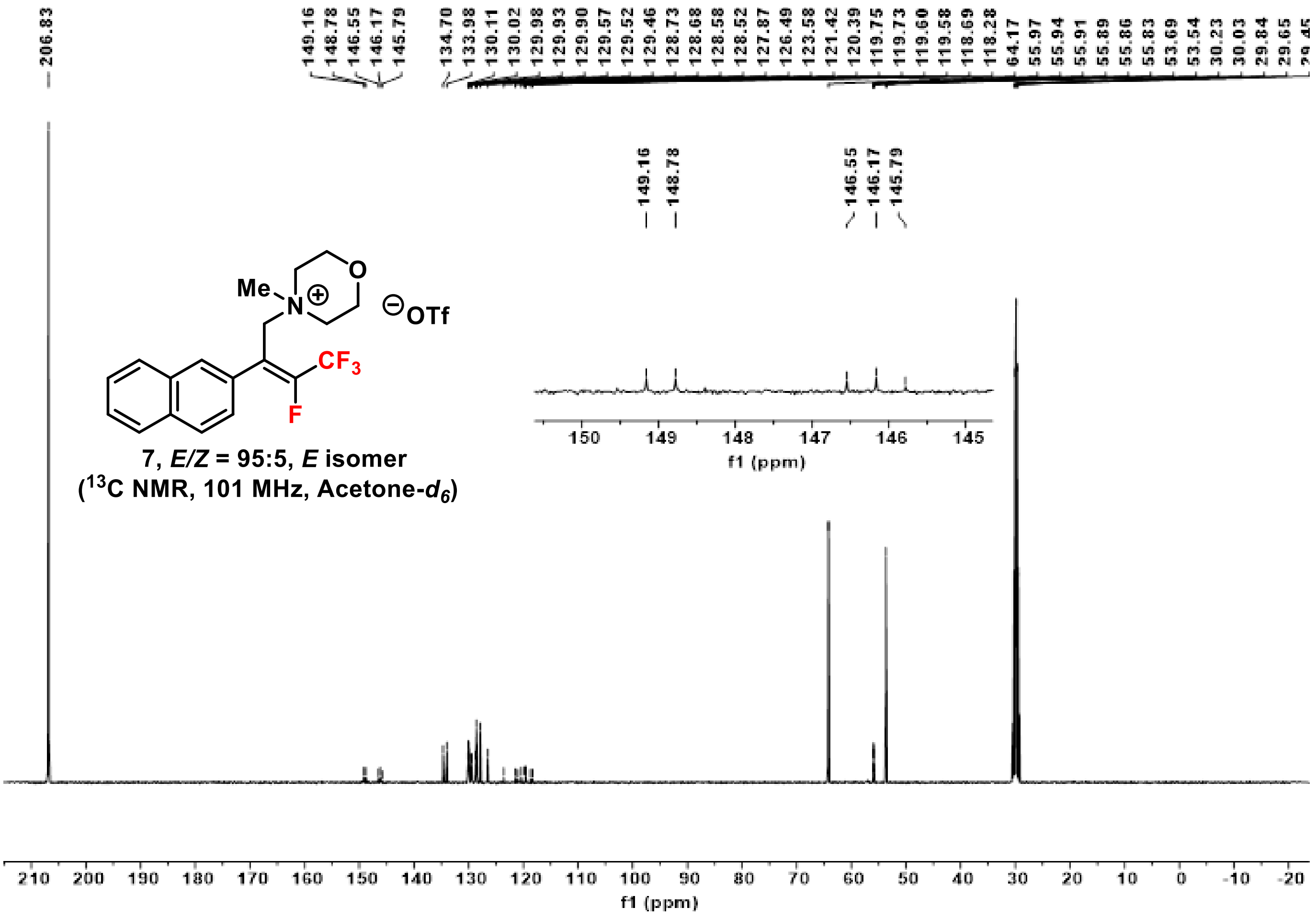
Me
N
O
⊖OTf
CF3
F
7, E/Z = 95:5, E isomer
(13C NMR, 101 MHz, Acetone-d6)
f1 (ppm)

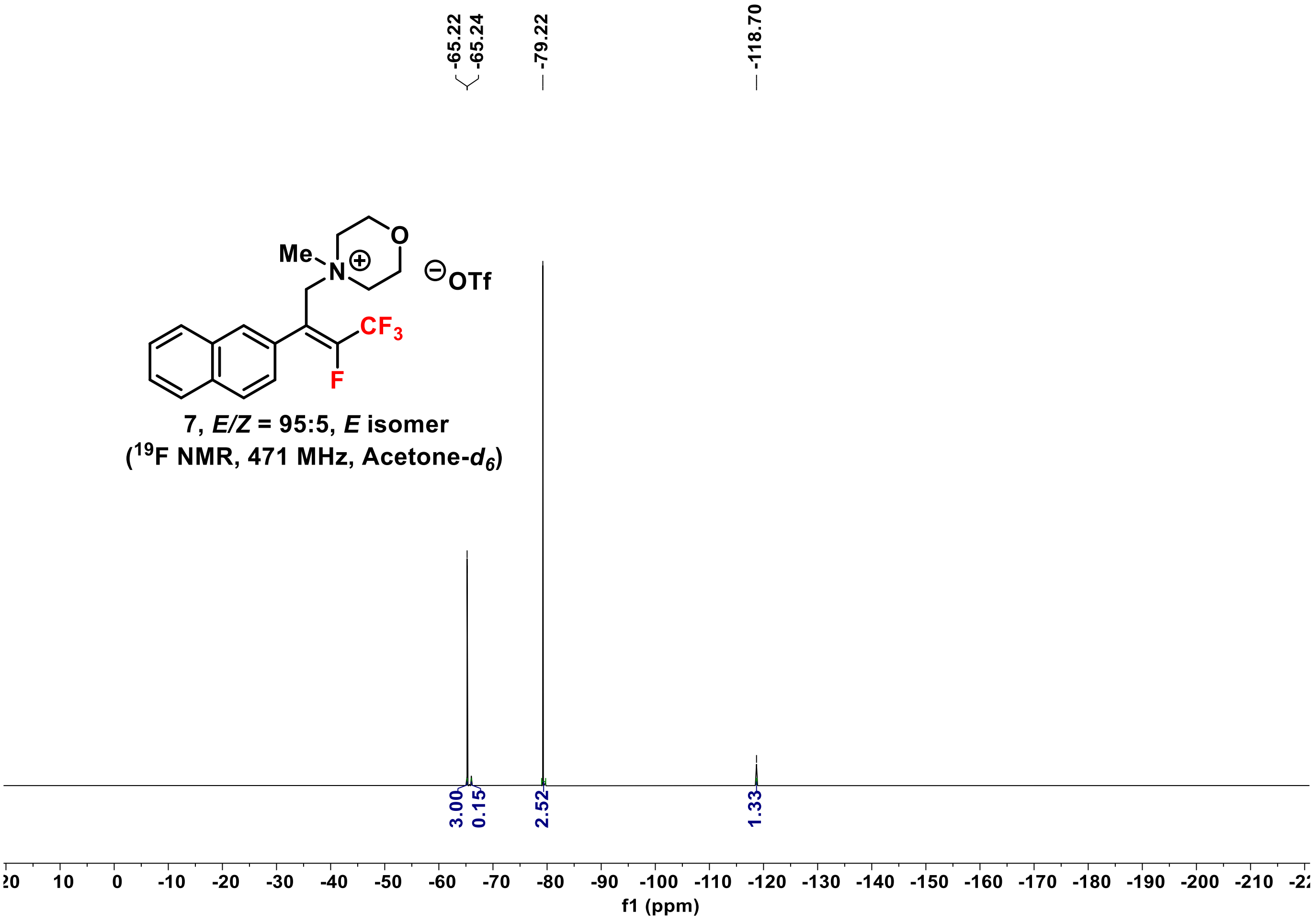
-65.22
-65.24
-79.22
-118.70
Me
N
O
⊖OTf
CF3
F
7, E/Z = 95:5, E isomer
(19F NMR, 471 MHz, Acetone-d6)
3.00
0.15
2.52
1.33
f1 (ppm)

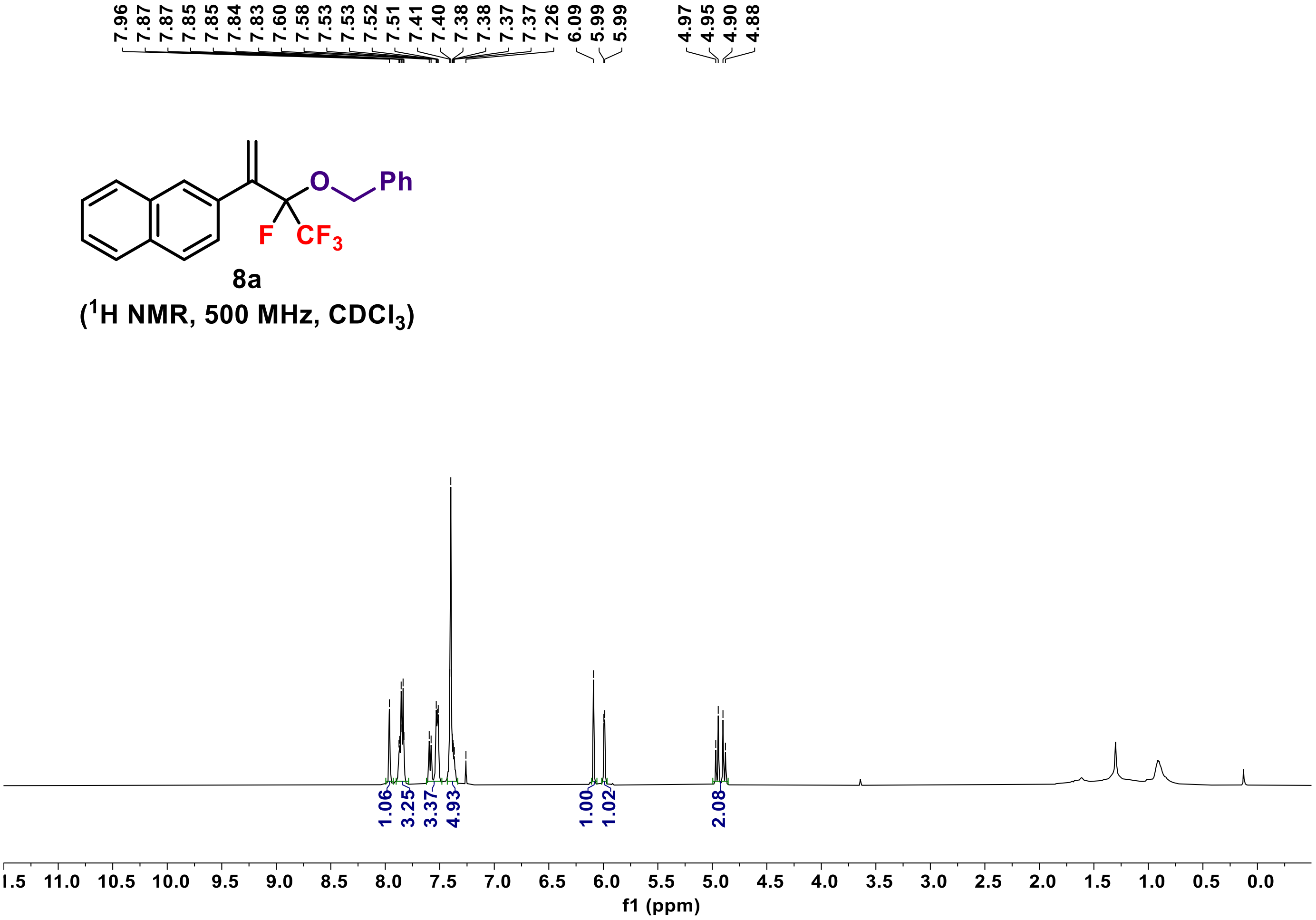

8a

(^{1}H NMR, 500 MHz, $CDCl_3$)

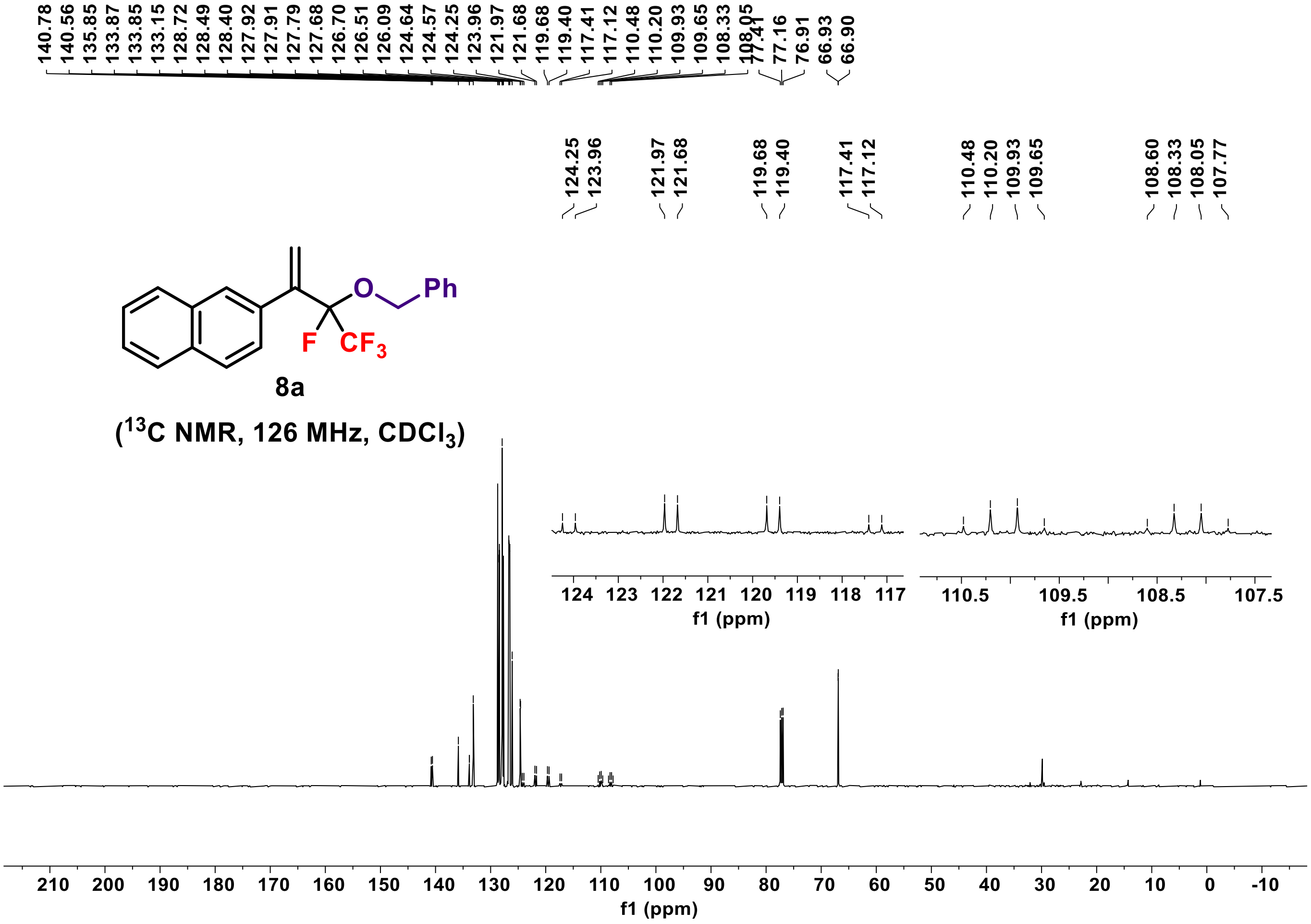

8a

(^{13}C NMR, 126 MHz, $CDCl_3$)

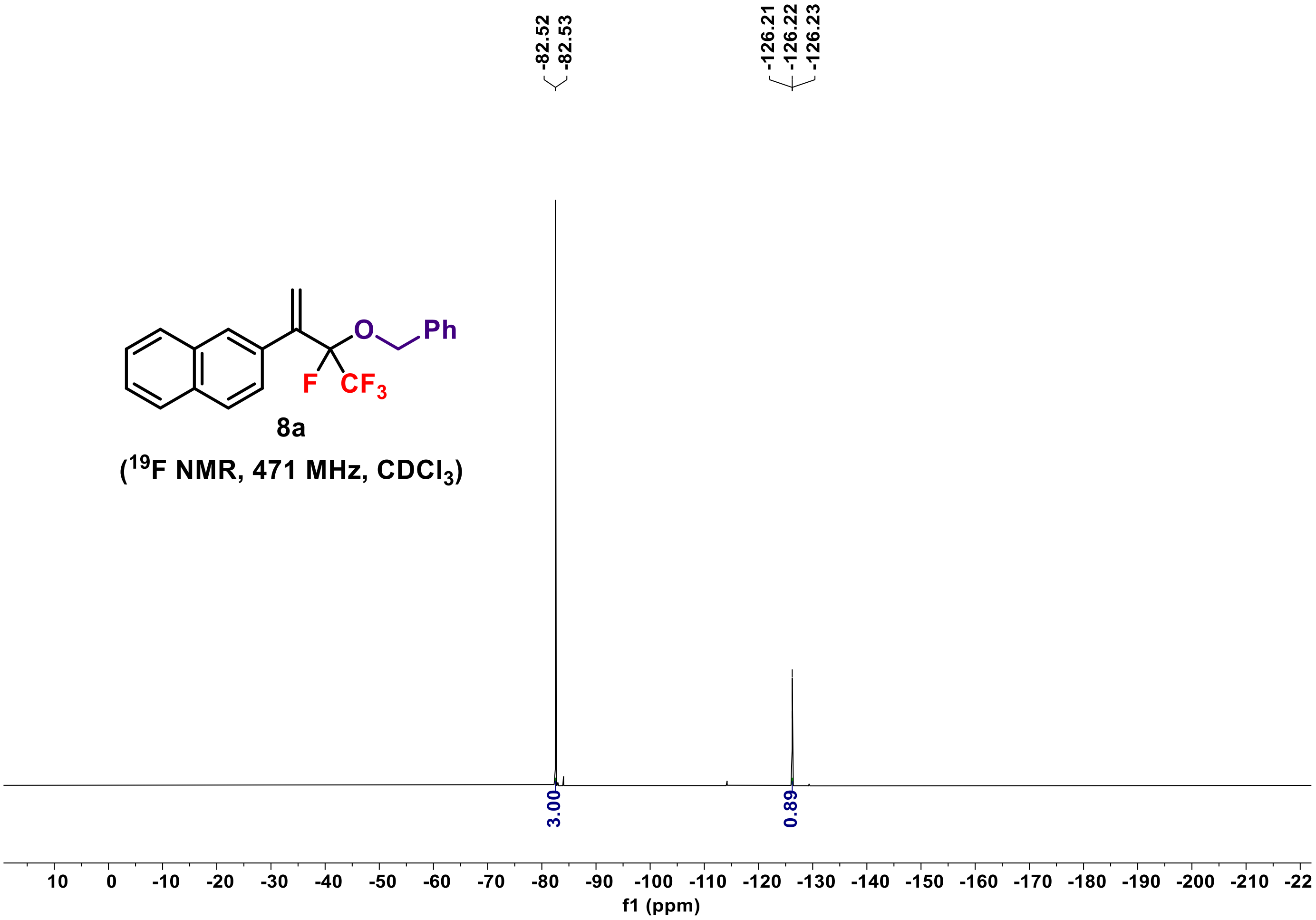

8a

(^{19}F NMR, 471 MHz, $CDCl_3$)

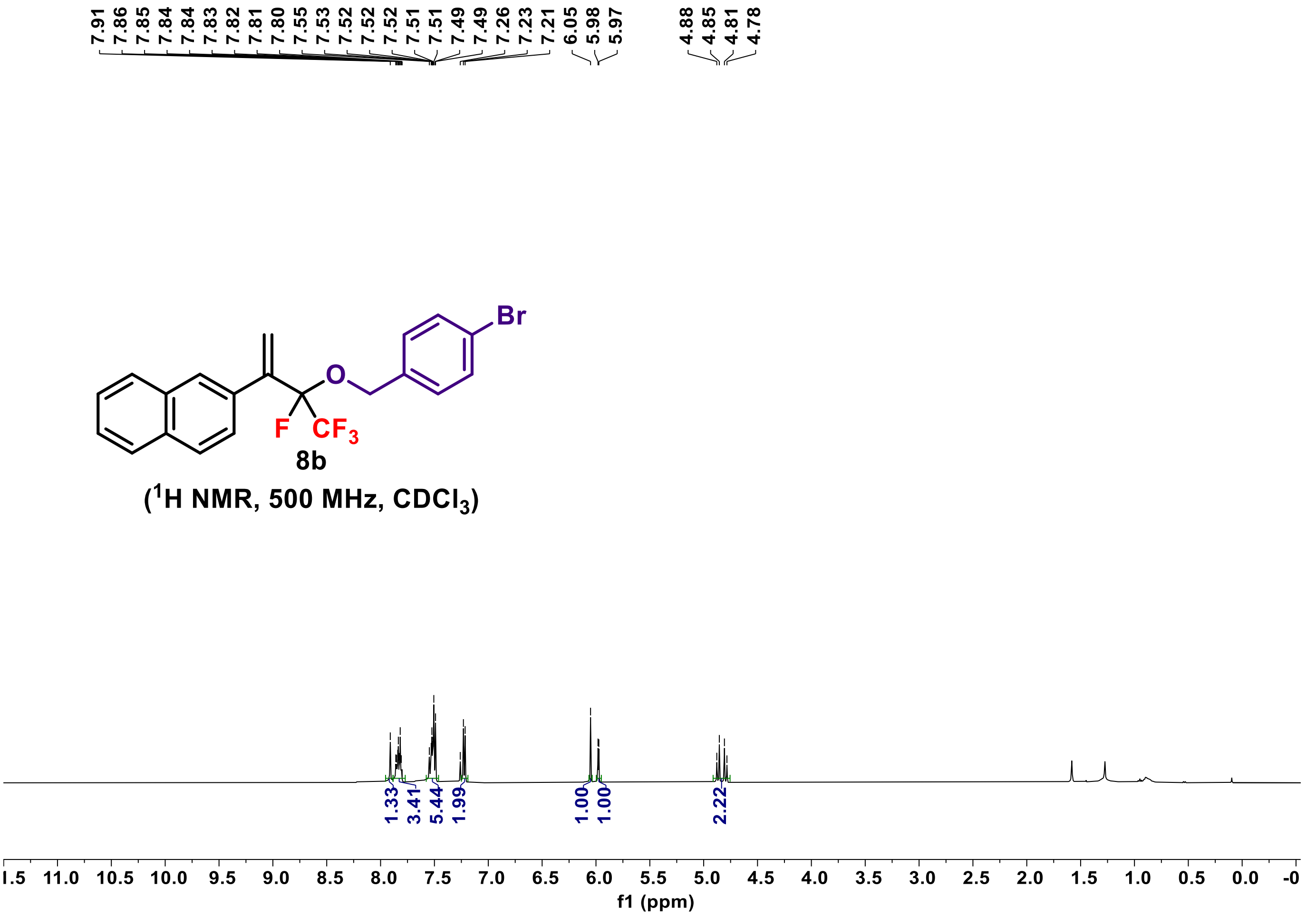

8b

(^{1}H NMR, 500 MHz, $CDCl_3$)

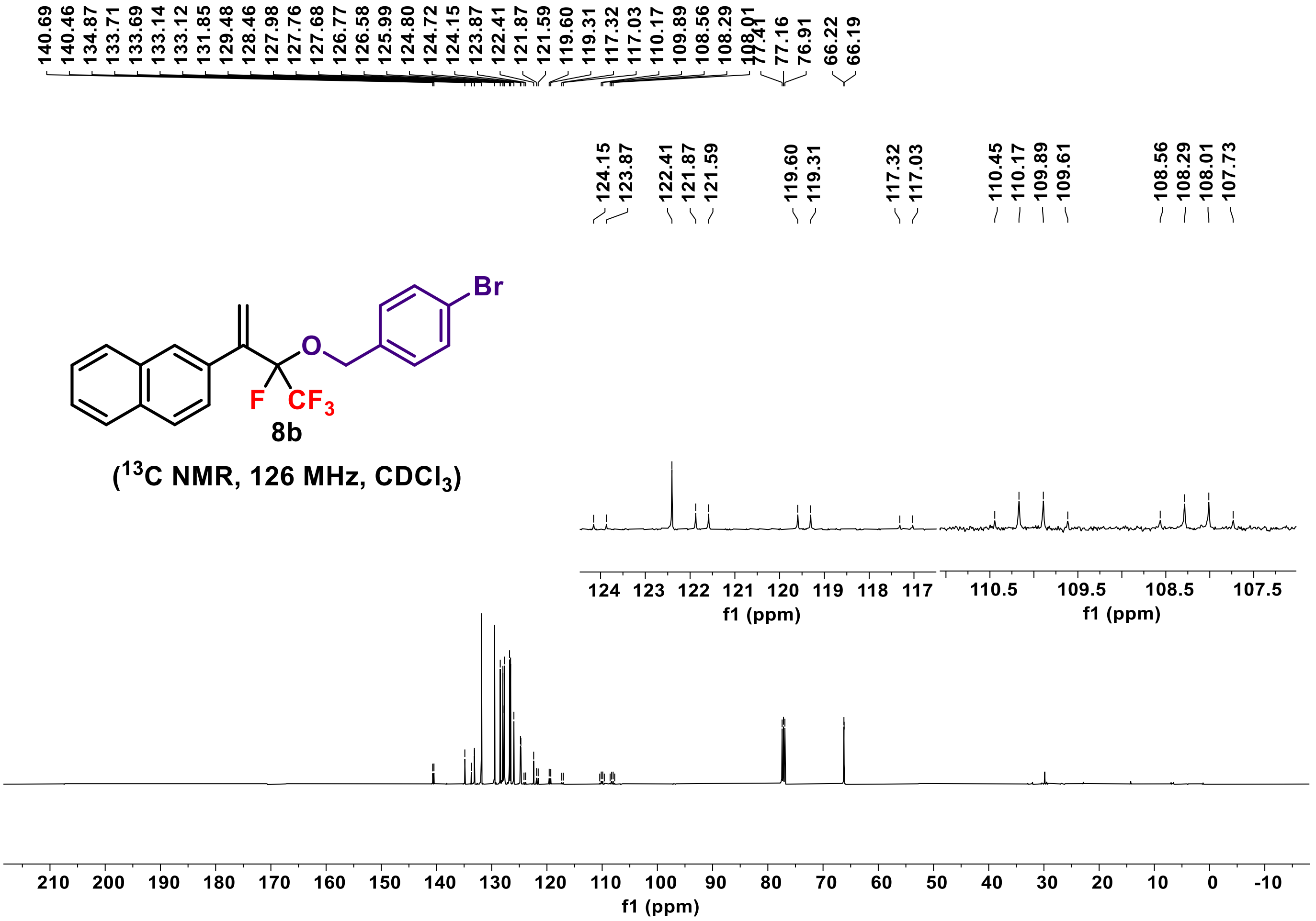

8b

(^{13}C NMR, 126 MHz, $CDCl_3$)

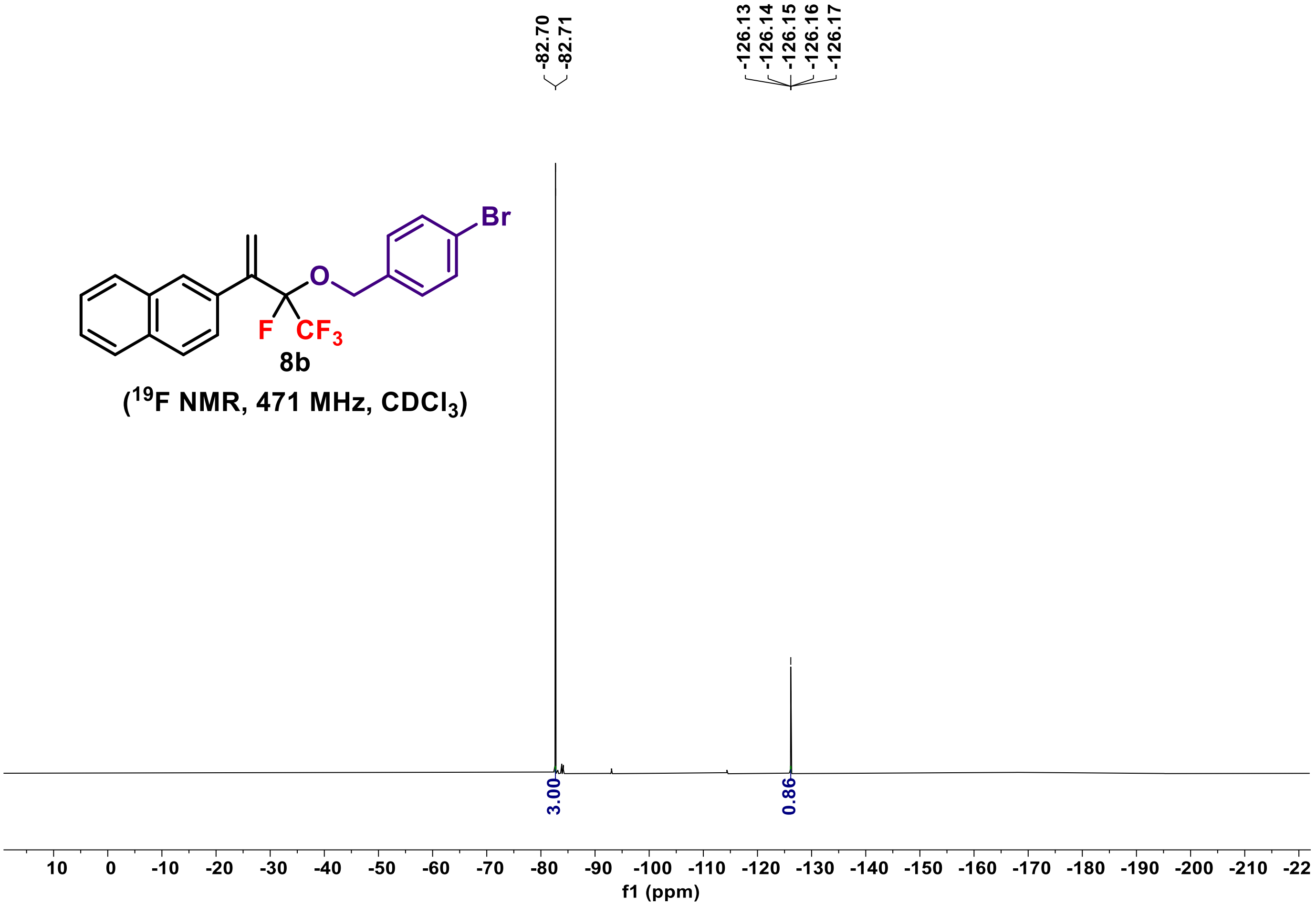

8b

(^{19}F NMR, 471 MHz, $CDCl_3$)

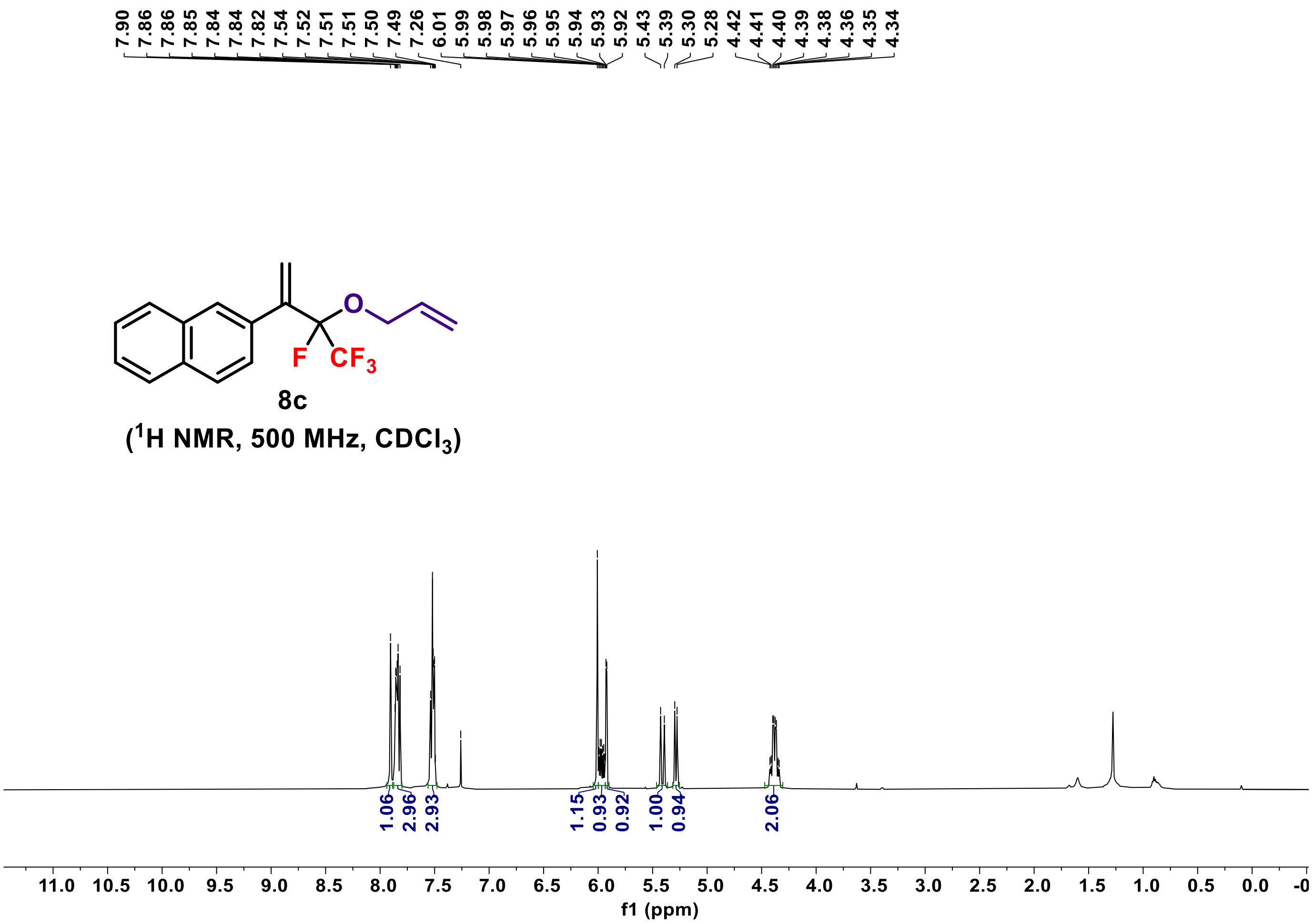

8c

(^{1}H NMR, 500 MHz, CDCl$_3$)

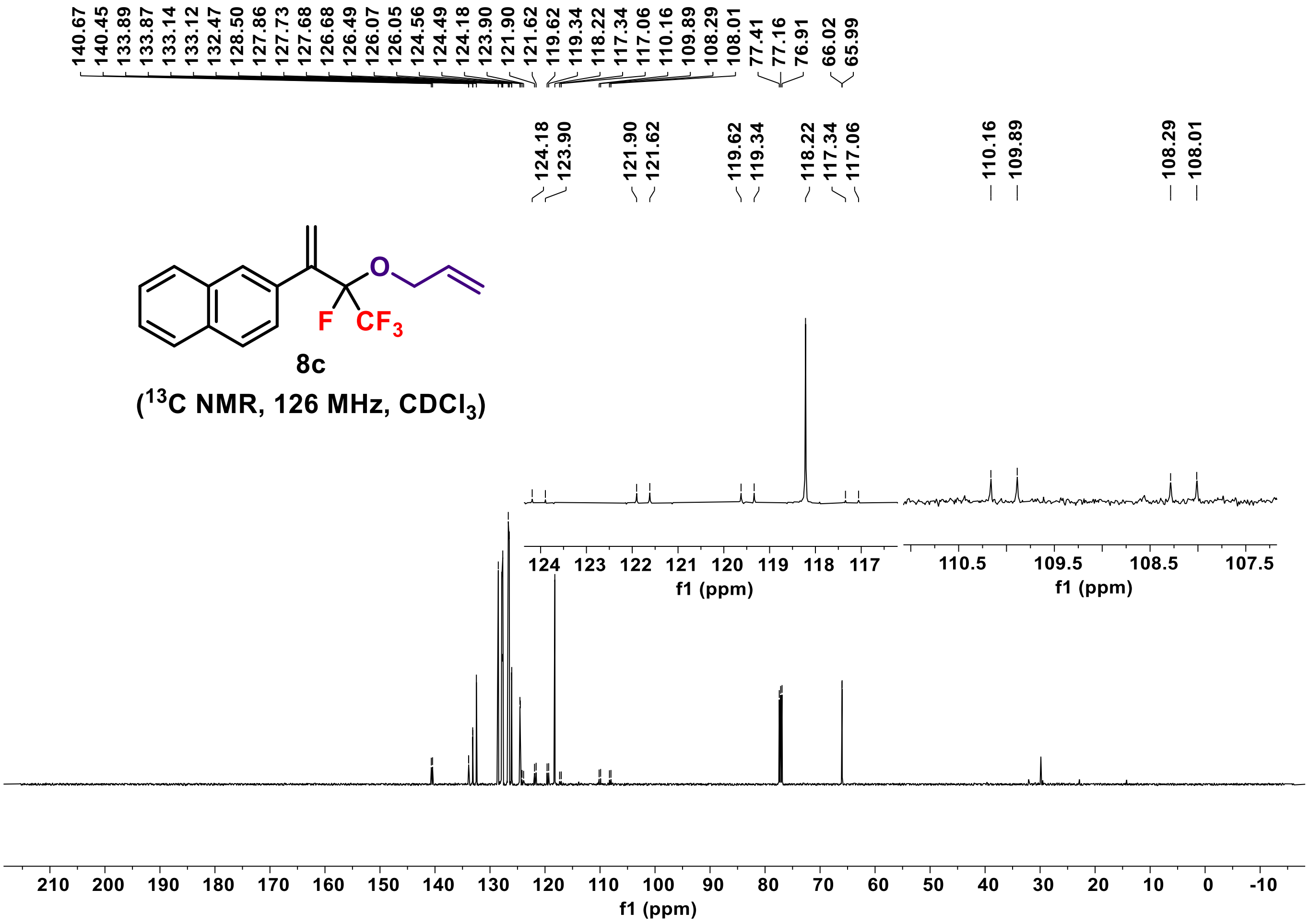

8c

(^{13}C NMR, 126 MHz, CDCl$_3$)

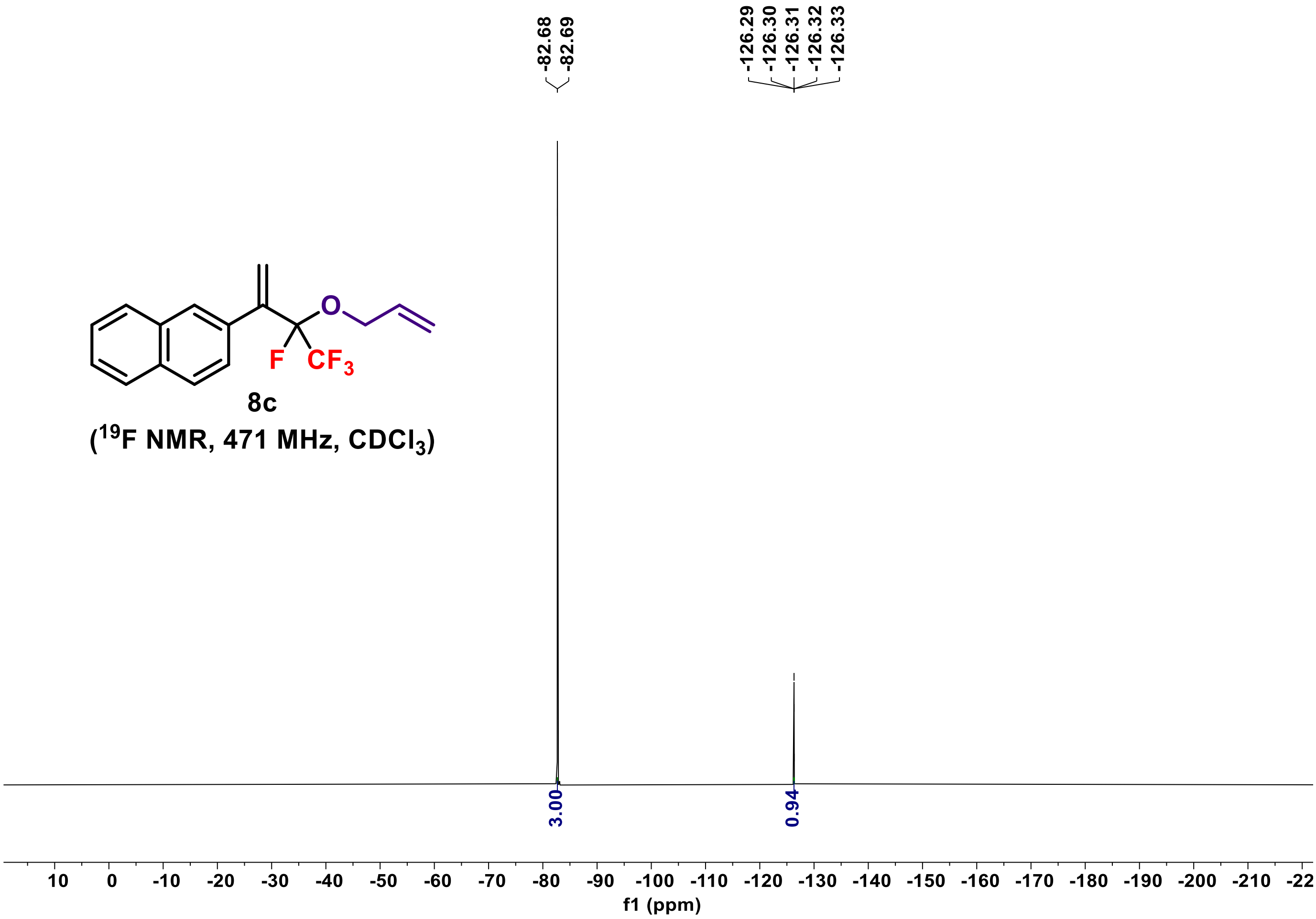

8c

(^{19}F NMR, 471 MHz, $CDCl_3$)

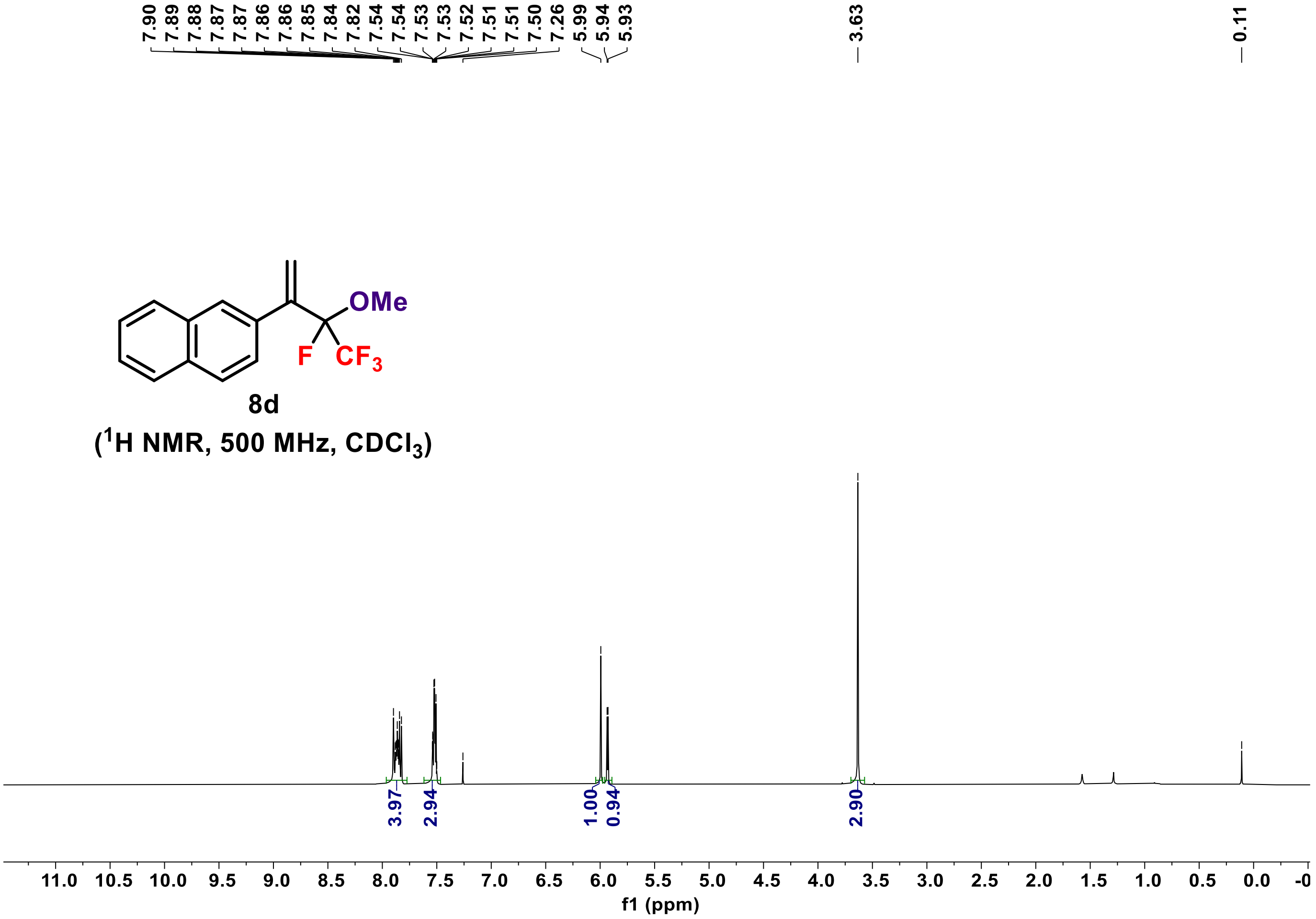

8d

(^{1}H NMR, 500 MHz, $CDCl_3$)

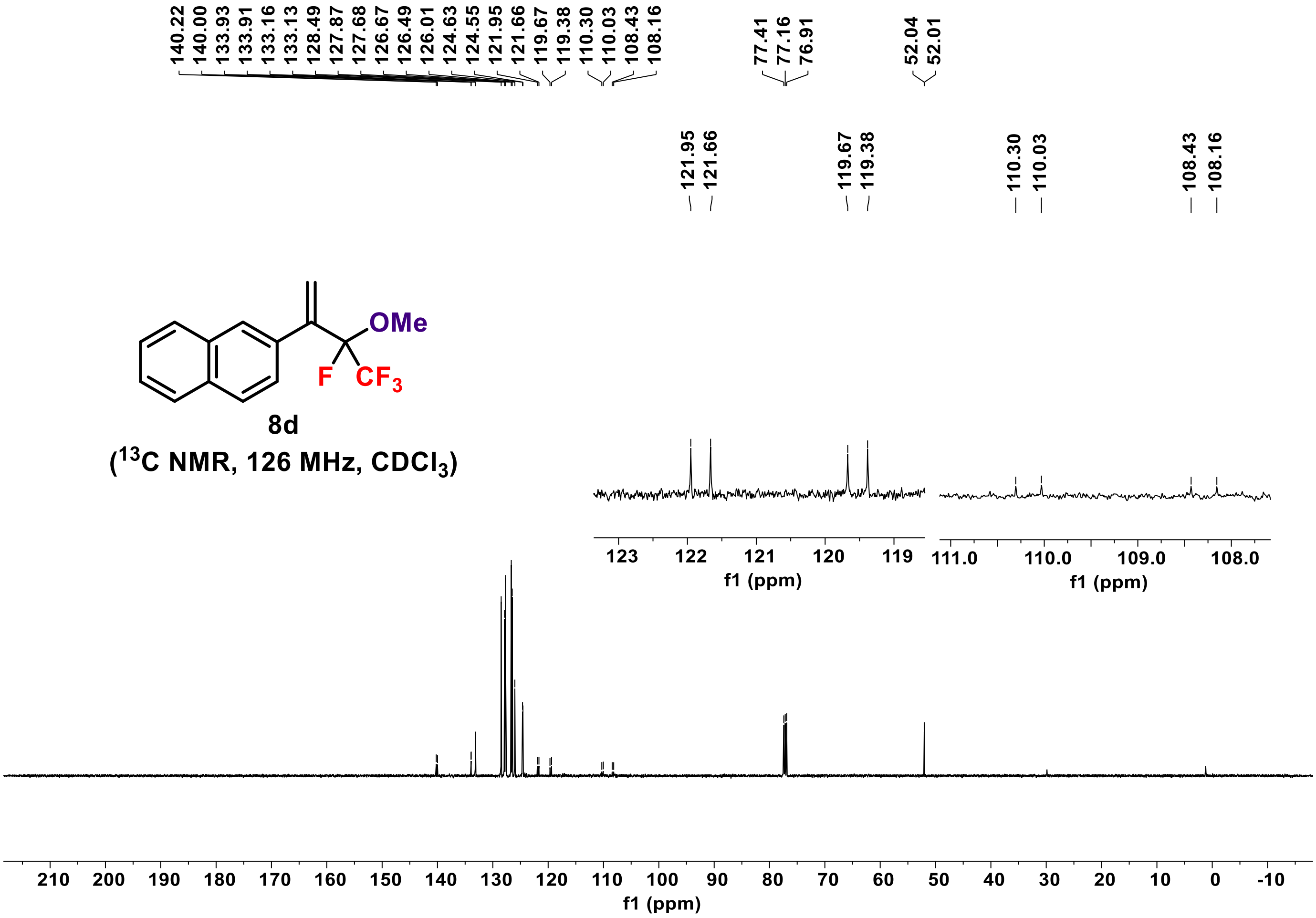
140.22
140.00
133.93
133.91
133.16
133.13
128.49
127.87
127.68
126.67
126.49
126.01
124.63
124.55
121.95
121.66
119.67
119.38
110.30
110.03
108.43
108.16
77.41
77.16
76.91
52.04
52.01
OMe
F
CF3
8d
(13C NMR, 126 MHz, CDCl3)
f1 (ppm)

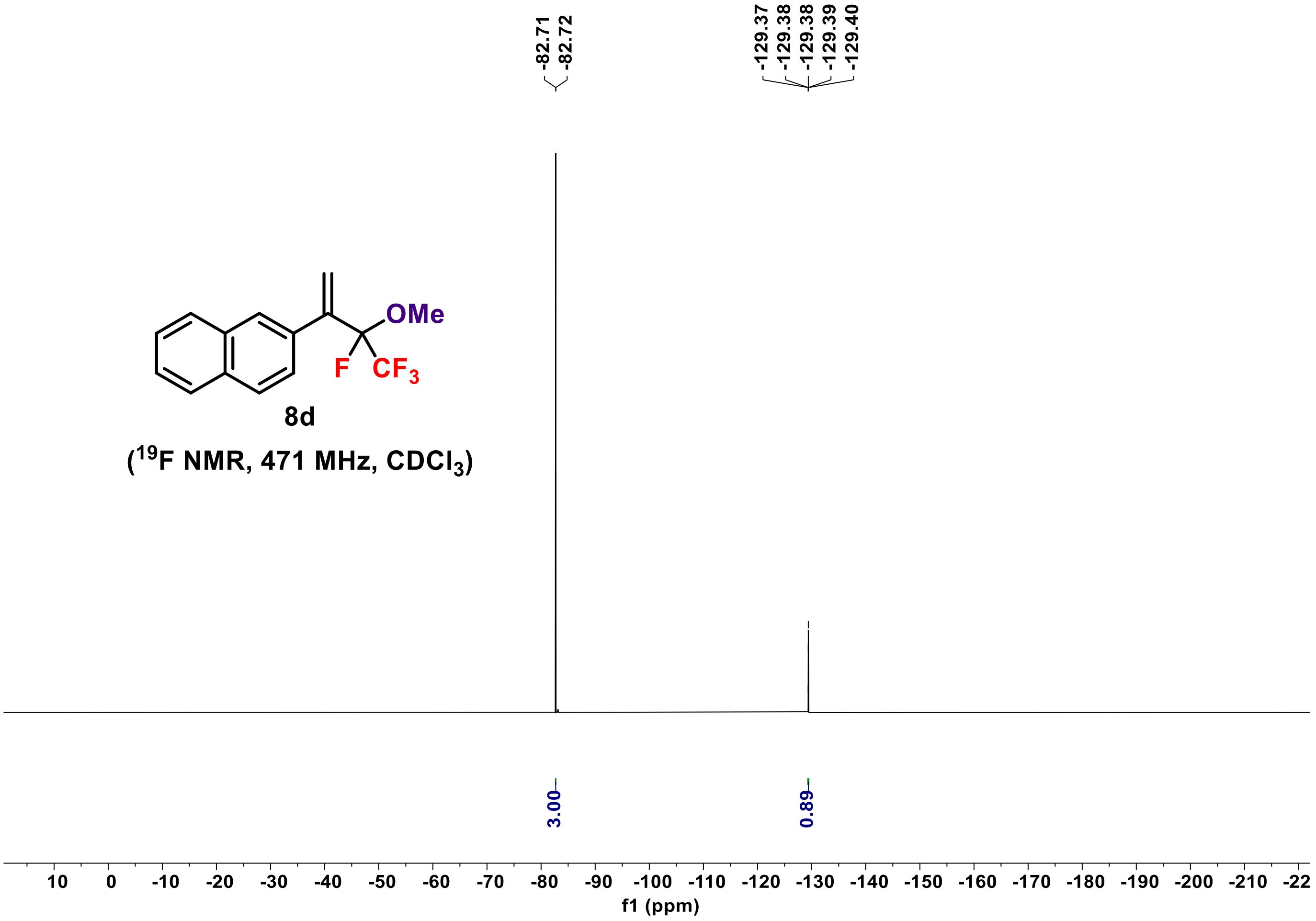
-82.71
-82.72
-129.37
-129.38
-129.38
-129.39
-129.40
OMe
F
CF3
8d
(19F NMR, 471 MHz, CDCl3)
3.00
0.89
f1 (ppm)

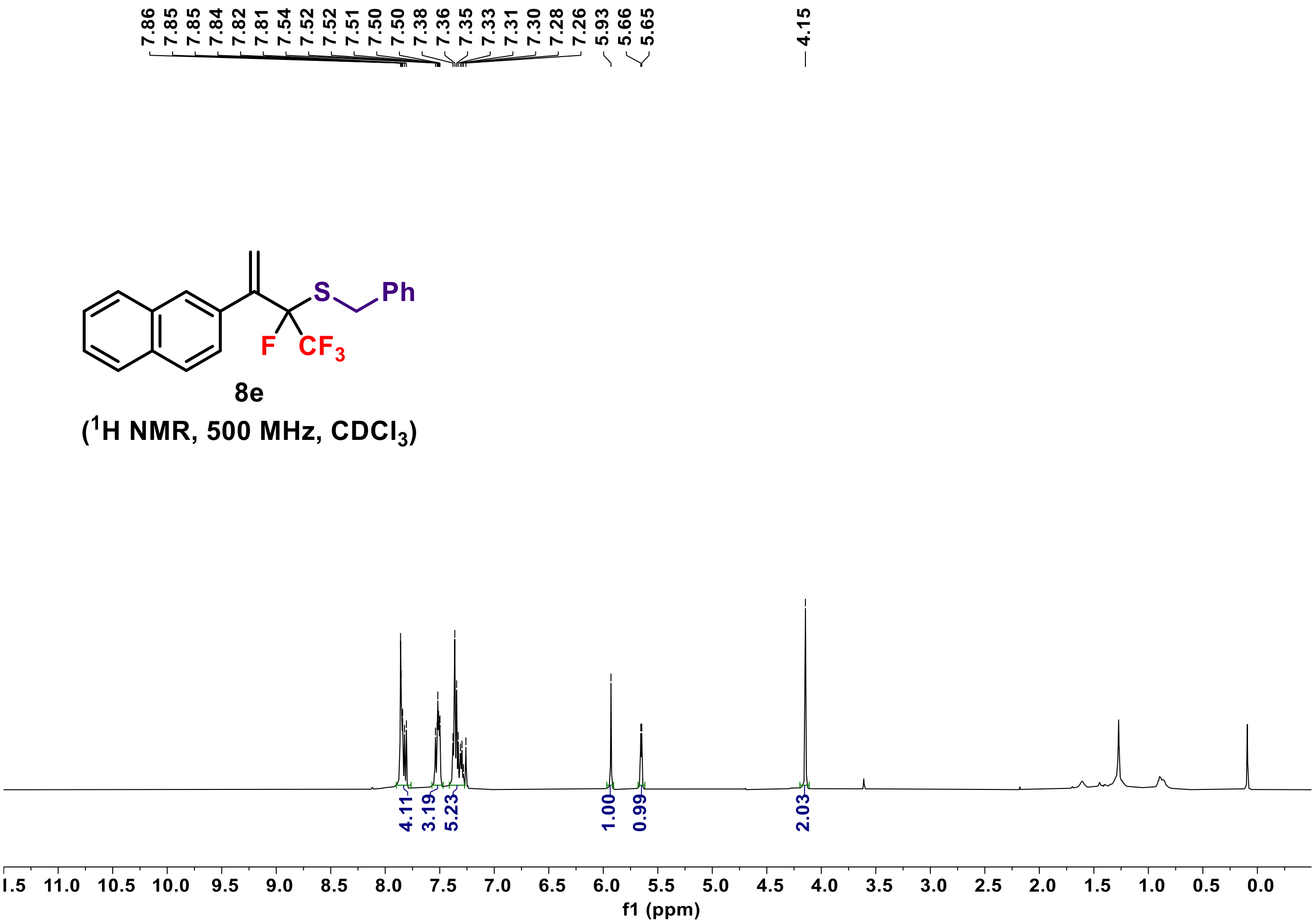
7.86 7.85 7.85 7.84 7.82 7.81 7.54 7.52 7.52 7.51 7.50 7.50 7.38 7.36 7.35 7.33 7.31 7.30 7.28 7.26 5.93 5.66 5.65
4.15
S
Ph
F
CF3
8e
(1H NMR, 500 MHz, CDCl3)
4.11 3.19 5.23 1.00 0.99 2.03
f1 (ppm)

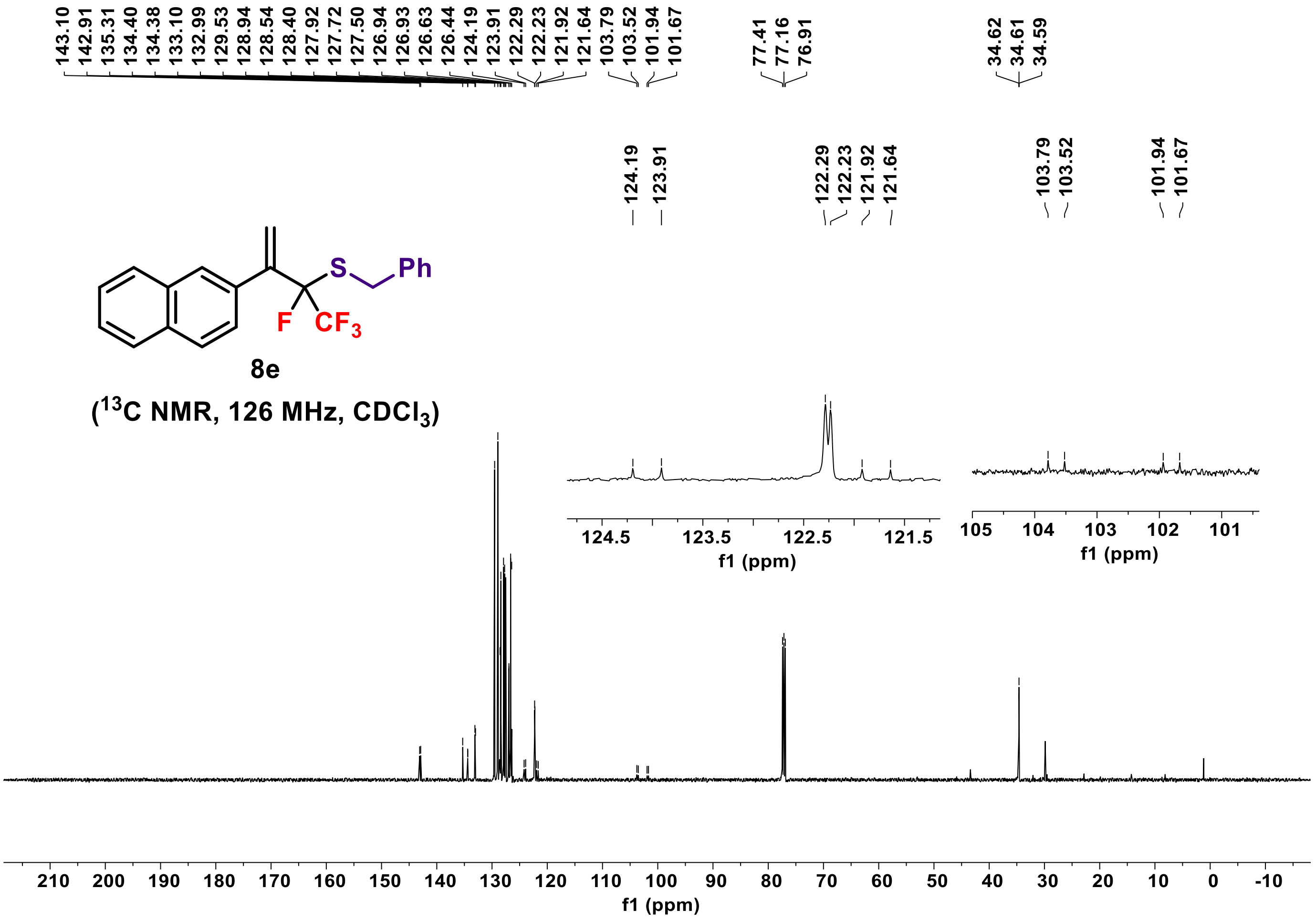
143.10 142.91 135.31 134.40 134.38 133.10 132.99 129.53 128.94 128.54 128.40 127.92 127.72 127.50 126.94 126.93 126.63 126.44 124.19 123.91 122.29 122.23 121.92 121.64 103.79 103.52 101.94 101.67
77.41 77.16 76.91
34.62 34.61 34.59
124.19 123.91
122.29 122.23 121.92 121.64
103.79 103.52
101.94 101.67
S
Ph
F
CF3
8e
(13C NMR, 126 MHz, CDCl3)
f1 (ppm)

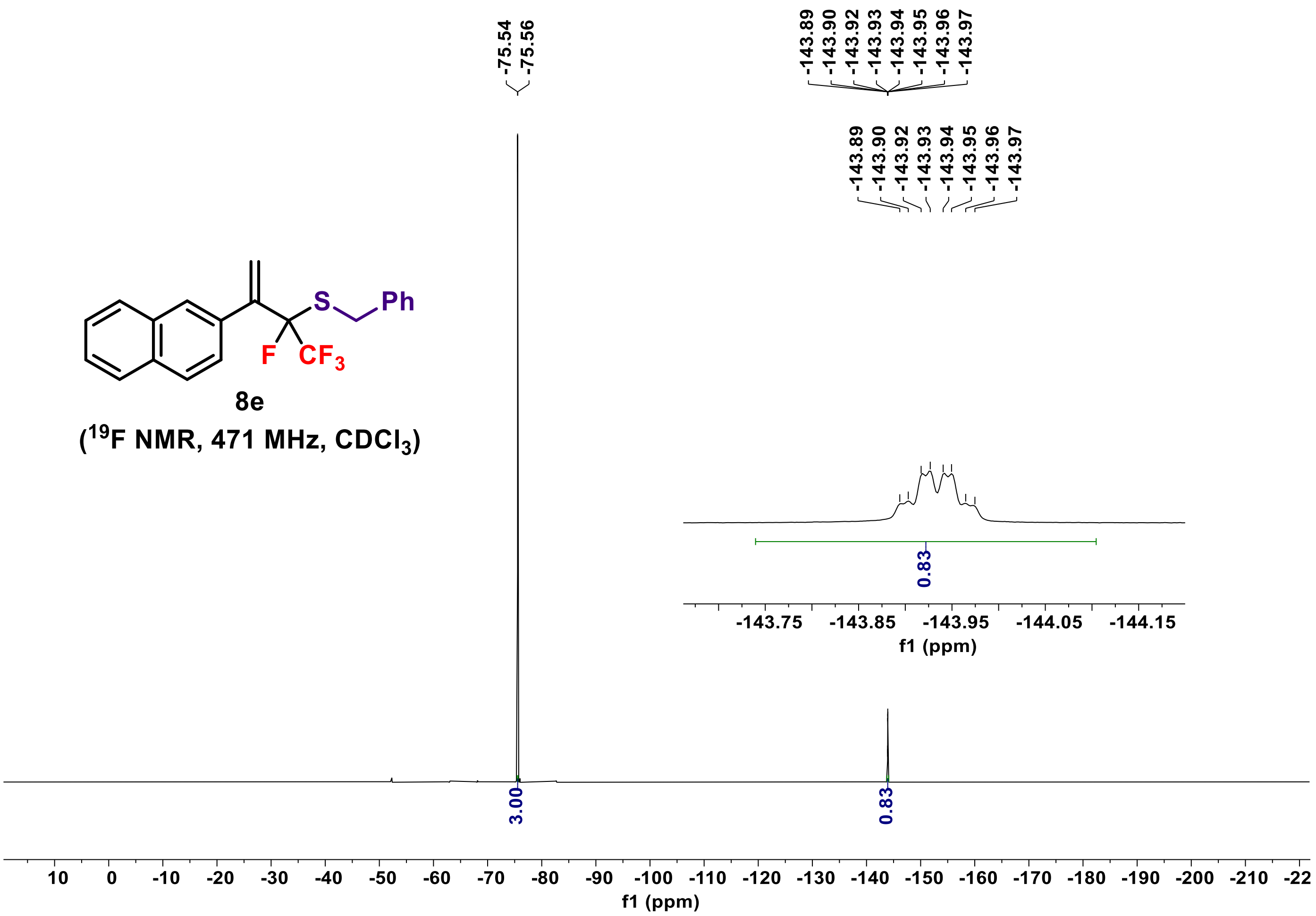

-75.54
-75.56
-143.89
-143.90
-143.92
-143.93
-143.94
-143.95
-143.96
-143.97
S
Ph
F
CF3
8e
(19F NMR, 471 MHz, CDCl3)
0.83
-143.75
-143.85
-143.95
-144.05
-144.15
f1 (ppm)
3.00
0.83
10
0
-10
-20
-30
-40
-50
-60
-70
-80
-90
-100
-110
-120
-130
-140
-150
-160
-170
-180
-190
-200
-210
-22
f1 (ppm)
